# Rapid Synthesis and Diversification of Thymine‐Containing Bridged Nucleic Acids Through Cascade Cyclization Reactions

**DOI:** 10.1002/anie.202509964

**Published:** 2025-08-11

**Authors:** Cohan Huxley, Ethan Fung, Bara Singh, Guillermo Caballero‐García, Garrett Muir, Steven M. Silverman, Louis‐Charles Campeau, Robert Britton

**Affiliations:** ^1^ Department of Chemistry Simon Fraser University Burnaby BC V5A 1S6 Canada; ^2^ Department of Process Research and Development Merck & Co., Inc. Rahway NJ 07065 USA

**Keywords:** Cascade cyclization, *De novo* synthesis, Locked nucleic acids, Nucleosides, Stereoselective synthesis

## Abstract

Bridged nucleic acids (BNAs) are nucleoside analogues (NAs) in which the 2′‐alcohol is linked to the C4′‐position on ribose. In oligonucleotide therapeutics (ONTs), BNAs can impart beneficial properties, including enhanced stability, duplex melting temperatures, and tissue half‐lives. However, their lengthy syntheses challenge medicinal chemistry efforts and larger‐scale production. Here we demonstrate that a wide range of BNAs can be produced with various locking ring sizes and substitution patterns from a common thymine‐containing aldol product through cascade cyclization processes. Critically, several clinically relevant BNAs are now made available in as little as 3–5 steps. We expect these strategies will inspire and support medicinal and process chemistry efforts in this critical area for ONTs.

## Graphical abstract

Several processes are reported that enable the rapid diversification of bridged nucleoside analogues (NAs) starting from a common and readily prepared fluorohydrin. Also, several clinically relevant bridged nucleosides are now prepared in as few as three synthetic steps, creating new opportunities for examining structure‐activity relationships within this important family of oligonucleotide building blocks. 2

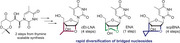

## Introduction

**Figure 1:**
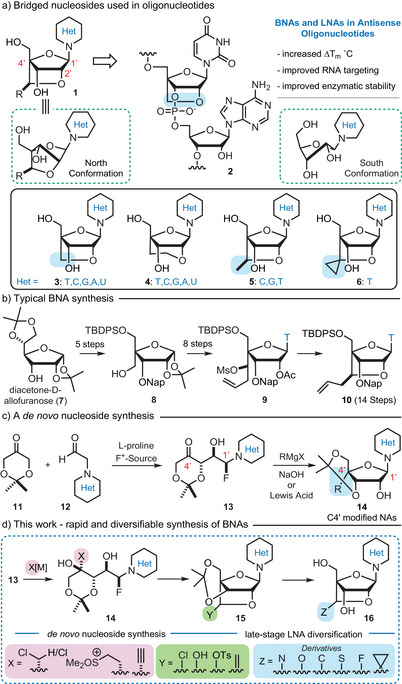
Bridged nucleosides and nucleic acids as building blocks for oligonucleotide drug discovery: structures and synthesis. Panel a: Examples of BNAs used in ONTs. Panel b: Synthesis of the BNA **10** from diacetone‐D‐allofuranose (**7**). Panel c: A platform for the *de novo* synthesis of NAs. Panel d: Rapid access to BNAs from fluorohydrins **13**. Het = heterocycle, Np = naphthyl, T = thymine, C = cytosine, G = guanine, U = uracil, A = adenine.

Nucleosides are abundant biomolecules that are essential to all life. Owing to this central role, synthetic mimics of nucleosides, known as nucleoside analogues (NAs), have played a historically important role in drug discovery. In fact, there are more than 35 NA drugs, with a significant number being antiviral or anticancer agents.^[^ ^1^ ^]^ NAs are typically modified at positions 2′ or 4′ on the furanose ring or incorporate an unnatural nucleobase and often act as toxic antimetabolites^[^ ^2^, ^3^ ^]^ or inhibitors of enzymes required for rapidly dividing cells^[^ ^4^, ^5^ ^]^ or viruses.^[^ ^1^ ^]^ Furanose modifications also impact the complex conformational equilibria, which include North (RNA‐like) or South (DNA‐like) conformations that can be vital for protein recognition and inhibition (Figure 1a).^[^ ^3^, ^5^, ^6^, ^7^ ^]^ This subtle interplay between structure and furanose ring conformation is also critical for oligonucleotide therapeutics (ONTs).^[^ ^8^ ^]^ Here, connecting the nucleoside 4′ position to the 2′‐alcohol via a two‐ or three‐atom ring (BNA: bridged nucleic acid)^[^ ^9^ ^]^ effectively locks the furanose ring in a North conformation. In ONTs, this modification often reduces the entropic penalty upon hybridization,^[^ ^8^ ^]^ improves stability toward nucleases,^[^ ^10^, ^11^ ^]^ increases duplex melting temperatures with RNA, and can improve tissue half‐lives.^[^ ^8^ ^]^ In fact, after phosphorothioate (generation I) and chemically modified riboses (for example, 2′‐fluoro, 2′‐methoxyethyl: generation II), BNAs are considered the third generation of chemically modified nucleosides used in ONTs.^[^ ^8^ ^]^

The first and most structurally simple BNA incorporates a methylene bridge and was reported independently by Imanishi (bicyclic NA) in 1997^[^ ^12^ ^]^ and Wengel (locked nucleic acid or LNA) in 1998.^[^ ^13^ ^]^ The ethylene BNA **4** reported by Koizumi in 2001 (ENA)^[^ ^14^ ^]^ further increased resistance toward exonucleases while maintaining affinity comparable to **3** to complementary RNA. Constrained ethyl LNA (cEt‐LNA (**5**))^[^ ^15^, ^16^ ^]^ and spirocyclopropylene BNA **6** (scpBNA)^[^ ^17^ ^]^ have similarly shown excellent duplex‐forming ability and enzymatic stability. Importantly, these advances have translated to clinical ONTs. For example, MRG‐110, an LNA (**3**)‐containing microRNA‐92a‐3p inhibitor, completed Phase I clinical trials in 2019 for wound healing.^[^ ^18^ ^]^ Miravirsen, which also incorporates LNA (**3**), promoted long‐term suppression of the Hepatitis C Virus without viral resistance.^[^ ^19^ ^]^ The inclusion of ENAs **4** in an antisense oligonucleotide (ASO) developed for Duchenne muscular dystrophy promoted dystrophin exon skipping in cardiac muscle and diaphragm, where conventionally modified ASOs did not.^[^ ^20^ ^]^ Also, AZD9150, a cEt‐LNA (**5**)‐containing ASO, showed single‐agent activity in a Phase 1 study involving patients with lymphoma and non‐small cell lung cancer targeting *STAT3*.^[^ ^21^ ^]^

Despite their clear importance, certain BNA‐containing ONTs have been associated with increased hepatotoxicity.^[^ ^22^ ^]^ While traditional medicinal chemistry approaches could address these complications through structural optimization, BNA synthesis is a chemistry‐intensive endeavor.^[^ ^13^, ^15^, ^16^, ^17^, ^23^, ^24^, ^25^, ^26^, ^27^, ^28^, ^29^, ^30^, ^31^ ^]^ As a result, medicinal chemistry efforts are often limited to a single target BNA or a small collection of closely related BNAs. In fact, methods used to prepare BNAs have not changed significantly since the first reported syntheses by Imanishi and Wengel (Figure 1, Panel b).^[^ ^12^, ^13^ ^]^ The major issues complicating BNA synthesis are the introduction of i) an electrophilic carbon at C4′ and ii) strain upon locking ring formation. These difficulties are manifest when targeting functionalized BNAs such as the allyl BNA **10** ^[^ ^28^ ^]^ or cEt‐LNA (**5**).^[^ ^16^ ^]^ For **5**, a 12‐step synthesis from diacetone glucose has been developed, the detailed optimization of which led the Process Research Group at AstraZeneca to state that “the synthetic burden of producing the (typically) four constituent cEt nucleosides for an oligonucleotide drug represents one of the largest seen in the “small molecule” arena.”^[^ ^23^ ^]^

With a goal of streamlining NA synthesis, we developed a *de novo* NA synthesis that relies on a one‐pot proline catalyzed α‐fluorination and subsequent aldol reaction of α‐heteroaryl acetaldehyde derivatives **12** (Figure 1, Panel c).^[^ ^32^, ^33^, ^34^ ^]^ The resulting ketofluorohydrins **13** have proven to be excellent building blocks for NAs, including C4′‐modified NAs **14**.^[^ ^32^, ^35^, ^36^ ^]^ Thus, we reasoned that this approach could provide complementary and expedient routes to BNAs that support medicinal chemistry efforts. Here, we describe several short BNA syntheses as well as straightforward platforms for BNA diversification. These concise processes should stimulate efforts to further explore the structure‐activity relationships within this important class of oligonucleotide building blocks.

## Results and Discussion

**Figure 2:**
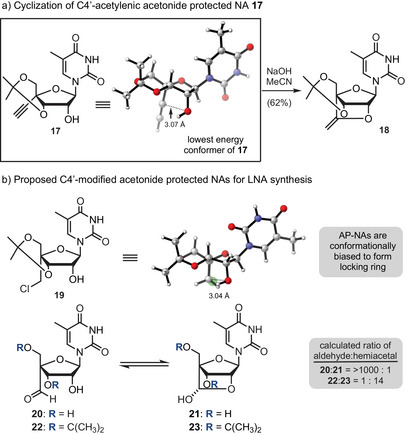
Conformational preferences of acetonide protected NAs and their exploitation to form BNAs. Panel a: Synthesis of the enol **18** from alkyne **17**. Panel b: Preferred conformations of acetonide protected NAs.

Previously we demonstrated that the C4′‐acetylenic acetonide protected NA **17** undergoes cyclization to BNA **18** (Figure 2a) by treatment with base under relatively mild conditions.^[^ ^32^ ^]^ To better understand this result, we carried out density functional theory (DFT) studies (see Supporting Information), which revealed that acetonide protected NAs adopt a North conformation that situates the C2′ hydroxy group and C4′ substituent in close contact (∼3 Å). In contrast, when the acetonide is removed from **17**, a South conformation is favored (see Figure S2) with the C2′‐OH and C4′ functional groups angled away from one another (∼4.7 Å). We also examined several C4′‐modified NAs that could support the synthesis of BNAs and found that both C4′‐CH_2_Cl and C4′‐CHO adopt similar conformations to **17** (Figure 2b). Further, for the acetonide‐protected aldehyde **22**, DFT calculations predict a ∼1.6 kcal mol^−1^ preference for the hemiacetal **23**, while the corresponding unprotected aldehyde **20** prefers the open form by ∼4.2 kcal mol^−1^. Thus, both the C4′‐chloromethyl (**19**) and C4′‐formyl (**22**) NAs should serve as useful building blocks for preparing BNAs.

**Figure 3:**
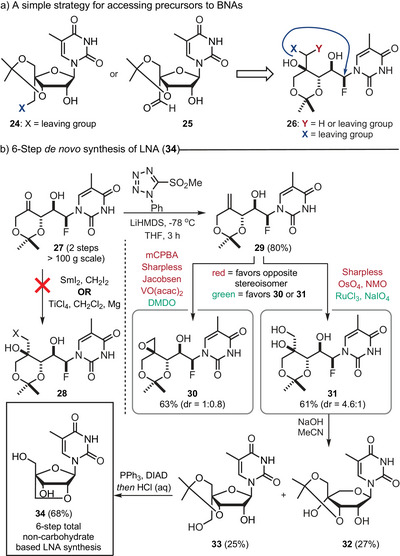
Strategy for accessing BNAs and the *de novo* synthesis of LNA (**34**). Panel a: Targeted precursors to BNAs. Panel b: A non‐carbohydrate‐based synthesis of LNA **34** from fluorohydrin **27**.

Armed with this insight, we set out to prepare the acetonide‐protected NAs **24** and **25** functionalized with electrophilic groups at the C4′‐position (Figure 3a). We first examined halomethylation of fluorohydrin **27** promoted by SmI_2_^[^ ^37^ ^]^ or TiCl_4_.^[^ ^38^ ^]^ Unfortunately, we were unable to generate the desired halohydrin **28** and only observed materials derived from decomposition. As an alternative route to **34**, we explored epoxidation or dihydroxylation of the alkene **29**, which was readily available through a Julia‐Kocienski olefination of **27**.^[^ ^39^ ^]^ Here, standard epoxidation conditions gave predominantly the (*S*)‐configured epoxide (not shown), which possesses the incorrect stereochemistry for BNA synthesis. We eventually found the (*R*)‐configured epoxide **30** could be favored using DMDO, though marginally (d.r. = 1:0.8). Likewise, common dihydroxylation strategies gave predominantly the undesired (*S*)‐configured diol (not shown). However, we were pleased to find that RuCl_3_‐catalyzed dihydroxylation, using the conditions reported by Shing,^[^ ^40^ ^]^ favored the (*R*)‐configured triol **31** (d.r. = 4.6:1). Unfortunately, under a variety of conditions, cyclization gave a ∼1:1 mixture of the pyranose **32** and the desired C4′‐hydroxymethyl NA **33** (25% yield). While pyranose formation was unavoidable, Mitsunobu cycloetherification^[^ ^41^ ^]^ carried out on **33** followed by in situ deprotection gave the LNA **34** in 68% yield. To the best of our knowledge, this six‐step synthesis of **34** is the first non‐carbohydrate based LNA synthesis.

**Figure 4:**
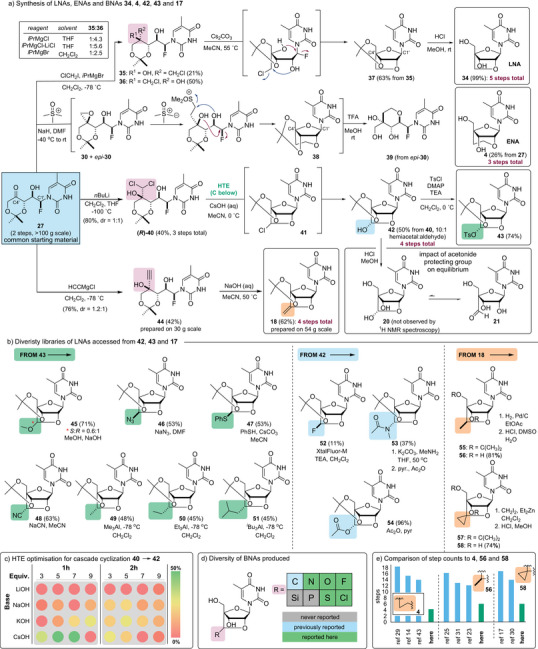
Cascade cyclization processes for rapid construction of LNAs. Panel a: *De novo* syntheses of LNA **34**, **4**, **42**, **43**, and **17**. Panel b: Rapid access to diverse LNAs from the bridge‐functionalized LNAs **42**, **43**, and **17**. Panel c: HTE for the optimization of the cascade cyclization process. Panel d: summary of new scaffolds produced in this study. Panel e: comparison of the present syntheses of ENA (**4**), cEt LNA (**56**), and scpBNA (**58**) to reported synthetic routes from carbohydrate starting materials.

Considering the competing formation of pyranose **32** from triol **31**, we reexamined the synthesis of halohydrins **28** and focused instead on the 1,2‐addition of ambiphilic reagents to ketone **27**. As depicted in Figure 4 (Panel a), the reaction of **27** with ClCH_2_MgCl, generated by metal‐halogen exchange from chloroiodomethane,^[^ ^42^ ^]^ gave a mixture of the densely functionalized chlorofluorohydrins **35** and **36** (d.r. = 1:2.5). Efforts to improve the diastereoselectivity by changing the solvent, equivalents of reagents, or temperature only led to an increase in yield of the undesired 1,3‐*anti* diol **36** or decreased conversion to both halohydrin products. Despite the unfavorable diastereoselectivity, the minor chlorofluorohydrin **35** could be produced in sufficient quantity (21% isolated yield on a gram scale) to evaluate a cascade cyclization process that would lead directly to the LNA **37** through consecutive formation of the ribose (red arrows) and locking (blue arrows) rings. In the event, we found the cascade could be carried out by heating a solution of **35** in MeCN with Cs_2_CO_3_. Notably, when the reaction was executed in MeCN‐*d*_3_ and monitored by ^1^H NMR spectroscopy, we observed a ribose intermediate, suggesting that fluoride displacement to form the ribose is followed by chloride displacement and LNA formation. Finally, removal of the acetonide protecting group under acidic conditions gave LNA **34**, which was prepared in a total of five steps (Figure 4a), a comparable accomplishment to the synthesis presented in Figure 3b.

Having established the feasibility of LNA synthesis via cascade cyclization processes, we explored the reaction of trimethylsulfoxonium anions to ketone **27**. While we expected that this may provide a more direct route to the LNA **34** via an intermediate like **24** (Figure 3a, X = ^⊕^SOMe_2_), we found that epoxide **30** formation predominated. Surprisingly, the formation of **30** was quickly followed by epoxide opening by a second equivalent of the sulfoxonium ylide and then by a cyclization cascade that gave the ENA **38** in reasonable yield over these several steps. In addition to **38**, we isolated the pyranose **39**. This latter material is produced through the opening of the diastereomeric epoxide (*epi*‐**30**) by the trimethylsulfoxonium anion followed by the same steps involved in forming the locking ring in **38** (blue arrows). As it proved easier to isolate the diol **4**, the reaction mixture was treated with MeOH/TFA to remove the acetonide, thus giving the known ENA (**4**) in a total of only three steps (26%). This synthesis compares well to the recent kg‐scale process reported by Daiichi Sankyo that required 14 steps.^[^ ^43^ ^]^

Building on the success of these cascade cyclization processes, we investigated the addition of several dihalomethyllithium reagents to fluorohydrin **28** and found that only LiCHCl_2_, derived from the reaction of CH_2_Cl_2_ and *n*‐butyl lithium, gave a low yield (<5%) of the desired product **40**. A screen of reaction conditions revealed that internal temperature monitoring was critical to the success of this process. When internal reaction temperatures rose above −95 °C, we observed rapid exothermic decomposition [caution!] of the ClCH_2_Li reagent. With this additional precaution, the 1,2‐addition reaction could be executed on a gram scale, resulting in an overall yield of 80% of a 1:1 mixture of (*S*)‐ and (*R*)‐**40**, which were readily separable by flash column chromatography. It is notable that these highly complex and densely functionalized dichlorofluorohdyrins are available from thymine in only three steps. We next evaluated the feasibility of a cascade cyclization process involving (*R*)‐**40** and found that when treated under our standard cyclization conditions (2.0 M NaOH, 40 °C),^[^ ^32^ ^]^ the dichloride (*R*)‐**40** was converted directly into the hemiacetal **42** in 20% yield. Through careful control of reaction time and equivalents of base, we could minimize hydrolysis of the chloride in **41**. However, complete avoidance of hydrolysis was not possible, so we optimized the reaction for conversion to the hemiacetal **42**, which was produced as a single diastereomer. Here, we took advantage of high‐throughput experimentation. As highlighted in part in Figure 2c, we screened several bases along with stoichiometry and reaction time. These studies identified cesium hydroxide (6 equiv.) as the optimal base. It was also found that extended reaction times led to hydrolysis, releasing thymine and other degradation products. Ultimately, we identified the conditions depicted in Figure 4a, where the cascade cyclization was executed at 0 °C with a reaction time of 45 min, affording the hemiacetal **42** in 50% yield on ∼2 mmol scale. Interestingly, in the ^1^H NMR spectrum of **42** (MeCN‐*d*_3_), the ratio between the hemiacetal **42** and aldehyde isomer was 10:1. These observations align with the calculated ratio of 14:1 (Figure 2b). Upon deprotection, only the C4′‐formyl NA **21** was observed by ^1^H NMR spectroscopy (MeCN‐*d*_3_), highlighting the profound impact the acetonide protecting group has on nucleoside conformation and locking ring formation.

With a rapid (four‐step total) synthesis of the hemiacetal **42** in hand, we were keen to explore its use for BNA diversification and medicinal chemistry. As highlighted in Figure 4a,b, several derivatives could be rapidly prepared from either hemiacetal **42** or the readily available tosylate **43**. For example, the reaction of the tosylate **43** with oxygen, nitrogen, and sulfur nucleophiles gave the unusually substituted BNAs **45–47**, predominantly with inversion of stereochemistry. From the hemiacetal **42**, reaction with Xtal‐FluorM led to the fluoro BNA **52**, while acylation gave the acetate **54**. Treatment of **42** with MeNH_2_ followed by acylation gave the *N*‐acyl hemiaminal **53** as a single stereoisomer. This unusual collection of BNAs **45**–**47** and **52**–**54** could serve as donors in pseudo glycosylation chemistry but were largely unstable to the acidic conditions required to remove the acetonide protecting group. Notably, the azide **46** could also serve as a building block for further modified BNAs through the use of azide click chemistry.^[^ ^44^ ^]^ Reaction of the tosylate **43** with cyanide or trialkyl aluminum^[^ ^45^ ^]^ reagents gave the nitrile (**48**), *c*Et (**49**), *c*Pr (**50**), and *c^i^* Pent (**51**) BNAs with retention of stereochemistry. Each of these compounds could be deprotected to provide the corresponding modified nucleoside by treatment with TFA (see Supporting Information). Collectively, these straightforward processes provide access to BNAs containing carbon, nitrogen, oxygen, sulfur, and halide substituents on the locking ring in six or less synthetic steps (Figure 4d), highlighting the unique opportunity presented here for late‐stage diversification.

Finally, based on these findings, we re‐examined the addition of alkynyl magnesium chloride to ketone **27**, which gave the tertiary alcohol **44**.^[^ ^32^ ^]^ This reaction could be carried out on a 30 g (95 mmol) scale without complication. Cyclization of the C4′‐alkyne could be carried out on scales as large as 54 g (158 mmol) to afford the exo‐alkenyl BNA **18**. With large amounts of **18** in hand, we explored subsequent diversification reactions. For example, a Simmons–Smith cyclopropanation^[^ ^30^ ^]^ gave the cyclopropyl BNA **57**, which could be deprotected to afford scpBNA (**58**). The spectral data derived from **58** was identical to that reported in the literature.^[^ ^17^ ^]^ Notably, this synthesis of scpBNA required only six steps from thymine, a significant reduction from those previously reported (Figure 4e). Similarly, reduction of the alkene function in **18** proved to be high yielding and highly diastereoselective, affording the cEt‐LNA **57**, which was deprotected to afford the known cEt‐LNA (**58**).^[^ ^15^ ^]^ Again, this synthesis (six steps) compares well to the most efficient synthesis of this compound (12 steps) reported by the AstraZeneca process research group.^[^ ^23^ ^]^

## Conclusion

Here, we have identified a unique feature of acetonide protectedNAs that positions C4′‐functional groups close to the C2′‐alcohol and supports facile BNA synthesis. Exploiting this finding, we developed rapid syntheses of C4′‐hydroxymethyl, C4′‐chloromethyl, C4′‐dichloromethyl and C4′‐alkynyl NAs. These functionalized NAs enable short, non‐carbohydrate‐based syntheses of BNAs. Moreover, we demonstrate that functionalized BNAs can be rapidly diversified to make known high value BNAs as well as new analogues. We expect that these convenient processes will inspire medicinal chemistry efforts within this important class of oligonucleotide building blocks.

## Conflict of Interests

The authors declare no conflict of interest.

## Supporting information



Supporting Information

## Data Availability

The data that support the findings of this study are available in the Supporting Information of this article.
