# Supplementary material for: Rapid Synthesis and Diversification of Thymine‐Containing Bridged Nucleic Acids Through Cascade Cyclization Reactions
Source: Angew Chem Int Ed Engl. 2025 Aug 11;64(39):e202509964. doi: 10.1002/anie.202509964 (PMC12455439; doi:10.1002/anie.202509964)
Supplement: Supplementary file 1 — Supporting Information [file ANIE-64-e202509964-s001.pdf]

# Supporting Information - Experimental

# Rapid Synthesis and Diversification of Thymine-Containing Bridged Nucleic Acids through Cascade Cyclization Reactions

Cohan Huxley,[a,c] Ethan Fung,[a,c] Bara Singh,[a] Guillermo Caballero-García,[a] Garrett Muir,[a] Steven M. Silverman,[b] Louis-Charles Campeau,[b] and Robert Britton*[a]

[a] Department of Chemistry, Simon Fraser University, Burnaby, BC V5A 1S6, Canada

Email: rbritton@sfu.ca

[b] Department of Process Research & Development, Merck & Co., Inc., Rahway, NJ, USA 07065

[c] Contributed equally

**Table of Contents**

1.0 General Experimental ........................................ 3
2.0 Experimental Procedures and Characterization Data ........................................ 4
3.0 NMR Spectra of Characterized Compounds ........................................ 18
4.0 Removal of acetonide protecting group on analytical samples of BNAs 48-50 ........................................ 48
5.0 Multi-Gram Preparation of Compound 18 ........................................ 52
6.0 Computational Details ........................................ 54
7.0 References ........................................ 90

## 1.0 General Experimental

All reactions were performed at ambient temperature and atmosphere unless otherwise specified. Flash chromatography was performed using 230-400 mesh silica gel (Merck, Silica Gel 60). Concentration of solutions and removal of solvents was done via a Büchi rotary evaporator using an acetone/dry ice condenser and vacuum applied from an Emerson vacuum pump.

All reagents, solvents, and starting materials were purchased from Sigma Aldrich, Fisher, Alfa Aesar, Oakwood Chemicals, TCI America or AK Scientific and were used without further purification or were prepared according to literature preparations where applicable. Dichloromethane ($CH_2Cl_2$) was freshly distilled over calcium hydride each time before use. Tetrahydrofuran (THF) was freshly distilled over sodium metal/benzophenone each time before use. Deuterodichloromethane ($CD_2Cl_2$) was freshly distilled over calcium hydride each time before use. -78 °C bath temperatures were maintained using an acetone/dry ice bath.

Nuclear magnetic resonance (NMR) spectra were recorded using deuterochloroform ($CDCl_3$), deuteroacetonitrile ($CD_3CN$), deuteromethanol ($CD_3OD$), or deuterodimethylsulfoxide ($(CD_3)_2SO$) as the solvent. Signal positions (δ) are given in parts per million from tetramethylsilane (δ 0) and were measured relative to the signal of the solvent ($^1$HNMR: $CDCl_3$: δ 7.26, $CD_3CN$: δ 1.94, $CD_3OD$: δ 3.31, $(CD_3)_2SO$: δ 2.50; $^{13}$C NMR: $CDCl_3$: δ 77.16, $CD_3CN$: δ 118.26, $CD_3OD$: δ 49.00, $(CD_3)_2SO$: δ 39.52). Coupling constants (*J*) are given in Hertz (Hz) and are reported to the nearest 0.1Hz. 1H NMR spectral data are tabulated in the order: multiplicity (s, singlet; d, doublet; t, triplet; q, quartet; hept, heptet; m, multiplet), coupling constants, number of protons. NMR spectra were recorded on a Bruker Advance II or III 600 equipped with a QNP or QCI cryoprobe (600 MHz), Bruker Advance III 400 (400 MHz), Bruker Advance III 500 (500 MHz) or Bruker Advance III 400_Solids (400 MHz). Diastereomeric ratios (d.r.) was determined by analysis of crude $^1$HNMR. Stereochemical assignments of compounds and signal assignments of $^1$HNMR and $^{13}$CNMR was based on analysis of COSY, HSQC, HMBC, and 2D NOESY spectra, and comparison to literature spectra, where applicable.

Optical rotation was measured on a Perkin Elmer 341 Polarimeter at 589 nm. Infrared (IR) spectra were recorded on a Thermo Nicolet Nexus 670 FTIR with neat samples. Only selected, characteristic absorption data are provided for each compound. High resolution mass spectrometry was performed on an Agilent 6210 TOF LC/MS using ESI-MS or a Bruker Maxis Impact using ESI-MS. High performance liquid chromatography (HPLC) analysis was performed on an Agilent 1290 Affinity II HPLC or an Agilent 1100 Series HPLC, equipped with a variable wavelength UV-Vis and mass spectrometry detector.

## 2.0 Experimental Procedures and Characterization Data

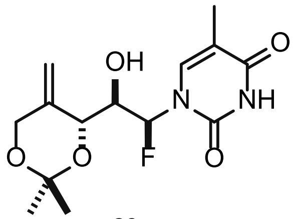

**29**

***Preparation of thymine olefin***

A solution of 5-(methylsulfonyl)-1-phenyl-1*H*-tetrazole (4.25 g, 19.0 mmol) and compound **27** (750 mg, 2.37 mmol) in THF (23.7 mL) was cooled to -78 °C. After stirring for 10 minutes LiHMDS (1M in THF, 9.49 mL, 9.49 mmol) was added dropwise. The resulting mixture was stirred for 2 hours at -78 °C. After completion the reaction was quenched with saturated $NH_4Cl$ and then diluted with EtOAc. The aqueous was extracted with EtOAc (x3) and the separated organic layer was dried with $MgSO_4$, filtered, and concentrated under reduced pressure. The crude was purified through flash column chromatography (EtOAc:Hex 60:40) to afford compound **29** as a white powder (597.0 mg, 80%)

**Compound 29: IR** (neat): υ = 3419, 2990, 2896, 1694, 1474, 1277, 1073, 916, 783 $cm^{-1}$; $[\alpha]_D^{20}$= -77 (c 0.17 in MeCN), **$^1$H NMR** (600 MHz, $CD_3CN$) δ 9.08 (s, 1H), 7.50 (m, 1H), 6.56 (dd, *J* = 43.7, 3.1 Hz, 1H), 5.11 (q, *J* = 1.4 Hz, 1H), 5.01 (m, 1H), 4.41 (m, 1H), 4.25 (m, 2H), 4.04 (ddd, *J* = 21.0, 7.8, 3.2 Hz, 1H), 3.83 (s, 1H), 1.84 (d, *J* = 1.3 Hz, 3H), 1.42 (s, 3H), 1.30 (s, 3H).δ; **$^{13}$C NMR** (151 MHz, $CD_3CN$) δ 164.3, 150.9, 143.1, 137.5, 111.1, 110.2, 100.5, 93.7, 92.3, 72.4, 64.8, 27.8, 22.6, 12.4; **$^{19}$F NMR** (565 MHz, $CD_3CN$) δ -174.74. **HRMS** ($ESI^+$) calcd for $[C_{14}H_{19}FN_2O_5+H]^+$ 315.1351 m/z found: 315.1350 m/z.

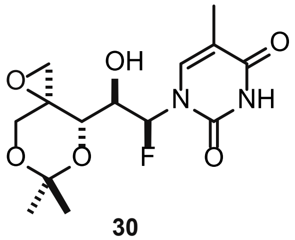

**30**

***Preparation of epoxide***

To a solution of DMDO (16 mL, 40 mM, 0.64 mmol) in acetone, compound **29** (103 mg, 0.33 mmol) was added and the mixture was stirred at 0 °C for 4 hours, until completed by TLC analysis. The reaction was quenched with the addition of 2-methyl-2-butene (180 uL, 1.54 mmol) and allowed to stir for an additional hour at 0 °C. The mixture was diluted with a saturated sodium thiosulfate solution and extracted with DCM (x3). Then, organic layers were combined and dried over sodium sulfate. 35% yield and 4.0:1 d.r. as determined by 1H NMR spectroscopic analysis using an internal standard. The resulting crude mixture was purified preparative HPLC (2 % MeCN/Water to 98 % MeCN/Water), to afford compound **30** as a white powder (34 mg, 31%).

**Compound 30: IR** (neat): υ = 3381, 2924, 2860, 1691, 1469, 1379, 1259, 1076, 854, 637 $cm^{-1}$; $[\alpha]_D^{20}$= -9.0 (c 0.18 in MeCN), **$^1$H NMR** (600 MHz, $CD_3CN$) δ 7.42 (p, *J* = 1.2 Hz, 1H), 6.46 (dd, *J* = 43.2, 2.3 Hz, 1H), 4.40 (d, *J* = 8.5 Hz, 1H), 4.26 (d, *J* = 12.9 Hz, 1H), 3.78 (m, 2H), 3.35 (d, *J* = 13.0 Hz, 1H), 3.17 (d, *J* = 4.6 Hz, 1H), 2.57 (d, *J* = 4.6 Hz, 1H), 1.83 (d, *J* = 1.3 Hz, 3H), 1.50 (s, 3H), 1.34 (s, 3H); **$^{13}$C NMR** (151 MHz, $CD_3CN$) δ 163.4, 149.9, 136.4, 110.0, 99.4, 92.3 (d, *J* = 205.8 Hz), 68.9, 68.2, 65.2, 55.0, 49.0, 27.3, 18.9, 11.5; **$^{19}$F NMR** (565 MHz, $CD_3CN$) δ -177.10; **HRMS** ($ESI^+$) calcd for $[C_{14}H_{19}FN_2O_6+H]^+$ 331.1300 m/z found: 331.1297 m/z.

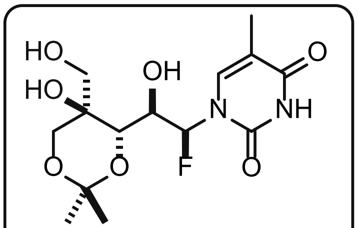
31

### *Preparation of thymine triol*

A slurry of sodium metaperiodate (44 mg, 0.21 mmol) and cerium (III) chloride heptahydrate (5.9 mg, 16 μmol) in water (1.5 mL) was heated with a heat gun until the slurry turned a bright yellow colour. Afterwards acetonitrile (3.8 mL) was added, and the solution was cooled to 0 °C. Then, ruthenium (III) chloride trihydrate (4.2 mg, 16 μmol) was added and the mixture was stirred for 5 minutes at 0 °C. Finally, compound **29** (50.0 mg, 0.16 mmol) was added and the reaction was stirred at 0 °C for 2 hours. Afterwards the reaction was quenched with saturated sodium thiosulfate and extracted with 25% MeOH in DCM (x7). The organic layers were dried over $MgSO_4$ and concentrated under reduced pressure. Purification proceeded through preparative HPLC (45 % MeCN/Water to 75 % MeCN/Water) to afford compound **31** as a white powder (29.8 mg, 54%).

**Compound 31: IR** (neat): υ = 3425, 3217, 2989, 1694, 1464, 1377, 1280, 1224, 1084, 887, 778, 591 cm$^{-1}$; $[\alpha]_D^{20}$= -10.3 (c 1.00 in MeCN), **$^1$H NMR** (600 MHz, $CD_3CN$) δ 9.05 (s, 1H), 7.47 (s, 1H), 6.38 (dd, *J* = 43.7, 3.5 Hz, 1H), 4.46 (d, *J* = 3.4 Hz, 1H), 4.06 (dddd, *J* = 20.5, 9.6, 4.4, 3.4 Hz, 1H), 3.91 (d, *J* = 9.6 Hz, 1H), 3.83 (dd, *J* = 11.5, 5.0 Hz, 1H), 3.68 (m, 3H), 3.54 (m, 1H), 3.13 (d, *J* = 5.6 Hz, 1H), 1.83 (d, *J* = 1.3 Hz, 3H), 1.41 (s, 3H), 1.26 (s, 3H); **$^{13}$C NMR** (151 MHz, $CD_3CN$) δ 164.3, 151.0, 137.6, 111.0, 100.3, δ 93.2 (d, *J* = 206.9 Hz), 73.1, 72.0 (d, *J* = 23.7 Hz)., 70.6, 67.6, 66.0, 62.4, 28.5, 19.3, 12.4.; **$^{19}$F NMR** (565 MHz, $CD_3CN$) δ -174.67; **HRMS** (ESI$^+$) calcd for $[C_{14}H_{21}FN_2O_7+H]^+$ 349.1406 m/z found: 349.1420 m/z.

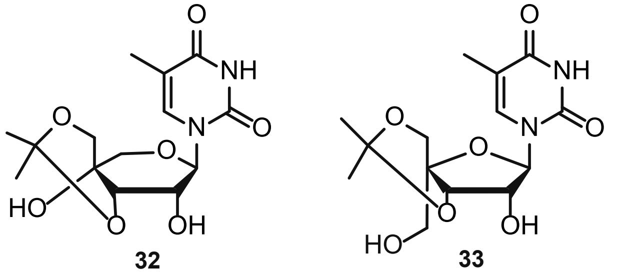
32 33

### *Cyclization of the thymine triol*

In a flask, compound **31** (7.3 mg, 21 μmol) was dissolved in acetonitrile (0.42 mL). Then cesium carbonate was added, and the mixture was stirred at 60 °C until completion. Once complete the mixture was filtered and the solvent was removed under reduce pressure. Purification proceeded through preparative HPLC (2 % MeCN/Water to 30 % MeCN/Water) to afford compound **32** as a white powder (1.7 mg, 25%) and compound **33** as a white powder (1.9 mg, 27%).

**Compound 32: IR** (neat): υ = 3389, 2998, 2940, 1681, 1458, 1264, 1192, 1026, 912, 589 cm$^{-1}$; $[\alpha]_D^{20}$= -19.0 (c 0.10 in MeCN), **$^1$H NMR** (600 MHz, $CD_3CN$) δ 7.37 (d, *J* = 1.3 Hz, 1H), 5.67 (d, *J* = 9.6 Hz, 1H), 4.26 (d, *J* = 12.3 Hz, 1H), 4.14 (dd, *J* = 3.5, 1.5 Hz, 1H), 3.99 (s, 1H), 3.69 (d, *J* = 11.8 Hz, 1H), 3.56 (dd, *J* = 12.3, 1.6 Hz, 2H), 3.41 (d, *J* = 11.8 Hz, 1H), 3.25 (d, *J* = 8.8 Hz, 1H), 1.85 (d, *J* = 1.3 Hz, 3H), 1.48 (s, 3H), 1.43 (s, 3H); **$^{13}$C NMR** (151 MHz, $CD_3CN$) δ 164.2, 151.9, 136.7, 111.5, 100.2, 81.1, 74.0, 70.4, 66.4, 65.8, 65.3, 29.3, 18.7, 12.4.; **HRMS** (ESI$^+$) calcd for $[C_{14}H_{20}N_2O_7+H]^+$ 329.1343 m/z found: 329.1343 m/z

**Compound 33: IR** (neat): υ = 3387, 2992, 2928, 1683, 1469, 1264, 1139, 1039, 850, 590 cm$^{-1}$; $[\alpha]_D^{20}$= -17.5 (c 0.50 in MeCN), $^{1}$**H NMR** (500 MHz, $CD_3CN$) δ 7.20 (d, *J* = 1.4 Hz, 1H), 5.92 (d, *J* = 1.2 Hz, 1H), 4.23 (m, 1H), 4.08 (m, 2H), 3.89 (dd, *J* = 11.2, 8.0 Hz, 2H), 3.77 (d, *J* = 12.4 Hz, 1H), 1.86 (d, *J* = 1.3 Hz, 3H), 1.54 (s, 3H), 1.45 (s, 3H); $^{13}$**C NMR** (126 MHz, $CD_3CN$) δ 164.6, 151.3, 137.2, 111.5, 102.9, 93.7, 79.5, 75.8, 73.8, 69.1, 61.0, 29.3, 20.0, 12.3; **HRMS** ($ESI^+$) calcd for $[C_{14}H_{20}N_2O_7+H]^+$ 329.1343 m/z found: 329.1342 m/z.

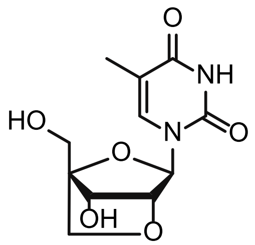


**34**

***Deprotection of locked nucleoside***

A solution of **37** (17.8 mg, 57.3 µmol) in MeOD (0.5 mL, 0.11 M) and DCl (12µL, 35 wt.% in $D_2O$, 115 µmol) was shaken at room temperature. NMR analysis showed complete deprotection after 15 mins. The volatiles were removed under reduced pressure, to afford a white powder, compound **34** (15.5 mg, Quant.).

**Compound 34: IR** (neat): υ = 3401, 2921, 2849, 1693, 1473, 1390, 1269, 1117, 1055, 779cm$^{-1}$; $[\alpha]_D^{20}$= -22.0 (c 0.12 in MeCN), $^{1}$**H NMR** (600 MHz, MeOD) δ 7.75 (d, *J* = 1.3 Hz, 1H), 5.54 (s, 1H), 4.27 (s, 1H), 4.07 (s, 1H), 3.96 (d, *J* = 7.8 Hz, 1H), 3.91 (d, *J* = 1.1 Hz, 2H), 3.75 (d, *J* = 7.9 Hz, 1H), 1.89 (d, *J* = 1.2 Hz, 3H); $^{13}$**C NMR** (151 MHz, MeOD) δ 166.5, 151.9, 136.8, 110.7, 90.4, 88.3, 80.9, 72.4, 70.4, 57.6, 12.6; **HRMS** ($ESI^+$) calcd for $[C_{11}H_{14}N_2O_6+H]^+$ 271.0925 m/z found: 271.0918 m/z.

Matches literature data from: Jakhlal, J.; Denhez, C.; Coantic-Castex, S.; Martinez, A.; Harakat, D.; Douki, T.; Guillaume, D.; Clivio, P. SN- and NS-Puckered Sugar Conformers Are Precursors of the (6-4) Photoproduct in Thymine Dinucleotide. *Org Biomol Chem* **2022**, *20* (11), 2300–2307. https://doi.org/10.1039/d2ob00044j.

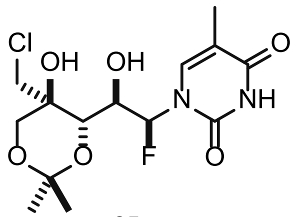


**35**

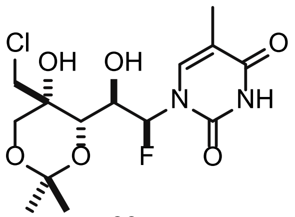


**36**

***1,2-Addition of dichloromethane to α-fluorohydrin***

A solution of isopropylmagnesium bromide (2.52 mL, 0.88 M, 2.21 mmol) and chloroiodomethane (207 µL, 2.85 mmol) was cooled to -78 °C under nitrogen and stirred for 1 hour. Compound **27** (100.0 mg, 61.3 mmol) in DCM (6.3 mL, 0.05 M) was slowly added over 15 mins. The reaction was allowed to stir for an additional 3.5 hours. 5 mL of a 1:1 solution of saturated ammonium chloride:methanol was slowly added and the reaction slowly brought to room temperature. The crude mixture was extracted 5 of DCM and the organic layers were combined and dried with sodium sulfate. The final product was purified by silica flash chromatography (20 % MeCN/DCM) to afford a white foam, compound **35** (23.8 mg, 21 %) and a white foam, compound **36** (40.0 mg, 34 %), 0.6:1 in crude mixture.

**Compound 35: IR** (neat): υ = 2958, 2928, 2859, 1720, 1462, 1379, 1267, 1117, 1102, 1019, 863, 730 cm$^{-1}$; $[\alpha]_D^{20}$= -47 (c 0.2 in $CH_2Cl_2$), **$^1$H NMR** (600 MHz, $D_3CCN$): δ 9.06 (s, 1H), 7.36 (s, 1H), 6.34 (dd, *J* = 43.8, 3.6 Hz, 1H), 4.46 (d, *J* = 4.9 Hz, 1H), 4.08 (d, *J* = 11.6 Hz, 1H), 4.07 – 4.03 (m, 1H), 4.03 – 4.00 (m, 1H), 3.91 (d, *J* = 11.8 Hz, 1H), 3.82 (s, 1H), 3.81 (dd, J = 11.6, 1.4 Hz, 1H), 3.58 (dd, *J* = 11.8, 1.4 Hz, 1H), 1.84 (d, J = 1.3 Hz, 3H), 1.41 (s, 3H), 1.27 (s, 3H); **$^{13}$C NMR** (150 MHz, $D_3CCN$): δ 164.22, 151.01, 137.41 (d, $J_{C\text{-}F}$ = 3.4 Hz), 111.26, 100.66, 93.96 (d, $J_{C\text{-}F}$ = 206.9 Hz), 73.56 (d, $J_{C\text{-}F}$ = 4.1 Hz), 71.54 (d, $J_{C\text{-}F}$ = 24.8 Hz), 69.62, 65.36, 47.72, 28.77, 18.96, 12.39; **$^{19}$F NMR** (565 MHz, $D_3CCN$): δ -173.3; HRMS (ESI$^+$) calcd for $[C_{14}H_{20}ClFN_2O_6+H]^+$ 367.1067 m/z found: 367.1075 m/z.

**Compound 36: IR** (neat): υ = 3386, 2959, 2929, 1694, 1464, 1378, 1266, 1118, 1102, 867, 731, 703, 548 cm$^{-1}$; $[\alpha]_D^{20}$= -39.9 (c 0.54 in $CH_2Cl_2$), **$^1$H NMR** (600 MHz, $D_3CCN$): δ 9.16 (br, 1H), 7.43 (d, *J* = 1.6 Hz, 1H), 6.46 (dd, *J* = 42.9, 2.8 Hz, 1H), 4.08 (dddd, *J* = 23.9, 8.9, 7.3, 2.8 Hz, 1H), 3.96 (d, *J* = 12.2 Hz, 1H), 3.90 (d, *J* = 8.8 Hz, 1H), 3.84 (d, *J* = 11.8 Hz, 1H), 3.83 – 3.80 (m, 1H), 3.72 (dd, *J* = 11.9, 1.3 Hz, 1H), 3.68 (d, *J* = 12.3 Hz, 1H), 3.66 (s, 1H), 1.84 (d, *J* = 1.3 Hz, 3H), 1.39 (s, 3H), 1.34 (s, 3H); **$^{13}$C NMR** (150 MHz, $D_3CCN$): δ 164.49, 150.86, 137.42, 110.84, 100.13, 93.66 (d, *J* = 205.6 Hz), 73.32 (d, *J* = 2.3 Hz), 70.12, 69.77 (d, *J* = 23.6 Hz), 66.99, 48.80, 28.04, 19.47, 12.46; **$^{19}$F NMR** (470 MHz, $D_3CCN$): δ -174.4; HRMS (ESI$^+$) calcd for $[C_{14}H_{20}ClFN_2O_6+H]^+$ 367.1067 m/z found: 367.1073 m/z.

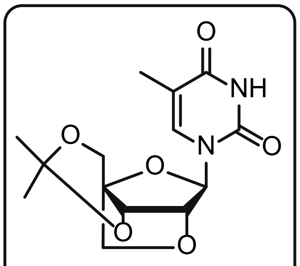

**37**

***Cyclisation to thymine locked nucleoside (Mitsunobu reaction)***

A solution of compound **33** (15.0 mg, 45.7 µmol) and triphenylphosphine (18.0 mg, 68.5 µmol) in THF (0.92 mL, 0.05 M) was stirred under nitrogen at 0 °C. DIAD (13.3 µL, 68.5 µmol) was added drop wise to the solution and the reaction was allowed to warm to room temperature overnight. After the reaction was complete determined by TLC analysis the solvent was removed under reduced pressure and the crude mixture was purified through purified by preparative HPLC (2 % MeCN/Water to 98 % MeCN/Water) to afford compound **37** as a white powder (9.6 mg, 68%)

***Cyclisation to thymine locked nucleoside (Monochloro cyclisation)***

To a solution of compound **35** (10.0 mg, 27 µmol) in acetonitrile (0.55 mL) was added cesium carbonate (13.0 mg, 41 µmol). The reaction was allowed to stir at 55 °C for 18 hours. Afterwards the reaction was filtered and the solvent was removed under reduce pressure. The final product was purified by silica flash chromatography (acetone:hexanes 0% to 50% acetone) to afford compound **37** as a white solid (5.3 mg, 63%)

**Compound 37: IR** (neat): υ = 2958, 2929, 1692, 1462, 1383, 1266, 1199, 1115, 1089, 1050, 849, 730, 580 cm$^{-1}$; $[\alpha]_D^{20}$= +21.2 (c 0.32 in $CH_2Cl_2$), **$^1$H NMR** (600 MHz, $D_3CCN$): δ 9.03 (br, 1H), 7.37 (q, *J* = 1.2 Hz, 1H), 5.52 (s, 1H), 4.47 (d, *J* = 11.1 Hz, 1H), 4.45 (s, 1H), 4.29 (d, *J* = 8.1 Hz, 1H), 4.06 (d, *J* = 11.0 Hz, 0H), 3.78 (d, *J* = 8.1 Hz, 1H), 3.76 (s, 1H), 1.87 (d, *J* = 1.3 Hz, 3H), 1.52 (s, 3H), 1.42 (s, 3H); **$^{13}$C NMR** (150 MHz, $D_3CCN$): δ 164.6, 151.0, 135.2, 110.7, 101.8, 89.7, 79.6, 79.3, 72.5, 72.1, 60.8, 29.3, 19.6, 12.5; **HRMS** (ESI$^+$) calcd for $[C_{14}H_{18}N_2O_6+H]^+$ 311.1238 m/z found: 311.1241 m/z.

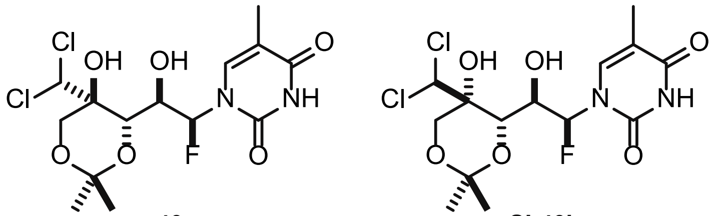

**40** **SI-40b**

***1,2-Addition of dichloromethane to α-fluorohydrin***

A solution of 1:2.8 DCM:THF (49 mL) was cooled to -100 °C (internally) under Argon. nBuLi (32 mL, 2.5 M, 79.3 mmol) was added by syringe pump over 1 hour. The internal temperature was maintained between -105 to -95 °C. The reaction was diluted by 46 mL DCM cooled to -78 °C via capillary transfer over 20 mins and stirred for an additional hour. Compound 27 (1.25 g, 3.96 mmol) in 18 mL THF was added via syringe pump over 1 hour. The reaction was stirred for an additional 5 hours and quenched with 20 mL of a 1:1 solution of saturated ammonium chloride:methanol followed by an additional 20 mL of saturated ammonium chloride then slowly brought to room temperature. The crude mixture was extracted 5 fives totalling 600 mL of DCM and the organic layers were combined and dried with sodium sulfate. The final product was purified by silica flash chromatography (20 % MeCN/DCM) to afford a white foam, compound **40** / **SI-40b** as a 1:1 mixture (1.26 g, 80 %)

**Compound 40: IR** (neat): υ = 3496, 2939, 1720, 1649, 1270, 1118, 864, 731 $cm^{-1}$; $[\alpha]_D^{20}$= -35.7 (c 0.46 in $CH_2Cl_2$), **$^1$H NMR** (600 MHz, $D_3CCN$): δ 9.14 (s, 1H, NH), 7.39 – 7.34 (m, 1H), 6.36 (dd, *J* = 43.3, 2.9 Hz, 1H), 6.34 (s, 1H), 4.42 (d, *J* = 6.0 Hz, 1H, OH), 4.32 (dddd, *J* = 21.4, 10.3, 6.0, 2.9 Hz, 1H), 4.22 (s, 1H, OH), 4.16 (d, *J* = 12.3 Hz, 1H), 4.12 (d, *J* = 10.3 Hz, 1H), 3.75 (d, *J* = 12.3 Hz, 1H), 1.84 (d, *J* = 1.3 Hz, 3H), 1.42 (s, 3H), 1.35 (s, 3H); **$^{13}$C NMR** (150 MHz, $D_3CCN$): δ 164.4, 150.9, 137.2 (d, $J_{C\text{-}F}$ = 4.9 Hz), 111.2, 101.9, 93.7 (d, $J_{C\text{-}F}$ = 208.1 Hz), 76.3, 74.8, 73.6 (d, $J_{C\text{-}F}$ = 4.0 Hz), 71.1 (d, $J_{C\text{-}F}$ = 23.6 Hz), 67.7, 27.1, 20.4, 12.4; **$^{19}$F NMR** (470 MHz, $D_3CCN$): δ -179.0; HRMS ($ESI^+$) calcd for $[C_{14}H_{19}Cl_2FN_2O_6+H]^+$ 401.0677 m/z found: 401.0682 m/z.

**Compound SI-40b: IR** (neat): υ = 3487, 2928, 2859, 1720, 1463, 1408, 1268, 1117, 1019, 873 $cm^{-1}$; $[\alpha]_D^{20}$= -13.5 (c 0.56 in $CH_2Cl_2$), **$^1$H NMR** (600 MHz, $D_3CCN$): δ 9.11 (s, 1H, NH), 7.41 – 7.38 (m, 1H), 6.45 (dd, *J* = 42.9, 2.9 Hz, 1H), 6.34 (s, 1H), 4.36 (d, *J* = 9.0 Hz, 1H), 4.33 (d, *J* = 12.6 Hz, 1H), 4.14 (s, 1H, OH), 4.12 (m, 1H), 3.97 (d, *J* = 7.2 Hz, 1H, OH), 3.82 (d, *J* = 12.6 Hz, 1H), 1.85 (d, *J* = 1.3 Hz, 3H), 1.40 (s, 3H), 1.34 (s, 3H); **$^{13}$C NMR** (150 MHz, $D_3CCN$): δ 164.1, 150.9, 137.3 (d, $J_{C\text{-}F}$ = 5.4 Hz), 111.0, 100.4, 93.9 (d, $J_{C\text{-}F}$ = 205.5 Hz), 76.7, 73.8, 71.2 (d, $J_{C\text{-}F}$ = 2.7 Hz), 69.9 (d, $J_{C\text{-}F}$ = 24.0 Hz) 64.3, 27.8, 20.2, 12.5; **$^{19}$F NMR** (470 MHz, $D_3CCN$): δ -178.0; **HRMS** ($ESI^+$) calcd for $[C_{14}H_{19}Cl_2FN_2O_6+H]^+$ 401.0.677 m/z found: 401.0687 m/z.

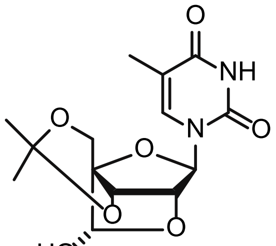

42

### *Double cyclisation, displacement to hemiacetal-locked nucleoside*

A solution of **40a** (200 mg, 0.50 mmol) and MeCN (10 mL, 0.05M) was stirred to 0 °C. CsOH 50 wt. % in $H_2O$ (0.90 mL, 3.0 mmol) was added, and the mixture was stirred for 45 mins followed by the addition of ammonium chloride (400.0 mg, 7.5 mmol). The mixture was diluted with water (10 mL) and frozen. Solvent was removed by freeze drying and the powdered solid was purified by silica flash chromatography (1 % IPA/70 % EtOAc/n-hexanes), to afford a white foam, compound **42** (82.5 mg, 51 %)

**Compound 42: IR** (neat): υ = 3219, 2958, 2928, 1693, 1463, 1384, 1268, 1117, 1076, 580 $cm^{-1}$; $[\alpha]_D^{20}$= +11 (c 0.84 in $CH_2Cl_2$), **$^1$H NMR** (600 MHz, $CD_3CN$): δ 9.11 (s, 1H, NH), 7.38 (d, *J* = 1.4 Hz, 1H), 5.74 (s, 1H), 5.61 (s, 1H), 4.48 (s, 1H), 4.42 (d, *J* = 11.0 Hz, 1H), 4.01 (d, *J* = 11.0 Hz, 1H), 3.93 (s, 1H), 1.88 (d, *J* = 1.3 Hz, 3H), 1.51 (s, 3H), 1.43 (s, 3H) ; **$^{13}$C NMR** (151 MHz, $CD_3CN$) δ 164.5, 150.9, 135.1, 110.9, 102.0, 99.4, 88.9, 79.9, 78.2, 74.5, 60.2, 29.0, 19.5, 12.5.; **HRMS** (ESI$^+$) calcd for $[C_{14}H_{18}N_2O_7+H]^+$ 327.1187 m/z found: 327.1195 m/z.

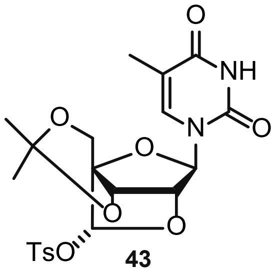

43

### *Tosylation of hemiacetal-locked nucleoside*

A solution of **42** (55.0 mg, 0.17 mmol), DMAP (2.0 mg, 17 µmol) and triethylamine (35 uL, 253 µmol) in DCM (3.4 mL, 0.05M) was stirred to 0 °C. 4-Toluenesulfonyl chloride (39.0 mg, 0.20 mmol) was added in one portion and the mixture was stirred for 4 hours. The crude mixture was loaded directly onto silica flash chromatography (1 % IPA/50 % EtOAc/n-hexanes), to afford a white powder, compound **43** (39.5 mg, 49 %). For the following substitution reactions compound **43** was not purified by silica flash chromatography but was instead diluted in MeCN and washed three times with n-hexanes. Acetonitrile was removed in under reduced pressure and the crude material (~80% pure) was used.

**Compound 43: IR** (neat): υ = 2958, 2927, 2858, 1720, 1462, 1375, 1268, 1192, 1178, 1100, 1019, 730, 583 $cm^{-1}$; $[\alpha]_D^{20}$= +47 (c 0.18 in $CH_2Cl_2$), **$^1$H NMR** (500 MHz, $D_3CCN$): δ 9.19 (s, 1H), 7.88 – 7.84 (m, 2H), 7.48 – 7.44 (m, 2H), 7.33 (q, *J* = 1.3 Hz, 1H), 6.30 (s, 1H), 5.50 (s, 1H), 4.61 (s, 1H), 4.42 (d, *J* = 11.5 Hz, 1H), 3.95 (s, 1H), 3.91 (d, *J* = 11.5 Hz, 1H), 2.46 (s, 3H), 1.86 (d, *J* = 1.3 Hz, 3H), 1.50 (s, 3H), 1.42 (s, 3H) ; **$^{13}$C NMR** (126 MHz, $D_3CCN$): δ 164.6, 150.9, 147.0, 135.2, 134.5, 131.2, 128.9, 111.1, 104.1, 102.5, 88.3, 82.2, 79.1, 74.6, 59.5, 28.9, 21.7, 19.5, 12.4; **HRMS** (ESI$^+$) calcd for $[C_{21}H_{24}N_2O_9S+H]^+$ 481.1275 m/z found: 481.1281 m/z.

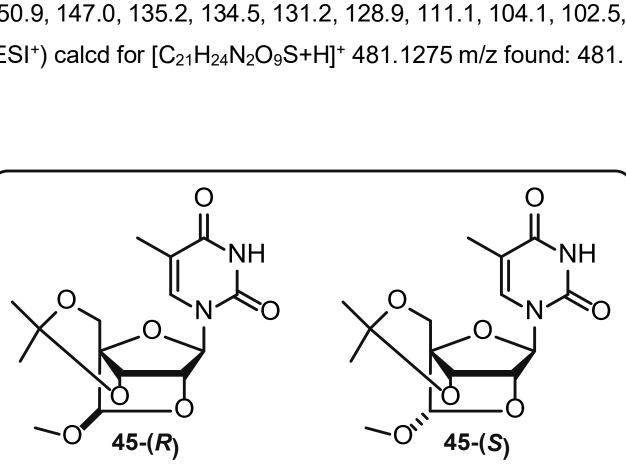

45-(*R*) 45-(*S*)

### *Preparation of (R)- and (S)-methoxy locked nucleosides*

Sodium (6.0 mg, 0.3 mmol) was added to methanol (0.4 mL) at 0 °C under nitrogen. Once the solution stopped bubbling, **43** (19.8 mg, 41 µmol) in methanol (0.4 mL) was added and stirred for 7 hours at 0 °C under nitrogen. Saturated ammonium carbonate (1 mL) was added, and the mixture was extracted 5 times

with DCM, organic layers were combined and dried over sodium sulfate. The crude mixture purified preparative silica flash chromatography (1 % IPA/40%EtOAc/n-hexanes), to afford a white powder, compounds **45-(*R*)** and **45-(*S*)** (9.9 mg, 71 %, 1:0.6). Analytical samples were separated by preparative HPLC (2 % MeCN/Water to 98 % MeCN/Water).

**Compound 45-(*R*): IR** (neat): υ = 2958, 2928, 2859, 1720, 1463, 1381, 1268, 1116, 1102, 731 $cm^{-1}$; $[\alpha]_D^{20}$= -12 (c 0.2 in $CH_2Cl_2$), **$^1$H NMR** (600 MHz, $D_3CCN$): δ 9.03 (s, 1H), 7.33 (d, *J* = 1.7 Hz, 1H), 5.32 (s, 1H), 4.81 (s, 1H), 4.65 (s, 1H), 4.45 (d, *J* = 11.3 Hz, 1H), 4.22 (d, *J* = 11.3 Hz, 1H), 3.74 (s, 1H), 3.43 (s, 3H), 1.87 (d, *J* = 1.3 Hz, 3H), 1.47 (s, 3H), 1.35 (s, 3H); **$^{13}$C NMR** (151 MHz, $D_3CCN$): δ 164.6, 151.0, 135.2, 110.9, 108.8, 101.0, 89.2, 77.5, 77.4, 71.2, 60.8, 57.3, 28.0, 20.2, 12.5; **HRMS** ($ESI^+$) calcd for $[C_{15}H_{21}N_2O_7+H]^+$ 341.1343 m/z found: 341.1350 m/z (±2.0 ppm), calcd for $[C_{15}H_{20}N_2O_7+Na]^+$ 363.1162 m/z found: 363.1162 m/z.

**Compound 45-(*S*): IR** (neat): υ = 2958, 2929, 2859, 1720, 1463, 1268, 1118, 1019, 731 $cm^{-1}$; $[\alpha]_D^{20}$= +79 (c 0.14 in $CH_2Cl_2$), **$^1$H NMR** (600 MHz, $D_3CCN$): δ 9.09 (s, 1H), 7.38 (d, *J* = 2.1 Hz, 1H), 5.53 (s, 1H), 5.49 (s, 1H), 4.50 (s, 1H), 4.41 (d, *J* = 11.1 Hz, 1H), 4.04 (d, *J* = 11.1 Hz, 1H), 3.87 (s, 1H), 3.51 (s, 3H), 1.86 (d, *J* = 1.5 Hz, 3H), 1.52 (s, 3H), 1.43 (s, 3H); **$^{13}$C NMR** (151 MHz, $D_3CCN$): δ 164.6, 151.0, 135.4, 110.6, 106.1, 101.9, 88.6, 80.4, 78.2, 74.9, 60.3, 57.6, 29.0, 19.5, 12.5; **HRMS** ($ESI^+$) calcd for $[C_{15}H_{21}N_2O_7+H]^+$ 341.1343 m/z found: 341.1350 m/z (±2.0 ppm), calcd for $[C_{15}H_{20}N_2O_7+Na]^+$ 363.1162 m/z found: 363.1162 m/z.

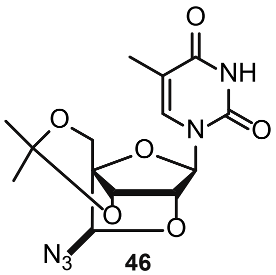

**46**

***Preparation of azide locked nucleoside***

A solution of **43** (12.7 mg, 26.4 μmol), in DMF (0.4 mL, 0.06M) was stirred to 60 °C under nitrogen. Sodium azide (8.6 mg, 132 μmol) was 20 hours. Saturated ammonium chloride (1 mL) was added, and the mixture was extracted 5 times with DCM, organic layers were combined and dried over sodium sulfate. The crude mixture purified preparative HPLC (2 % MeCN /Water to 98 % MeCN /Water), to afford a white powder, compound **46** (4.9 mg, 53%).

**Compound 46: IR** (neat): υ = 2959, 2929, 2857, 2127, 1721, 1463, 1268, 1117, 1019, 731 $cm^{-1}$; $[\alpha]_D^{20}$= -24 (c 0.08 in $CH_2Cl_2$), **$^1$H NMR** (500 MHz, $D_3CCN$): δ 9.03 (br, 1H, NH), 7.32 (d, *J* = 1.3 Hz, 1H), 5.43 (s, 1H), 4.91 (s, 1H), 4.80 (s, 1H), 4.54 (d, J = 11.6 Hz, 1H), 4.23 (d, *J* = 11.6 Hz, 1H), 3.86 (s, 1H), 1.87 (d, *J* = 1.3 Hz, 3H), 1.54 (s, 3H), 1.48(s, 3H); **$^{13}$C NMR** (126 MHz, $D_3CCN$): δ 164.5, 150.9, 134.9, 111.1, 101.8, 91.4, 89.0, 78.9, 77.8, 71.1, 60.6, 28.5, 19.7, 12.5; **HRMS** ($ESI^+$) calcd for $[C_{14}H_{17}N_5O_6+H]^+$ 352.1253 m/z found: 352.1251 m/z.

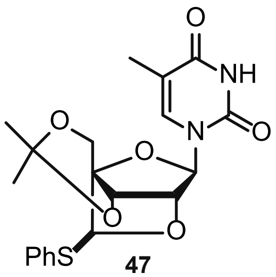

**47**

***Preparation of thiophenyl-locked nucleoside***

A solution of **43** (20.0 mg, 41.6 μmol), caesium carbonate (54.2 mg, 166 μmol) and thiophenol (12.9 μL, 125 μmol) in DMF (0.4 mL, 0.1M) was stirred to 40 °C under nitrogen for 1.5 hours. Water (2 mL) was added, and the mixture was extracted 5 times with DCM, organic layers were combined and dried over sodium sulfate. 53% yield as determined by 1H NMR spectroscopic analysis using internal standard. The crude mixture purified preparative HPLC (2 % MeCN/Water to 98 % MeCN/Water), to afford a white powder, compound **47** (4.9 mg, 28% isolated).

**Compound 47:** **IR** (neat): υ = 2959, 2936, 1721, 1465, 1268, 1116, 749, 729 $cm^{-1}$; $[\alpha]_D^{20}$= -78 (c 0.1 in $CH_2Cl_2$), **$^1$H NMR** (600 MHz, $D_3CCN$): δ 9.01 (s, 1H), 7.56 – 7.51 (m, 2H), 7.39 – 7.32 (m, 3H), 7.30 – 7.25 (m, 1H), 5.51 (s, 1H), 5.45 (s, 1H), 4.72 (s, 1H), 4.58 (d, *J* = 11.3 Hz, 1H), 4.29 (dd, *J* = 11.2, 1.0 Hz, 1H), 3.86 (s, 1H), 1.87 (d, *J* = 1.3 Hz, 3H), 1.55 (s, 3H), 1.52 (s, 3H); **$^{13}$C NMR** (150 MHz, $D_3CCN$): δ 164.4, 150.8, 135.0, 130.9, 130.1, 127.8, 111.0, 101.6, 92.5, 89.3, 80.5, 79.1, 71.9, 61.3, 28.2, 19.6, 12.5; **HRMS** ($ESI^+$) calcd for $[C_{20}H_{22}N_2O_6S+H]^+$ 419.1271 m/z found: 419.1280 m/z.

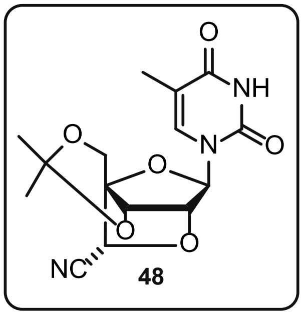


**48**

***Preparation of cyano-locked nucleoside***

To a solution of **43** (14.5 mg, 30.2 µmol), in MeCN (0.5 mL, 0.06M), 18-crown-6 (16.0 mg, 60.4 µmol) and potassium cyanide (4.0 mg, 60.4 µmol) was added and stirred for 16 hours at 25 °C under nitrogen. Saturated $NaHCO_3$ (1 mL) was added, and the mixture was extracted 5 times with DCM, organic layers were combined and dried over sodium sulfate. The crude mixture purified preparative HPLC (2 % MeCN/Water to 98 % MeCN/Water), to afford a white powder, compound **48** (6.4 mg, 63 %).

**Compound 48:** **IR** (neat): υ = 2958, 2929, 1697, 1463, 1269, 1198, 1108, 1048, 866, 731 $cm^{-1}$; $[\alpha]_D^{20}$= +21.6 (c 0.57 in $CH_2Cl_2$), **$^1$H NMR** (500 MHz, $D_3CCN$): δ 9.14 (br, 1H, NH), 7.35 (q, *J* = 1.4 Hz, 1H), 5.69 (s, 1H), 5.33 (s, 1H), 4.75 (s, 1H), 4.55 (d, *J* = 11.8 Hz, 1H), 4.17 (d, *J* = 11.8, 1H), 4.02 (s, 1H), 1.88 (d, *J* = 1.3 Hz, 3H), 1.53 (s, 3H), 1.44 (s, 3H); **$^{13}$C NMR** (126 MHz, $D_3CCN$): δ 164.5, 150.9, 135.0, 117.0, 111.3, 102.7, 89.7, 81.2, 80.9, 72.8, 71.3, 59.9, 29.0, 19.5, 12.5; **HRMS** ($ESI^+$) calcd for $[C_{15}H_{17}N_3O_6+H]^+$ 336.1190 m/z found: 336.1198 m/z.

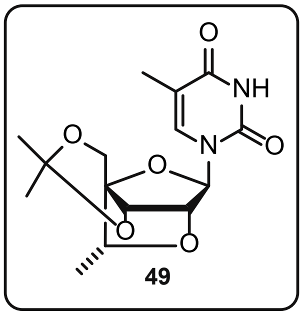


**49**

***Preparation of (R)-methyl locked nucleoside***

A solution of **43** (20.0 mg, 41.6 µmol), in DCM (0.4 mL, 0.1M) was stirred to -78 °C under nitrogen. Triethylaluminium in hexanes (100 µL, 2.0 M, 208 µmmol) was added in dropwise and the mixture was stirred for 2 hours. Saturated sodium bicarbonate (1 mL) was added, and the mixture was allowed to warm to room temperature. The mixture was diluted with Rochelle's Salt and extracted 5 times with DCM, organic layers were combined and dried over sodium sulfate. 48% yield as determined by 1H NMR spectroscopic analysis using internal standard. The crude mixture purified by silica flash chromatography (1 % IPA/50 % EtOAc/n-hexanes), to afford a white powder, compound **49** (4.3 mg, 32% isolated).

**Compound 49:** **IR** (neat): υ = 2958, 2928, 2859, 1717, 1691, 1462, 1383, 1288, 1099, 1066, 1039, 857, 730, 577 $cm^{-1}$; $[\alpha]_D^{20}$= +28 (c 0.29 in $CH_2Cl_2$), **$^1$H NMR** (500 MHz, $D_3CCN$): δ 9.03 (br, 1H, NH), 7.39 (q, *J* = 1.2 Hz, 1H), 5.46 (s, 1H), 4.65 (q, *J* = 6.5 Hz, 1H), 4.43 (d, *J* = 11.2 Hz, 1H), 4.37 (s, 1H), 3.97 (d, *J* = 11.2, 1H), 3.83 (s, 1H), 1.87 (d, *J* = 1.3 Hz, 3H), 1.51 (s, 3H), 1.42 (s, 3H), 1.24 (d, *J* = 6.5 Hz, 3H); **$^{13}$C NMR** (126 MHz, $D_3CCN$): δ 164.3, 151.1, 135.1, 110.7, 101.8, 89.2, 79.7, 79.3, 76.5, 73.7, 60.7, 29.3, 19.5, 13.3, 12.5; **HRMS** ($ESI^+$) calcd for $[C_{15}H_{20}N_2O_6+H]^+$ 325.1400 m/z found: 325.1394 m/z.

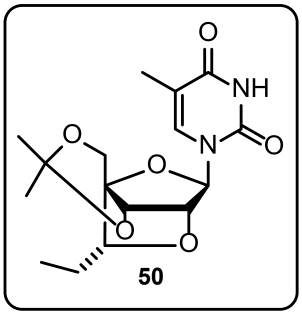

***Preparation of (R)-ethyl locked nucleoside***

To a solution of **43** (20.0 mg, 41.6 µmol), in DCM (0.4 mL, 0.1M), triethylaluminum (0.2 mL, 1M in n-hexanes, 208 µmol) was added and stirred for 3.5 hours at -78 °C under nitrogen. Saturated sodium bicarbonate (0.5 mL) was added, the mixture was raised to room temperature a diluted with a Rochelle's salt solution. The crude mixture was extracted 5 times with DCM, organic layers were combined and dried over sodium sulfate. The crude mixture purified preparative HPLC (2 % MeCN/Water to 98 % MeCN/Water), to afford a white powder, compound **50** (6.3 mg, 45 %).

**Compound 50: IR** (neat): υ = 2959, 2928, 1718, 1463, 1268, 1100, 1045, 1019, 731, 580 cm$^{-1}$; $[\alpha]_D^{20}$= +31 (c 0.29 in $CH_2Cl_2$), **$^1$H NMR** (500 MHz, $D_3CCN$): δ 9.04 (br, 1H), 7.38 (d, *J* = 1.5 Hz, 1H), 5.44 (s, 1H), 4.44 (d, *J* = 11.2 Hz, 1H), 4.41 – 4.36 (m, 2H), 4.03 (d, *J* = 11.2 Hz, 1H), 3.82 (s, 1H), 1.87 (d, *J* = 1.3 Hz, 3H), 1.69 – 1.55 (m, 2H), 1.51 (s, 3H), 1.43 (s, 3H), 1.05 (t, *J* = 7.4 Hz, 3H); **$^{13}$C NMR** (126 MHz, $D_3CCN$): δ 164.6, 151.0, 135.2, 110.7, 101.8, 89.3, 81.8, 79.7, 79.2, 74.1, 61.2, 29.3, 22.3, 19.6, 12.5, 11.2; **HRMS** (ESI$^+$) calcd for $[C_{16}H_{22}N_2O_6+H]^+$ 339.1550 m/z found: 339.1560 m/z.

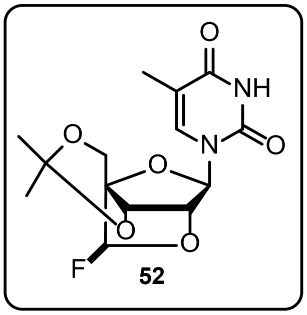

***Preparation of fluoro-locked nucleoside and fluorobutoxy-locked nucleoside***

To a solution of XtalFluor-M (17.9 mg, 73.5 µmol) and TEA (12.4 mg, 123 µmol) in DCM (0.6 mL, 0.1 M), **42** (20.0 mg, 61.3 µmol) in THF (0.6 mL, *totalling* 0.05 M) was slowly added stirred for 2.5 hours at -78 °C to 0 °C under nitrogen. Saturated sodium bicarbonate (0.5 mL) was added, the mixture was raised to room temperature and diluted with water (2 mL). The crude mixture was extracted 5 times with DCM, organic layers were combined and dried over sodium sulfate. The crude mixture purified preparative HPLC (2 % MeCN/Water to 98 % MeCN/Water), to afford compound **52** (2.3 mg, 11%).

**Compound 52: IR** (neat): υ = 2959, 2928, 1721, 1463, 1268, 1117, 1105, 1019, 731 cm$^{-1}$; $[\alpha]_D^{20}$= +16 (c 0.06 in $CH_2Cl_2$), **$^1$H NMR** (600 MHz, $D_3CCN$): δ 9.02 (br, 1H), 7.38 (q, *J* = 1.3 Hz, 1H), 6.29 (d, $J_{H-F}$ = 71.5 Hz, 1H), 5.64 (s, 1H), 4.72 (s, 1H), 4.48 (d, J = 11.4 Hz, 1H), 4.16 (d, *J* = 11.3 Hz, 1H), 4.00 (s, 1H), 1.87 (d, J = 1.3 Hz, 3H), 1.53 (s, 3H), 1.43 (s, 3H); **$^{13}$C NMR** (126 MHz, $D_3CCN$): δ 164.5, 150.9, 135.3, 113.0 (d, $J_{C-F}$ = 228.9 Hz), 111.1, 102.3, 88.4, 82.2 (d, $J_{C-F}$ = 2.7 Hz), 78.2 (d, $J_{C-F}$ = 18.1 Hz), 75.6, 59.7, 28.9, 19.6, 12.5; **$^{19}$F NMR** (471 MHz, $D_3CCN$): δ -153.9; **HRMS** (ESI$^+$) calcd for $[C_{14}H_{17}FN_2O_6+H]^+$ 329.1143 m/z found: 329.1147 m/z.

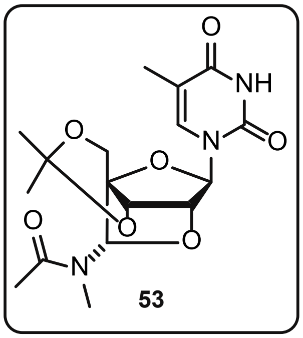

***Preparation of N-methylacetamide locked nucleoside***

To a solution of **42** (10.0 mg, 30.6 µmol) and potassium carbonate (21.2 mg, 153 µmol) in MeCN (0.6 mL, 0.05 M), methylamine (23 µL, 33 w. % in ethanol, 184 µmol) was added and stirred at 50 °C for 6 hours until the reaction was completed determined by TLC analysis. The solution was filtered through celite, and volatiles were removed under reduced. The crude mixture was added to a solution of pyridine (11 uL, 0.13 mmol), acetic anhydride (7.5 uL, 80 µmol) and DMAP (0.3 mg, 2 µmol) in DCM (0.2 mL, 0.15M) at 0 °C and stirred for 5 hours. Volatiles were removed under reduced pressure. 37% yield as determined by 1H NMR spectroscopic analysis using internal standard. The resulting crude mixture was purified preparative HPLC (2 % MeCN/Water to 98 % MeCN/Water), to afford a white powder, compound **53** (1.9 mg, 19% isolated).

**Compound 53: IR** (neat): υ = 2958, 2928, 2859, 1720, 1463, 1377, 1268, 1117, 1102, 1045, 1019, 873, 730 $cm^{-1}$; $[\alpha]_D^{20}$= +9 (c 0.13 in $CH_2Cl_2$), **$^1$H NMR** (600 MHz, $D_3CCN$, 320 K): δ 8.93 (s, 1H), 7.35 (s, 1H), 6.84*, 6.31* (br, 1H) 5.76 (s, 1H), 4.66 (s, 1H), 4.44 (d, J = 11.0 Hz, 1H), 4.04 (d, J = 11.4 Hz, 1H), 3.94 (s, 1H), 3.06 (s, 3H), 2.13 (s, 3H), 1.89 (s, 3H), 1.52 (s, 3H), 1.48 (s, 3H); **$^{13}$C NMR** (150 MHz, $D_3CCN$, 320 K): δ 173.6, 164.6, 151.0, 135.1, 111.3, 102.5, 89.3, 88.3$^†$, 84.5$^†$, 79.8, 79.1, 74.5, 60.8, 31.2$^‡$, 29.5, 27.3$^‡$ 23.6, 19.7, 12.6; **HRMS** ($ESI^+$) calcd for $[C_{17}H_{23}N_3O_7+H]^+$ 382.1609 m/z found: 382.1592 m/z and $[C_{17}H_{23}N_3O_7+Na]^+$ 404.1428 m/z found: 404.1418 m/z. * $^{† ‡}$(600 MHz, $D_3CCN$, 280 K) as rotamers signals.

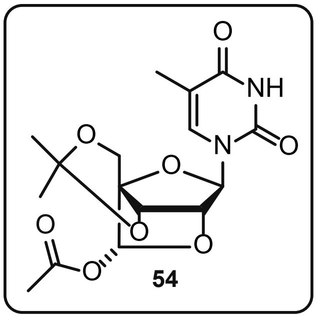

***Preparation of acetyl locked nucleoside***

A solution of **42** (20.0 mg, 61.3 µmol), pyridine (50 µL, 613 µmol), DMAP (0.7 mg, 6 µmol) and acetic anhydride (7 µL, 73.5 µmol) in DCM (0.6 mL, 0.1 M) was stirred to 0 °C under nitrogen for 3 hours. A solution of saturated ammonium chloride (1 mL) was added, and the mixture was extracted 5 times with DCM, organic layers were combined, dried over sodium sulfate and removed under reduced pressure, to afford a white powder, compound **54** (22.6 mg, Quant.).

**Compound 54: IR** (neat): υ = 2958, 2927, 2858, 1719, 1408, 1380, 1265, 1100, 1019, 944, 730, 580 $cm^{-1}$; $[\alpha]_D^{20}$= +42 (c 0.09 in $CH_2Cl_2$), **$^1$H NMR** (600 MHz, $D_3CCN$): δ 9.23 (s, 1H), 7.40 (s, 1H), 6.52 (s, 1H), 5.63 (s, 1H), 4.61 (s, 1H), 4.45 (d, *J* = 11.3 Hz, 1H), 4.09 (d, *J* = 11.3 Hz, 1H), 3.96 (s, 1H), 2.10 (s, 3H), 1.88 (s, 3H), 1.54 (s, 3H), 1.45 (s, 3H); **$^{13}$C NMR** (150 MHz, $D_3CCN$): δ 171.3, 164.7, 151.1, 135.3, 111.0, 102.2, 97.9, 88.6, 81.2, 78.7, 74.9, 60.0, 29.1, 21.3, 19.6, 12.5; **HRMS** ($ESI^+$) calcd for $[C_{16}H_{20}N_2O_8+H]^+$ 369.1292 m/z found: 369.1300 m/z.

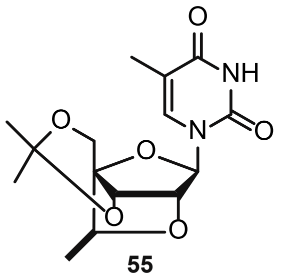

55

***Preparation of (S)-methyl locked nucleoside***

A room temperature stirred solution of alkene **17** (150.0 mg, 0.456 mmol) and Pd/C (10%, 49.5 mg, 46.5 μmol) in ethyl acetate (4.65 mL) was put under an inert environment. $H_2$ was introduced via a balloon and the solution was sparged with $H_2$ for 15 minutes. The needle was then pulled above the surface of the solvent and left in the headspace in the flask. The resulting mixture was maintained for 1 hour with stirring. The reaction mixture was then filtered through celite with EtOAc and concentrated under reduced pressure to afford compound **55** as a white solid (145.8 mg, 0.450 mmol, 97%).

**Compound 55: IR** (neat): υ = 3455, 2974, 1691, 1273, 1200, 1045, 1005, 855, 817 $cm^{-1}$; $[\alpha]_D^{20}$= +2.6 (c 0.44 in $CH_2Cl_2$), **$^1$H NMR** (400 MHz, $D_3CCN$) δ 9.06 (s, 1H), 7.36 (q, *J* = 1.2 Hz, 1H), 5.44 (s, 1H), 4.52 (d, *J* = 11.2 Hz, 1H), 4.51 (s, 1H), 4.16 (dd, *J* = 11.2, 0.9 Hz, 1H), 4.07 (q, *J* = 6.8 Hz, 1H), 3.75 (s, 1H), 1.87 (d, *J* = 1.3 Hz, 3H), 1.53 (s, 3H), 1.40 (s, 3H), 1.37 (d, *J* = 6.8 Hz, 3H); **$^{13}$C NMR** (151 MHz, $D_3CCN$) δ 164.56, 150.97, 135.23, 110.71, 101.33, 89.38, 82.98, 78.59, 78.08, 72.52, 61.54, 28.36, 19.57, 19.00, 12.51; **HRMS** ($ESI^+$) calcd for $[C_{15}H_{21}N_2O_6+H]^+$ 325.1394 m/z found: 325.1397 m/z.

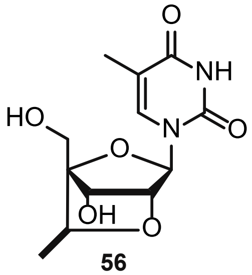

56

***Deprotection of (S)-methyl locked nucleoside.***

At room temperature, protected LNA **55** (145.8 mg, 0.450 mmol) was dissolved in 1 mL of 90% TFA in water and the resulting mixture was maintained for 10 minutes. The reaction mixture was then concentrated under reduced pressure. Afterwards, the crude product was dissolved in methanol to azeotrope off the TFA, this was repeated 3 times. After all solvent was removed, compound **56** was obtained as a white solid (108.3 mg, 0.381 mmol, 85%).

**Compound 56: IR** (neat): υ = 3374, 2932, 2821, 1681, 1467, 1274, 1047, 833, 571 $cm^{-1}$; $[\alpha]_D^{20}$= +15.2 (c 1.50 in MeCN), **$^1$H NMR** (600 MHz, MeOD) δ 7.70 (s, 1H), 5.50 (s, 1H), 4.33 (s, 1H), 4.06 (d, *J* = 8.8 Hz, 2H), 3.96 (s, 2H), 1.88 (d, *J* = 1.2 Hz, 3H), 1.32 (d, *J* = 6.8 Hz, 3H); **$^{13}$C NMR** (151 MHz, MeOD) δ 166.5, 151.9, 136.9, 110.6, 90.5, 88.1, 82.3, 81.3, 71.3, 57.8, 16.6, 12.6; **HRMS** ($ESI^+$) calcd for $[C_{12}H_{16}N_2O_6+H]^+$ 285.1081 m/z found: 285.1084 m/z.

Matches literature data from: Blade, H.; Bradley, D.; Diorazio, L.; Evans, T.; Hayter, B. R.; Howell, G. P. Modular Synthesis of Constrained Ethyl (CEt) Purine and Pyrimidine Nucleosides. *Journal of Organic Chemistry* **2015**, *80* (10), 5337–5343. https://doi.org/10.1021/acs.joc.5b00607.

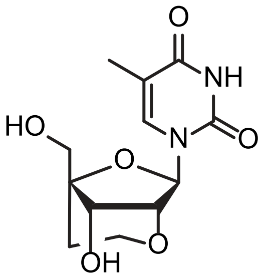

**4**

***Preparation of ethylene bridged nucleoside***

A mixture of sodium hydride (60% in oil, 52 mg, 1.26 mmol) and TMSOI (282 mg, 1.26 mmol) were placed in an oven-dried flask and dry DMF (3 mL) was added to the mixture at room temperature. After hydrogen evolution ceased, the milky solution turned clear. The reaction mixture was stirred for 15 min at room temperature and then shifted to -40 °C. Compound **27** (100 mg, 0.316 mmol) was dissolved in dry DMF (0.5 mL), added to the clear solution dropwise at -40 °C and slowly warm to room temperature. The reaction mixture was stirred for 3 days and then was quenched using 2.0 mL of 1.0 M aq. $KH_2PO_4$ and extracted with EtOAc 4 times. The organic extracts were combined, dried over sodium sulfate, and concentrated. Afterwards the crude mixture was dissolved in MeOH (1 mL) and TFA (0.13 mL, 1.58 mmol) was added. The reaction was stirred at room temperature for 30 minutes then the solvent was removed under reduced pressure. The crude product was then dissolved in methanol again to azeotrope off the TFA this was repeated 3 times. An NMR yield was obtained for the deprotected ENA (23.0 mg, 26% over 2 steps). Purification proceeded through preparative HPLC (2 % MeCN/Water to 50 % MeCN/Water) to afford compound **4** as a white powder.

***0.4 mmol scale preparation of compound 4***

A mixture of sodium hydride (60% in oil, 520 mg, 12.96 mmol) and TMSOI (2.92 g, 13.28 mmol) was placed in an oven-dried flask, and dry DMF (30 mL) was added to the mixture at room temperature. After hydrogen evolution ceased, the milky solution turned clear. The reaction mixture was stirred for 25 min at room temperature and then shifted to -40 °C. Compound **27** (1.0 g, 3.16 mmol) was dissolved in dry DMF (5.0 mL), added to the clear solution dropwise at -40 °C, and slowly warmed to room temperature. The reaction mixture was stirred for 3 days and then was quenched using 20.0 mL of 1.0 M aq. $KH_2PO_4$ and extracted with EtOAc 5 times. The organic extracts were combined, dried over anhydrous sodium sulfate, and concentrated. The resulting oil was purified by flash chromatography (gradient of EtOAc: hexanes 1:1 to 4:1) to afford protected ENA **38** as a white solid (140 mg, 14%). Afterward, ENA **38** was dissolved in MeOH (10.0 mL), and TFA (1.5 mL, 15.8 mmol) was added. The reaction was stirred at room temperature for 30 minutes then the solvent was removed under reduced pressure. The crude was then dissolved in methanol again to azeotrope off the TFA. This was repeated 3 times. Compound **4** was collected as a white powder (120.0 mg, 97%).

**Compound 4: IR** (neat): υ = 3384, 2966, 2928, 1686, 1468, 1274, 1105, 1054, 822 $cm^{-1}$; $[\alpha]_D^{20}$= +26.0 (c 0.4 in MeOH), **$^1$H NMR** (500 MHz, MeOD) δ 8.28 (d, *J* =f 1.3 Hz, 1H), 4.10 (d, *J* = 3.3 Hz, 1H), 4.05 (d, *J* = 3.3 Hz, 1H), 3.98 (m, 2H), 3.75 (d, *J* = 12.3 Hz, 1H), 3.68 (d, *J* = 12.4 Hz, 1H), 2.13 (ddd, *J* = 13.3, 11.6, 7.4 Hz, 1H), 1.87 (d, *J* = 1.2 Hz, 3H), 1.33 (dd, *J* = 13.2, 4.3 Hz, 1H); **$^{13}$C NMR** 151 MHz, MeOD) δ 166.7, 152.0, 137.8, 110.3, 86.7, 85.9, 80.2, 65.8, 62.8, 61.7, 27.9, 12.6;; **HRMS** (ESI$^+$) calcd for $[C_{12}H_{16}N_2O_6+H]^+$ 285.1081 m/z found: 285.1093 m/z.

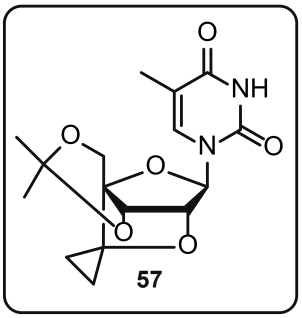

**57**

***Preparation of cyclopropyl locked nucleoside***

To a cold, 0 °C, stirred solution of alkene **17** (75 mg, 0.23 mmol) in $CH_2Cl_2$ (2.3 mL) was added diiodomethane (94 µL, 1.2 mmol), followed by diethylzinc (1.2 mL, 1.0 M in hexanes, 1.2 mmol). The resulting mixture was maintained at 0 °C and stirred for 2 hours or until completion. The reaction mixture was then diluted with a saturated ammonium chloride solution (5 mL) and DCM (8 mL) and the phases were separated. The aqueous phase was washed with DCM (3 x 15 mL) and the organic phases were combined and washed with brine, dried over MgSO4, filtered, and concentrated under reduced pressure. The resulting crude mixture was purified by flash chromatography (gradient of EtOAc:hexanes 0:1 to 4:1) to afford protected LNA **57** as a white solid (61.6 mg, 79%).

**Compound 57: IR** (neat): υ = 2990, 1691, 1273, 1199, 1108, 1052, 859, 761 $cm^{-1}$; $[\alpha]_D^{20}$= +16.8 (c 0.63 in $CH_2Cl_2$), **$^1$H NMR** (600 MHz, $D_3CCN$) δ 9.17 (s, 1H), 7.42 (q, *J* = 1.3 Hz, 1H), 5.57 (s, 1H), 4.51 (s, 1H), 4.41 (d, *J* = 11.4 Hz, 1H), 3.89 (s, 1H), 3.61 (dd, *J* = 11.4, 1.0 Hz, 1H), 1.88 (d, *J* = 1.3 Hz, 3H), 1.52 (s, 3H), 1.40 (s, 3H), 1.00 (m, 2H), 0.79 (m, 2H); **$^{13}$C NMR** (151 MHz, $D_3CCN$) δ 164.71, 151.04, 135.42, 110.68, 101.15, 89.39, 79.24, 78.85, 73.23, 68.54, 59.88, 28.84, 19.67, 12.50, 11.56, 5.18; **HRMS** ($ESI^+$) calcd for $[C_{16}H_{21}N_2O_6+H]^+$ 337.1394 m/z found: 337.1390 m/z.

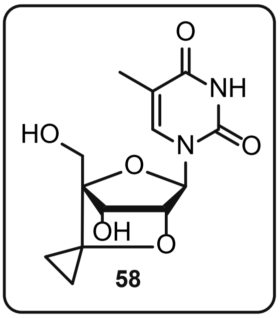

**58**

***Deprotection of cyclopropyl locked nucleoside***

At room temperature, protected LNA **57** (61.6 mg, 0.183 mmol) was dissolved in 1 mL of 90% TFA in water and the resulting mixture was maintained for 15 minutes. The reaction mixture was then concentrated under reduced pressure. Afterwards, the crude product was dissolved in methanol and the solvent was removed under reduced pressure. This was repeated 3 times. The resulting crude mixture was purified by flash chromatography (MeOH:DCM 1:10) to afford protected LNA **58** as a white solid (42.1 mg, 78%).

**Compound 58: IR** (neat): υ = 3389, 2994, 2925, 1691, 1473, 1270, 1107, 1043, 862, 576 $cm^{-1}$; $[\alpha]_D^{20}$= +14.0 (c 0.24 in MeCN), **$^1$H NMR** (600 MHz, $CD_3CN$) δ 9.03 (s, 1H), 7.59 (d, *J* = 1.3 Hz, 1H), 5.56 (s, 1H), 4.23 (s, 1H), 4.13 (s, 1H), 3.69 (d, *J* = 12.7 Hz, 1H), 3.53 (d, *J* = 12.7 Hz, 1H), 1.84 (d, *J* = 1.2 Hz, 3H), 0.77 (m, 4H); **$^{13}$C NMR** (151 MHz, $CD_3CN$) δ 164.7, 151.0, 135.9, 110.0, 89.3, 87.5, 80.4, 72.1, 68.2, 56.8, 12.7, 9.8, 5.0; **HRMS** ($ESI^+$) calcd for $[C_{13}H_{16}N_2O_6 +H]^+$ 297.1081 m/z found: 297.1079 m/z.

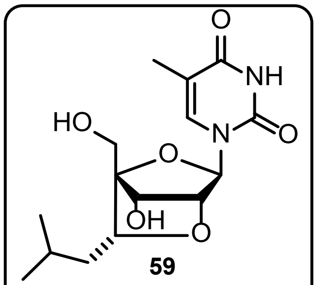

**59**

***Preparation of (R)-isobutyl locked nucleoside***

A solution of **43** (15.0 mg, 31.2 µmol), in DCM (0.31 mL, 0.1M) was stirred to -50 °C under nitrogen. Triisobutylaluminium in hexanes (187 µL, 1.0 M, 187 µmol) was added in dropwise and the mixture was stirred for 7 hours. Saturated sodium bicarbonate (1 mL) was added, and the mixture was allowed to warm to room temperature. The mixture was diluted with Rochelle's Salt and extracted 3 times with $EtO_2$ and 3 times with EtOAc, organic layers were combined and dried over sodium sulfate. An NMR yield was taken of the acetonide protected product **51** (4.6 mg, 40%). The crude

mixture was deprotected with TFA (5 drops) in MeOH (1 mL) and then purified by preparative HPLC (2 % MeCN/Water to 98 % MeCN/Water) to afford compound **59** as a white powder (2.1 mg, 21% over 2 steps).

**Compound 59: IR** (neat): υ = 3396, 2956, 2934, 2875, 1687, 1469, 1264, 1052, 717, 628 $cm^{-1}$; $[\alpha]_D^{20}$= +12 (c 0.19 in $CH_2Cl_2$), **$^1$H NMR** (600 MHz, $CD_3CN$) δ 8.99 (s, 1H), 7.62 (q, *J* = 1.2 Hz, 1H), 5.41 (s, 1H), 4.14 (s, 1H), 4.10 (dd, *J* = 10.4, 2.7 Hz, 1H), 4.07 (s, 1H), 3.73 (s, 2H), 1.82 (s, 3H), 1.79 (m, 4H), 1.61 (ddd, *J* = 14.0, 10.4, 5.2 Hz, 1H), 1.22 (m, 2H), 0.96 (d, *J* = 6.7 Hz, 3H), 0.91 (d, *J* = 6.6 Hz, 3H); **$^{13}$C NMR** (151 MHz, $CD_3CN$) δ 164.71, 151.00, 135.86, 109.94, 90.07, 87.06, 79.80, 78.74, 71.59, 57.00, 38.55, 26.05, 23.99, 22.22, 12.82.; **HRMS** (ESI$^+$) calcd for $[C_{15}H_{22}N_2O_6+H]^+$ 327.1551 m/z found: 327.1552 m/z.

## 3.0 NMR Spectra of Characterized Compounds

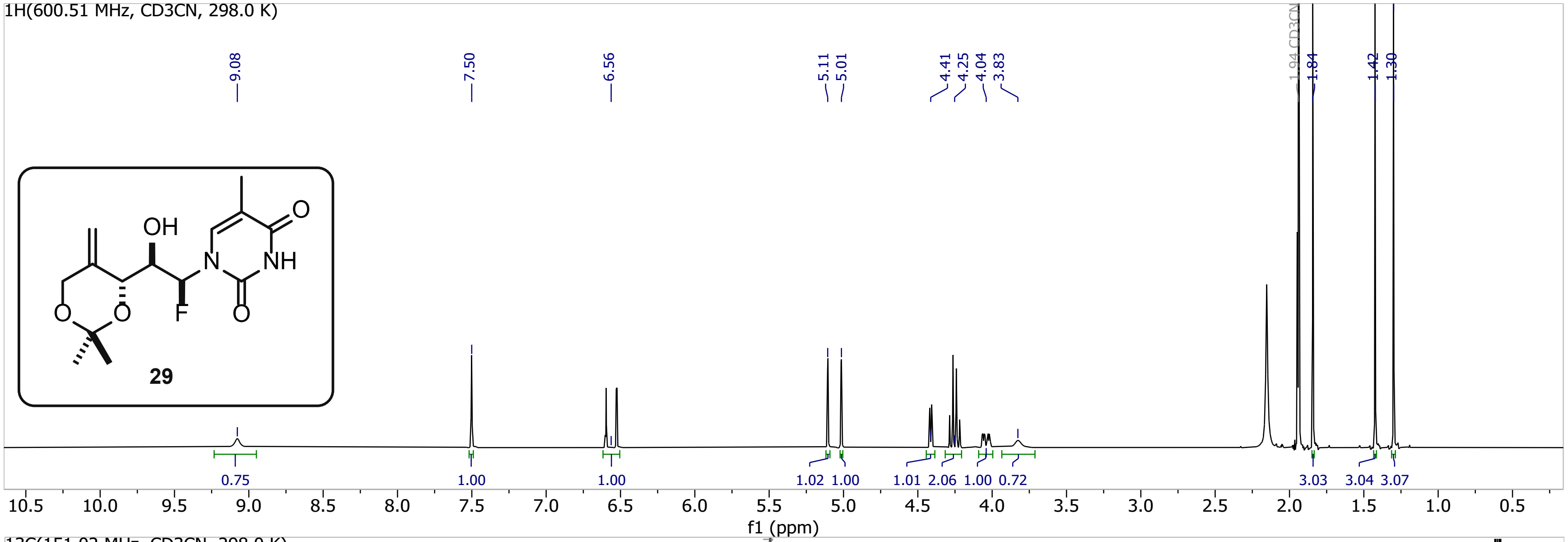
1H(600.51 MHz, CD3CN, 298.0 K)
9.08
7.50
6.56
5.11
5.01
4.41
4.25
4.04
3.83
1.94 CD3CN
1.84
1.42
1.30
OH
N
O
NH
O
O
O
F
29
0.75
1.00
1.00
1.02 1.00
1.01 2.06 1.00 0.72
3.03
3.04 3.07
10.5 10.0 9.5 9.0 8.5 8.0 7.5 7.0 6.5 6.0 5.5 5.0 4.5 4.0 3.5 3.0 2.5 2.0 1.5 1.0 0.5
f1 (ppm)

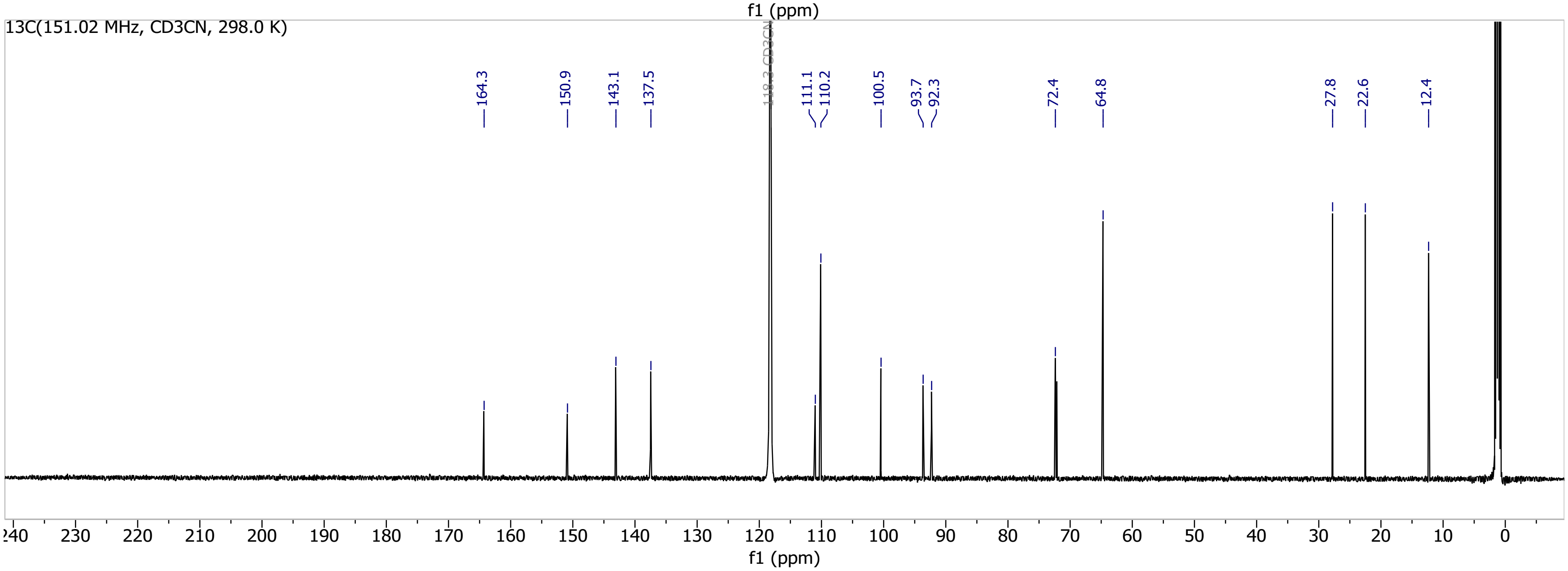
13C(151.02 MHz, CD3CN, 298.0 K)
164.3
150.9
143.1
137.5
118.3 CD3CN
111.1
110.2
100.5
93.7
92.3
72.4
64.8
27.8
22.6
12.4
240 230 220 210 200 190 180 170 160 150 140 130 120 110 100 90 80 70 60 50 40 30 20 10 0
f1 (ppm)

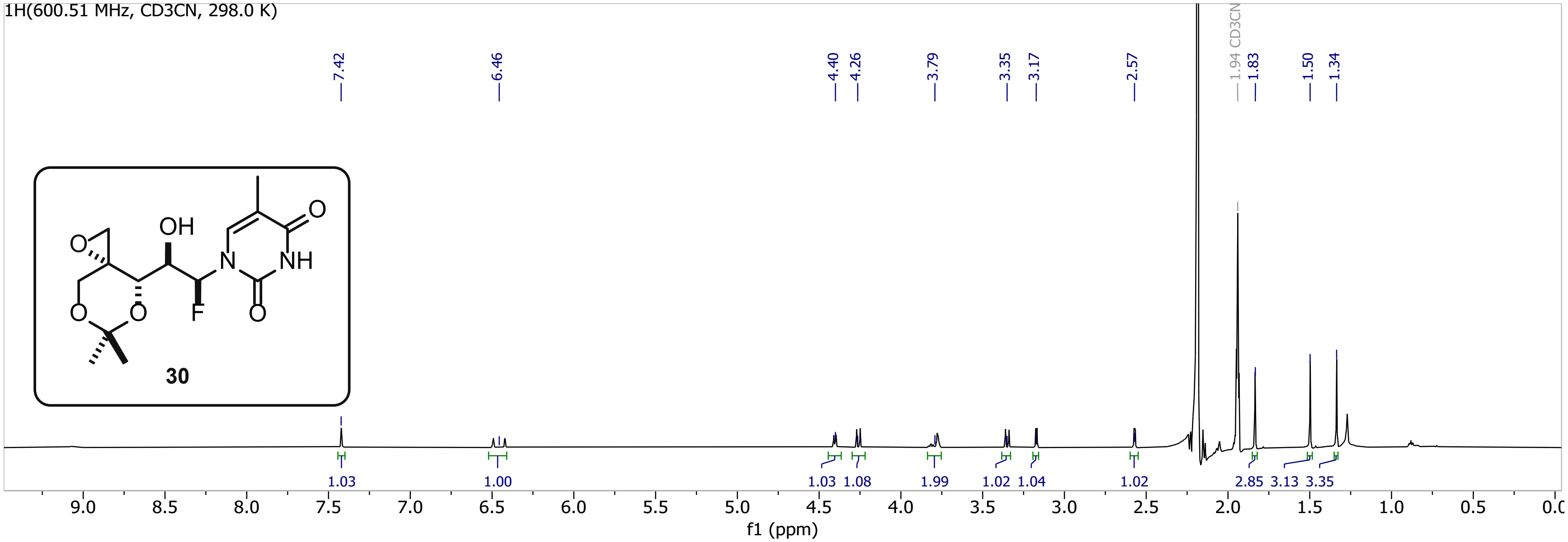
1H(600.51 MHz, CD3CN, 298.0 K)
7.42
6.46
4.40
4.26
3.79
3.35
3.17
2.57
1.94 CD3CN
1.83
1.50
1.34
OH
O
N
NH
O
O
F
O
O
30
1.03
1.00
1.03 1.08
1.99
1.02 1.04
1.02
2.85 3.13 3.35
9.0 8.5 8.0 7.5 7.0 6.5 6.0 5.5 5.0 4.5 4.0 3.5 3.0 2.5 2.0 1.5 1.0 0.5
f1 (ppm)

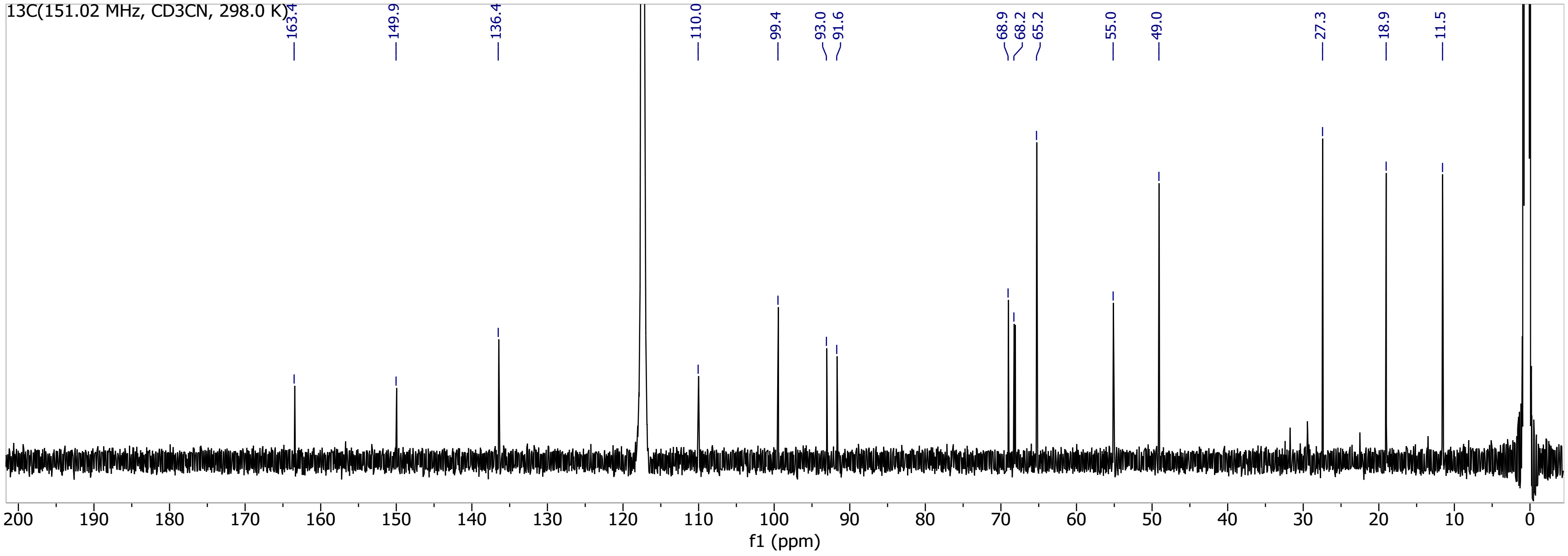
13C(151.02 MHz, CD3CN, 298.0 K)
163.4
149.9
136.4
110.0
99.4
93.0
91.6
68.9
68.2
65.2
55.0
49.0
27.3
18.9
11.5
200 190 180 170 160 150 140 130 120 110 100 90 80 70 60 50 40 30 20 10 0
f1 (ppm)

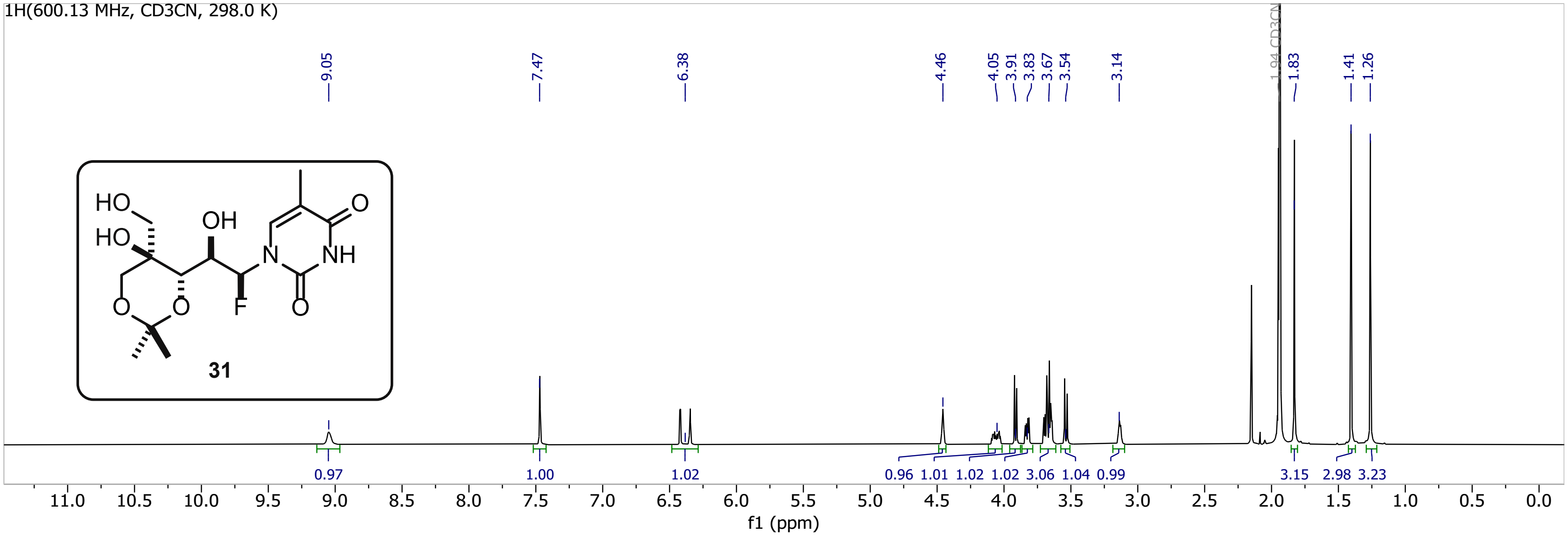
1H(600.13 MHz, CD3CN, 298.0 K)
9.05
7.47
6.38
4.46
4.05
3.91
3.83
3.67
3.54
3.14
1.94 CD3CN
1.83
1.41
1.26
HO
HO
OH
N
NH
O
O
O
O
F
31
0.97
1.00
1.02
0.96
1.01
1.02
1.02
3.06
1.04
0.99
3.15
2.98
3.23
11.0
10.5
10.0
9.5
9.0
8.5
8.0
7.5
7.0
6.5
6.0
5.5
5.0
4.5
4.0
3.5
3.0
2.5
2.0
1.5
1.0
0.5
0.0
f1 (ppm)

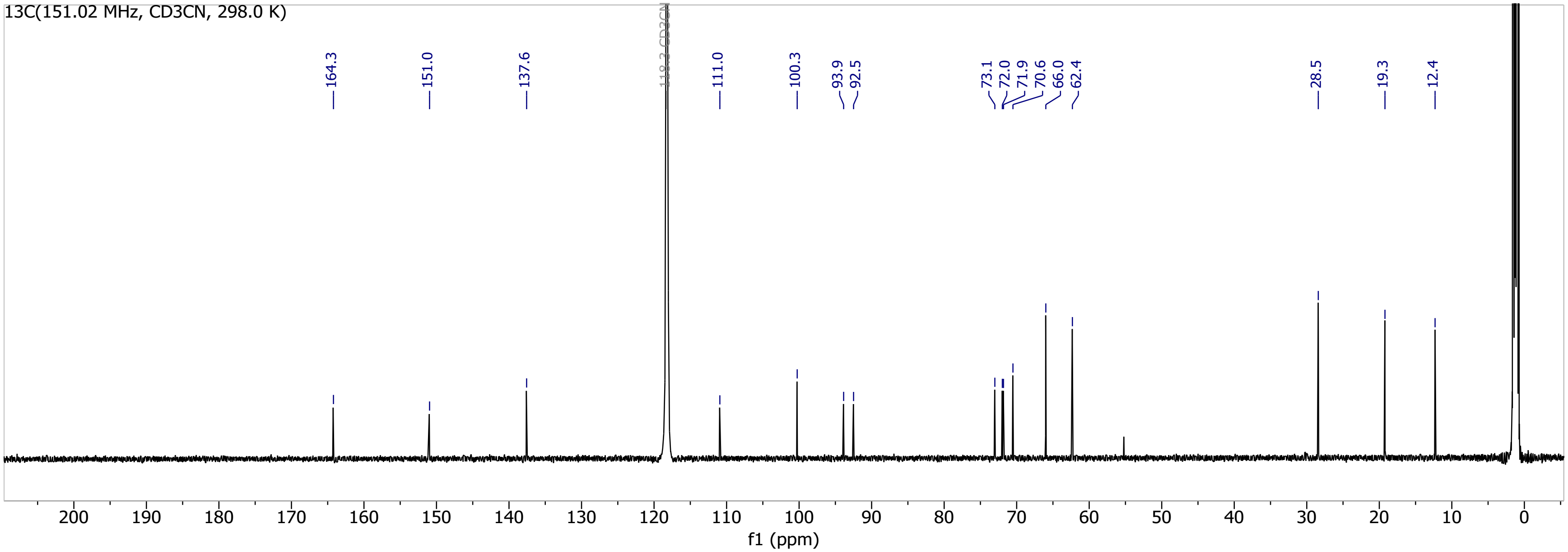
13C(151.02 MHz, CD3CN, 298.0 K)
164.3
151.0
137.6
118.3 CD3CN
111.0
100.3
93.9
92.5
73.1
72.0
71.9
70.6
66.0
62.4
28.5
19.3
12.4
200
190
180
170
160
150
140
130
120
110
100
90
80
70
60
50
40
30
20
10
0
f1 (ppm)

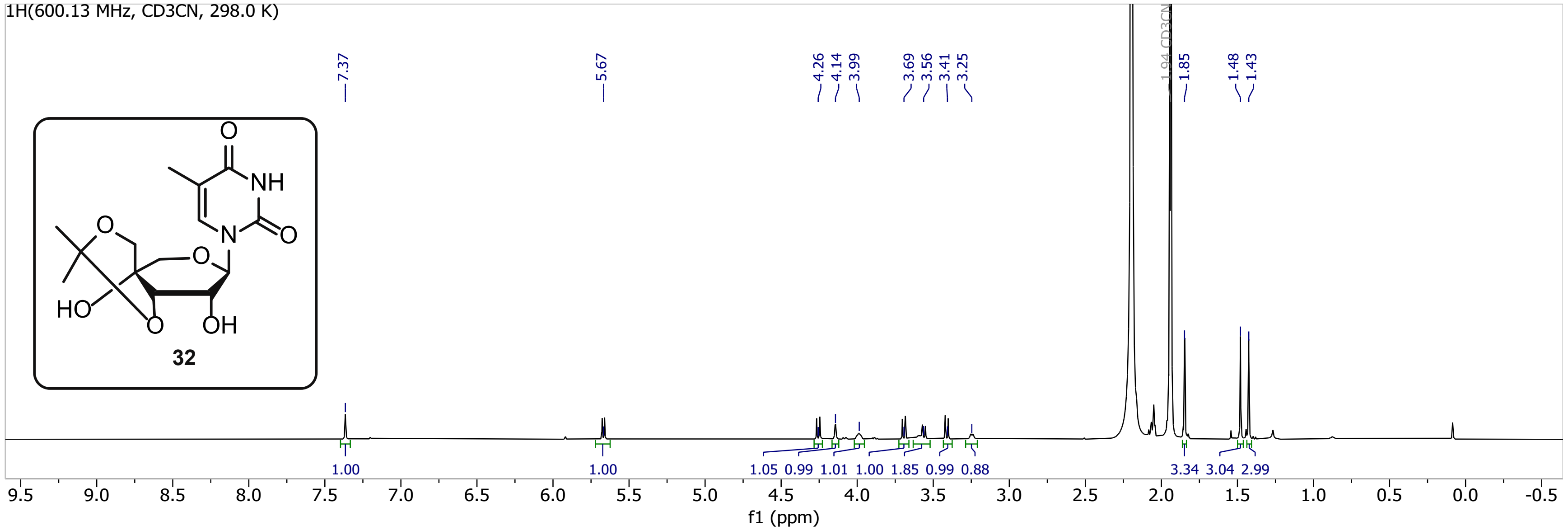
1H(600.13 MHz, CD3CN, 298.0 K)
7.37
5.67
4.26
4.14
3.99
3.69
3.56
3.41
3.25
1.94 CD3CN
1.85
1.48
1.43
O
NH
O
N
O
O
HO
O
OH
32
1.00
1.00
1.05 0.99 1.01 1.00 1.85 0.99 0.88
3.34 3.04 2.99
9.5 9.0 8.5 8.0 7.5 7.0 6.5 6.0 5.5 5.0 4.5 4.0 3.5 3.0 2.5 2.0 1.5 1.0 0.5 0.0 -0.5
f1 (ppm)

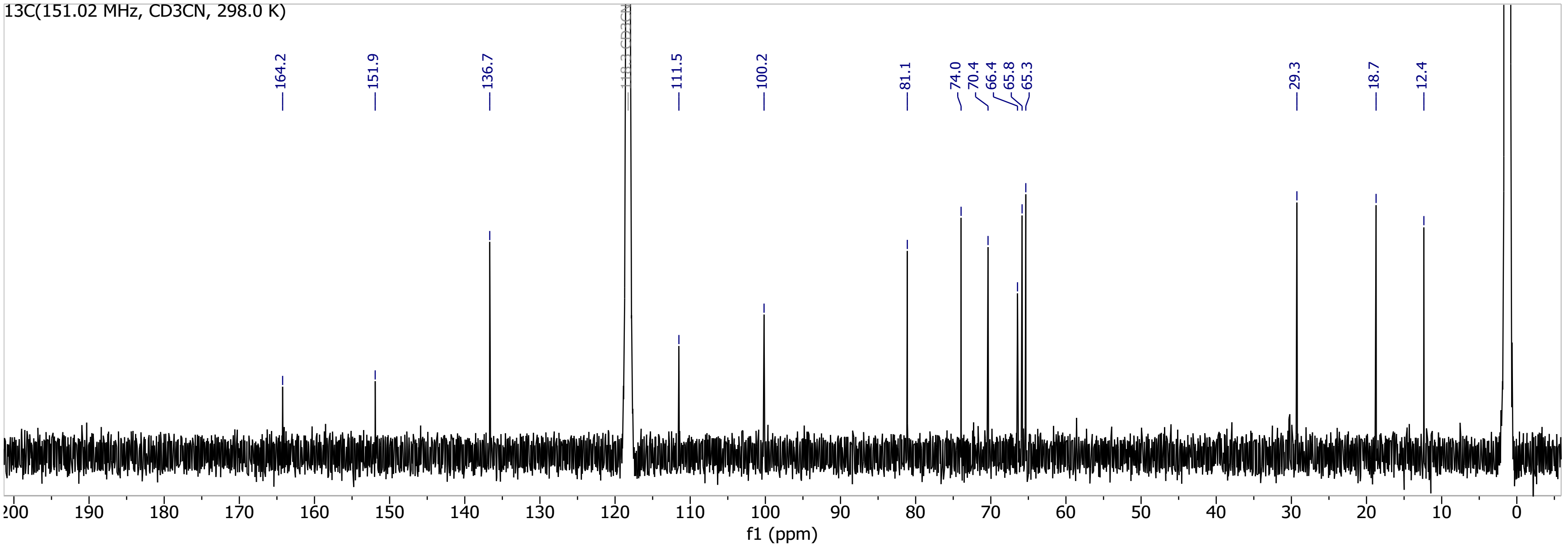
13C(151.02 MHz, CD3CN, 298.0 K)
164.2
151.9
136.7
118.3 CD3CN
111.5
100.2
81.1
74.0
70.4
66.4
65.8
65.3
29.3
18.7
12.4
200 190 180 170 160 150 140 130 120 110 100 90 80 70 60 50 40 30 20 10 0
f1 (ppm)

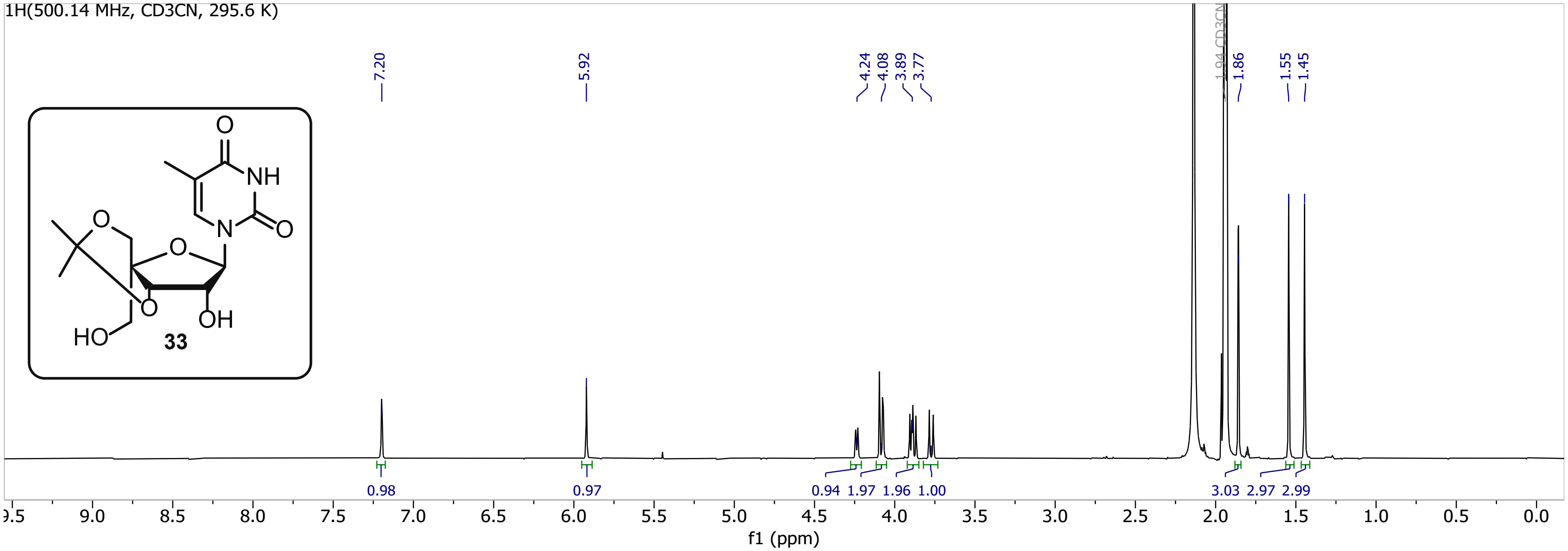
1H(500.14 MHz, CD3CN, 295.6 K)
33
7.20
5.92
4.24
4.08
3.89
3.77
1.94 CD3CN
1.86
1.55
1.45
0.98
0.97
0.94
1.97
1.96
1.00
3.03
2.97
2.99
f1 (ppm)

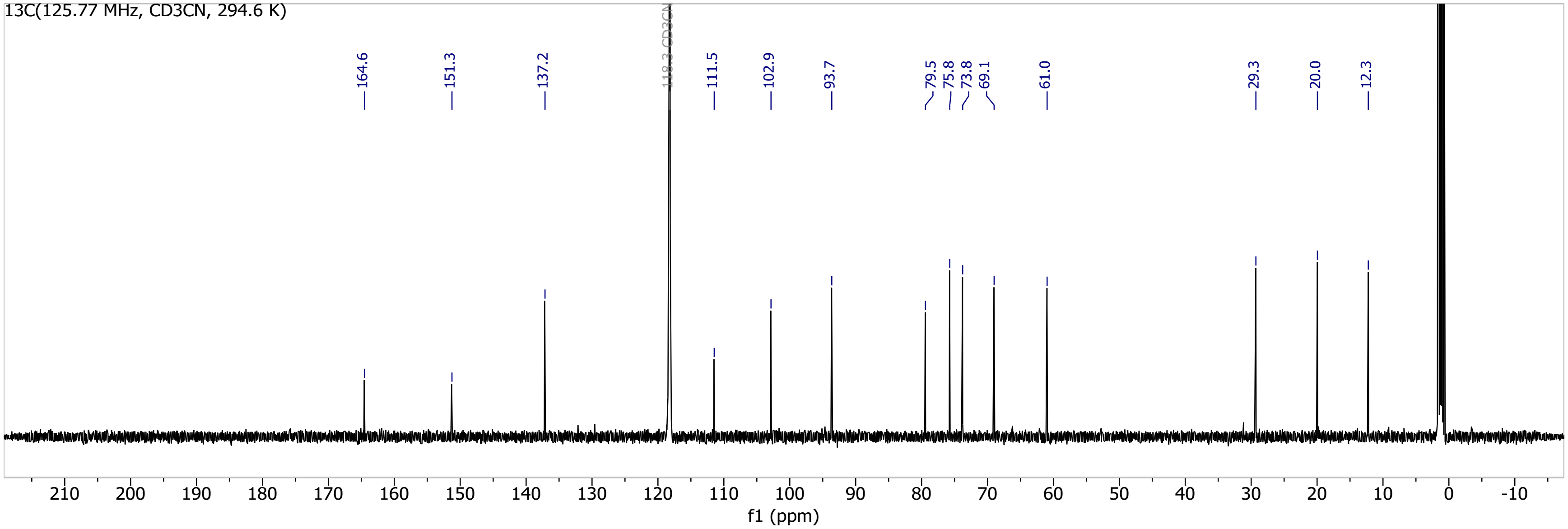
13C(125.77 MHz, CD3CN, 294.6 K)
164.6
151.3
137.2
118.3 CD3CN
111.5
102.9
93.7
79.5
75.8
73.8
69.1
61.0
29.3
20.0
12.3
f1 (ppm)

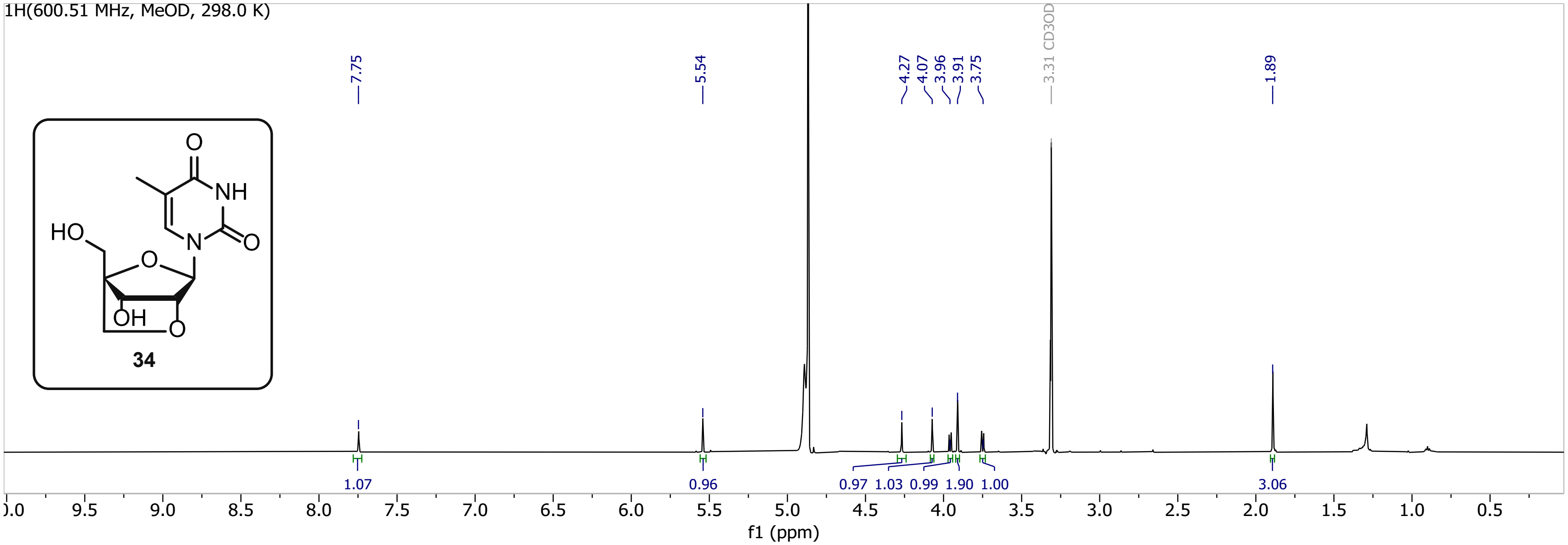
1H(600.51 MHz, MeOD, 298.0 K)
7.75
5.54
4.27
4.07
3.96
3.91
3.75
3.31 CD3OD
1.89
HO
O
NH
N
OH
34
1.07
0.96
0.97 1.03 0.99 1.90 1.00
3.06
f1 (ppm)

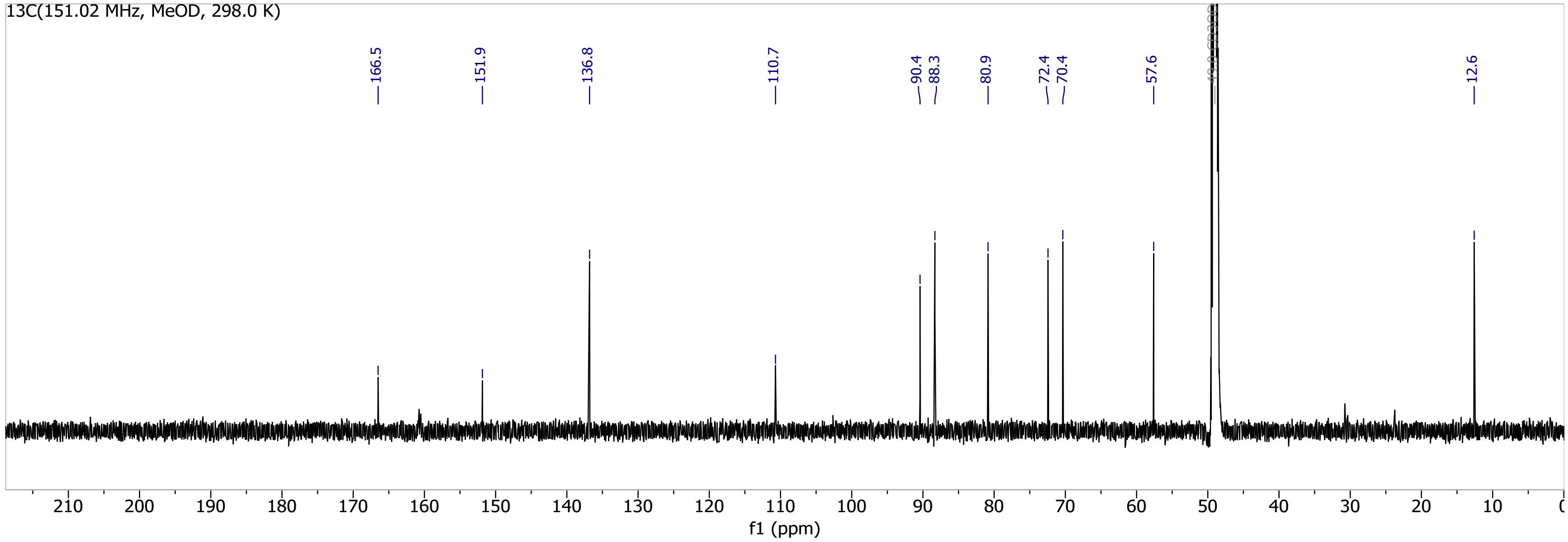
13C(151.02 MHz, MeOD, 298.0 K)
166.5
151.9
136.8
110.7
90.4
88.3
80.9
72.4
70.4
57.6
12.6
f1 (ppm)

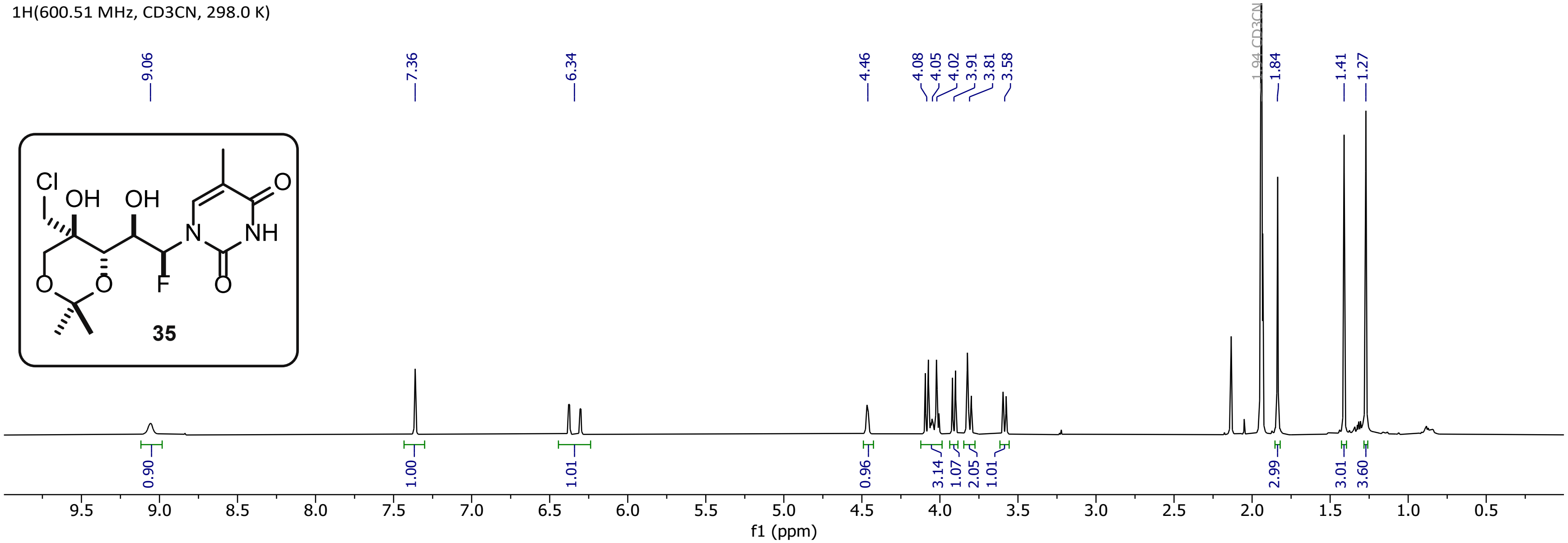
1H(600.51 MHz, CD3CN, 298.0 K)
9.06
7.36
6.34
4.46
4.08
4.05
4.02
3.91
3.81
3.58
1.94 CD3CN
1.84
1.41
1.27
Cl
OH
OH
N
NH
O
O
O
O
F
35
0.90
1.00
1.01
0.96
3.14
1.07
2.05
1.01
2.99
3.01
3.60
9.5
9.0
8.5
8.0
7.5
7.0
6.5
6.0
5.5
5.0
4.5
4.0
3.5
3.0
2.5
2.0
1.5
1.0
0.5
f1 (ppm)

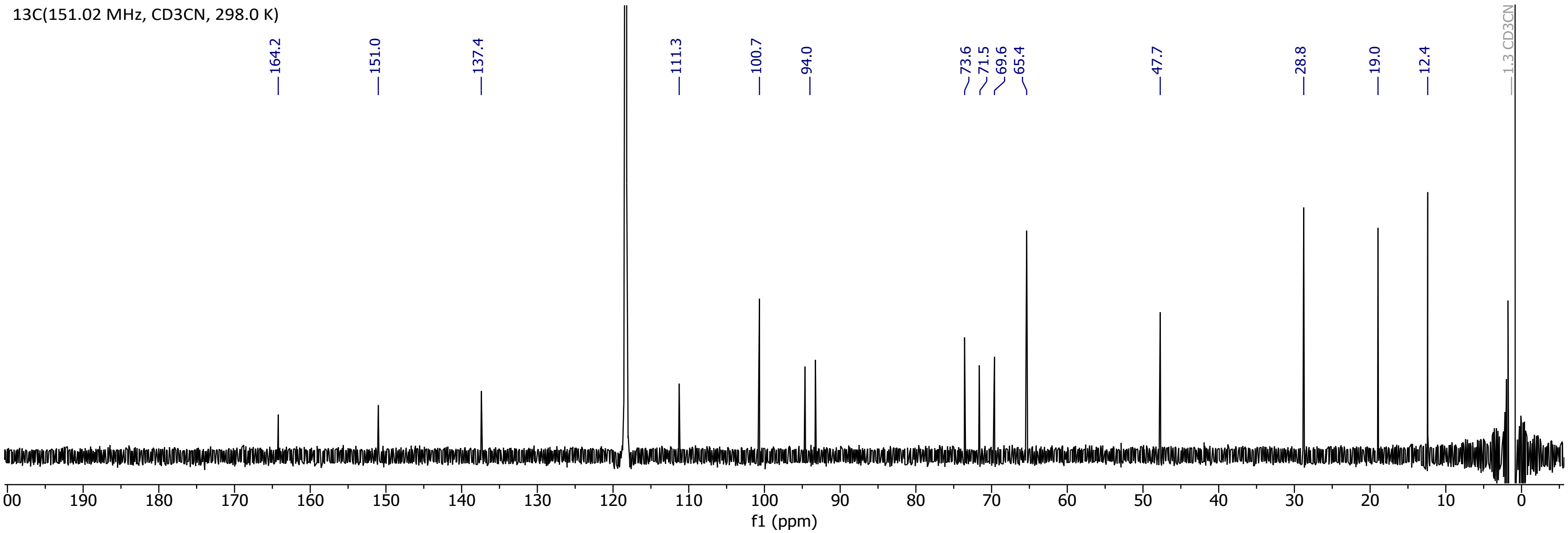
13C(151.02 MHz, CD3CN, 298.0 K)
164.2
151.0
137.4
111.3
100.7
94.0
73.6
71.5
69.6
65.4
47.7
28.8
19.0
12.4
1.3 CD3CN
00
190
180
170
160
150
140
130
120
110
100
90
80
70
60
50
40
30
20
10
0
f1 (ppm)

1H(600.13 MHz, CD3CN, 298.0 K)

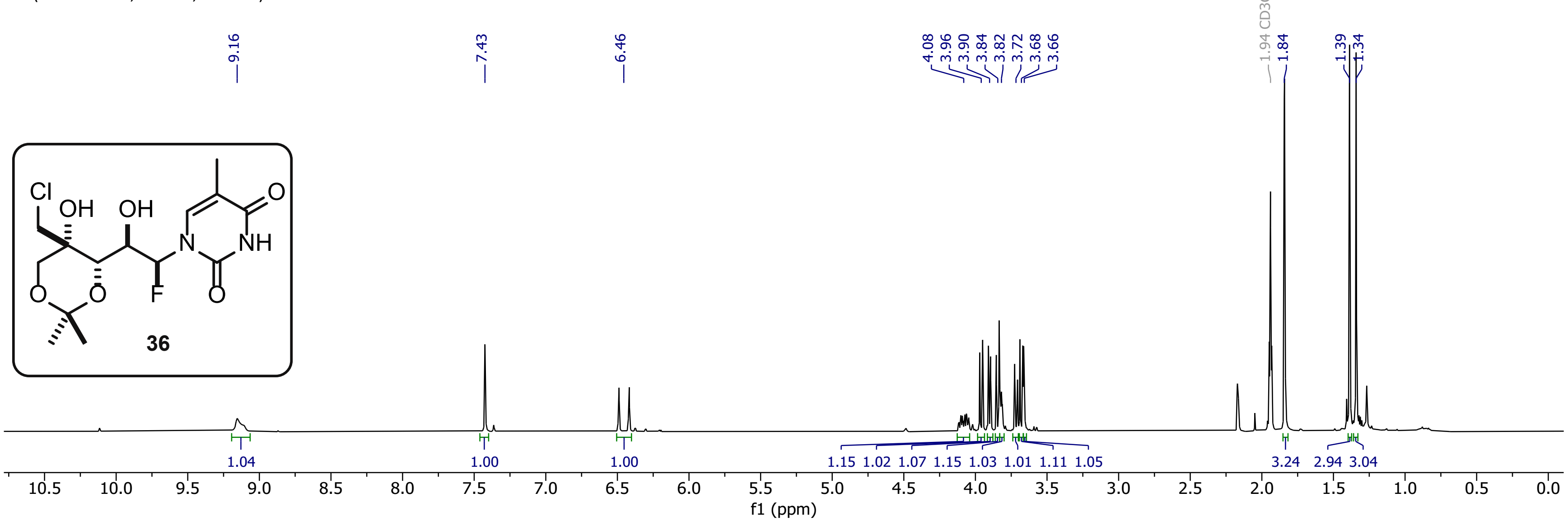


13C(150.92 MHz, CD3CN, 298.0 K)

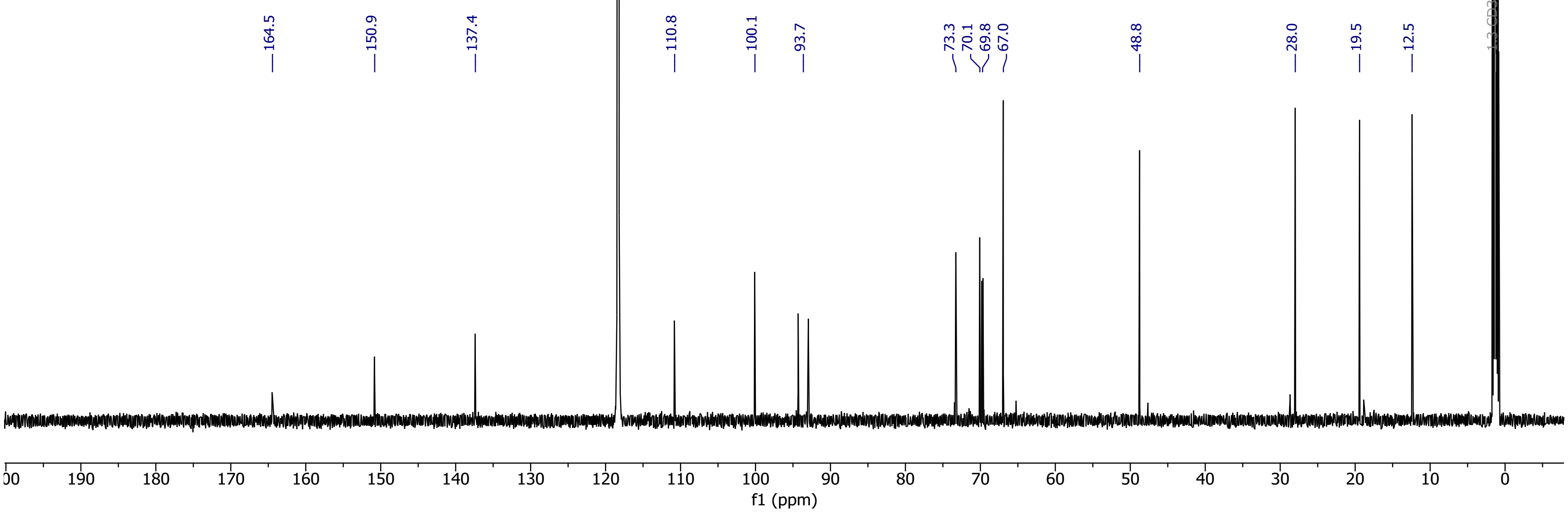

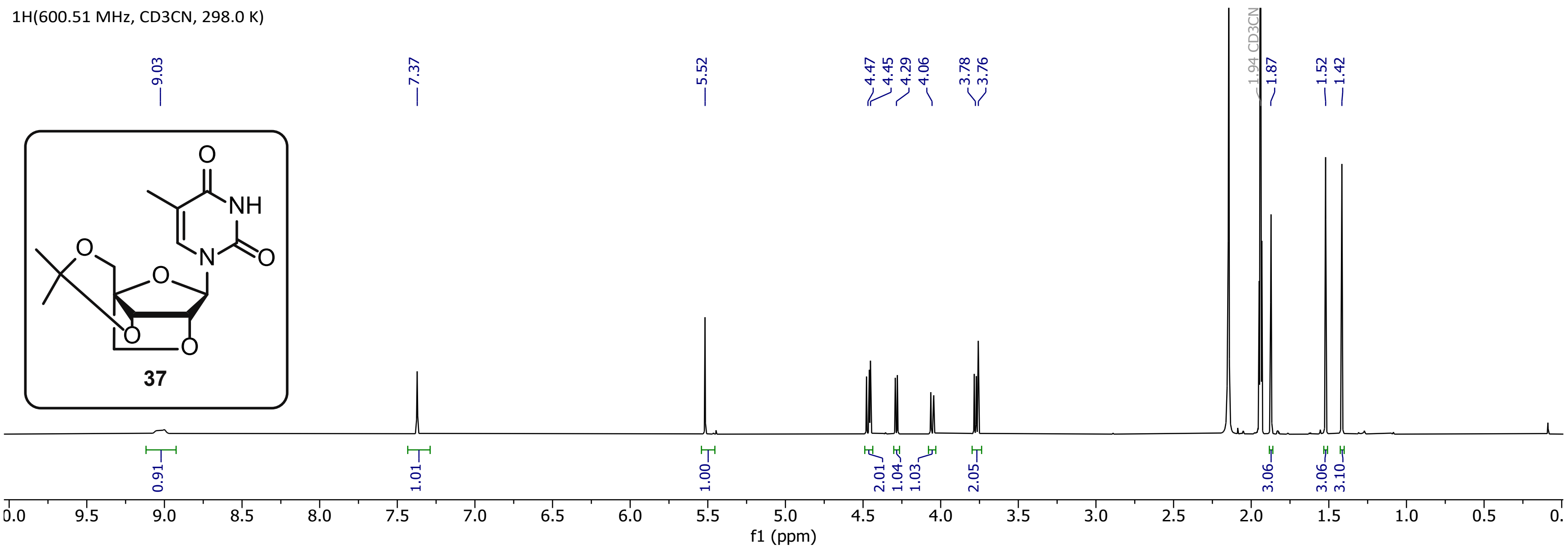
1H(600.51 MHz, CD3CN, 298.0 K)
9.03
7.37
5.52
4.47
4.45
4.29
4.06
3.78
3.76
1.94 CD3CN
1.87
1.52
1.42
0.91
1.01
1.00
2.01
1.04
1.03
2.05
3.06
3.06
3.10
O
NH
N
O
O
O
O
O
37
f1 (ppm)

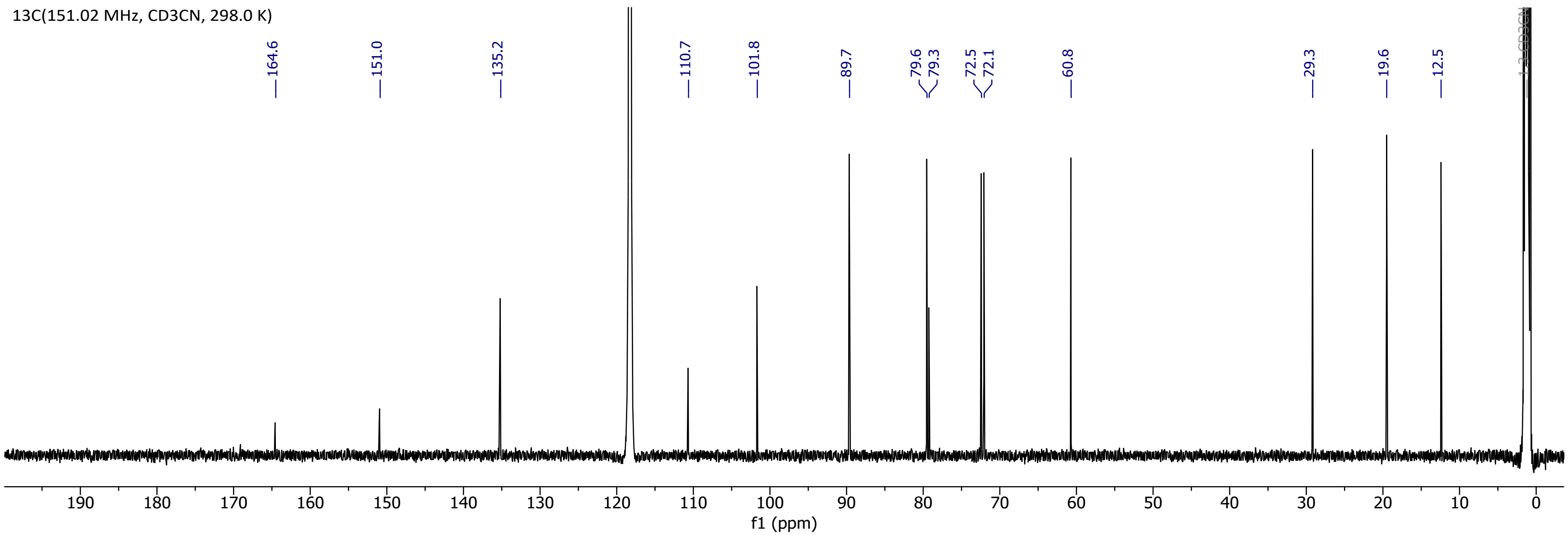
13C(151.02 MHz, CD3CN, 298.0 K)
164.6
151.0
135.2
110.7
101.8
89.7
79.6
79.3
72.5
72.1
60.8
29.3
19.6
12.5
f1 (ppm)

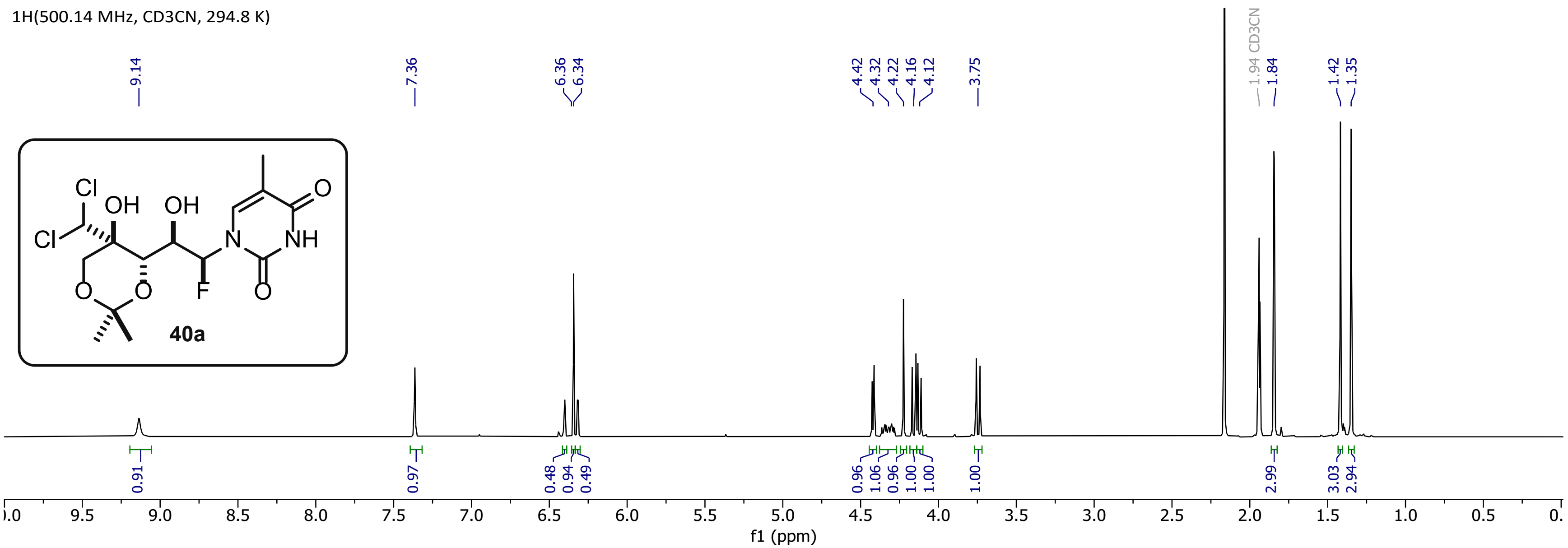
1H(500.14 MHz, CD3CN, 294.8 K)
9.14
7.36
6.36
6.34
4.42
4.32
4.22
4.16
4.12
3.75
1.94 CD3CN
1.84
1.42
1.35
0.91
0.97
0.48
0.94
0.49
0.96
1.06
0.96
1.00
1.00
1.00
2.99
3.03
2.94
40a
f1 (ppm)

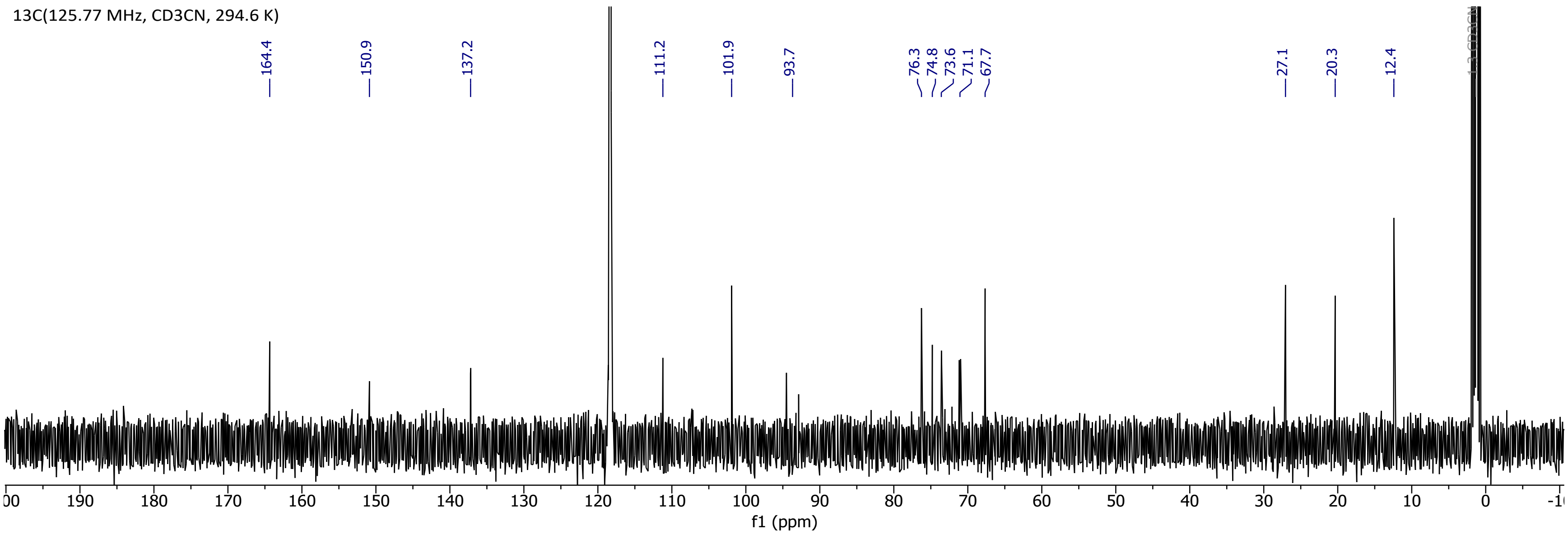
13C(125.77 MHz, CD3CN, 294.6 K)
164.4
150.9
137.2
111.2
101.9
93.7
76.3
74.8
73.6
71.1
67.7
27.1
20.3
12.4
f1 (ppm)

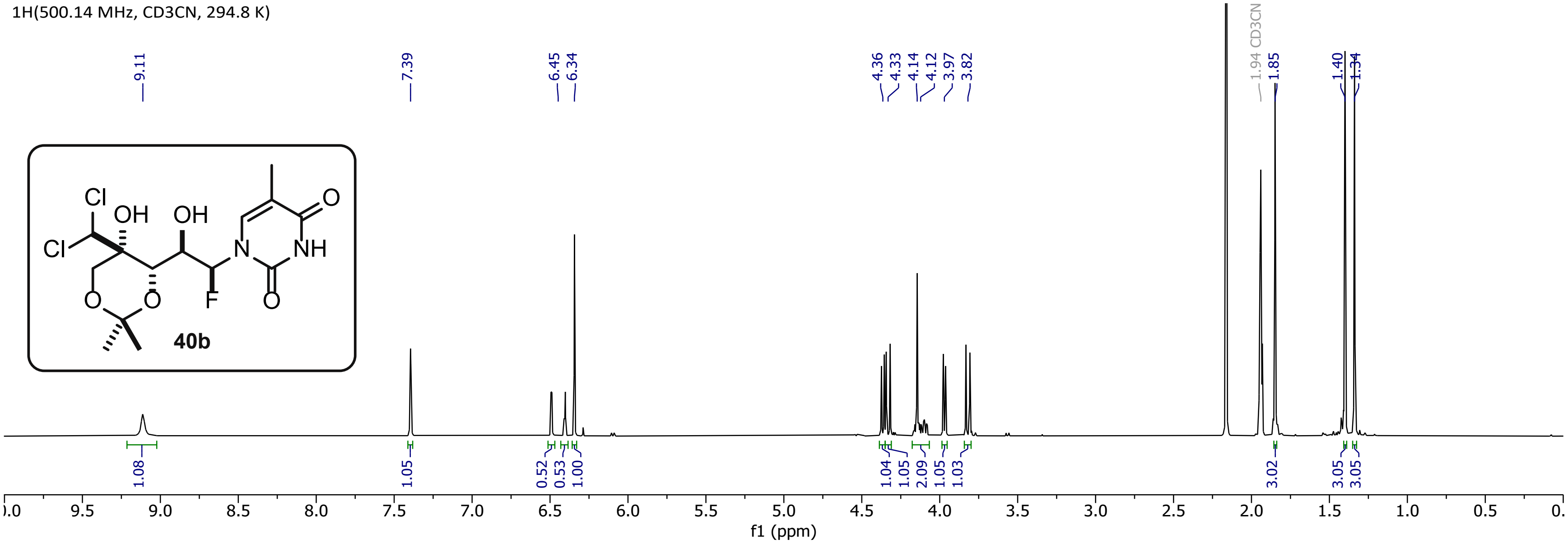
1H(500.14 MHz, CD3CN, 294.8 K)
9.11
7.39
6.45
6.34
4.36
4.33
4.14
4.12
3.97
3.82
1.94 CD3CN
1.85
1.40
1.34
1.08
1.05
0.52
0.53
1.00
1.04
1.05
2.09
1.05
1.03
3.02
3.05
3.05
40b
f1 (ppm)

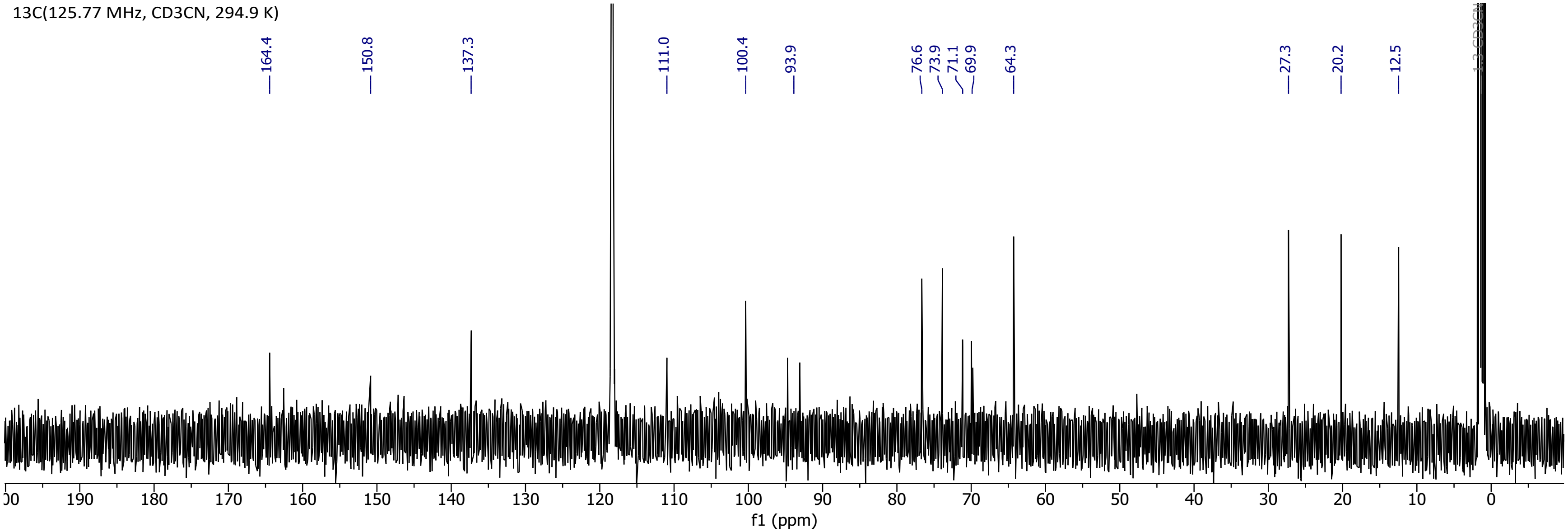
13C(125.77 MHz, CD3CN, 294.9 K)
164.4
150.8
137.3
111.0
100.4
93.9
76.6
73.9
71.1
69.9
64.3
27.3
20.2
12.5
f1 (ppm)

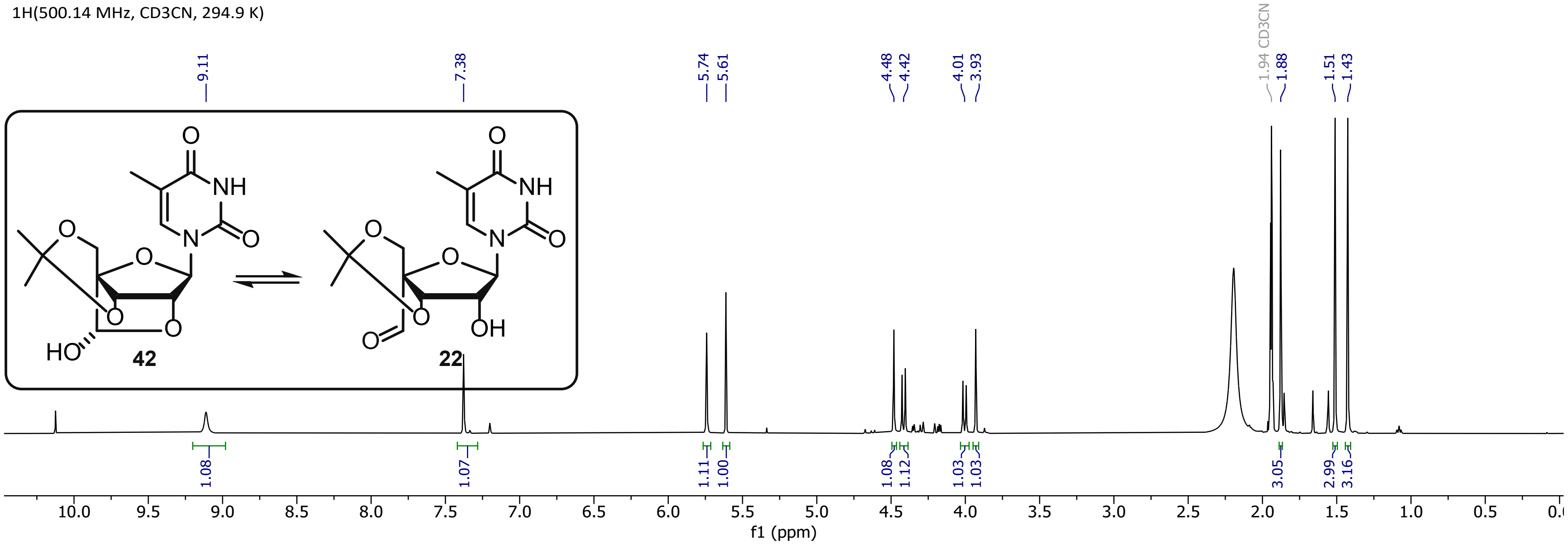
1H(500.14 MHz, CD3CN, 294.9 K)
9.11
7.38
5.74
5.61
4.48
4.42
4.01
3.93
1.94 CD3CN
1.88
1.51
1.43
42
22
1.08
1.07
1.11
1.00
1.08
1.12
1.03
1.03
3.05
2.99
3.16
f1 (ppm)

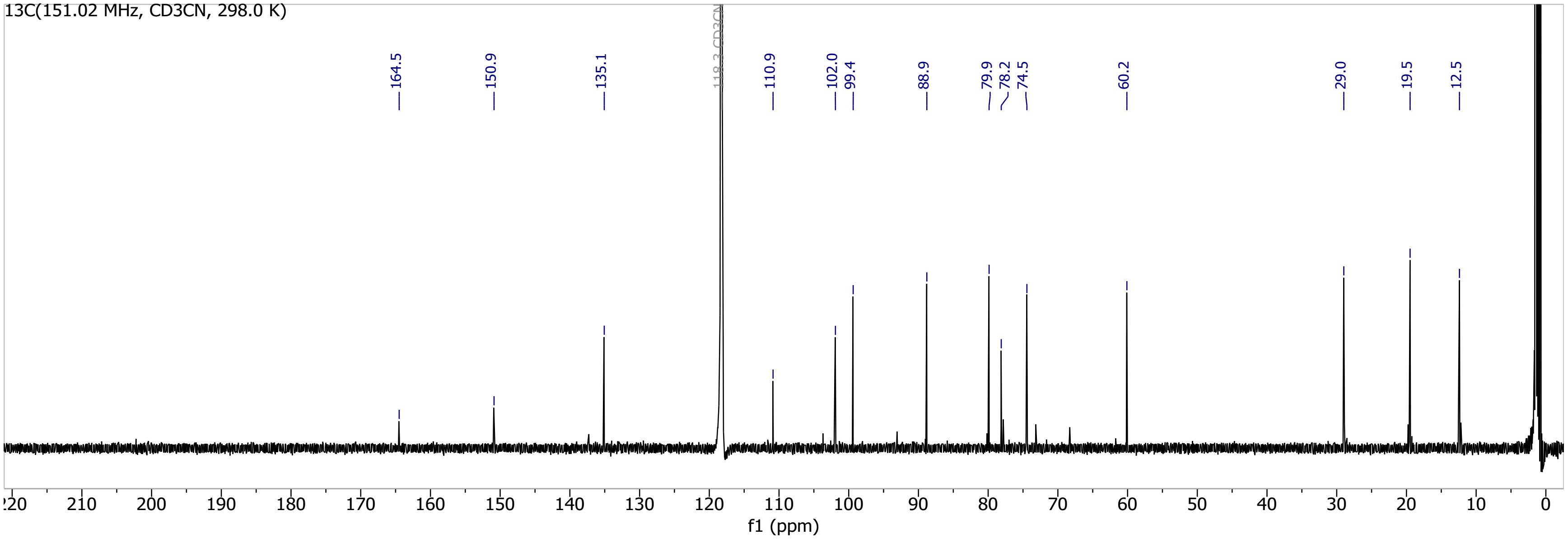
13C(151.02 MHz, CD3CN, 298.0 K)
164.5
150.9
135.1
110.9
102.0
99.4
88.9
79.9
78.2
74.5
60.2
29.0
19.5
12.5
f1 (ppm)

1H(500.14 MHz, CD3CN, 294.6 K)

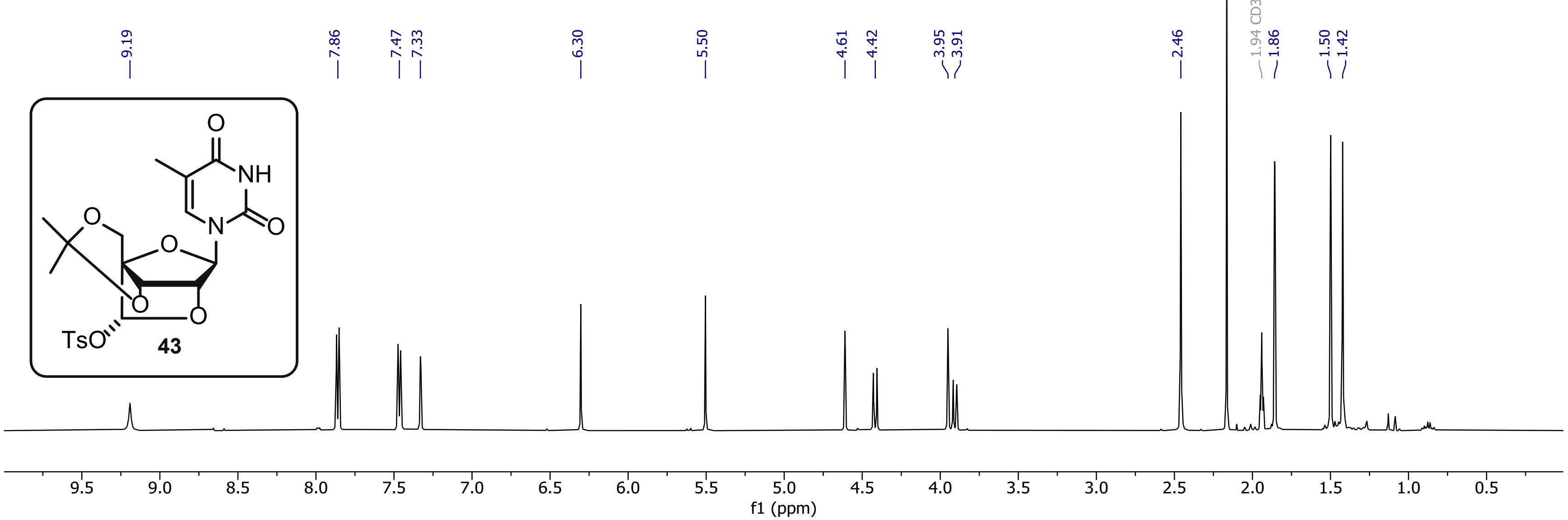


13C(125.77 MHz, CD3CN, 295.1 K)

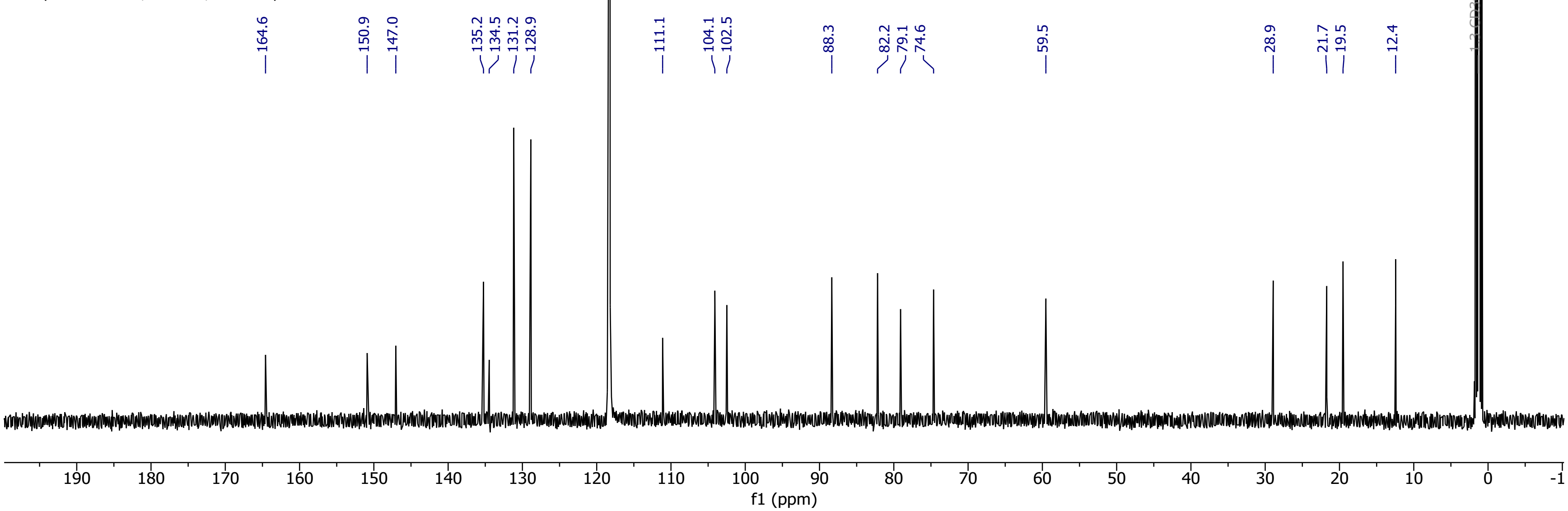

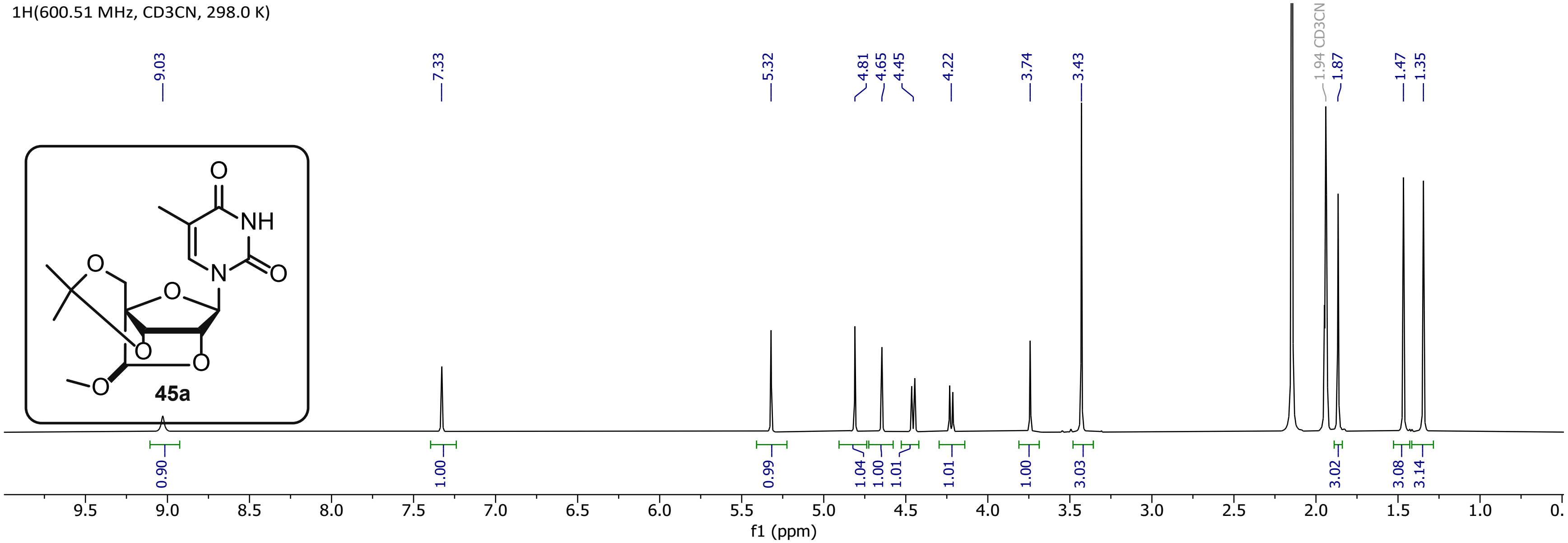
1H(600.51 MHz, CD3CN, 298.0 K)
9.03
7.33
5.32
4.81
4.65
4.45
4.22
3.74
3.43
1.94 CD3CN
1.87
1.47
1.35
O
NH
O
N
O
O
O
O
O
O
45a
0.90
1.00
0.99
1.04
1.00
1.01
1.01
1.00
3.03
3.02
3.08
3.14
f1 (ppm)

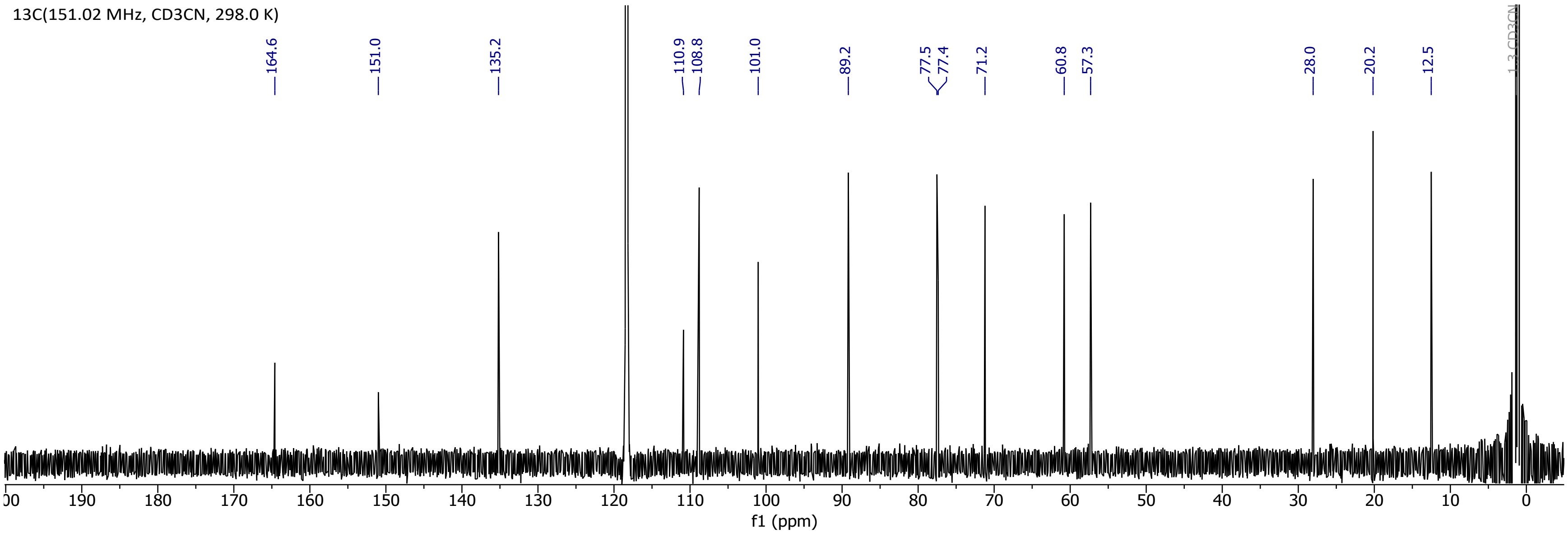
13C(151.02 MHz, CD3CN, 298.0 K)
164.6
151.0
135.2
110.9
108.8
101.0
89.2
77.5
77.4
71.2
60.8
57.3
28.0
20.2
12.5
1.3 CD3CN
f1 (ppm)

1H(600.51 MHz, CD3CN, 286.9 K)

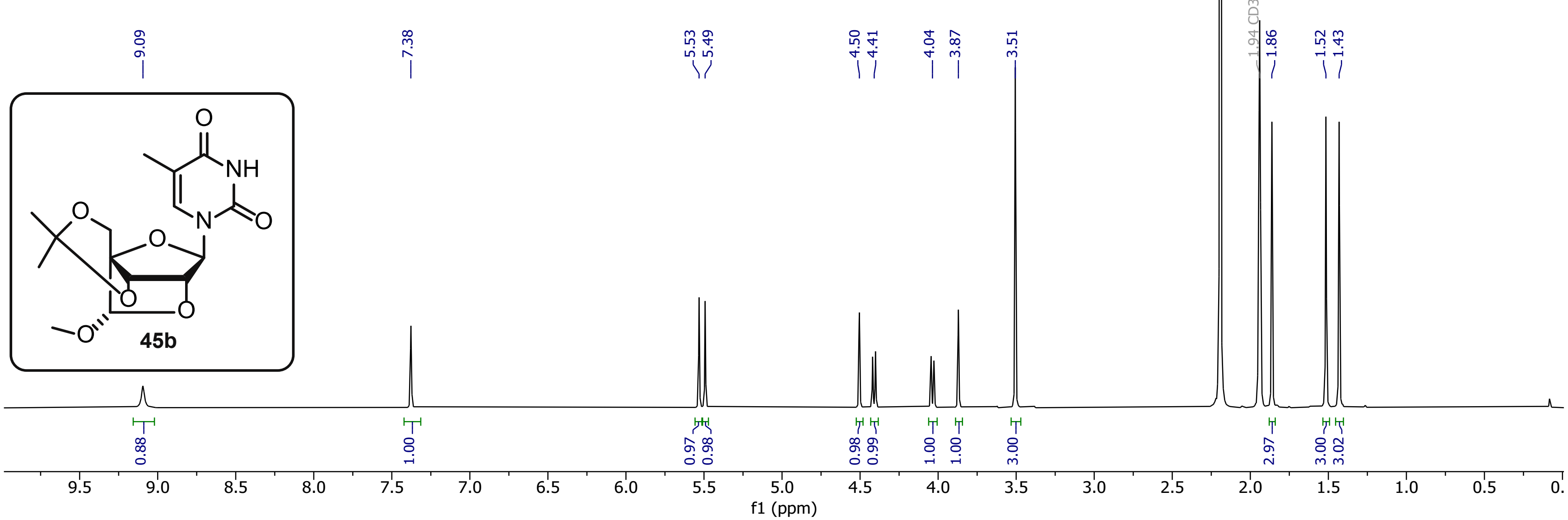


13C(151.02 MHz, CD3CN, 287.0 K)

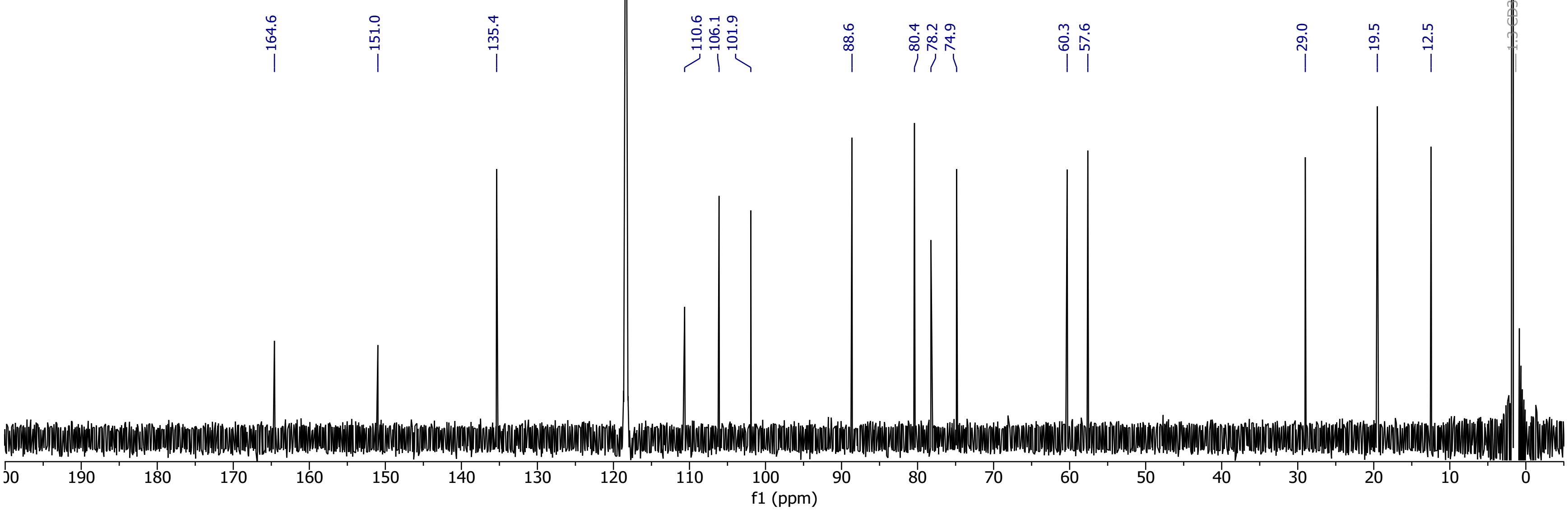

1H(500.14 MHz, CD3CN, 294.6 K)

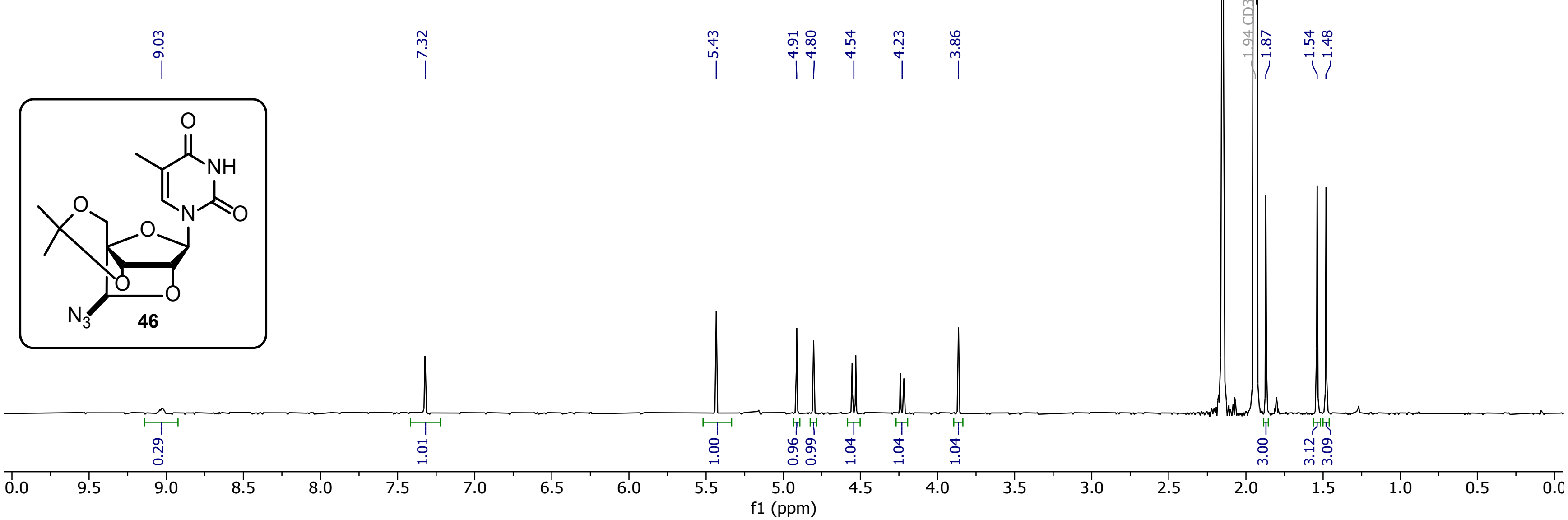


13C(150.92 MHz, CDCl3, 298.0 K)

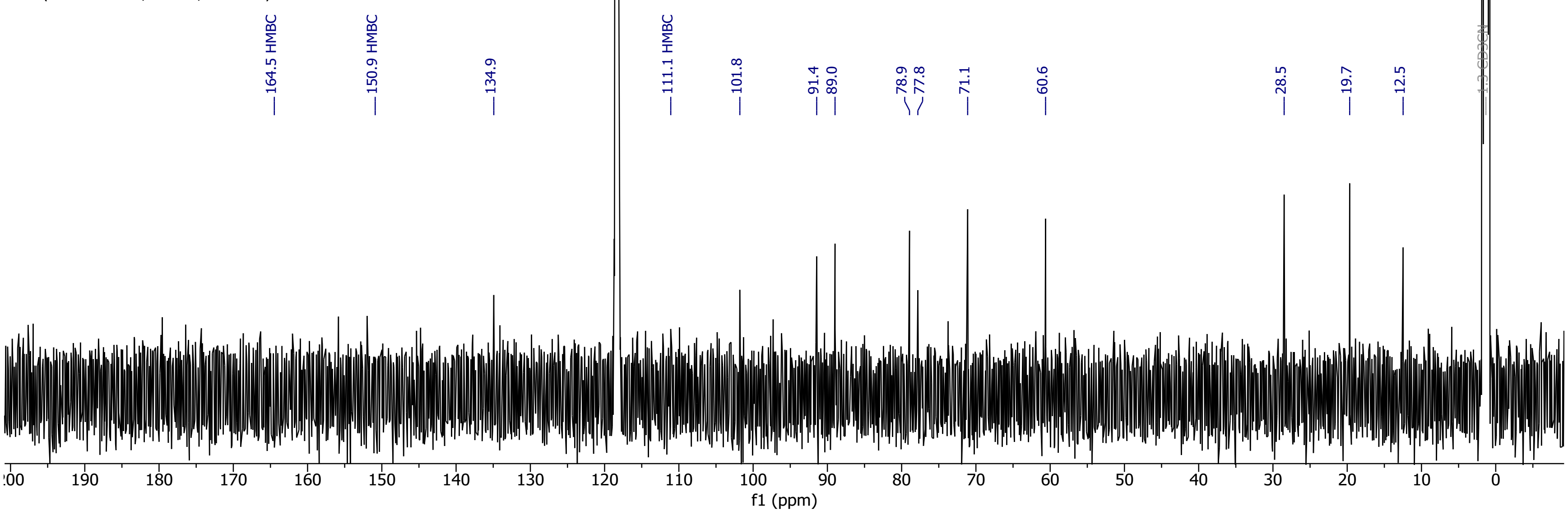

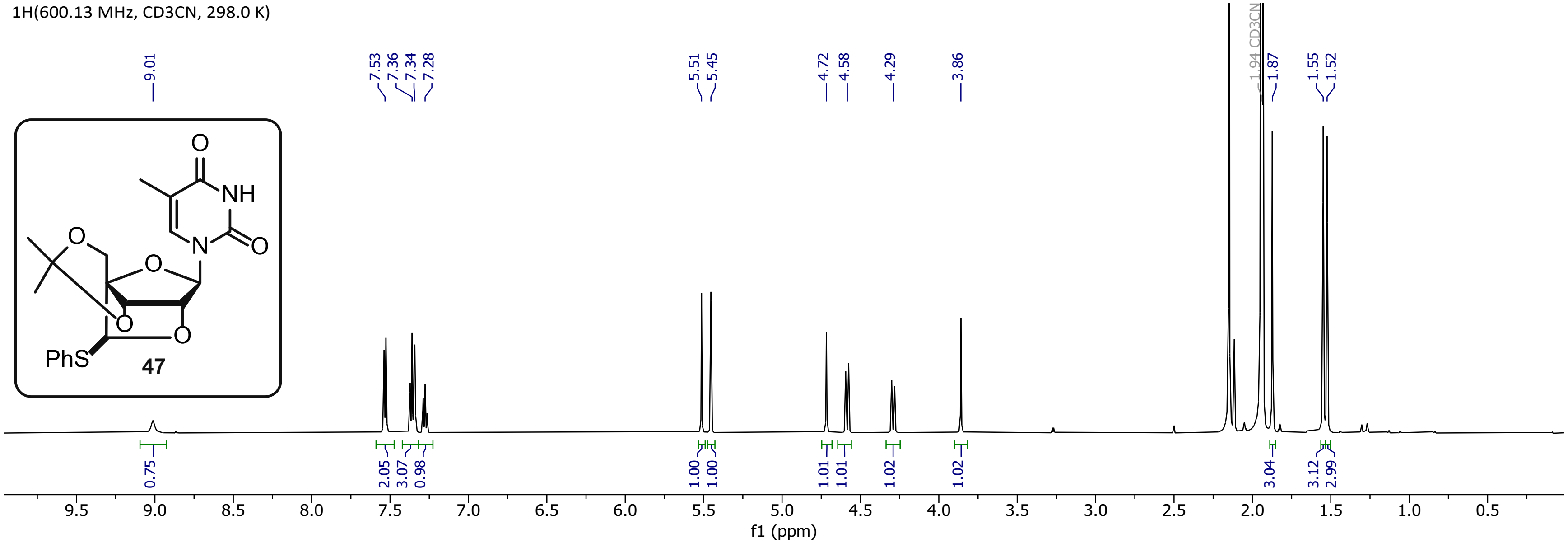
1H(600.13 MHz, CD3CN, 298.0 K)
9.01
7.53
7.36
7.34
7.28
5.51
5.45
4.72
4.58
4.29
3.86
1.94 CD3CN
1.87
1.55
1.52
0.75
2.05
3.07
0.98
1.00
1.00
1.01
1.01
1.02
1.02
3.04
3.12
2.99
O
NH
O
N
O
O
O
O
PhS
47
f1 (ppm)

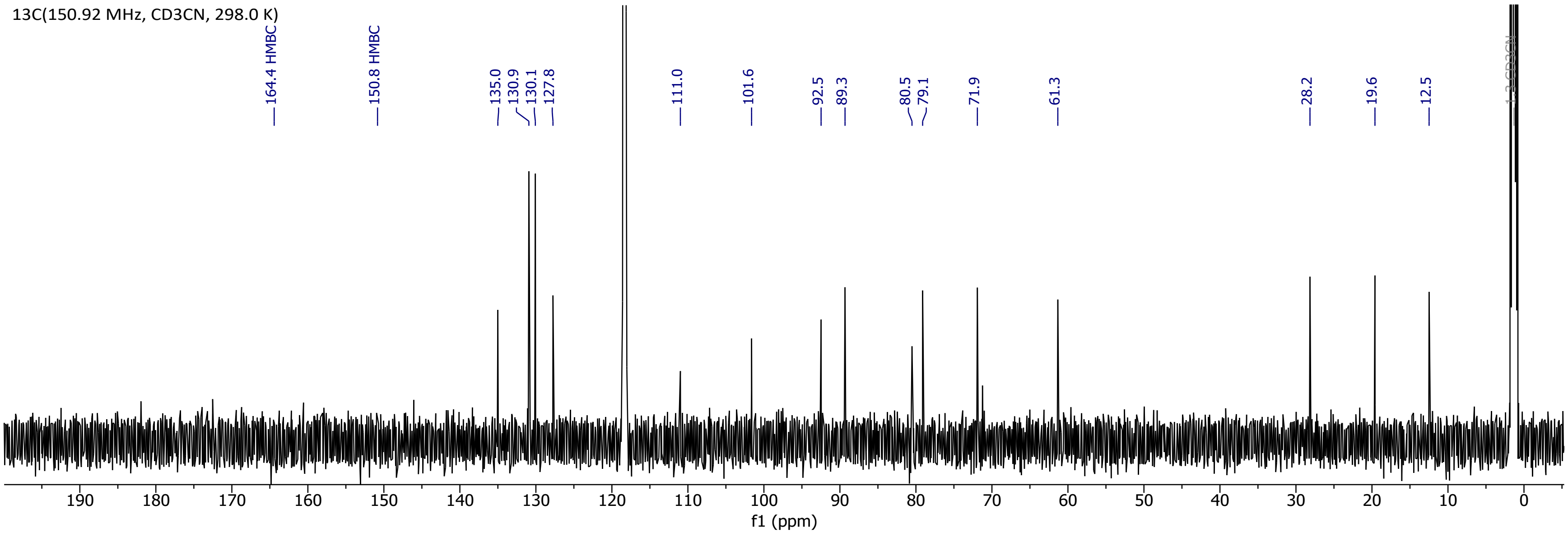
13C(150.92 MHz, CD3CN, 298.0 K)
164.4 HMBC
150.8 HMBC
135.0
130.9
130.1
127.8
111.0
101.6
92.5
89.3
80.5
79.1
71.9
61.3
28.2
19.6
12.5
1.3 CD3CN
f1 (ppm)

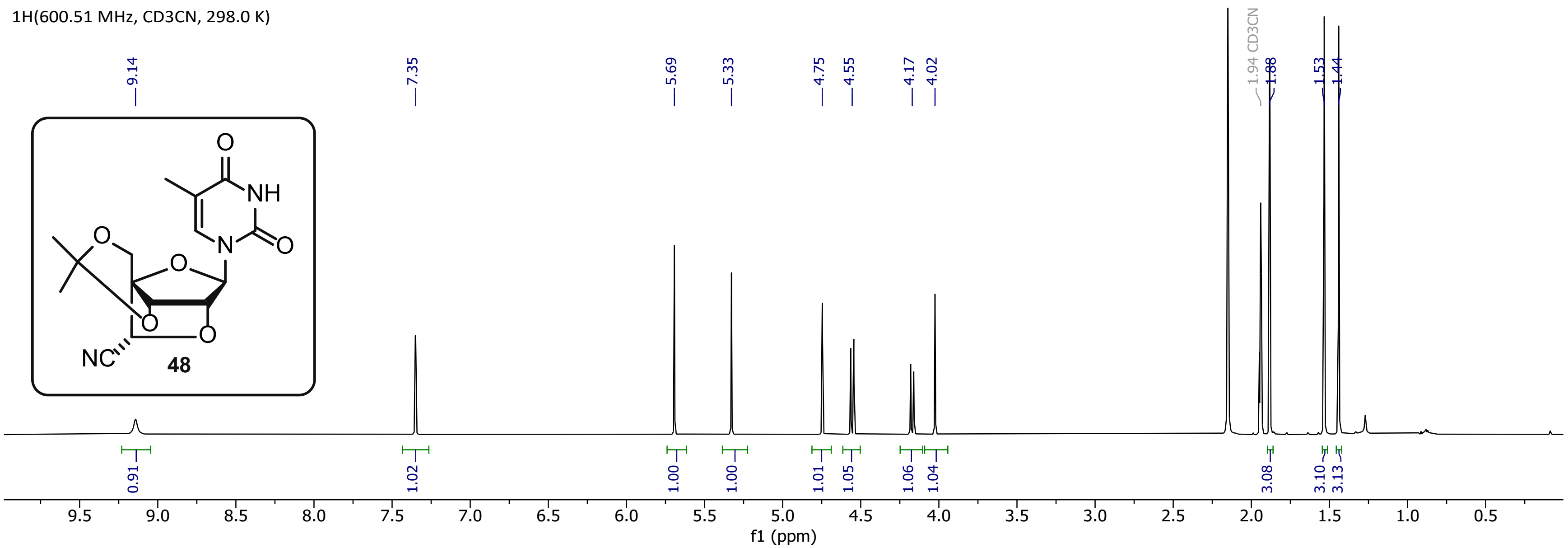
1H(600.51 MHz, CD3CN, 298.0 K)
9.14
7.35
5.69
5.33
4.75
4.55
4.17
4.02
1.94 CD3CN
1.88
1.53
1.44
NH
O
N
NC
48
0.91
1.02
1.00
1.00
1.01
1.05
1.06
1.04
3.08
3.10
3.13
9.5 9.0 8.5 8.0 7.5 7.0 6.5 6.0 5.5 5.0 4.5 4.0 3.5 3.0 2.5 2.0 1.5 1.0 0.5
f1 (ppm)

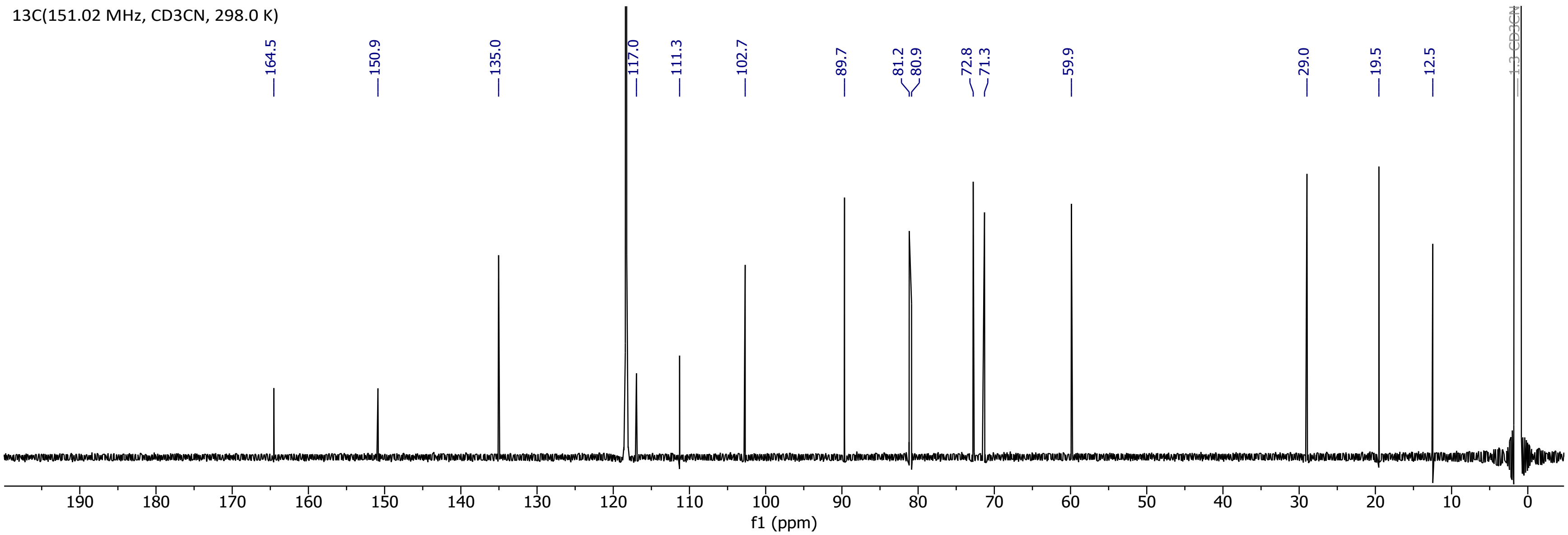
13C(151.02 MHz, CD3CN, 298.0 K)
164.5
150.9
135.0
117.0
111.3
102.7
89.7
81.2
80.9
72.8
71.3
59.9
29.0
19.5
12.5
1.3 CD3CN
190 180 170 160 150 140 130 120 110 100 90 80 70 60 50 40 30 20 10 0
f1 (ppm)

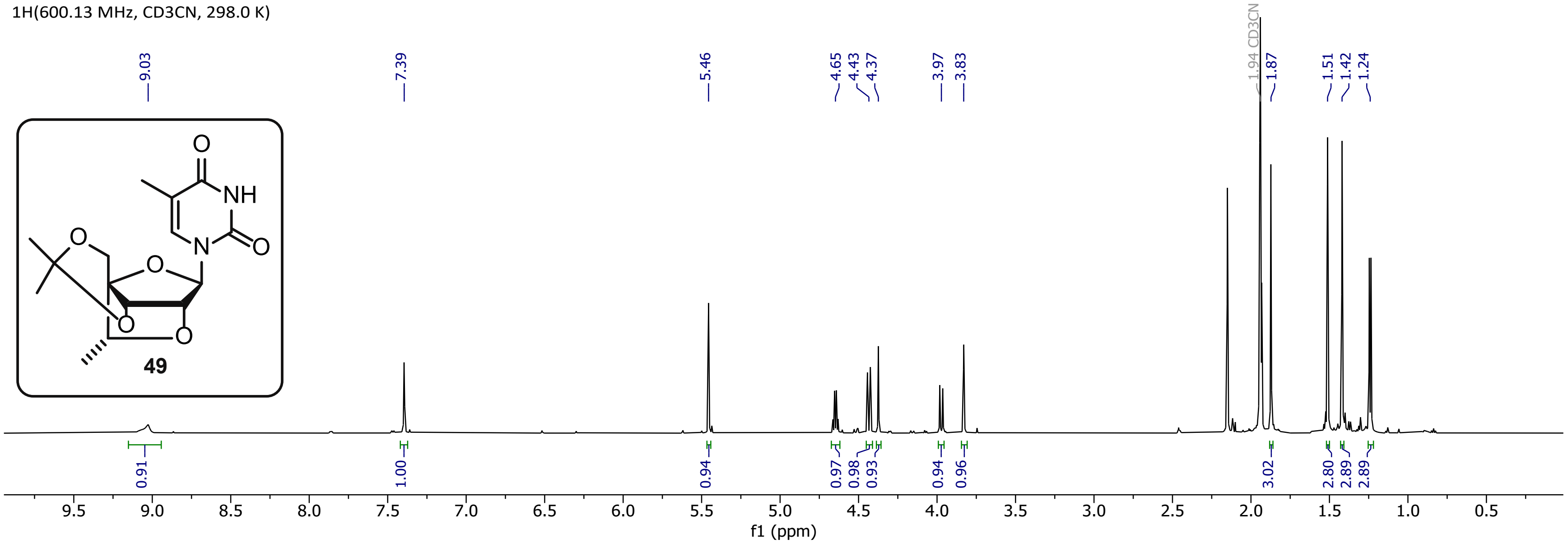
1H(600.13 MHz, CD3CN, 298.0 K)
9.03
7.39
5.46
4.65
4.43
4.37
3.97
3.83
1.94 CD3CN
1.87
1.51
1.42
1.24
O
NH
O
N
O
O
O
O
49
0.91
1.00
0.94
0.97
0.98
0.93
0.94
0.96
3.02
2.80
2.89
2.89
9.5 9.0 8.5 8.0 7.5 7.0 6.5 6.0 5.5 5.0 4.5 4.0 3.5 3.0 2.5 2.0 1.5 1.0 0.5
f1 (ppm)

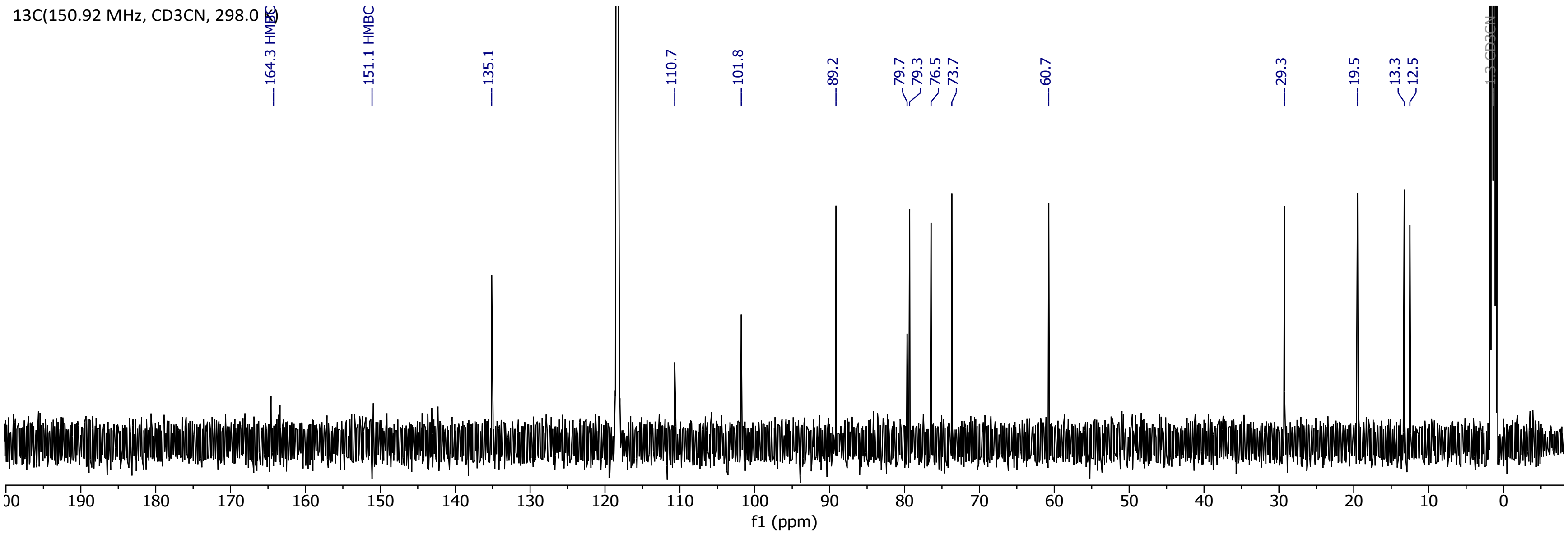
13C(150.92 MHz, CD3CN, 298.0 K)
164.3 HMBC
151.1 HMBC
135.1
110.7
101.8
89.2
79.7
79.3
76.5
73.7
60.7
29.3
19.5
13.3
12.5
190 180 170 160 150 140 130 120 110 100 90 80 70 60 50 40 30 20 10 0
f1 (ppm)

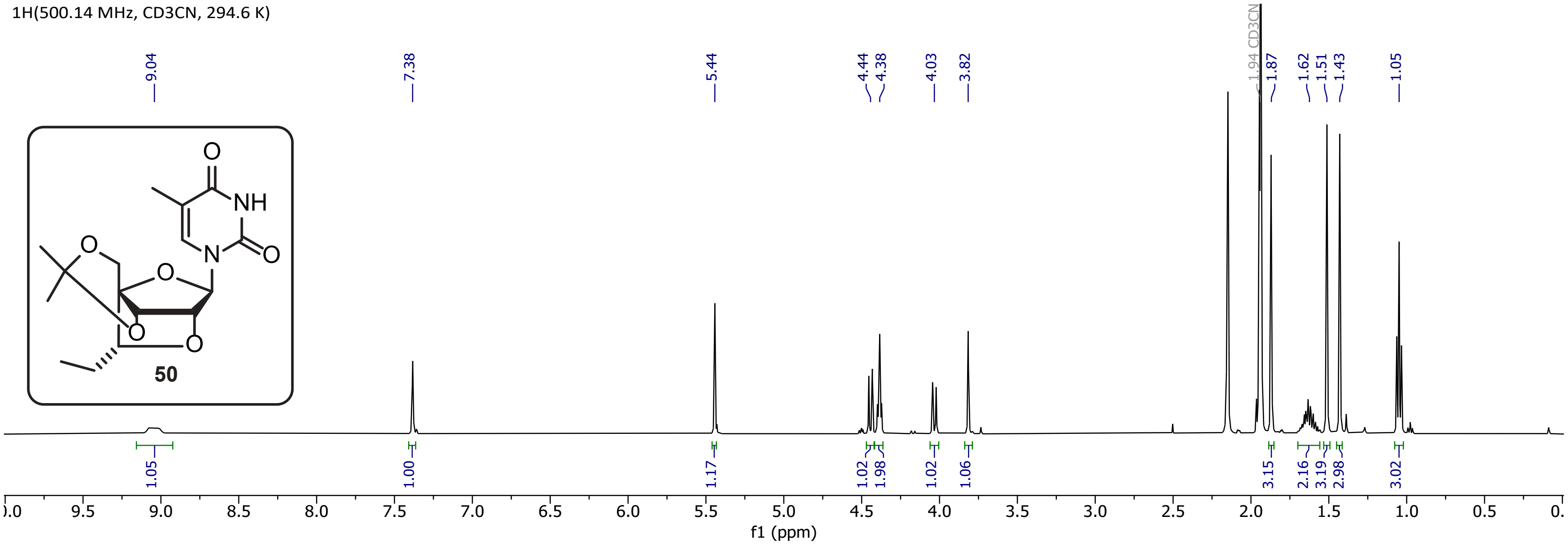
1H(500.14 MHz, CD3CN, 294.6 K)
9.04
7.38
5.44
4.44
4.38
4.03
3.82
1.94 CD3CN
1.87
1.62
1.51
1.43
1.05
1.05
1.00
1.17
1.02
1.98
1.02
1.06
3.15
2.16
3.19
2.98
3.02
O
NH
N
O
O
O
O
O
50
f1 (ppm)

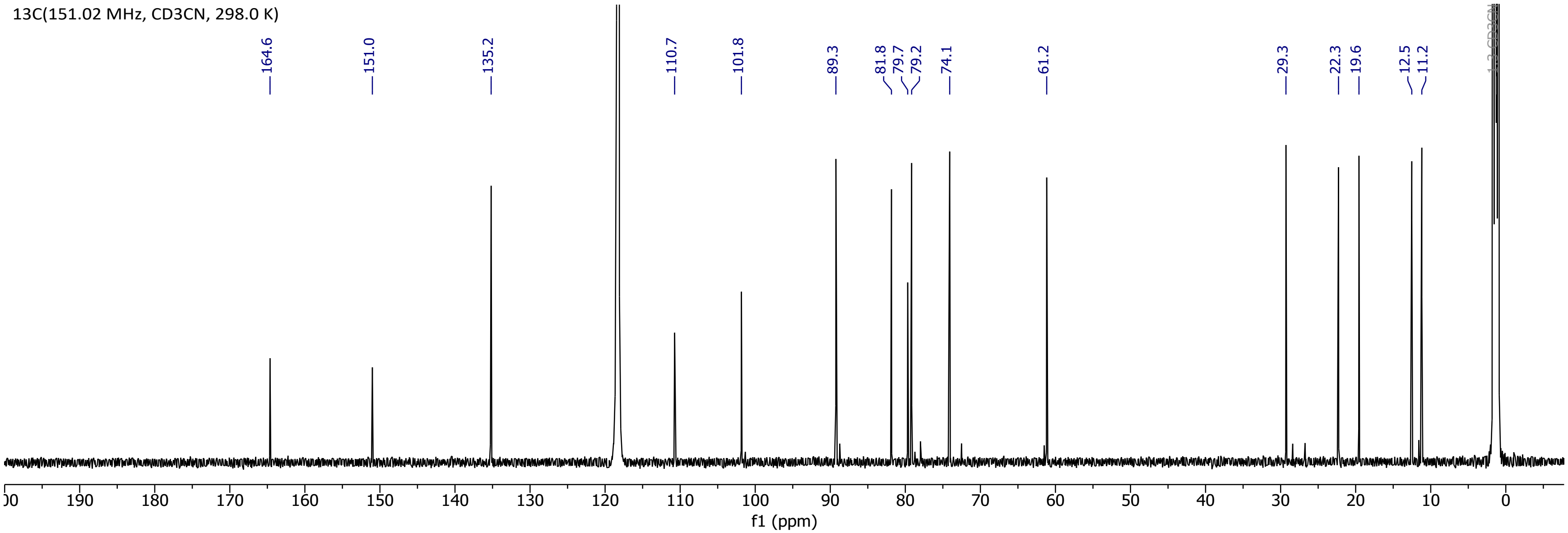
13C(151.02 MHz, CD3CN, 298.0 K)
164.6
151.0
135.2
110.7
101.8
89.3
81.8
79.7
79.2
74.1
61.2
29.3
22.3
19.6
12.5
11.2
f1 (ppm)

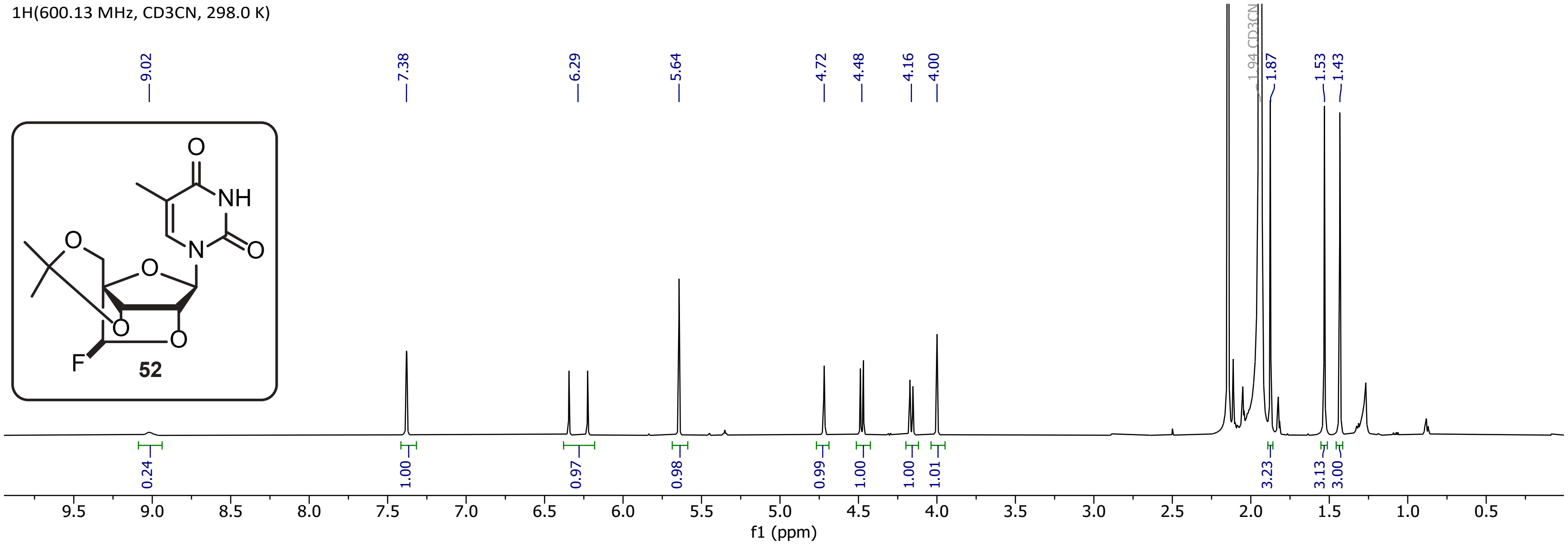
1H(600.13 MHz, CD3CN, 298.0 K)
9.02
7.38
6.29
5.64
4.72
4.48
4.16
4.00
1.94 CD3CN
1.87
1.53
1.43
NH
52
0.24
1.00
0.97
0.98
0.99
1.00
1.00
1.01
3.23
3.13
3.00
f1 (ppm)

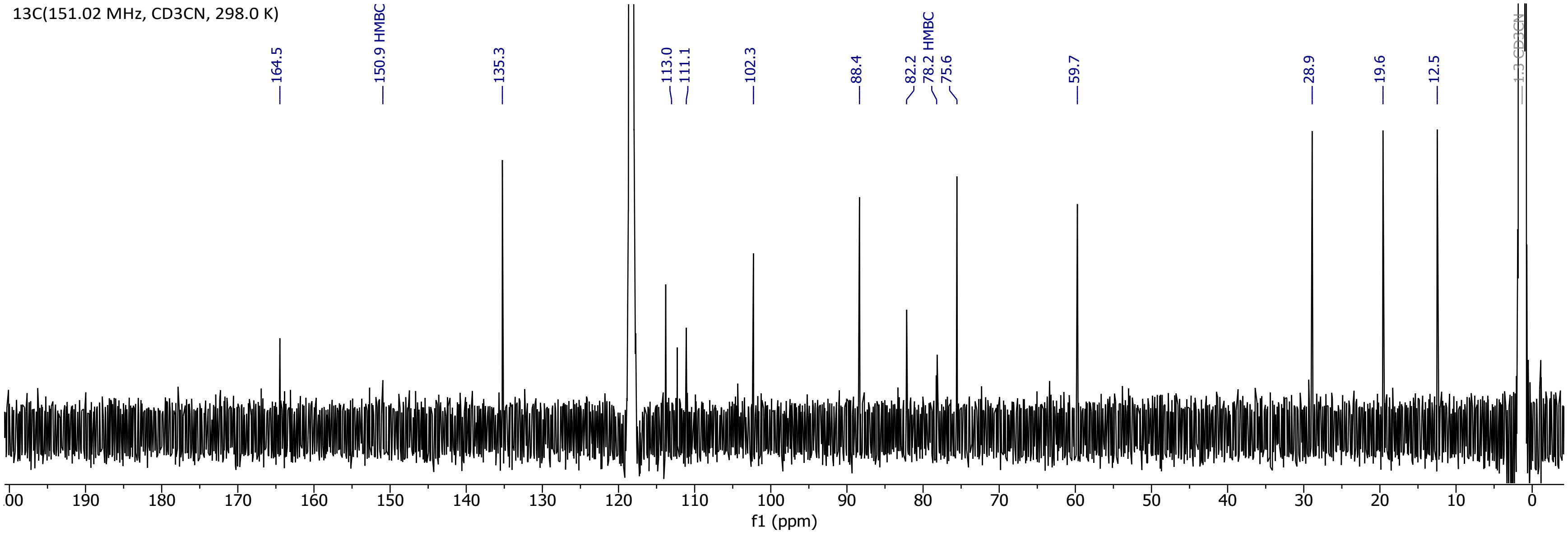
13C(151.02 MHz, CD3CN, 298.0 K)
164.5
150.9 HMBC
135.3
113.0
111.1
102.3
88.4
82.2
78.2 HMBC
75.6
59.7
28.9
19.6
12.5
1.3 CD3CN
f1 (ppm)

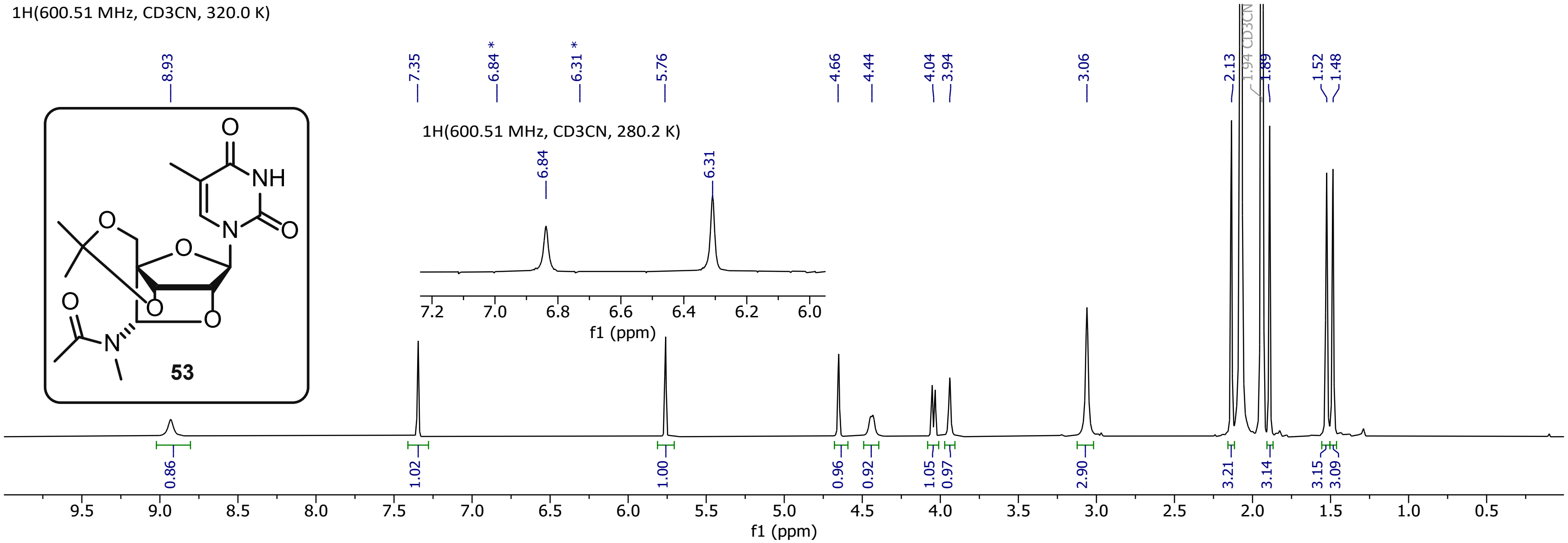
1H(600.51 MHz, CD3CN, 320.0 K)
8.93
7.35
6.84 *
6.31 *
5.76
4.66
4.44
4.04
3.94
3.06
2.13
1.94 CD3CN
1.89
1.52
1.48
1H(600.51 MHz, CD3CN, 280.2 K)
6.84
6.31
f1 (ppm)
53
0.86
1.02
1.00
0.96
0.92
1.05
0.97
2.90
3.21
3.14
3.15
3.09
f1 (ppm)

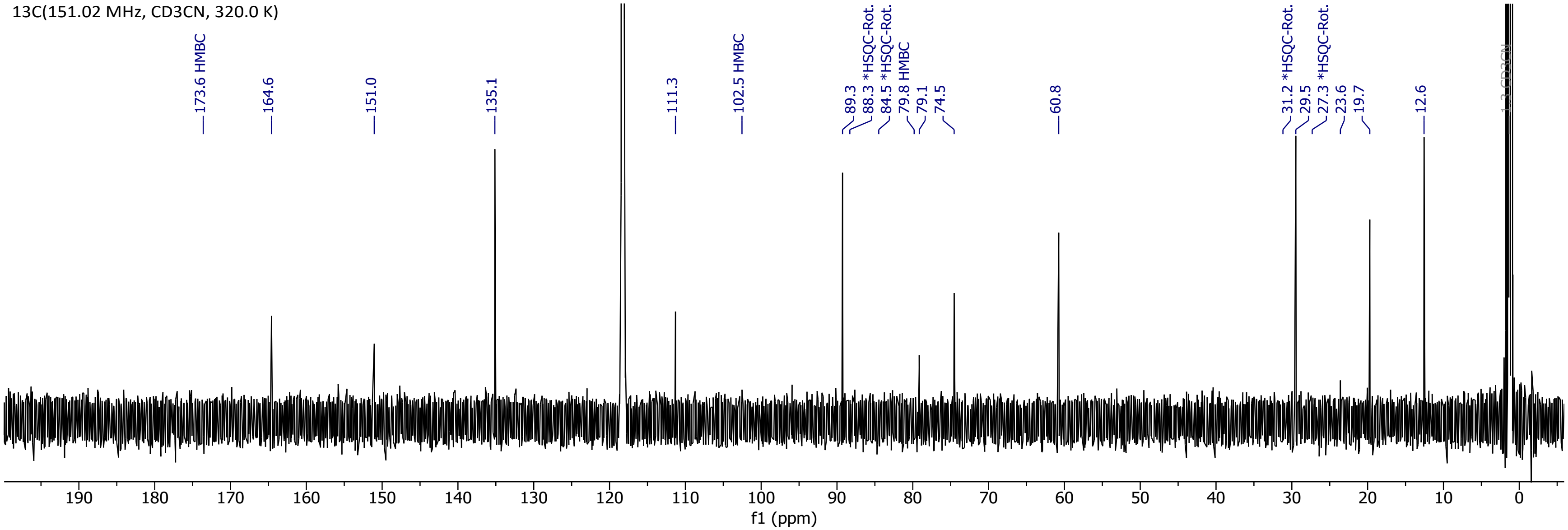
13C(151.02 MHz, CD3CN, 320.0 K)
173.6 HMBC
164.6
151.0
135.1
111.3
102.5 HMBC
89.3
88.3 *HSQC-Rot.
84.5 *HSQC-Rot.
79.8 HMBC
79.1
74.5
60.8
31.2 *HSQC-Rot.
29.5
27.3 *HSQC-Rot.
23.6
19.7
12.6
1.3 CD3CN
f1 (ppm)

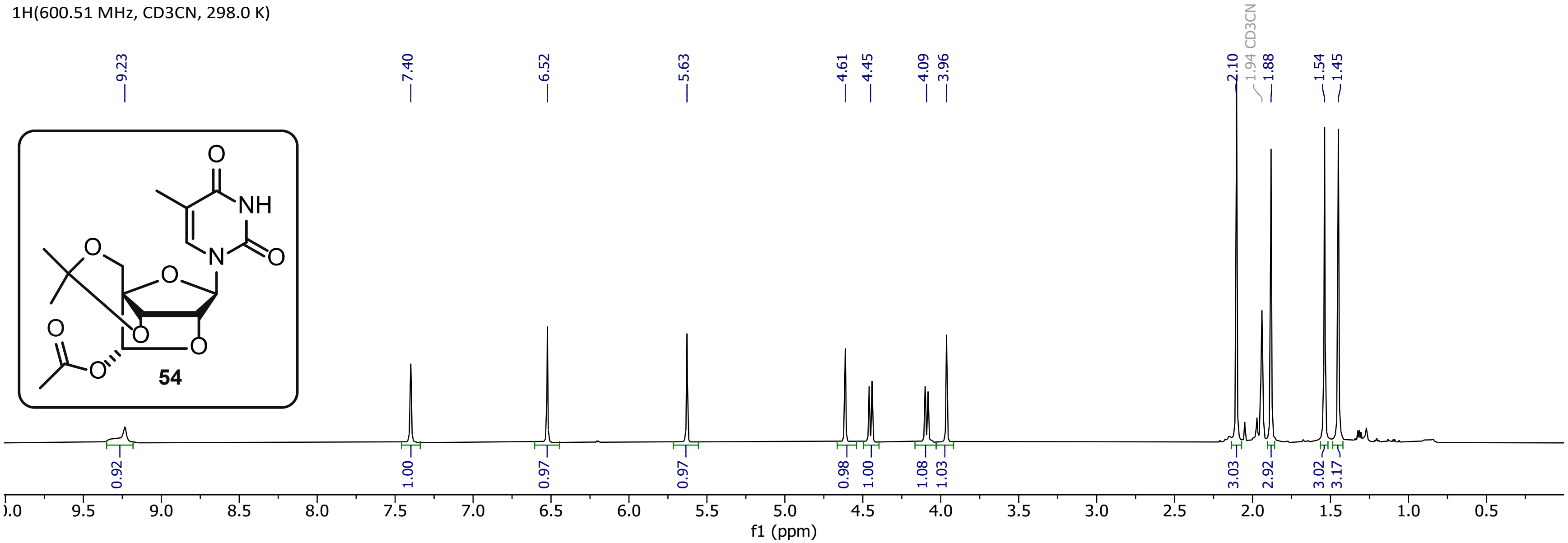
1H(600.51 MHz, CD3CN, 298.0 K)
9.23
7.40
6.52
5.63
4.61
4.45
4.09
3.96
2.10
1.94 CD3CN
1.88
1.54
1.45
NH
O
N
54
0.92
1.00
0.97
0.97
0.98
1.00
1.08
1.03
3.03
2.92
3.02
3.17
f1 (ppm)

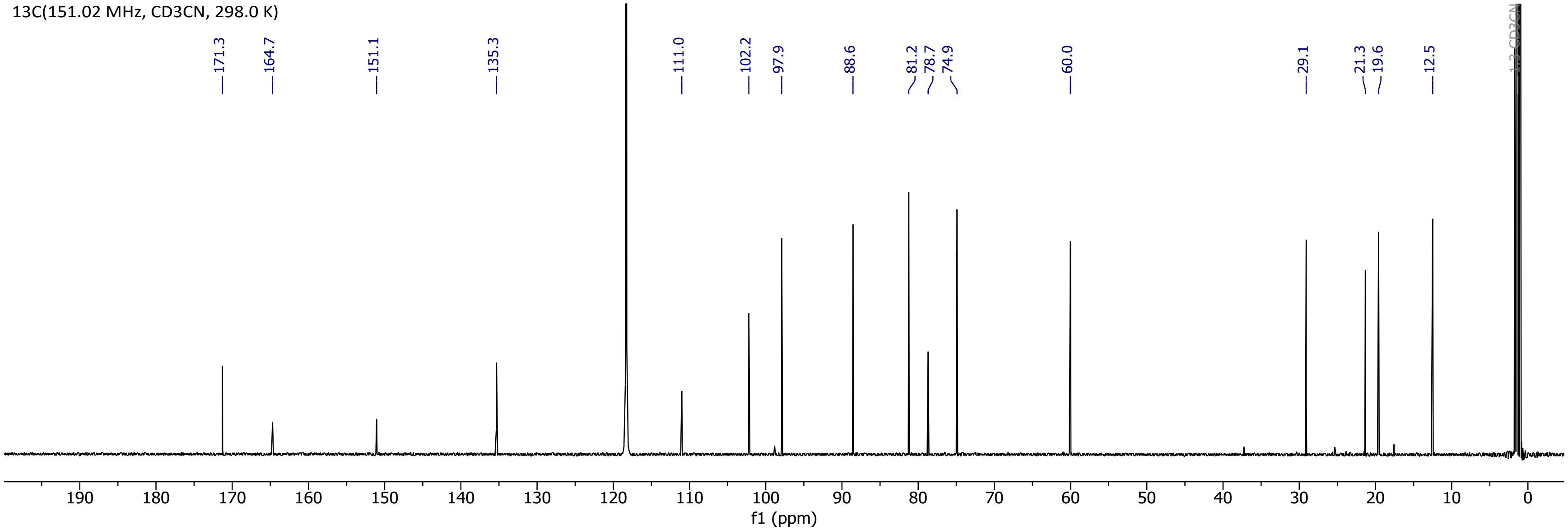
13C(151.02 MHz, CD3CN, 298.0 K)
171.3
164.7
151.1
135.3
111.0
102.2
97.9
88.6
81.2
78.7
74.9
60.0
29.1
21.3
19.6
12.5
f1 (ppm)

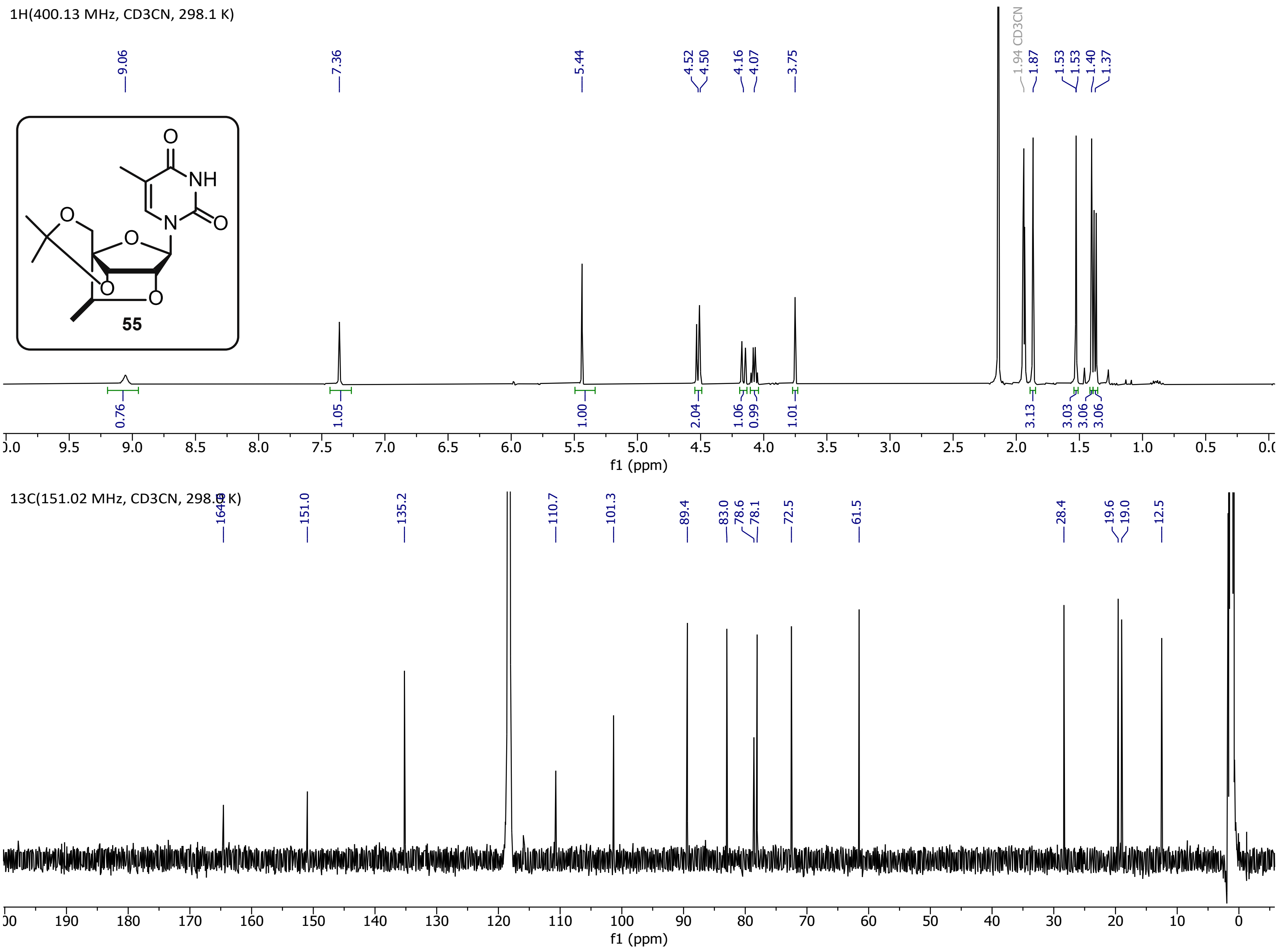
1H(400.13 MHz, CD3CN, 298.1 K)
9.06
7.36
5.44
4.52
4.50
4.16
4.07
3.75
1.94 CD3CN
1.87
1.53
1.53
1.40
1.37
0.76
1.05
1.00
2.04
1.06
0.99
1.01
3.13
3.03
3.06
3.06
f1 (ppm)
55
13C(151.02 MHz, CD3CN, 298.0 K)
164.0
151.0
135.2
110.7
101.3
89.4
83.0
78.6
78.1
72.5
61.5
28.4
19.6
19.0
12.5
f1 (ppm)

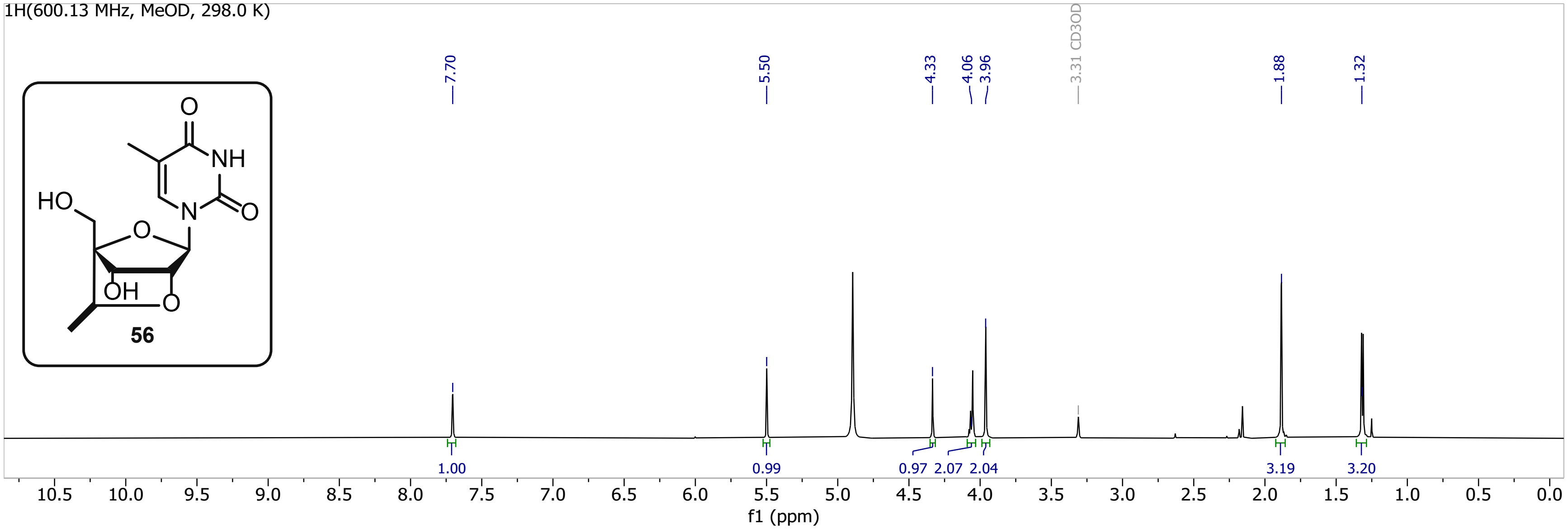
1H(600.13 MHz, MeOD, 298.0 K)
56
7.70
5.50
4.33
4.06
3.96
3.31 CD3OD
1.88
1.32
1.00
0.99
0.97
2.07
2.04
3.19
3.20
f1 (ppm)

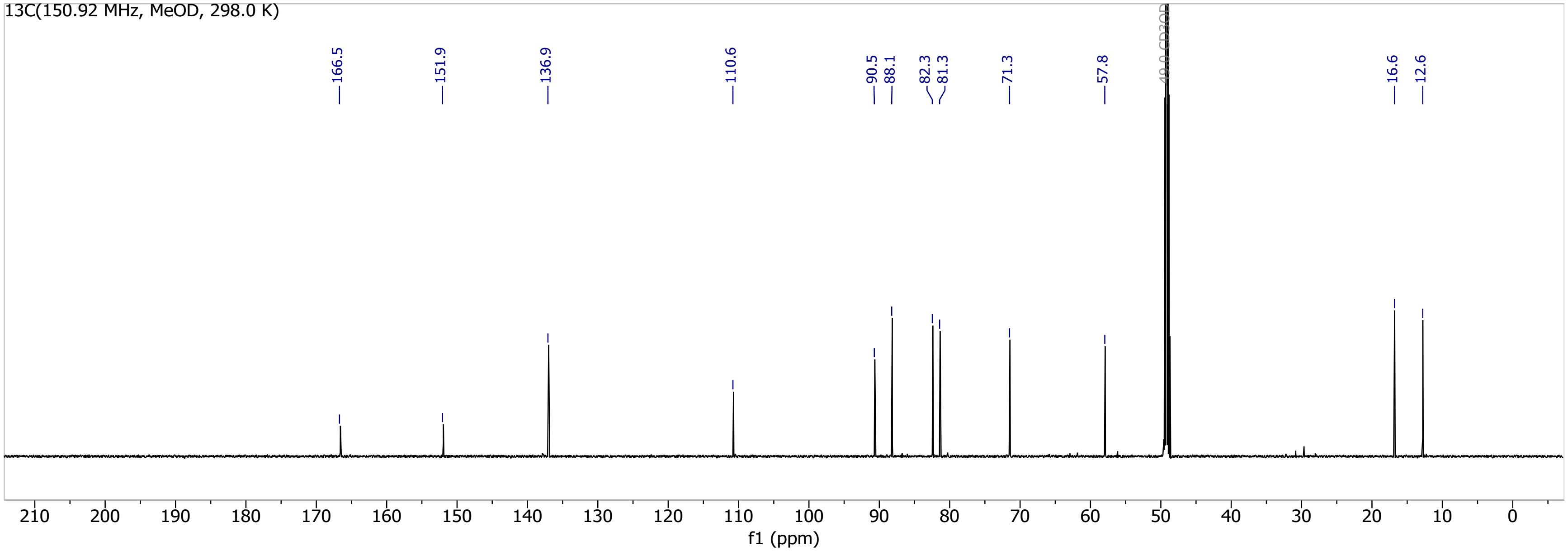
13C(150.92 MHz, MeOD, 298.0 K)
166.5
151.9
136.9
110.6
90.5
88.1
82.3
81.3
71.3
57.8
16.6
12.6
f1 (ppm)

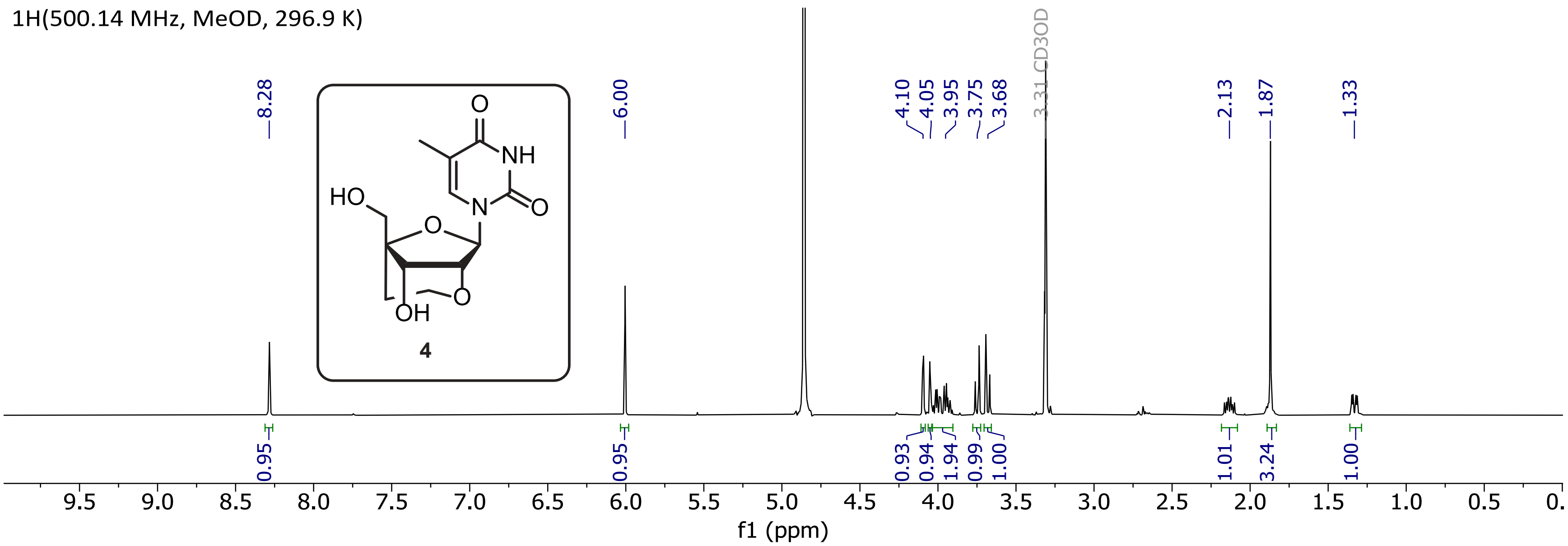
1H(500.14 MHz, MeOD, 296.9 K)
8.28
6.00
4.10
4.05
3.95
3.75
3.68
3.31 CD3OD
2.13
1.87
1.33
0.95
0.95
0.93
0.94
1.94
0.99
1.00
1.01
3.24
1.00
O
NH
HO
N
O
O
O
OH
4
9.5 9.0 8.5 8.0 7.5 7.0 6.5 6.0 5.5 5.0 4.5 4.0 3.5 3.0 2.5 2.0 1.5 1.0 0.5 0.
f1 (ppm)

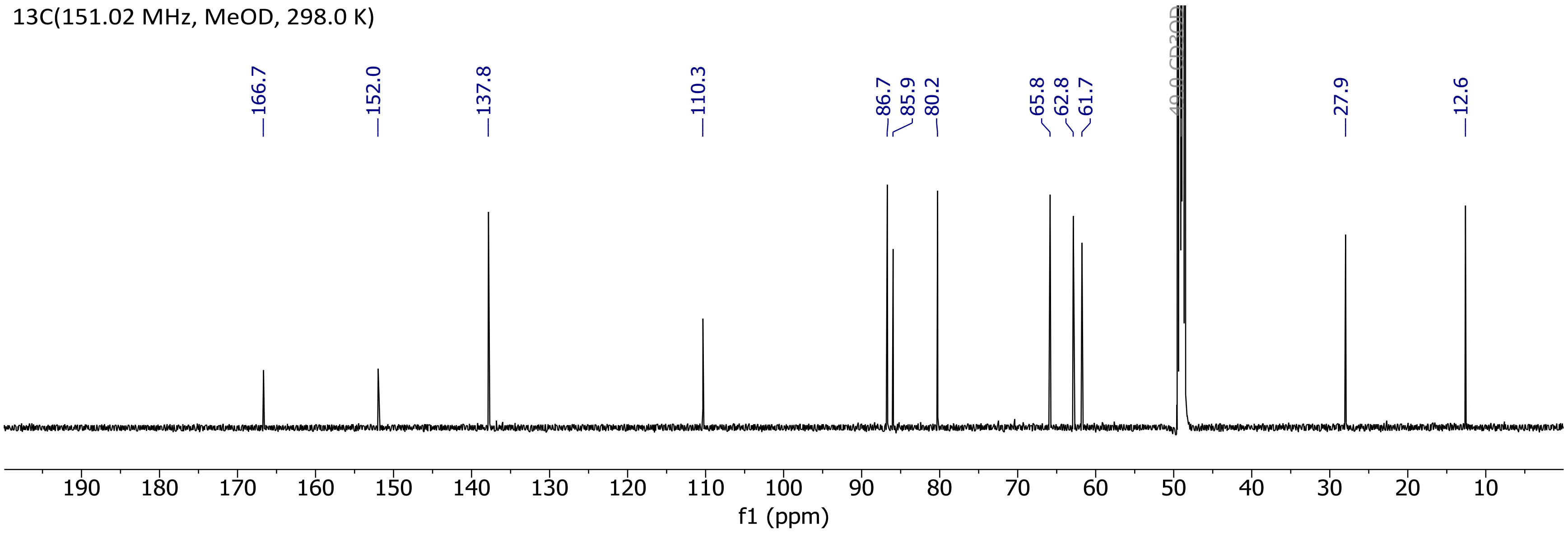
13C(151.02 MHz, MeOD, 298.0 K)
166.7
152.0
137.8
110.3
86.7
85.9
80.2
65.8
62.8
61.7
49.0 CD3OD
27.9
12.6
190 180 170 160 150 140 130 120 110 100 90 80 70 60 50 40 30 20 10
f1 (ppm)

1H(600.13 MHz, CD3CN, 298.0 K)

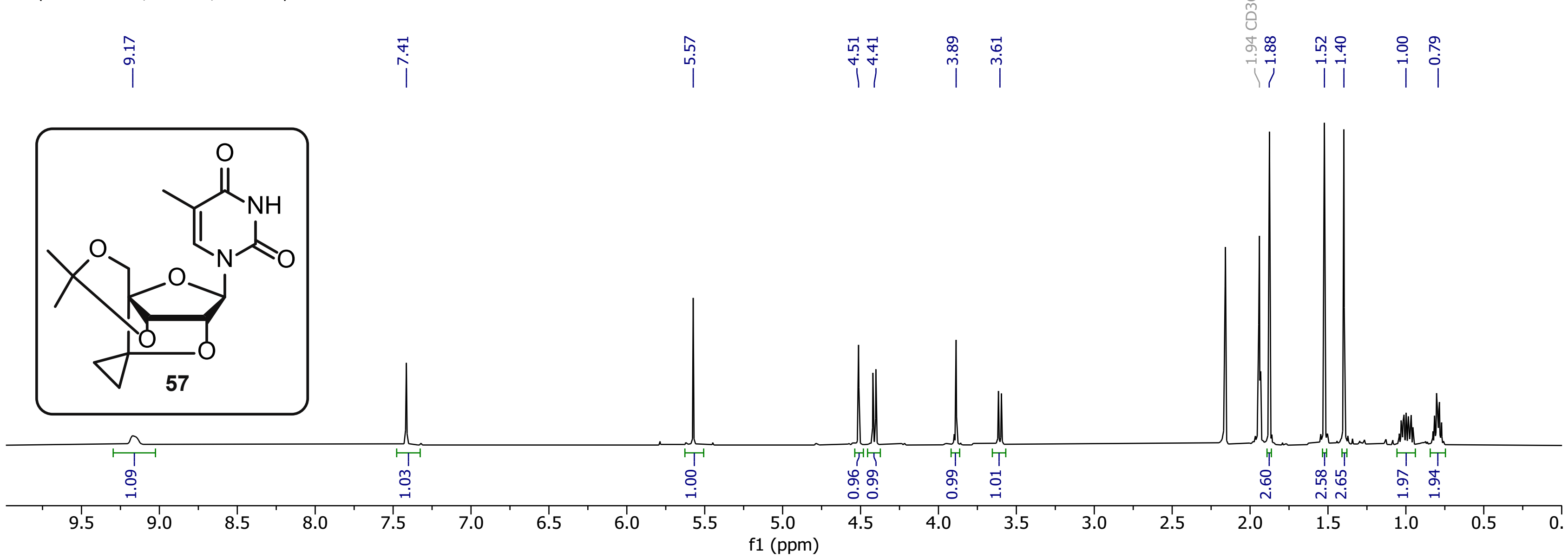


13C(150.92 MHz, CD3CN, 298.0 K)

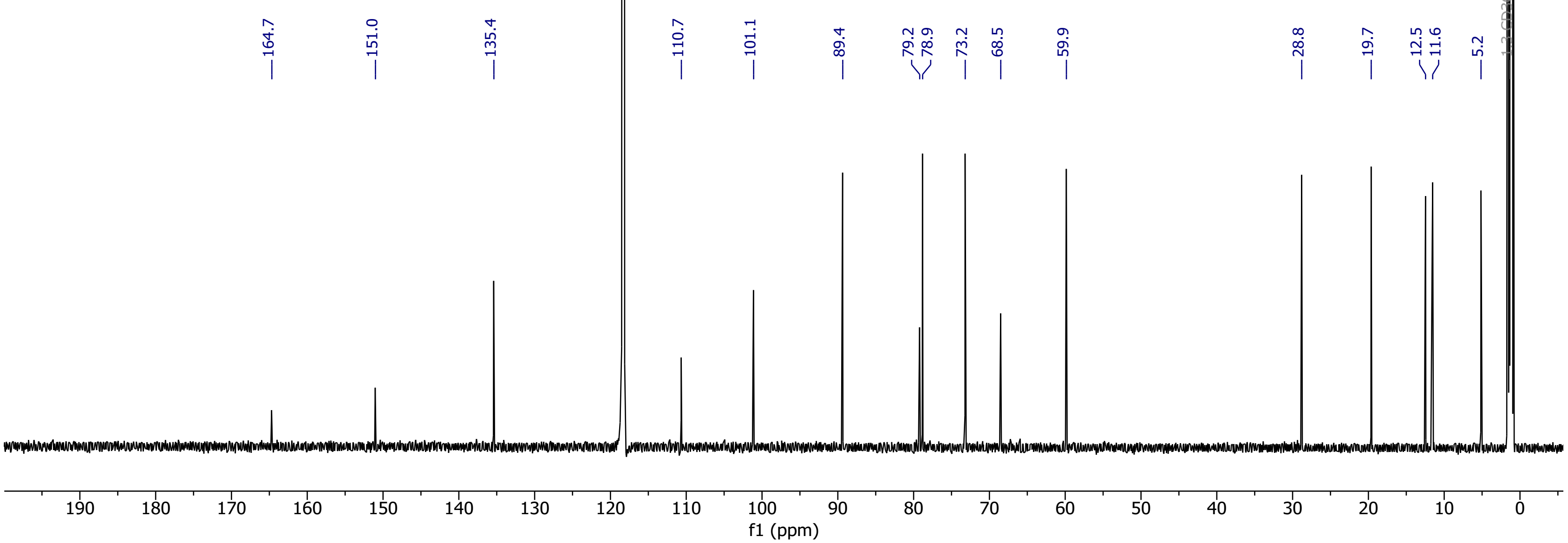

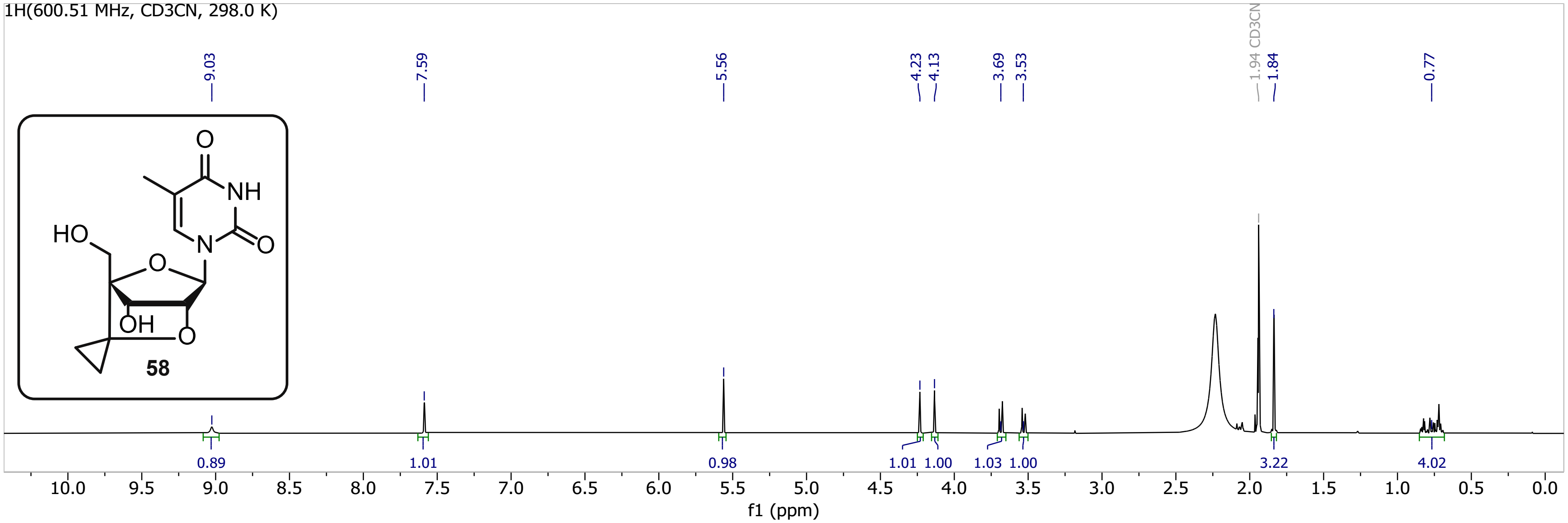
1H(600.51 MHz, CD3CN, 298.0 K)
9.03
7.59
5.56
4.23
4.13
3.69
3.53
1.94 CD3CN
1.84
0.77
O
NH
HO
O
N
O
OH
O
58
0.89
1.01
0.98
1.01
1.00
1.03
1.00
3.22
4.02
f1 (ppm)

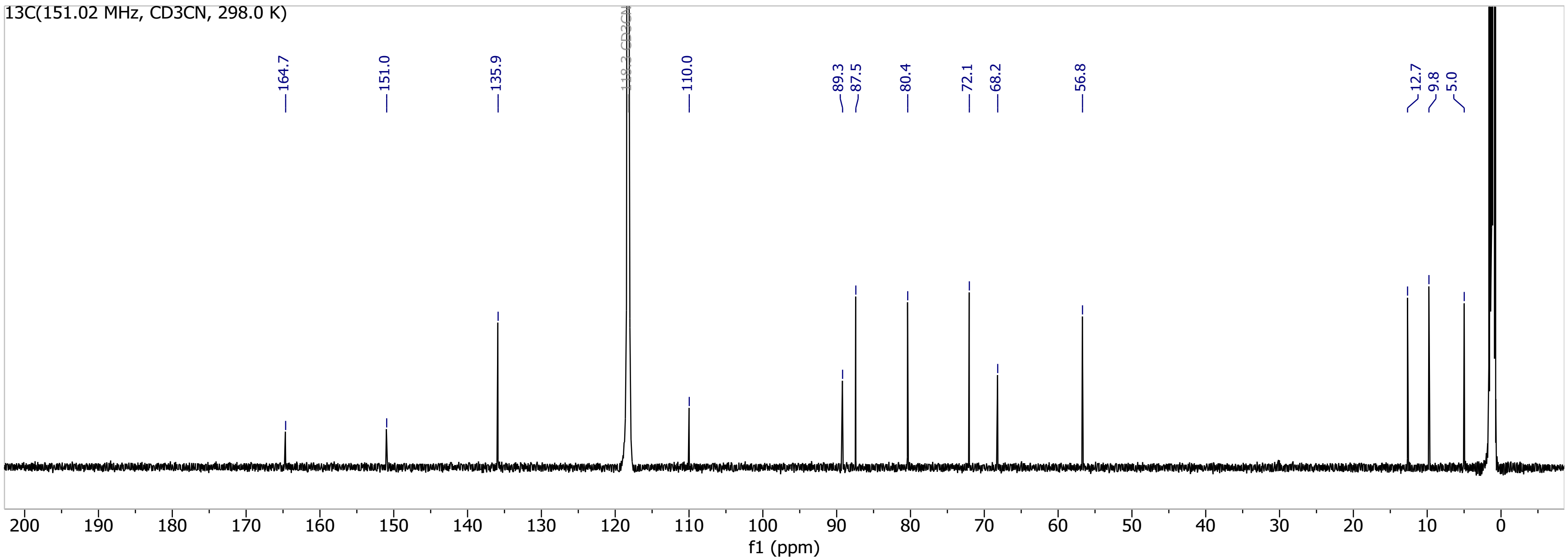
13C(151.02 MHz, CD3CN, 298.0 K)
164.7
151.0
135.9
118.3 CD3CN
110.0
89.3
87.5
80.4
72.1
68.2
56.8
12.7
9.8
5.0
f1 (ppm)

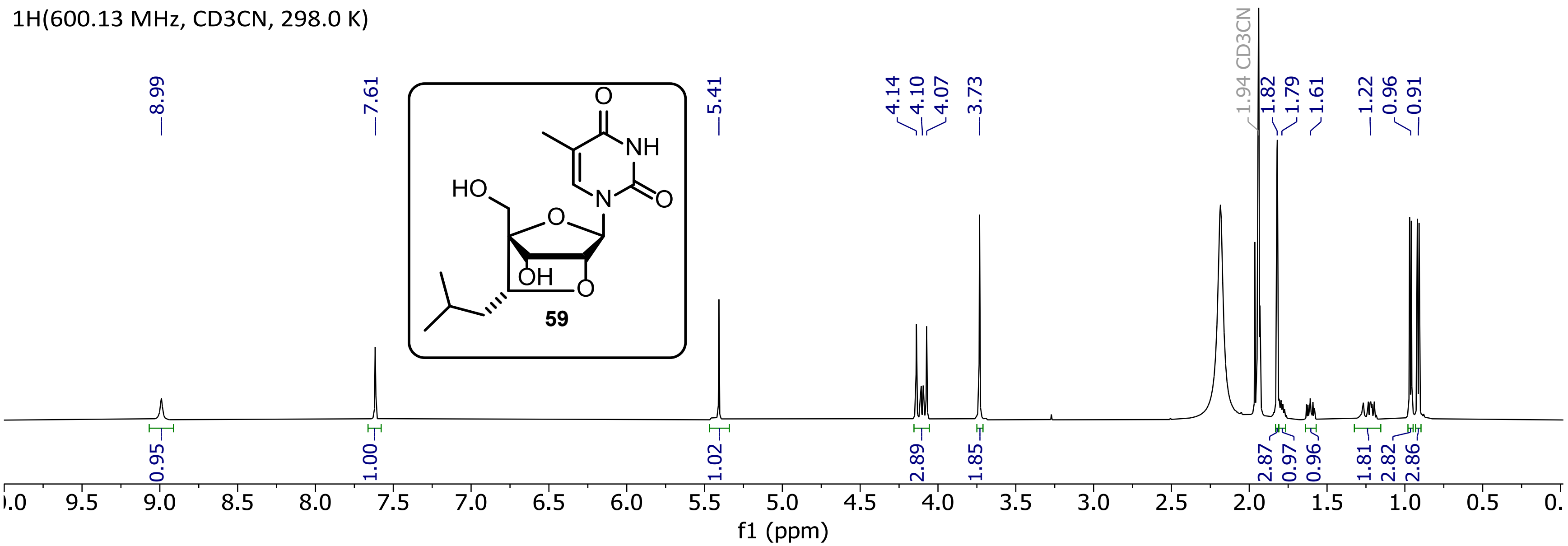
1H(600.13 MHz, CD3CN, 298.0 K)
8.99
7.61
5.41
4.14
4.10
4.07
3.73
1.94 CD3CN
1.82
1.79
1.61
1.22
0.96
0.91
0.95
1.00
1.02
2.89
1.85
2.87
0.97
0.96
1.81
2.82
2.86
59
f1 (ppm)

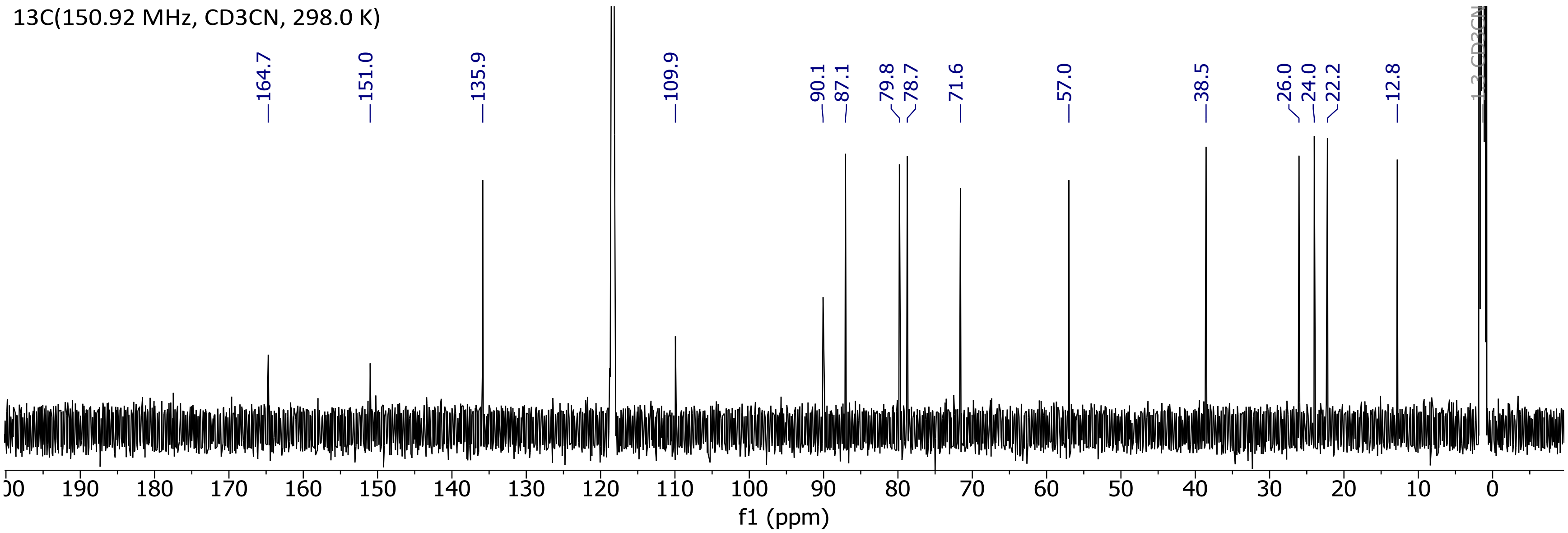
13C(150.92 MHz, CD3CN, 298.0 K)
164.7
151.0
135.9
109.9
90.1
87.1
79.8
78.7
71.6
57.0
38.5
26.0
24.0
22.2
12.8
f1 (ppm)

## 4.0 Removal of acetonide protecting group on analytical samples of BNAs 48-50

An analytical sample (~2 mg) of compound **48**, **49** or **50** was dissolved in 0.20 mL of a 95% aqueous TFA solution and stirred at ambient temperature for 10 min. MeOH was then added and the TFA–MeOH was removed under reduced pressure. The addition of MeOH and concentration was repeated three to four times to ensure complete removal of TFA and residual acetone. The resulting crude product was purified by silica gel flash chromatography (50:50 acetone/hexanes) to yield BNA **SI60**, **SI61**, or **SI62** as white foams.

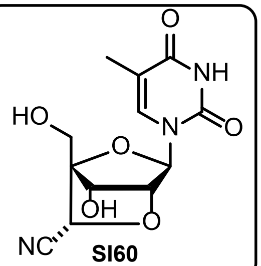
**SI60**

**Compound SI60: IR** (neat): υ = 3361, 2925, 2853, 2252, 1692, 1478, 1269, 1047, 587 cm$^{-1}$; $[\alpha]_D^{20}$= +24.6 (c 0.13 in MeOH), **$^1$H NMR** (600 MHz, $CD_3OD$) δ 7.71 (s, 1H), 5.70 (s, 1H), 4.93 (s, 1H), 4.46 (s, 1H), 4.28 (s, 1H), 3.97 (s, 2H), 1.89 (s, 3H); **$^{13}$C NMR (**151 MHz, $CD_3OD$) δ 166.5, 151.9, 136.3, 117.5, 111.2, 90.9, 88.1, 82.2, 71.4, 70.8, 56.7, 12.6.; **HRMS** (ESI$^+$) calcd for $[C_{12}H_{13}N_3O_6+H]^+$ 296.0877 m/z found: 296.0884 m/z.

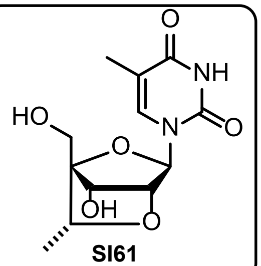
**SI61**

**Compound SI61: IR** (neat): υ = 3357, 2924, 2851, 1658, 1450, 1271, 1017, 880, 581 cm$^{-1}$; $[\alpha]_D^{20}$= +50.3 (c 0.1 in MeOH), **$^1$H NMR** (600 MHz, $CD_3OD$) δ 7.79 (d, $J$ = 1.3 Hz, 1H), 5.50 (s, 1H), 4.24 (q, $J$ = 6.4 Hz, 1H), 4.19 (s, 1H), 4.18 (s, 1H), 3.79 (d, $J$ = 1.4 Hz, 2H), 1.88 (d, $J$ = 1.2 Hz, 3H), 1.24 (d, $J$ = 6.4 Hz, 3H).; **$^{13}$C NMR** (151 MHz, $CD_3OD$) δ 169.4 (HMBC), 154.1 (HMBC), 136.6, 110.8, 90.0, 87.7, 80.7, 77.2, 71.7, 57.2, 14.2, 13.0.; **HRMS** (ESI$^+$) calcd for $[C_{12}H_{16}N_2O_6+H]^+$ 285.1081 m/z found: 285.1088 m/z.

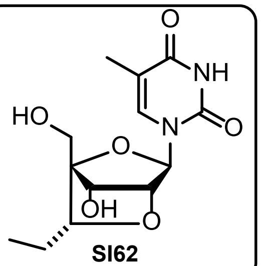
**SI62**

**Compound SI62: IR** (neat): υ = 3357, 2924, 2852, 1659, 1466, 1269, 1101, 1020, 586 cm$^{-1}$; $[\alpha]_D^{20}$= +34.1 (c 0.1 in MeOH), **$^1$H NMR** (600 MHz, $CD_3OD$_SPE) δ 7.82 (d, $J$ = 1.3 Hz, 1H), 5.48 (s, 1H), 4.20 (s, 1H), 4.18 (s, 1H), 4.00 (dd, $J$ = 9.8, 3.6 Hz, 1H), 3.86-3.78 (m, 2H), 1.89 (d, $J$ = 1.2 Hz, 3H), 1.73-1.65 (m, 1H), 1.57-1.50 (m, 1H), 1.05 (t, $J$ = 7.5 Hz, 3H); **$^{13}$C NMR** (151 MHz, $CD_3OD$_SPE) δ 166.6, 152.0, 136.9, 110.6, 90.4, 87.7, 82.8, 80.4, 71.8, 57.2, 23.2, 12.6, 10.9.; **HRMS** (ESI$^+$) calcd for $[C_{13}H_{18}N_2O_6+H]^+$ 299.1238 m/z found: 299.1244 m/z.

(1H(600.51 MHz, MeOD, 298.0 K))

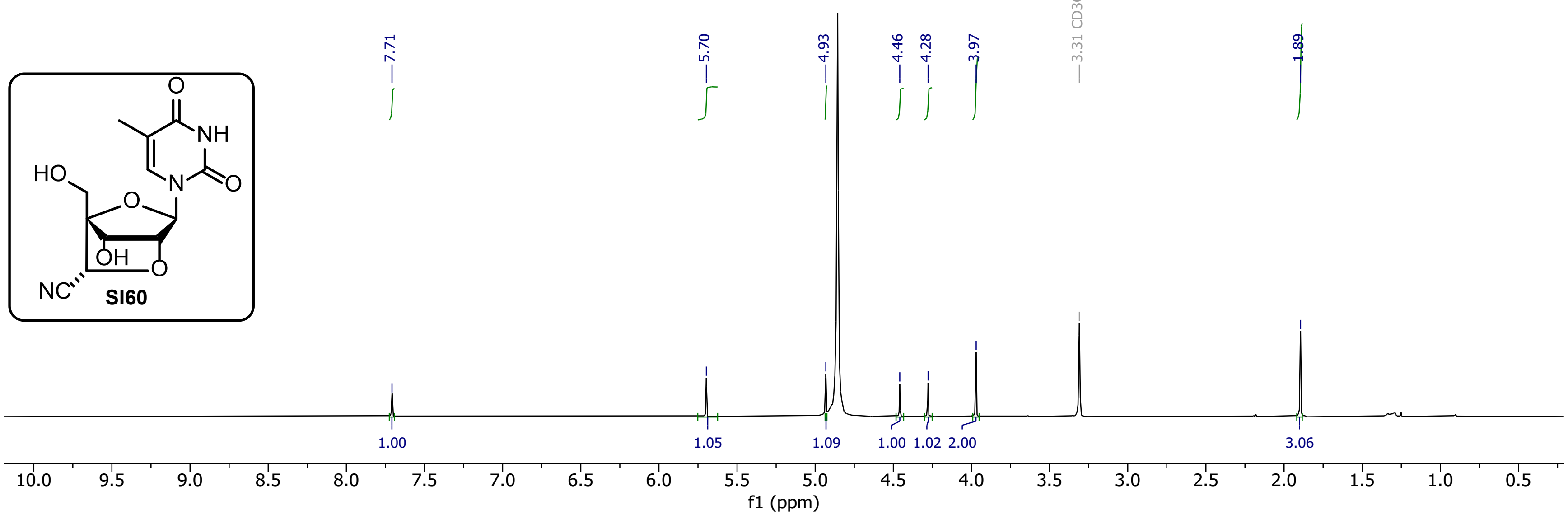


(13C(151.02 MHz, MeOD, 298.0 K))

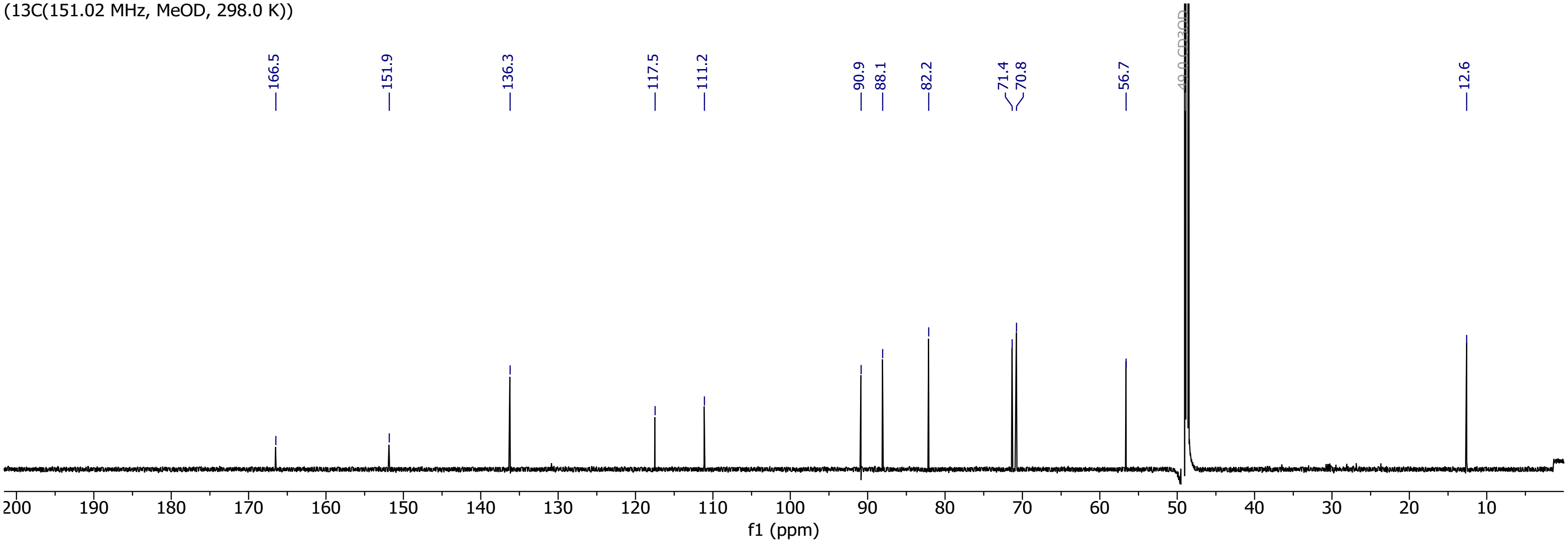

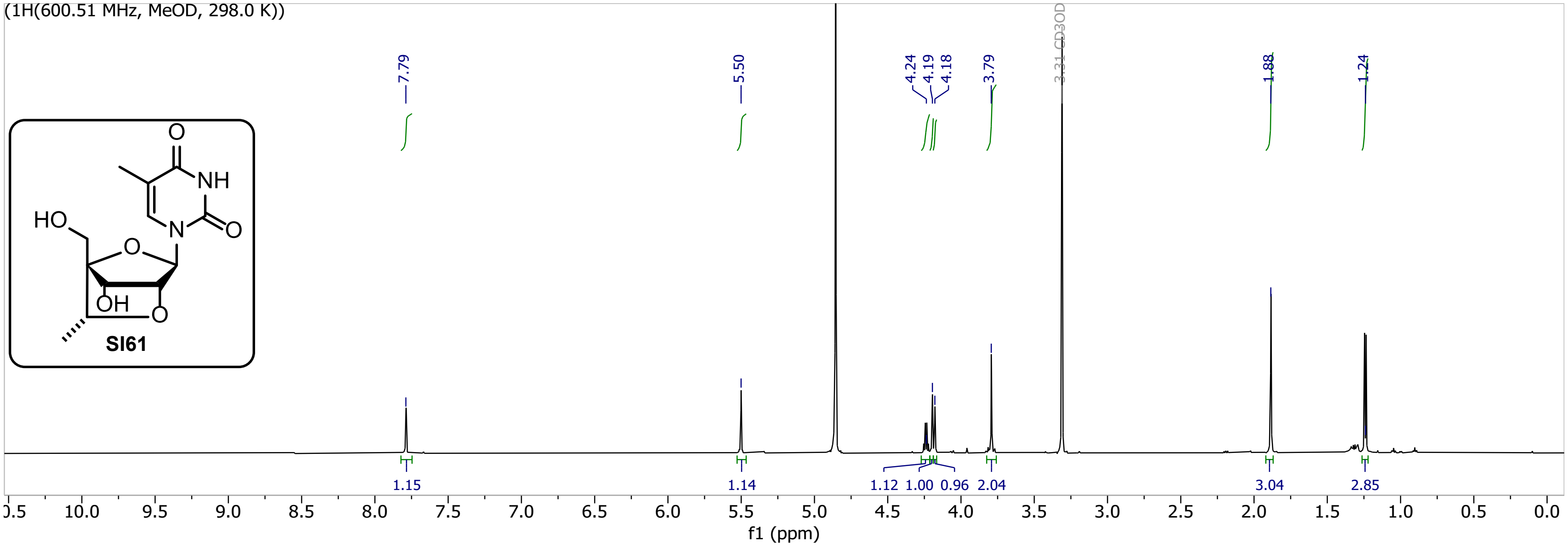
(1H(600.51 MHz, MeOD, 298.0 K))
7.79
5.50
4.24
4.19
4.18
3.79
3.31 CD3OD
1.88
1.24
1.15
1.14
1.12
1.00
0.96
2.04
3.04
2.85
f1 (ppm)
SI61

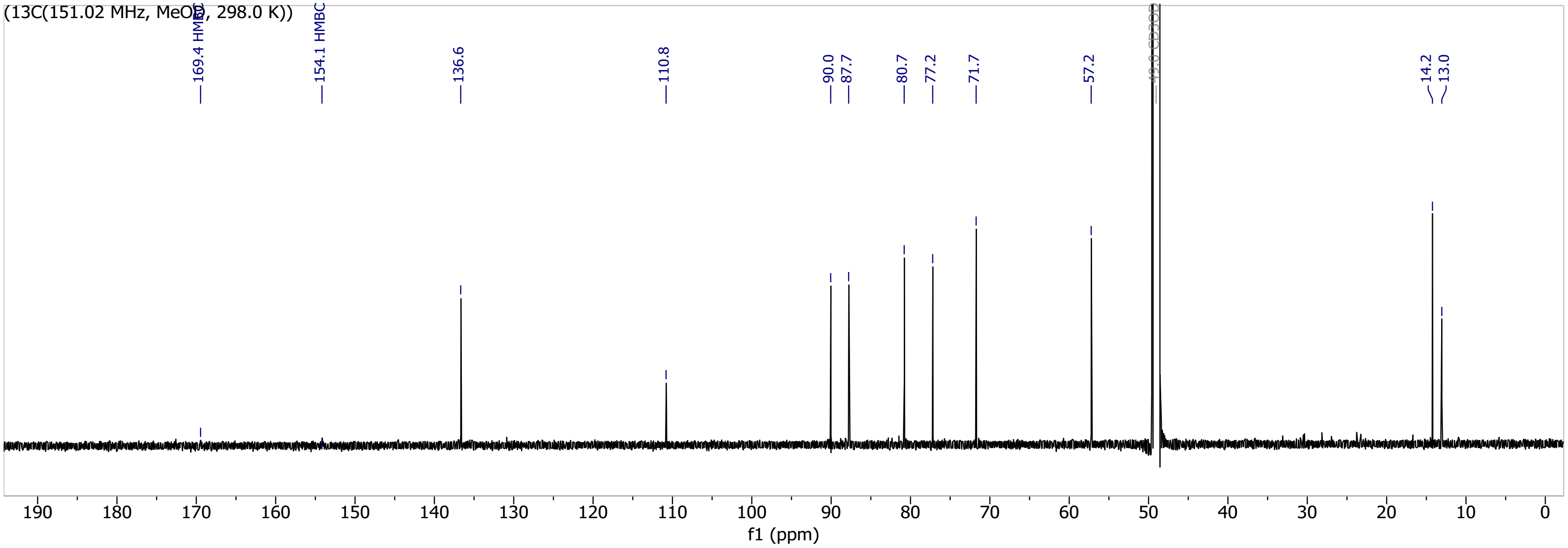
(13C(151.02 MHz, MeOD, 298.0 K))
169.4 HMBC
154.1 HMBC
136.6
110.8
90.0
87.7
80.7
77.2
71.7
57.2
14.2
13.0
f1 (ppm)

(1H(600.13 MHz, CD3OD_SPE, 298.0 K))

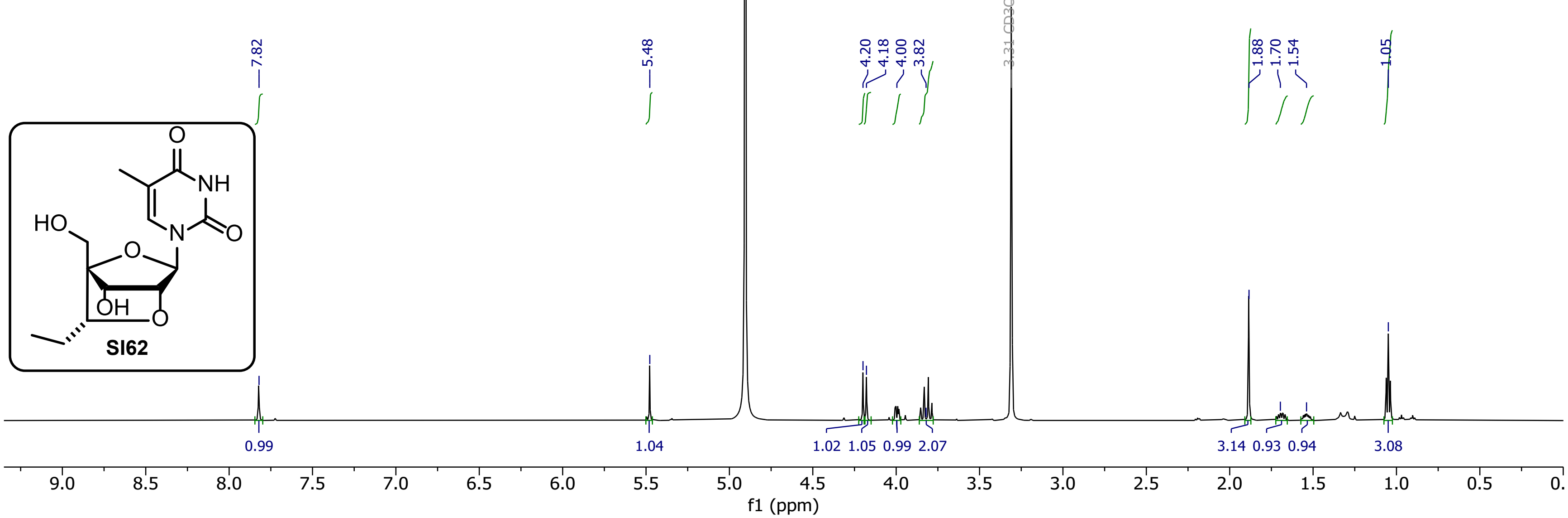


(13C(150.92 MHz, CD3OD_SPE, 298.0 K))

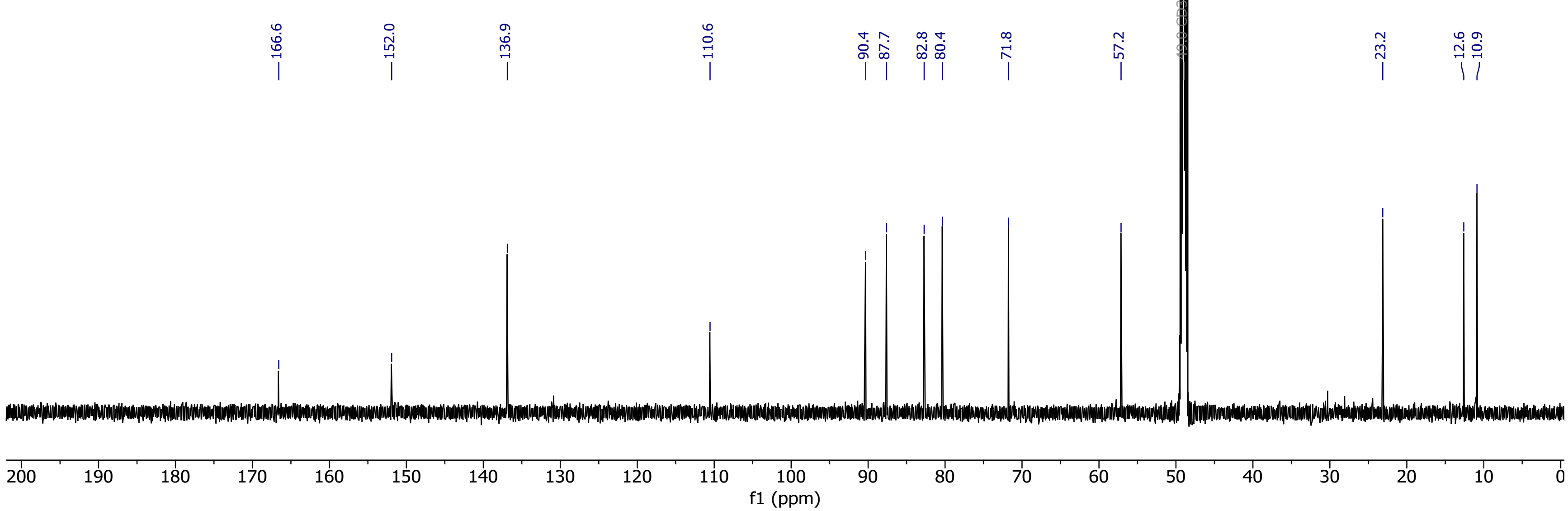

## 5.0 Multi-Gram Preparation of Compound 18

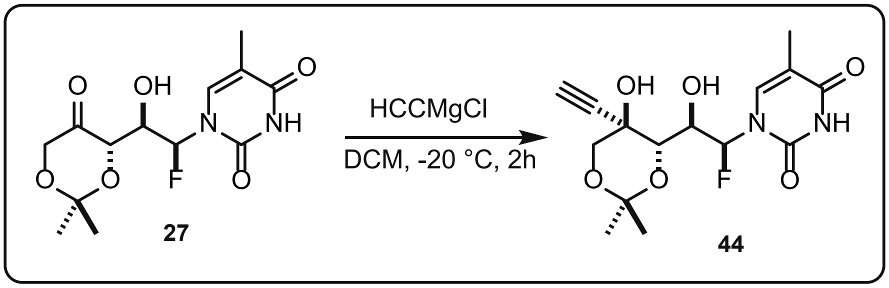


A solution of HCCMgCl (758 mL, 379 mmol, 0.5 M in DCM) in DCM (2.32 L) was added to compound **27** (30.0 g, 94.9 mmol) and the mixture was cooled to -20 °C. The reaction was allowed to stir at -20 °C for 2 hours and then was quenched with saturated $NH_4Cl$ (1.50 L). The mixture was warmed to room temperature, extracted with DCM (1.2 L x 3), dried over $Na_2SO_4$, and the solvent was removed under reduced pressure to yield crude compound **44** as a yellow solid (32.5 g).

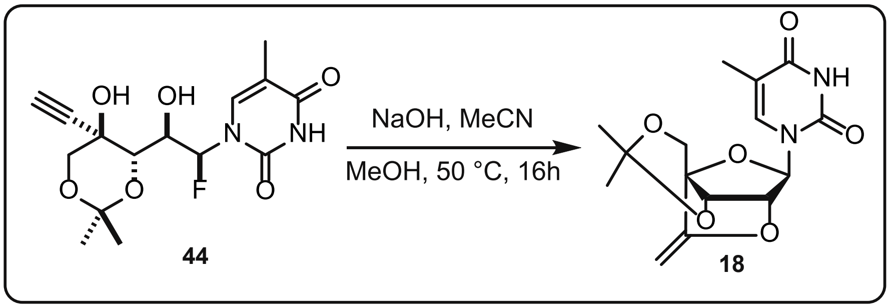


A solution of NaOH (63.3 g, 1.58 mol) was dissolved in MeCN (1.4 L) and MeOH (350 mL) and added to crude compound **44** (54.1 g, 158 mmol). The reaction was allowed to stir at 50 °C for 16 h. Once all starting material was consumed, the solvent was removed under reduced pressure and saturated $NH_4Cl$ (500 mL) and EtOAc (250 mL) was added to the crude. The aqueous layer was extracted with EtOAc (180 mL x 3) and the organic layers were combined, dried over $Na_2SO_4$, and the solvent was removed under reduced pressure. The residue was then purified by flash column chromatography (DCM:THF, 0% THF to 100% THF) to achieve a crude product (92% pure) which was further purified by prep-HPLC (column: Ultimate XB-DIOL 250x50mm, 10um; mobile phase: Heptane:EtOH, 0% to 32% EtOH over 18 min) to afford compound **18** as a yellow solid (13.5 g, 41.9 mmol, 27% over 2 steps).

**Compound 18: IR** (neat): υ = 2996, 2825, 1705. 1671, 1473, 1371, 1265, 1044, 909, 731, 574 $cm^{-1}$; $[\alpha]_D^{20}$= +1.5 (c 1.20 in MeCN), **$^1$H NMR** (500 MHz, $CD_3CN$) δ 9.16 (s, 1H), 7.38 (q, *J* = 1.3 Hz, 1H), 5.45 (s, 1H), 4.79 (s, 1H), 4.51 (m, 2H), 4.35 (d, *J* = 2.9 Hz, 1H), 4.23 (dd, *J* = 11.4, 1.0 Hz, 1H), 3.90 (s, 1H), 1.88 (d, *J* = 1.3 Hz, 3H), 1.50 (s, 3H), 1.34 (s, 3H); **$^{13}$C NMR** (126 MHz, $CD_3CN$) δ 164.6, 160.2, 150.9, 135.3, 111.0, 101.8, 88.8, 83.5, 79.6, 77.8, 73.3, 59.9, 29.1, 19.6, 12.4. **HRMS** ($ESI^+$) calcd for $[C_{15}H_{18}N_2O_6+H]^+$ 323.1338 m/z found: 323.1343 m/z.

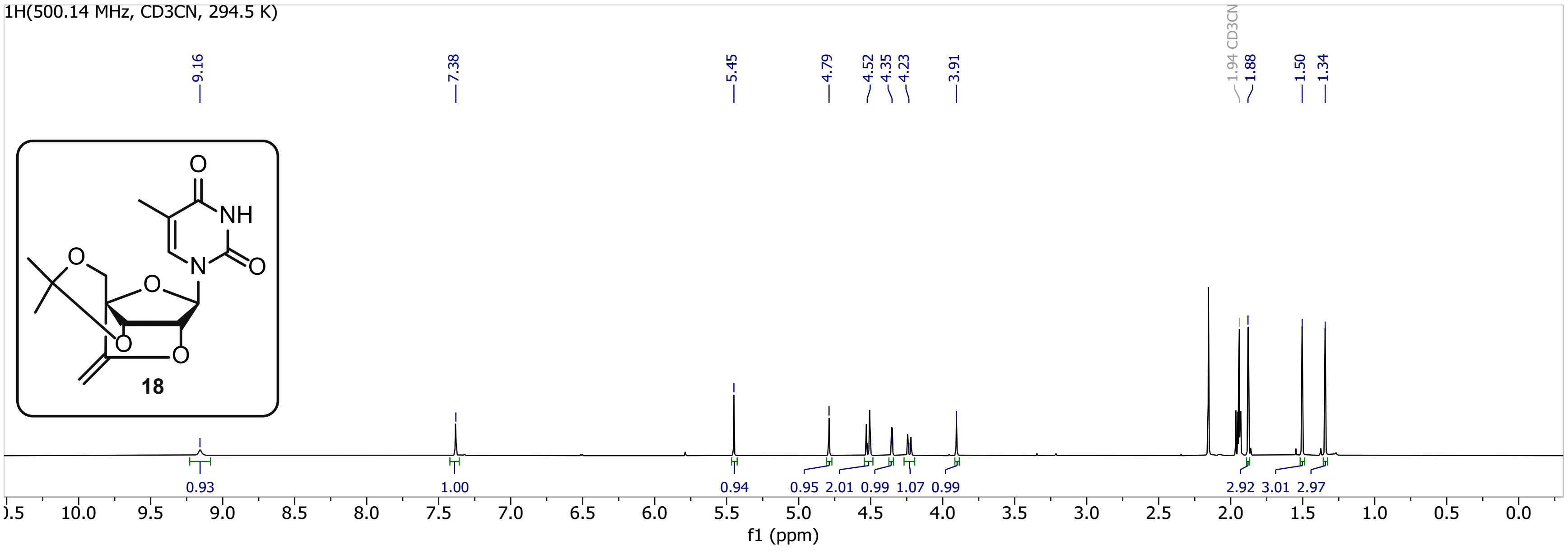
1H(500.14 MHz, CD3CN, 294.5 K)
9.16
7.38
5.45
4.79
4.52
4.35
4.23
3.91
1.94 CD3CN
1.88
1.50
1.34
NH
O
N
18
0.93
1.00
0.94
0.95
2.01
0.99
1.07
0.99
2.92
3.01
2.97
f1 (ppm)

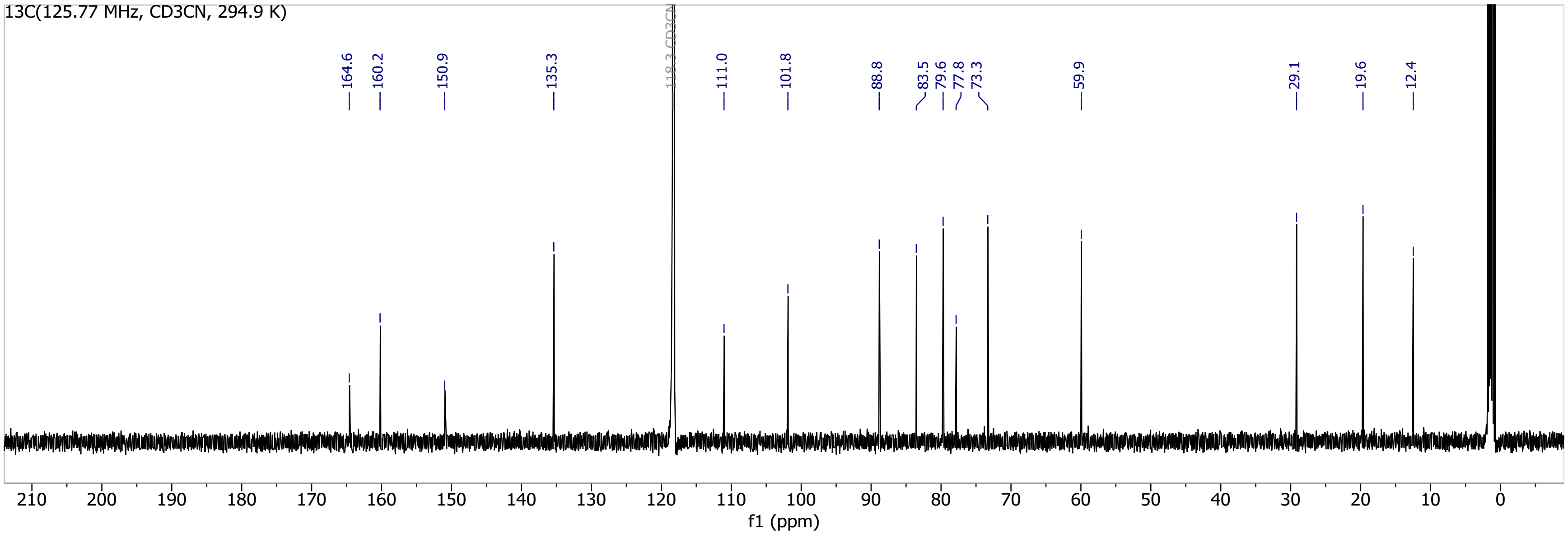
13C(125.77 MHz, CD3CN, 294.9 K)
164.6
160.2
150.9
135.3
111.0
101.8
88.8
83.5
79.6
77.8
73.3
59.9
29.1
19.6
12.4
f1 (ppm)

## 6.0 Computational Details

Gas-phase conformation searches were performed using MacroModel interfaced to the Maestro program.[1–3] The Monte Carlo Multiple Minimum (MCMM) torsional sampling method was used to generate the initial set of conformers.[4] A maximum of 5000 steps was set up to find the most relevant conformers, at least 10 times each, within an energy window of 5 kcal mol$^{-1}$. The OPLS3 force field was used along with a RMSD cut-off of 0.25 Å,[5] as implemented on MacroModel, setting a 0.01 threshold converging on gradient.[6]

Density Functional Theory (DFT) calculations were performed using the Gaussian 16 Rev. A.01 suite of programs.[7] Gas-phase optimizations were performed with the ωB97X-D functional[8] using the 6-31G(*d*) basis set[9] and an ultrafine integration grid. To verify the nature of the stationary points, frequency calculations were performed at the same level of theory at which the optimization was done—no imaginary frequencies were found for ground-state minima. Subsequently, single point energies were recalculated on each optimized structure using the ωB97X-D functional and the def2-TZVPP basis set,[10,11] along with the SMD solvation model (solvent=acetonitrile).[12] This level of theory has been widely suggested and proven to give accurate results to describe organic reactions.[13–16] A superfine integration gris was used for the latter calculations.

Gibbs free energies were calculated using the entropic quasi-harmonic treatment with a frequency cut-off value of 100 cm$^{-1}$, using Grimme's mRRHO treatment[17] and the higher-level single point energies. Thermal correction at 298 K (25 °C) was also applied. Reported values are Boltzmann-averaged over the found conformers, unless otherwise specified. These post-calculations were done using the GoodVibes v3.2 program developed by Robert Paton's group.[18]

Ground-state figures were rendered using the CYLview software.[19]

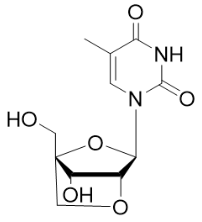


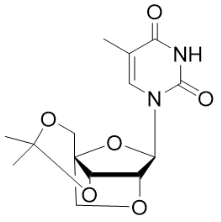


**34** **37**

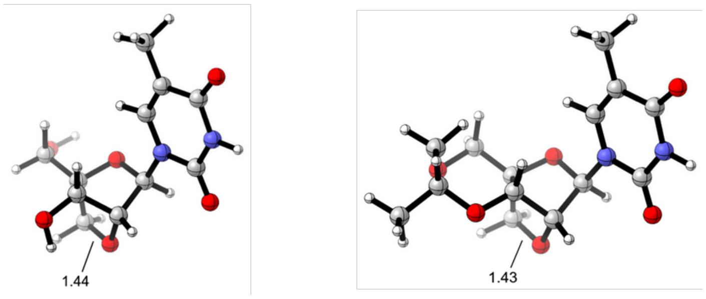


**Figure S1.** Optimized, lowest-energy conformer structures for compounds **34** (left) and **37** (right). Relevant distances between the C2' and C4' positions locking the NA are given in Å.

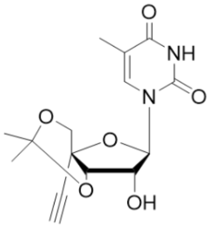


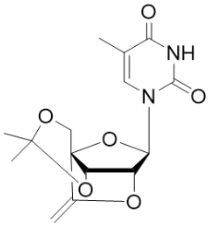


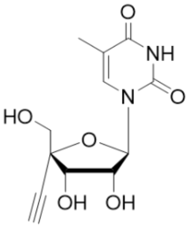


**17** **18** **17'**

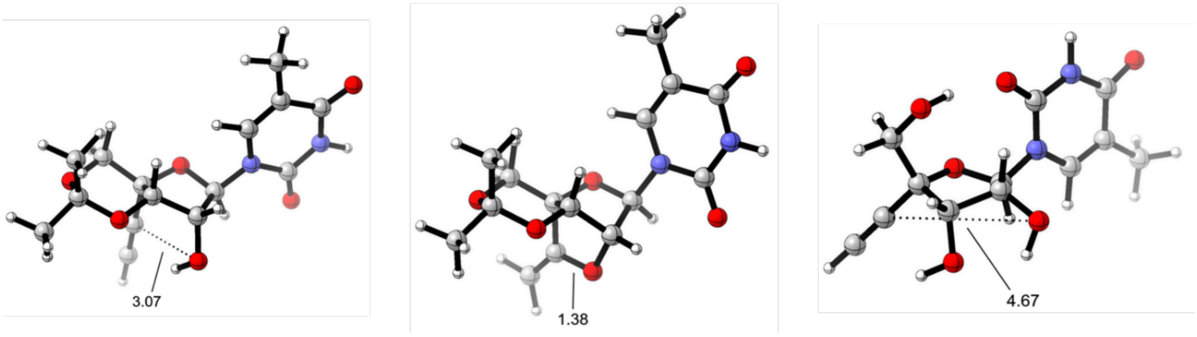


**Figure S2**. Optimized, lowest-energy conformer structures for compounds **17** (left), **18** (center) and **17'** (right). Relevant distances between the C2' and C4' positions locking the NA are given in Å.

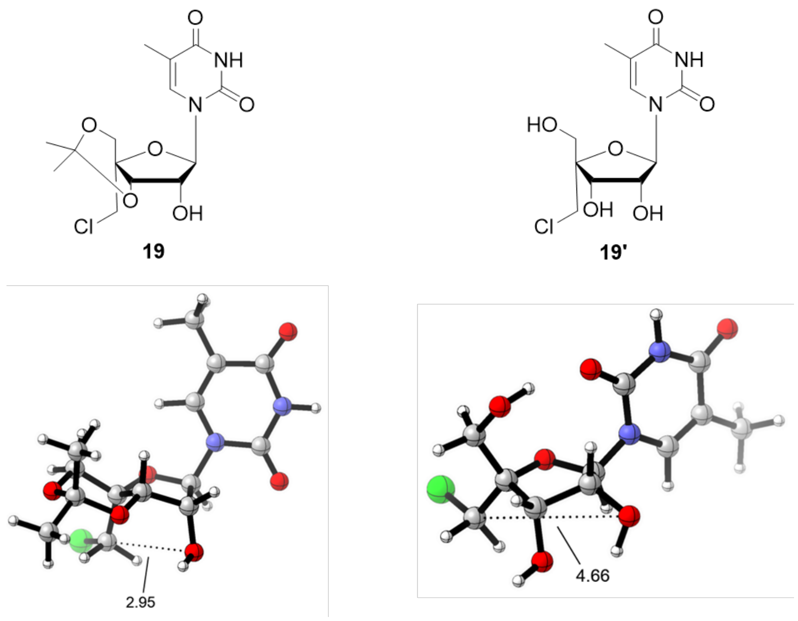


**Figure S3.** Optimized, lowest-energy conformer structures for compounds **19** (left) and **19'** (right). Relevant distances between the C2' and C4' positions locking the NA are given in Å.

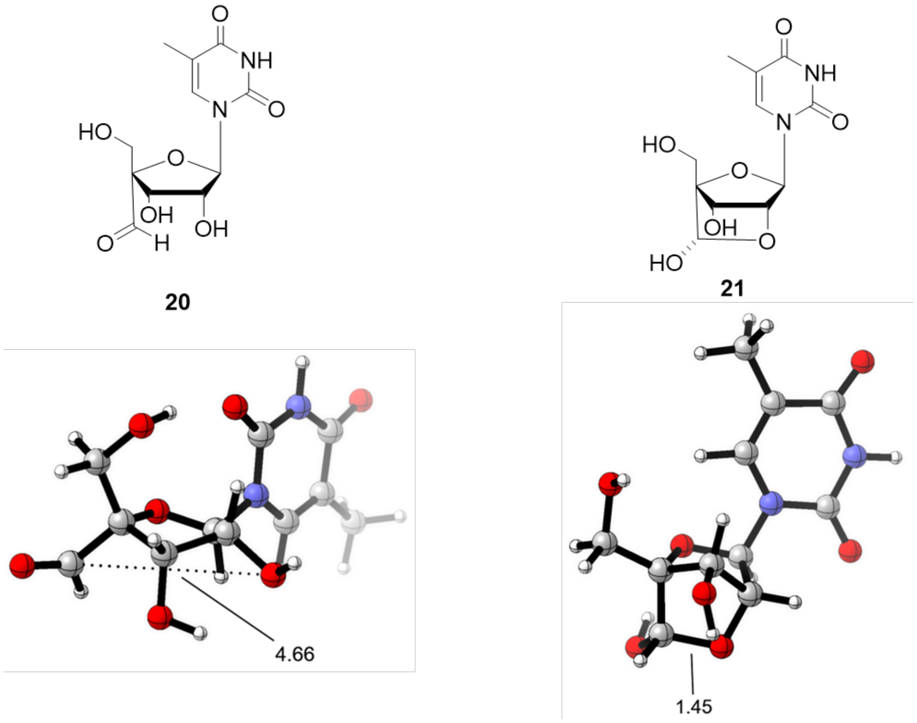


**Figure S4.** Optimized, lowest-energy conformer structures for compounds **20** (left) and **21** (right). Relevant distances between the C2' and C4' positions locking the NA are given in Å.

Calculated equilibrium ratio between **20** and **21**. Unprotected (C3'-C5') NA favors the open form, i.e. compound **20** by 4.21 kcal $mol^{-1}$. >100:1 ratio (**20**:**21**). ΔG value is Boltzmann-averaged.

| Selectivity | Excess (%) | Ratio (%) | Ratio | Major Iso | ΔG |
|---|---|---|---|---|---|

************************************************************************************************************

| | | | | | |
|---|---|---|---|---|---|
| | 99.84 | 0:100 | 1:1218 | **20** | 4.21 |

************************************************************************************************************

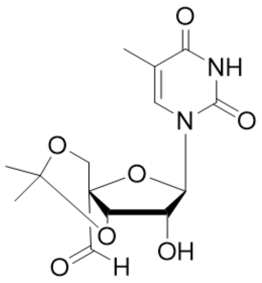


**22**

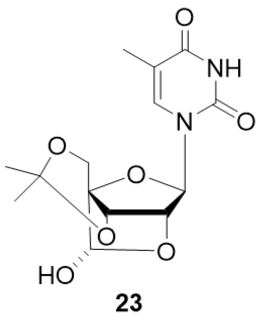


**23**

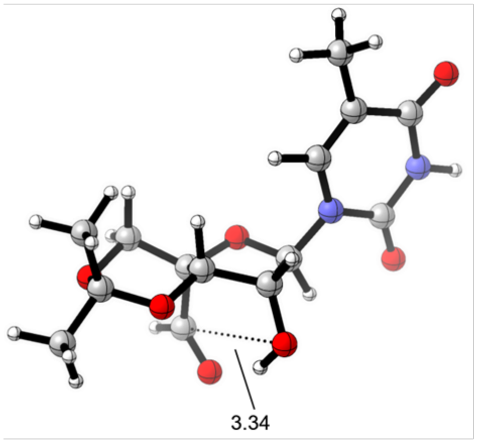


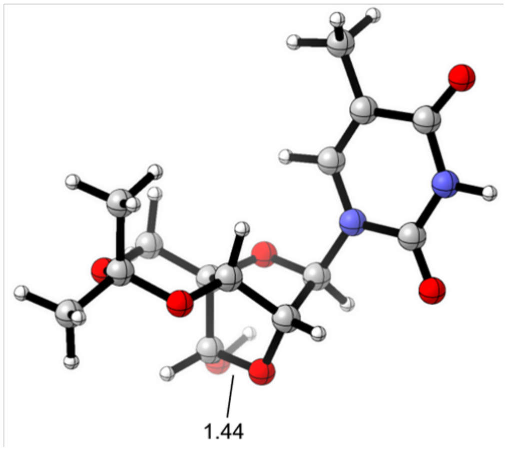


**Figure S5.** Optimized, lowest-energy conformer structures for compounds **22** (left) and **23** (right). Relevant distances between the C2’ and C4’ positions locking the NA are given in Å.

Calculated equilibrium ratio between **22** and **23**. Acetonide-protected (C3’-C5’) AP-NA favors the locked form, i.e. hemiacetal **23** by 1.58 kcal mol$^{-1}$. 14:1 ratio (**23**:**22**). ΔG value is Boltzmann-averaged.

| Selectivity | Excess (%) | Ratio (%) | Ratio | Major Iso | ΔG |
|---|---|---|---|---|---|
| | 86.97 | 7:93 | 1:14 | **23** | 1.58 |

************************************************************************************************************

## Table of energies for optimized structures

**Table S1.** Energies for compound **34**. Electronic energies, enthalpies, entropies, and free energies are presented in atomic units.

| Structure | E_SPC | E | ZPE | H_SPC | T.S | T.qh-S | G(T)_SPC | qh-G(T)_SPC | Boltz |
|---|---|---|---|---|---|---|---|---|---|
| LNA-LockedmonoCl-Diol_1 | -988.573451 | -988.144290 | 0.266198 | -988.289752 | 0.061961 | 0.059864 | -988.351713 | -988.349616 | 0.193 |
| LNA-LockedmonoCl-Diol_10 | -988.571907 | -988.139733 | 0.265790 | -988.288438 | 0.062355 | 0.060222 | -988.350792 | -988.348659 | 0.070 |
| LNA-LockedmonoCl-Diol_11 | -988.568160 | -988.139279 | 0.265856 | -988.284653 | 0.062123 | 0.060215 | -988.346776 | -988.344868 | 0.001 |
| LNA-LockedmonoCl-Diol_12 | -988.572342 | -988.139168 | 0.265635 | -988.289001 | 0.062313 | 0.060253 | -988.351314 | -988.349254 | 0.131 |
| LNA-LockedmonoCl-Diol_13 | -988.573547 | -988.145332 | 0.266537 | -988.289640 | 0.061295 | 0.059475 | -988.350935 | -988.349115 | 0.113 |
| LNA-LockedmonoCl-Diol_14 | -988.572151 | -988.145645 | 0.266529 | -988.288376 | 0.061128 | 0.059274 | -988.349504 | -988.347650 | 0.024 |
| LNA-LockedmonoCl-Diol_15 | -988.568724 | -988.139457 | 0.266062 | -988.285111 | 0.062276 | 0.060128 | -988.347387 | -988.345239 | 0.002 |
| LNA-LockedmonoCl-Diol_16 | -988.571882 | -988.140927 | 0.265670 | -988.288559 | 0.062174 | 0.060153 | -988.350733 | -988.348712 | 0.074 |
| LNA-LockedmonoCl-Diol_2 | -988.572092 | -988.145653 | 0.266420 | -988.288414 | 0.061126 | 0.059294 | -988.349540 | -988.347708 | 0.026 |
| LNA-LockedmonoCl-Diol_3 | -988.571869 | -988.140926 | 0.265621 | -988.288588 | 0.062213 | 0.060179 | -988.350800 | -988.348766 | 0.078 |
| LNA-LockedmonoCl-Diol_4 | -988.573290 | -988.144502 | 0.266278 | -988.289549 | 0.061499 | 0.059636 | -988.351048 | -988.349185 | 0.122 |
| LNA-LockedmonoCl-Diol_5 | -988.568657 | -988.139458 | 0.266099 | -988.285039 | 0.062134 | 0.060035 | -988.347174 | -988.345075 | 0.002 |
| LNA-LockedmonoCl-Diol_6 | -988.570692 | -988.138471 | 0.266260 | -988.286901 | 0.063016 | 0.060435 | -988.349916 | -988.347336 | 0.017 |
| LNA-LockedmonoCl-Diol_7 | -988.567662 | -988.141167 | 0.266338 | -988.284014 | 0.061528 | 0.059581 | -988.345543 | -988.343595 | 0.000 |
| LNA-LockedmonoCl-Diol_8 | -988.567026 | -988.136202 | 0.265610 | -988.283732 | 0.062459 | 0.060375 | -988.346191 | -988.344108 | 0.001 |
| LNA-LockedmonoCl-Diol_9 | -988.573581 | -988.145330 | 0.266373 | -988.289814 | 0.061359 | 0.059533 | -988.351173 | -988.349347 | 0.145 |

**Table S2.** Energies for compound **37**. Electronic energies, enthalpies, entropies, and free energies are presented in atomic units.

| Structure | E_SPC | E | ZPE | H_SPC | T.S | T.qh-S | G(T)_SPC | qh-G(T)_SPC | Boltz |
|---|---|---|---|---|---|---|---|---|---|
| o LNA-LockedmonoCl-Acet_1 | -1105.303096 | -1104.850607 | 0.330097 | -1104.953459 | 0.065921 | 0.063713 | -1105.019380 | -1105.017172 | 0.913 |
| o LNA-LockedmonoCl-Acet_2 | -1105.298304 | -1104.846441 | 0.330160 | -1104.948552 | 0.066259 | 0.063981 | -1105.014811 | -1105.012533 | 0.007 |
| o LNA-LockedmonoCl-Acet_3 | -1105.300590 | -1104.843946 | 0.330213 | -1104.950758 | 0.066429 | 0.064067 | -1105.017187 | -1105.014824 | 0.076 |
| o LNA-LockedmonoCl-Acet_4 | -1105.295305 | -1104.843619 | 0.330453 | -1104.945354 | 0.065935 | 0.063642 | -1105.011289 | -1105.008996 | 0.000 |
| o LNA-LockedmonoCl-Acet_5 | -1105.297488 | -1104.844994 | 0.329732 | -1104.948102 | 0.066351 | 0.063970 | -1105.014453 | -1105.012072 | 0.004 |
| o LNA-LockedmonoCl-Acet_6 | -1105.292157 | -1104.840158 | 0.329690 | -1104.942763 | 0.066711 | 0.064246 | -1105.009473 | -1105.007009 | 0.000 |

**Table S3.** Energies for compound **17**. Electronic energies, enthalpies, entropies, and free energies are presented in atomic units.

| Structure | E_SPC | E | ZPE | H_SPC | T.S | T.qh-S | G(T)_SPC | qh-G(T)_SPC | Boltz |
|---|---|---|---|---|---|---|---|---|---|
| o LNA-OpenAlkynyl-Acet_1 | -1143.348532 | -1142.869539 | 0.331841 | -1142.994643 | 0.071119 | 0.068579 | -1143.065762 | -1143.063222 | 0.128 |
| o LNA-OpenAlkynyl-Acet_2 | -1143.349808 | -1142.866630 | 0.331583 | -1142.996151 | 0.071765 | 0.068874 | -1143.067917 | -1143.065025 | 0.863 |
| o LNA-OpenAlkynyl-Acet_3 | -1143.345646 | -1142.865566 | 0.331766 | -1142.991912 | 0.071524 | 0.068728 | -1143.063436 | -1143.060640 | 0.008 |
| o LNA-OpenAlkynyl-Acet_4 | -1143.340410 | -1142.864271 | 0.331229 | -1142.986938 | 0.071516 | 0.069051 | -1143.058454 | -1143.055989 | 0.000 |
| o LNA-OpenAlkynyl-Acet_5 | -1143.341809 | -1142.862237 | 0.331186 | -1142.988302 | 0.072303 | 0.069470 | -1143.060605 | -1143.057772 | 0.000 |

**Table S4.** Energies for compound **18**. Electronic energies, enthalpies, entropies, and free energies are presented in atomic units.

| Structure | E_SPC | E | ZPE | H_SPC | T.S | T.qh-S | G(T)_SPC | qh-G(T)_SPC | Boltz |
|---|---|---|---|---|---|---|---|---|---|
| o LNA-LockedAlkynyl-Acet_1 | -1143.390087 | -1142.922372 | 0.334240 | -1143.035294 | 0.068067 | 0.065699 | -1143.103361 | -1143.100993 | 0.899 |
| o LNA-LockedAlkynyl-Acet_2 | -1143.385959 | -1142.918551 | 0.334408 | -1143.030966 | 0.068301 | 0.065881 | -1143.099267 | -1143.096847 | 0.011 |
| o LNA-LockedAlkynyl-Acet_3 | -1143.385113 | -1142.919589 | 0.334060 | -1143.030479 | 0.068149 | 0.065772 | -1143.098628 | -1143.096252 | 0.006 |
| o LNA-LockedAlkynyl-Acet_4 | -1143.387522 | -1142.914899 | 0.334245 | -1143.032621 | 0.068783 | 0.066135 | -1143.101404 | -1143.098756 | 0.084 |

**Table S5.** Energies for compound **17’**. Electronic energies, enthalpies, entropies, and free energies are presented in atomic units.

| Structure | E_SPC | E | ZPE | H_SPC | T.S | T.qh-S | G(T)_SPC | qh-G(T)_SPC | Boltz |
|---|---|---|---|---|---|---|---|---|---|
| o LNA-OpenAlkynyl-Diol_1 | -1026.627881 | -1026.170916 | 0.268508 | -1026.339915 | 0.065502 | 0.063553 | -1026.405417 | -1026.403467 | 0.164 |
| o LNA-OpenAlkynyl-Diol_10 | -1026.624665 | -1026.167910 | 0.267473 | -1026.337164 | 0.067754 | 0.064924 | -1026.404918 | -1026.402087 | 0.038 |
| o LNA-OpenAlkynyl-Diol_11 | -1026.626186 | -1026.174306 | 0.268321 | -1026.338533 | 0.065290 | 0.063300 | -1026.403823 | -1026.401833 | 0.029 |
| o LNA-OpenAlkynyl-Diol_2 | -1026.629121 | -1026.175992 | 0.268476 | -1026.341227 | 0.065439 | 0.063462 | -1026.406666 | -1026.404689 | 0.597 |
| o LNA-OpenAlkynyl-Diol_3 | -1026.624515 | -1026.172414 | 0.268184 | -1026.336874 | 0.066022 | 0.063658 | -1026.402896 | -1026.400532 | 0.007 |
| o LNA-OpenAlkynyl-Diol_4 | -1026.626690 | -1026.174018 | 0.268016 | -1026.339073 | 0.066184 | 0.063899 | -1026.405257 | -1026.402973 | 0.097 |
| o LNA-OpenAlkynyl-Diol_5 | -1026.624603 | -1026.171964 | 0.267976 | -1026.337078 | 0.065987 | 0.063822 | -1026.403066 | -1026.400900 | 0.011 |
| o LNA-OpenAlkynyl-Diol_6 | -1026.624677 | -1026.171960 | 0.267889 | -1026.337225 | 0.066027 | 0.063873 | -1026.403252 | -1026.401098 | 0.013 |
| o LNA-OpenAlkynyl-Diol_7 | -1026.623760 | -1026.163066 | 0.267364 | -1026.336361 | 0.067047 | 0.064760 | -1026.403408 | -1026.401121 | 0.014 |
| o LNA-OpenAlkynyl-Diol_8 | -1026.625248 | -1026.173089 | 0.268284 | -1026.337305 | 0.066402 | 0.064092 | -1026.403707 | -1026.401397 | 0.018 |
| o LNA-OpenAlkynyl-Diol_9 | -1026.623870 | -1026.171038 | 0.267851 | -1026.336071 | 0.067858 | 0.064967 | -1026.403929 | -1026.401039 | 0.012 |

**Table S6.** Energies for compound **19**. Electronic energies, enthalpies, entropies, and free energies are presented in atomic units.

| Structure | E_SPC | E | ZPE | H_SPC | T.S | T.qh-S | G(T)_SPC | qh-G(T)_SPC | Boltz |
|---|---|---|---|---|---|---|---|---|---|
| o LNA-OpenmonoCl-Acet_1 | -1566.143244 | -1565.650817 | 0.343002 | -1565.777951 | 0.072470 | 0.069521 | -1565.850421 | -1565.847472 | 0.339 |
| o LNA-OpenmonoCl-Acet_2 | -1566.143007 | -1565.647687 | 0.342637 | -1565.777978 | 0.073269 | 0.069952 | -1565.851247 | -1565.847930 | 0.550 |
| o LNA-OpenmonoCl-Acet_3 | -1566.142853 | -1565.650451 | 0.343547 | -1565.777236 | 0.072104 | 0.069182 | -1565.849340 | -1565.846418 | 0.111 |
| o LNA-OpenmonoCl-Acet_4 | -1566.134497 | -1565.641515 | 0.343067 | -1565.769155 | 0.072324 | 0.069395 | -1565.841479 | -1565.838550 | 0.000 |

**Table S7.** Energies for compound **19’**. Electronic energies, enthalpies, entropies, and free energies are presented in atomic units.

| Structure | E_SPC | E | ZPE | H_SPC | T.S | T.qh-S | G(T)_SPC | qh-G(T)_SPC | Boltz |
|---|---|---|---|---|---|---|---|---|---|
| o LNA-OpenmonoCl-Diol_1 | -1449.423744 | -1448.952948 | 0.279560 | -1449.124434 | 0.066943 | 0.064626 | -1449.191378 | -1449.189060 | 0.101 |
| o LNA-OpenmonoCl-Diol_10 | -1449.421423 | -1448.951828 | 0.278895 | -1449.122482 | 0.068348 | 0.065467 | -1449.190829 | -1449.187949 | 0.031 |
| o LNA-OpenmonoCl-Diol_11 | -1449.421553 | -1448.956235 | 0.279380 | -1449.122411 | 0.067024 | 0.064589 | -1449.189435 | -1449.187001 | 0.011 |
| o LNA-OpenmonoCl-Diol_12 | -1449.418697 | -1448.952123 | 0.279330 | -1449.119636 | 0.066687 | 0.064439 | -1449.186323 | -1449.184075 | 0.001 |
| o LNA-OpenmonoCl-Diol_13 | -1449.421237 | -1448.956786 | 0.279521 | -1449.122206 | 0.066425 | 0.064046 | -1449.188630 | -1449.186252 | 0.005 |
| o LNA-OpenmonoCl-Diol_14 | -1449.419237 | -1448.952049 | 0.278742 | -1449.120262 | 0.068527 | 0.065700 | -1449.188789 | -1449.185962 | 0.004 |
| o LNA-OpenmonoCl-Diol_15 | -1449.422405 | -1448.957039 | 0.279466 | -1449.123274 | 0.067071 | 0.064433 | -1449.190345 | -1449.187708 | 0.024 |
| o LNA-OpenmonoCl-Diol_16 | -1449.421353 | -1448.954443 | 0.278884 | -1449.122458 | 0.068194 | 0.065370 | -1449.190653 | -1449.187828 | 0.027 |
| o LNA-OpenmonoCl-Diol_17 | -1449.421366 | -1448.954442 | 0.278703 | -1449.122599 | 0.068378 | 0.065511 | -1449.190977 | -1449.188110 | 0.037 |
| o LNA-OpenmonoCl-Diol_18 | -1449.420584 | -1448.946694 | 0.278490 | -1449.121838 | 0.068288 | 0.065653 | -1449.190126 | -1449.187491 | 0.019 |
| o LNA-OpenmonoCl-Diol_19 | -1449.421007 | -1448.955902 | 0.279278 | -1449.122008 | 0.066971 | 0.064578 | -1449.188979 | -1449.186586 | 0.007 |
| o LNA-OpenmonoCl-Diol_2 | -1449.423660 | -1448.953213 | 0.279413 | -1449.124473 | 0.067015 | 0.064678 | -1449.191487 | -1449.189151 | 0.111 |
| o LNA-OpenmonoCl-Diol_20 | -1449.422758 | -1448.954416 | 0.279494 | -1449.123590 | 0.066479 | 0.064363 | -1449.190069 | -1449.187953 | 0.031 |
| o LNA-OpenmonoCl-Diol_21 | -1449.415650 | -1448.948788 | 0.279057 | -1449.116662 | 0.068065 | 0.065094 | -1449.184727 | -1449.181756 | 0.000 |
| o LNA-OpenmonoCl-Diol_22 | -1449.420480 | -1448.955334 | 0.279329 | -1449.121449 | 0.067163 | 0.064568 | -1449.188612 | -1449.186017 | 0.004 |
| o LNA-OpenmonoCl-Diol_23 | -1449.420582 | -1448.950502 | 0.279002 | -1449.121813 | 0.067021 | 0.064576 | -1449.188834 | -1449.186389 | 0.006 |
| o LNA-OpenmonoCl-Diol_24 | -1449.424148 | -1448.951286 | 0.278914 | -1449.125241 | 0.067790 | 0.065210 | -1449.193031 | -1449.190451 | 0.441 |
| o LNA-OpenmonoCl-Diol_25 | -1449.418147 | -1448.949686 | 0.278736 | -1449.119554 | 0.068516 | 0.065163 | -1449.188070 | -1449.184717 | 0.001 |
| o LNA-OpenmonoCl-Diol_26 | -1449.416694 | -1448.949928 | 0.279340 | -1449.117474 | 0.067325 | 0.064826 | -1449.184799 | -1449.182300 | 0.000 |
| o LNA-OpenmonoCl-Diol_27 | -1449.418319 | -1448.950323 | 0.278994 | -1449.119539 | 0.067855 | 0.064865 | -1449.187394 | -1449.184404 | 0.001 |
| o LNA-OpenmonoCl-Diol_28 | -1449.418376 | -1448.949559 | 0.279293 | -1449.119153 | 0.067486 | 0.064975 | -1449.186638 | -1449.184127 | 0.001 |
| o LNA-OpenmonoCl-Diol_29 | -1449.419717 | -1448.954475 | 0.279509 | -1449.120593 | 0.067174 | 0.064476 | -1449.187767 | -1449.185069 | 0.001 |
| o LNA-OpenmonoCl-Diol_3 | -1449.423094 | -1448.953109 | 0.279838 | -1449.123780 | 0.066201 | 0.063920 | -1449.189981 | -1449.187700 | 0.024 |
| o LNA-OpenmonoCl-Diol_30 | -1449.417916 | -1448.952696 | 0.279468 | -1449.118645 | 0.067355 | 0.064767 | -1449.186000 | -1449.183412 | 0.000 |
| o LNA-OpenmonoCl-Diol_31 | -1449.417194 | -1448.944010 | 0.278148 | -1449.118554 | 0.069108 | 0.066266 | -1449.187661 | -1449.184820 | 0.001 |
| o LNA-OpenmonoCl-Diol_32 | -1449.421522 | -1448.953106 | 0.279047 | -1449.122644 | 0.067829 | 0.065006 | -1449.190473 | -1449.187650 | 0.023 |
| o LNA-OpenmonoCl-Diol_33 | -1449.416805 | -1448.949466 | 0.278754 | -1449.117775 | 0.068033 | 0.065614 | -1449.185808 | -1449.183389 | 0.000 |
| o LNA-OpenmonoCl-Diol_34 | -1449.421464 | -1448.948922 | 0.279079 | -1449.122421 | 0.067743 | 0.065102 | -1449.190164 | -1449.187523 | 0.020 |
| o LNA-OpenmonoCl-Diol_35 | -1449.415260 | -1448.944268 | 0.278674 | -1449.116495 | 0.068001 | 0.065295 | -1449.184497 | -1449.181790 | 0.000 |
| o LNA-OpenmonoCl-Diol_4 | -1449.421594 | -1448.956241 | 0.279312 | -1449.122503 | 0.067135 | 0.064651 | -1449.189639 | -1449.187154 | 0.013 |
| o LNA-OpenmonoCl-Diol_5 | -1449.421005 | -1448.956161 | 0.279039 | -1449.122136 | 0.067399 | 0.064747 | -1449.189536 | -1449.186883 | 0.010 |
| o LNA-OpenmonoCl-Diol_6 | -1449.421668 | -1448.952708 | 0.279174 | -1449.122514 | 0.067491 | 0.065167 | -1449.190005 | -1449.187682 | 0.024 |

| | | | | | | | | | |
|---|---|---|---|---|---|---|---|---|---|
| o LNA-OpenmonoCl-Diol_7 | -1449.418790 | -1448.951493 | 0.279049 | -1449.119663 | 0.067988 | 0.065346 | -1449.187651 | -1449.185009 | 0.001 |
| o LNA-OpenmonoCl-Diol_8 | -1449.421023 | -1448.955896 | 0.279027 | -1449.122183 | 0.067387 | 0.064835 | -1449.189571 | -1449.187018 | 0.012 |
| o LNA-OpenmonoCl-Diol_9 | -1449.421003 | -1448.956170 | 0.279499 | -1449.121801 | 0.067019 | 0.064465 | -1449.188820 | -1449.186267 | 0.005 |

************************************************************************************************************************************************

**Table S8.** Energies for compound **20**. Electronic energies, enthalpies, entropies, and free energies are presented in atomic units.

| Structure | E_SPC | E | ZPE | H_SPC | T.S | T.qh-S | G(T)_SPC | qh-G(T)_SPC | Boltz |
|---|---|---|---|---|---|---|---|---|---|
| o LNA-AldeFree_1 | -1063.820337 | -1063.347090 | 0.268568 | -1063.532269 | 0.065992 | 0.063850 | -1063.598261 | -1063.596119 | 0.406 |
| o LNA-AldeFree_10 | -1063.817908 | -1063.352368 | 0.269256 | -1063.529551 | 0.065657 | 0.063172 | -1063.595208 | -1063.592722 | 0.011 |
| o LNA-AldeFree_11 | -1063.817180 | -1063.350061 | 0.268343 | -1063.529357 | 0.066168 | 0.063810 | -1063.595525 | -1063.593168 | 0.018 |
| o LNA-AldeFree_12 | -1063.814145 | -1063.343519 | 0.268247 | -1063.526283 | 0.066773 | 0.064273 | -1063.593056 | -1063.590556 | 0.001 |
| o LNA-AldeFree_13 | -1063.817209 | -1063.350051 | 0.268250 | -1063.529458 | 0.066208 | 0.063853 | -1063.595667 | -1063.593312 | 0.021 |
| o LNA-AldeFree_14 | -1063.817134 | -1063.349548 | 0.268845 | -1063.529141 | 0.065118 | 0.063079 | -1063.594260 | -1063.592220 | 0.007 |
| o LNA-AldeFree_15 | -1063.815195 | -1063.349877 | 0.268986 | -1063.526852 | 0.066065 | 0.063580 | -1063.592916 | -1063.590431 | 0.001 |
| o LNA-AldeFree_2 | -1063.817282 | -1063.352305 | 0.269194 | -1063.528969 | 0.065248 | 0.063026 | -1063.594217 | -1063.591995 | 0.005 |
| o LNA-AldeFree_3 | -1063.819811 | -1063.350750 | 0.269076 | -1063.531520 | 0.065321 | 0.063275 | -1063.596841 | -1063.594795 | 0.100 |
| o LNA-AldeFree_4 | -1063.817267 | -1063.350060 | 0.268289 | -1063.529504 | 0.066159 | 0.063812 | -1063.595663 | -1063.593316 | 0.021 |
| o LNA-AldeFree_5 | -1063.815553 | -1063.347229 | 0.268849 | -1063.527335 | 0.066317 | 0.063763 | -1063.593652 | -1063.591098 | 0.002 |
| o LNA-AldeFree_6 | -1063.817111 | -1063.349547 | 0.268750 | -1063.529183 | 0.065253 | 0.063150 | -1063.594435 | -1063.592333 | 0.007 |
| o LNA-AldeFree_7 | -1063.817964 | -1063.352370 | 0.269238 | -1063.529607 | 0.065739 | 0.063223 | -1063.595346 | -1063.592830 | 0.012 |
| o LNA-AldeFree_8 | -1063.815180 | -1063.349881 | 0.268985 | -1063.526835 | 0.066000 | 0.063567 | -1063.592835 | -1063.590403 | 0.001 |
| o LNA-AldeFree_9 | -1063.820505 | -1063.344608 | 0.268692 | -1063.532378 | 0.065935 | 0.063697 | -1063.598313 | -1063.596075 | 0.387 |

************************************************************************************************************************************************

**Table S9.** Energies for compound **21**. Electronic energies, enthalpies, entropies, and free energies are presented in atomic units.

| Structure | E_SPC | E | ZPE | H_SPC | T.S | T.qh-S | G(T)_SPC | qh-G(T)_SPC | Boltz |
|---|---|---|---|---|---|---|---|---|---|
| o LNA-OH-Free_1 | -1063.816895 | -1063.347693 | 0.271000 | -1063.527426 | 0.063663 | 0.061692 | -1063.591089 | -1063.589118 | 0.298 |
| o LNA-OH-Free_10 | -1063.812989 | -1063.341532 | 0.270404 | -1063.523720 | 0.064491 | 0.062520 | -1063.588211 | -1063.586240 | 0.014 |
| o LNA-OH-Free_11 | -1063.811014 | -1063.341837 | 0.271070 | -1063.521499 | 0.063711 | 0.061752 | -1063.585210 | -1063.583250 | 0.001 |
| o LNA-OH-Free_12 | -1063.815262 | -1063.339893 | 0.270809 | -1063.525843 | 0.064311 | 0.062129 | -1063.590154 | -1063.587972 | 0.088 |
| o LNA-OH-Free_13 | -1063.811548 | -1063.340144 | 0.270261 | -1063.522569 | 0.064391 | 0.062261 | -1063.586961 | -1063.584831 | 0.003 |
| o LNA-OH-Free_14 | -1063.814871 | -1063.342276 | 0.270845 | -1063.525555 | 0.063687 | 0.061721 | -1063.589242 | -1063.587276 | 0.042 |
| o LNA-OH-Free_15 | -1063.813815 | -1063.340323 | 0.270891 | -1063.524378 | 0.064098 | 0.061990 | -1063.588476 | -1063.586368 | 0.016 |
| o LNA-OH-Free_16 | -1063.810064 | -1063.338148 | 0.270240 | -1063.520971 | 0.064778 | 0.062673 | -1063.585749 | -1063.583645 | 0.001 |
| o LNA-OH-Free_17 | -1063.810396 | -1063.341113 | 0.271240 | -1063.520779 | 0.063790 | 0.061706 | -1063.584569 | -1063.582485 | 0.000 |
| o LNA-OH-Free_18 | -1063.812680 | -1063.342462 | 0.271344 | -1063.523090 | 0.063093 | 0.061299 | -1063.586184 | -1063.584390 | 0.002 |
| o LNA-OH-Free_19 | -1063.809511 | -1063.337521 | 0.270212 | -1063.520383 | 0.065105 | 0.062815 | -1063.585487 | -1063.583198 | 0.001 |
| o LNA-OH-Free_2 | -1063.815080 | -1063.345859 | 0.270951 | -1063.525693 | 0.063752 | 0.061710 | -1063.589445 | -1063.587403 | 0.048 |
| o LNA-OH-Free_20 | -1063.811669 | -1063.340575 | 0.270315 | -1063.522540 | 0.064480 | 0.062493 | -1063.587020 | -1063.585033 | 0.004 |
| o LNA-OH-Free_3 | -1063.815595 | -1063.344606 | 0.270588 | -1063.526391 | 0.064184 | 0.062080 | -1063.590575 | -1063.588471 | 0.150 |
| o LNA-OH-Free_4 | -1063.816140 | -1063.345725 | 0.270710 | -1063.526721 | 0.064161 | 0.062199 | -1063.590882 | -1063.588920 | 0.242 |

| | | | | | | | | | |
|---|---|---|---|---|---|---|---|---|---|
| o LNA-OH-Free_5 | -1063.810254 | -1063.341472 | 0.270943 | -1063.520815 | 0.063808 | 0.061862 | -1063.584624 | -1063.582677 | 0.000 |
| o LNA-OH-Free_6 | -1063.812045 | -1063.342276 | 0.270655 | -1063.522795 | 0.064233 | 0.061977 | -1063.587029 | -1063.584772 | 0.003 |
| o LNA-OH-Free_7 | -1063.812388 | -1063.342169 | 0.270745 | -1063.523024 | 0.064108 | 0.062106 | -1063.587132 | -1063.585130 | 0.004 |
| o LNA-OH-Free_8 | -1063.812494 | -1063.340825 | 0.270366 | -1063.523397 | 0.064388 | 0.062275 | -1063.587784 | -1063.585672 | 0.008 |
| o LNA-OH-Free_9 | -1063.814711 | -1063.343898 | 0.270454 | -1063.525582 | 0.064233 | 0.062225 | -1063.589815 | -1063.587807 | 0.074 |

***************************************************************************************************************************************

**Table S10.** Energies for compound **22**. Electronic energies, enthalpies, entropies, and free energies are presented in atomic units.

| Structure | E_SPC | E | ZPE | H_SPC | T.S | T.qh-S | G(T)_SPC | qh-G(T)_SPC | Boltz |
|---|---|---|---|---|---|---|---|---|---|
| o LNA-AldeAcet_1 | -1180.539412 | -1180.043947 | 0.332337 | -1180.185193 | 0.070815 | 0.068387 | -1180.256009 | -1180.253580 | 0.264 |
| o LNA-AldeAcet_10 | -1180.533307 | -1180.039871 | 0.332020 | -1180.179516 | 0.070914 | 0.068215 | -1180.250430 | -1180.247731 | 0.001 |
| o LNA-AldeAcet_2 | -1180.534387 | -1180.039433 | 0.332419 | -1180.179997 | 0.071461 | 0.068608 | -1180.251458 | -1180.248605 | 0.001 |
| o LNA-AldeAcet_3 | -1180.538457 | -1180.041831 | 0.332114 | -1180.184443 | 0.071394 | 0.068678 | -1180.255837 | -1180.253121 | 0.162 |
| o LNA-AldeAcet_4 | -1180.538696 | -1180.044108 | 0.332752 | -1180.184243 | 0.070735 | 0.068040 | -1180.254978 | -1180.252283 | 0.067 |
| o LNA-AldeAcet_5 | -1180.536655 | -1180.041022 | 0.331965 | -1180.182969 | 0.070482 | 0.068014 | -1180.253451 | -1180.250984 | 0.017 |
| o LNA-AldeAcet_6 | -1180.534399 | -1180.038052 | 0.332071 | -1180.180320 | 0.072184 | 0.068978 | -1180.252505 | -1180.249299 | 0.003 |
| o LNA-AldeAcet_7 | -1180.538733 | -1180.044116 | 0.332987 | -1180.184113 | 0.070550 | 0.067877 | -1180.254664 | -1180.251990 | 0.049 |
| o LNA-AldeAcet_8 | -1180.539910 | -1180.040672 | 0.332455 | -1180.185688 | 0.071237 | 0.068367 | -1180.256925 | -1180.254055 | 0.436 |
| o LNA-AldeAcet_9 | -1180.531149 | -1180.038729 | 0.332228 | -1180.177171 | 0.070576 | 0.068108 | -1180.247746 | -1180.245278 | 0.000 |

***************************************************************************************************************************************

**Table S11.** Energies for compound **23**. Electronic energies, enthalpies, entropies, and free energies are presented in atomic units.

| Structure | E_SPC | E | ZPE | H_SPC | T.S | T.qh-S | G(T)_SPC | qh-G(T)_SPC | Boltz |
|---|---|---|---|---|---|---|---|---|---|
| o LNA-OHAcet_1 | -1180.546976 | -1180.053239 | 0.334999 | -1180.191378 | 0.068171 | 0.065854 | -1180.259549 | -1180.257232 | 0.880 |
| o LNA-OHAcet_2 | -1180.539738 | -1180.046441 | 0.334841 | -1180.184173 | 0.068456 | 0.066145 | -1180.252630 | -1180.250318 | 0.001 |
| o LNA-OHAcet_3 | -1180.545010 | -1180.047857 | 0.335066 | -1180.189349 | 0.068290 | 0.065923 | -1180.257639 | -1180.255271 | 0.110 |
| o LNA-OHAcet_4 | -1180.539553 | -1180.046783 | 0.335342 | -1180.183734 | 0.067929 | 0.065590 | -1180.251663 | -1180.249324 | 0.000 |
| o LNA-OHAcet_5 | -1180.541382 | -1180.048033 | 0.334517 | -1180.186163 | 0.068576 | 0.066071 | -1180.254740 | -1180.252235 | 0.004 |
| o LNA-OHAcet_6 | -1180.541184 | -1180.042438 | 0.334691 | -1180.185679 | 0.069296 | 0.066591 | -1180.254975 | -1180.252270 | 0.005 |
| o LNA-OHAcet_7 | -1180.538077 | -1180.043813 | 0.334253 | -1180.182983 | 0.068825 | 0.066339 | -1180.251808 | -1180.249321 | 0.000 |
| o LNA-OHAcet_8 | -1180.536417 | -1180.043534 | 0.334730 | -1180.181026 | 0.068706 | 0.066145 | -1180.249731 | -1180.247171 | 0.000 |

***************************************************************************************************************************************

## Cartesian coordinates for optimized structures

### Cartesian coordinates for compound 34

LNA-LockedmonoCl-Diol_1
Eopt -988.144290

| | | | |
|---|---|---|---|
| C | 1.708200 | -0.622200 | 4.976200 |
| C | 1.690500 | -2.146300 | 4.741200 |
| O | 0.699000 | -2.281000 | 3.747000 |
| C | 0.156400 | -0.972600 | 3.509400 |
| C | 1.425700 | -0.141300 | 3.564000 |
| N | 2.950300 | -2.798500 | 4.376900 |
| C | 3.943800 | -2.811000 | 5.339100 |
| N | 5.154500 | -3.250200 | 4.894100 |
| C | 5.541900 | -3.630500 | 3.650700 |
| C | 4.465400 | -3.559100 | 2.657600 |
| C | 3.246200 | -3.148100 | 3.071600 |
| C | 4.775600 | -3.953900 | 1.218500 |
| O | 6.698400 | -3.985500 | 3.442200 |
| O | 3.824300 | -2.451300 | 6.508400 |
| C | -0.629600 | -0.944100 | 2.184800 |
| O | -1.766900 | -1.782700 | 2.277900 |
| O | 1.282600 | 1.267500 | 3.463800 |
| C | -0.607900 | -0.536800 | 4.774900 |
| O | 0.492900 | -0.239800 | 5.650800 |
| H | 2.626100 | -0.265000 | 5.448300 |
| H | 1.366300 | -2.678600 | 5.642400 |
| H | 2.170700 | -0.483200 | 2.840600 |
| H | 5.882200 | -3.278200 | 5.587400 |
| H | 2.411500 | -3.088800 | 2.388300 |
| H | 3.907400 | -3.871000 | 0.564500 |
| H | 5.564800 | -3.318800 | 0.811600 |
| H | 5.130400 | -4.985400 | 1.175500 |
| H | -0.950700 | 0.072800 | 1.952000 |
| H | 0.003100 | -1.274000 | 1.359200 |
| H | -1.460200 | -2.669500 | 2.496100 |
| H | 0.879200 | 1.571200 | 4.285800 |
| H | -1.219800 | 0.351000 | 4.608900 |
| H | -1.244700 | -1.331100 | 5.167100 |

LNA-LockedmonoCl-Diol_10
Eopt -988.139733

| | | | |
|---|---|---|---|
| C | 1.700900 | -0.707000 | 4.962700 |
| C | 1.754900 | -2.233600 | 4.753100 |
| O | 0.778700 | -2.424900 | 3.755600 |
| C | 0.181000 | -1.149700 | 3.480000 |
| C | 1.413300 | -0.263700 | 3.539800 |
| N | 3.042800 | -2.838400 | 4.404800 |
| C | 4.044900 | -2.755400 | 5.354300 |
| N | 5.273300 | -3.147500 | 4.913700 |
| C | 5.668300 | -3.561800 | 3.683700 |
| C | 4.578500 | -3.599400 | 2.703500 |
| C | 3.343100 | -3.236100 | 3.114000 |
| C | 4.893100 | -4.051100 | 1.282200 |
| O | 6.841700 | -3.857800 | 3.476100 |
| O | 3.919100 | -2.350600 | 6.508400 |
| C | -0.574500 | -1.160100 | 2.130500 |
| O | -1.792000 | -1.889000 | 2.189200 |
| O | 1.206700 | 1.136700 | 3.419600 |
| C | -0.613500 | -0.732600 | 4.732600 |
| O | 0.460800 | -0.365000 | 5.614400 |
| H | 2.596100 | -0.300000 | 5.437800 |
| H | 1.449600 | -2.768400 | 5.659000 |
| H | 2.180900 | -0.584000 | 2.829800 |
| H | 6.008500 | -3.103900 | 5.597900 |
| H | 2.496900 | -3.261900 | 2.441400 |
| H | 4.011900 | -4.054700 | 0.640300 |
| H | 5.636900 | -3.392900 | 0.829400 |
| H | 5.307400 | -5.061000 | 1.287300 |
| H | -0.788700 | -0.139000 | 1.809900 |
| H | 0.061000 | -1.598400 | 1.359200 |
| H | -2.487500 | -1.337700 | 2.555300 |
| H | 0.836500 | 1.438800 | 4.257600 |
| H | -1.266100 | 0.122300 | 4.552400 |
| H | -1.208600 | -1.555200 | 5.132800 |

LNA-LockedmonoCl-Diol_11
Eopt -988.139279

| | | | |
|---|---|---|---|
| C | 1.307600 | -0.932500 | 5.425900 |
| C | 1.603300 | -2.297600 | 4.762600 |
| O | 0.622400 | -2.339600 | 3.752700 |
| C | -0.128600 | -1.114000 | 3.826400 |
| C | 0.953500 | -0.115300 | 4.187500 |
| N | 2.900400 | -2.657000 | 4.182100 |
| C | 4.001700 | -1.820500 | 4.271500 |
| N | 5.165500 | -2.379500 | 3.833800 |
| C | 5.413100 | -3.620400 | 3.344600 |
| C | 4.219600 | -4.468100 | 3.278400 |
| C | 3.048000 | -3.943200 | 3.699200 |
| C | 4.360400 | -5.885100 | 2.735200 |
| O | 6.549800 | -3.940500 | 3.008900 |
| O | 4.047300 | -0.666000 | 4.688300 |
| C | -0.907700 | -0.877300 | 2.516500 |
| O | -0.040700 | -0.969400 | 1.399800 |
| O | 0.535000 | 1.223300 | 4.439000 |
| C | -0.975000 | -1.168100 | 5.111000 |
| O | 0.047800 | -1.052900 | 6.111600 |
| H | 2.105200 | -0.566900 | 6.072600 |
| H | 1.441800 | -3.083000 | 5.506000 |
| H | 1.748600 | -0.109000 | 3.437000 |
| H | 5.972500 | -1.782400 | 3.887100 |
| H | 2.121100 | -4.503100 | 3.664400 |
| H | 3.412700 | -6.423600 | 2.719500 |
| H | 4.751100 | -5.864400 | 1.716000 |
| H | 5.062700 | -6.457600 | 3.343900 |
| H | -1.699100 | -1.620800 | 2.411900 |
| H | -1.386200 | 0.103700 | 2.524600 |
| H | 0.456900 | -1.787900 | 1.496600 |
| H | 1.290100 | 1.725600 | 4.759300 |
| H | -1.664500 | -0.325300 | 5.185100 |
| H | -1.537500 | -2.097300 | 5.206300 |

LNA-LockedmonoCl-Diol_12
Eopt -988.139168

| | | | |
|---|---|---|---|
| C | 1.647100 | -0.737100 | 5.168500 |
| C | 1.655300 | -2.233800 | 4.799300 |
| O | 0.679600 | -2.286800 | 3.780100 |
| C | 0.142200 | -0.961000 | 3.636900 |
| C | 1.411000 | -0.139700 | 3.786600 |
| N | 2.927200 | -2.842300 | 4.400800 |
| C | 3.916100 | -2.905000 | 5.365900 |
| N | 5.139800 | -3.277900 | 4.896800 |
| C | 5.541500 | -3.558100 | 3.631600 |
| C | 4.465300 | -3.451200 | 2.641400 |
| C | 3.235000 | -3.099900 | 3.076900 |
| C | 4.787400 | -3.748200 | 1.181400 |
| O | 6.708700 | -3.861500 | 3.402100 |
| O | 3.784100 | -2.642700 | 6.559100 |
| C | -0.615500 | -0.840700 | 2.301100 |
| O | -1.738500 | -1.701200 | 2.298800 |
| O | 1.291500 | 1.279900 | 3.753100 |
| C | -0.651100 | -0.610000 | 4.911700 |
| O | 0.414900 | -0.466700 | 5.859800 |
| H | 2.541900 | -0.411600 | 5.702300 |
| H | 1.323700 | -2.845900 | 5.645400 |
| H | 2.183500 | -0.444200 | 3.075400 |
| H | 5.864600 | -3.335900 | 5.591200 |
| H | 2.399300 | -3.024000 | 2.396900 |
| H | 3.917200 | -3.653600 | 0.531900 |
| H | 5.557500 | -3.066400 | 0.815500 |
| H | 5.173800 | -4.764000 | 1.077400 |
| H | -0.953200 | 0.184300 | 2.139800 |
| H | 0.039600 | -1.099200 | 1.467700 |
| H | -1.430700 | -2.585700 | 2.527100 |
| H | 1.159200 | 1.584000 | 2.851800 |
| H | -1.194700 | 0.331000 | 4.815500 |
| H | -1.352200 | -1.394400 | 5.198600 |

LNA-LockedmonoCl-Diol_13
Eopt -988.145332

| | | | |
|---|---|---|---|
| C | 1.898000 | -0.049800 | 4.482700 |
| C | 2.034000 | -1.526400 | 4.893100 |
| O | 0.810200 | -2.074300 | 4.474600 |
| C | -0.010600 | -0.979900 | 4.030600 |
| C | 1.013900 | -0.181800 | 3.247000 |
| N | 3.168700 | -2.339800 | 4.448800 |
| C | 3.442900 | -3.491600 | 5.168200 |
| N | 4.416100 | -4.275400 | 4.625300 |
| C | 5.149800 | -4.103300 | 3.497800 |
| C | 4.821400 | -2.872500 | 2.772100 |
| C | 3.860300 | -2.070300 | 3.281900 |
| C | 5.579300 | -2.563000 | 1.486100 |
| O | 5.994400 | -4.932800 | 3.172300 |
| O | 2.909000 | -3.852400 | 6.215600 |
| C | -1.241300 | -1.515600 | 3.269900 |
| O | -0.842800 | -2.422500 | 2.258100 |
| O | 0.619700 | 1.078800 | 2.722300 |
| C | -0.302400 | -0.070900 | 5.242500 |
| O | 0.973200 | 0.584000 | 5.390700 |
| H | 2.853900 | 0.464800 | 4.371100 |
| H | 2.082700 | -1.558600 | 5.988500 |
| H | 1.436100 | -0.791500 | 2.445600 |
| H | 4.622600 | -5.114600 | 5.138600 |
| H | 3.592700 | -1.152900 | 2.785200 |
| H | 5.259400 | -1.629500 | 1.022900 |
| H | 6.650600 | -2.491100 | 1.683200 |
| H | 5.433700 | -3.363800 | 0.758500 |
| H | -1.914000 | -2.028800 | 3.958900 |
| H | -1.803800 | -0.696400 | 2.818800 |
| H | -0.292500 | -3.092300 | 2.680100 |
| H | 0.468800 | 1.666200 | 3.471300 |
| H | -1.086300 | 0.660800 | 5.041900 |
| H | -0.574200 | -0.644100 | 6.130700 |

LNA-LockedmonoCl-Diol_14
Eopt -988.145645

| | | | |
|---|---|---|---|
| C | 1.834600 | -0.265900 | 4.306000 |
| C | 2.146300 | -1.666100 | 4.863600 |
| O | 0.991700 | -2.391600 | 4.523400 |
| C | 0.027200 | -1.445000 | 4.039200 |
| C | 0.935500 | -0.637400 | 3.131700 |
| N | 3.365800 | -2.383400 | 4.483300 |
| C | 3.798500 | -3.394700 | 5.325800 |
| N | 4.848600 | -4.116900 | 4.844400 |
| C | 5.531800 | -3.996200 | 3.679000 |
| C | 5.039900 | -2.910000 | 2.826000 |

| | | | |
|---|---|---|---|
| C | 3.996700 | -2.174500 | 3.270600 |
| C | 5.730300 | -2.666700 | 1.488700 |
| O | 6.465200 | -4.751500 | 3.422400 |
| O | 3.341200 | -3.682800 | 6.430300 |
| C | -1.157500 | -2.173700 | 3.372000 |
| O | -2.022300 | -1.250500 | 2.731200 |
| O | 0.366700 | 0.484700 | 2.469600 |
| C | -0.319900 | -0.452600 | 5.170500 |
| O | 0.870700 | 0.361200 | 5.175400 |
| H | 2.722500 | 0.334000 | 4.099700 |
| H | 2.202000 | -1.579600 | 5.955800 |
| H | 1.408700 | -1.286200 | 2.391400 |
| H | 5.167600 | -4.856100 | 5.446200 |
| H | 3.608000 | -1.362000 | 2.680600 |
| H | 5.292300 | -1.838000 | 0.932300 |
| H | 6.788700 | -2.447000 | 1.641500 |
| H | 5.669300 | -3.559500 | 0.863100 |
| H | -0.789300 | -2.890300 | 2.635800 |
| H | -1.718000 | -2.745600 | 4.113200 |
| H | -1.491700 | -0.713600 | 2.127100 |
| H | 0.171100 | 1.141400 | 3.150800 |
| H | -1.199000 | 0.153800 | 4.946900 |
| H | -0.478300 | -0.959100 | 6.124100 |

LNA-LockedmonoCl-Diol_15
Eopt -988.139457

| | | | |
|---|---|---|---|
| C | 1.672700 | -0.798400 | 5.153100 |
| C | 1.760200 | -2.167600 | 4.463600 |
| O | 0.805100 | -2.082200 | 3.440400 |
| C | 0.112700 | -0.835000 | 3.647700 |
| C | 1.271200 | 0.088100 | 3.985400 |
| N | 3.057700 | -2.714000 | 4.060100 |
| C | 3.634000 | -2.330700 | 2.859800 |
| N | 4.850100 | -2.896300 | 2.618900 |
| C | 5.580100 | -3.760700 | 3.366900 |
| C | 4.933300 | -4.125000 | 4.630600 |
| C | 3.723900 | -3.587100 | 4.902700 |
| C | 5.658900 | -5.081700 | 5.569800 |
| O | 6.671400 | -4.160200 | 2.971000 |
| O | 3.188200 | -1.566400 | 2.008900 |
| C | -0.709200 | -0.466800 | 2.399200 |
| O | -1.729200 | -1.428200 | 2.197000 |
| O | 0.955000 | 1.418500 | 4.378500 |
| C | -0.648900 | -0.908900 | 4.990000 |
| O | 0.449500 | -0.778100 | 5.921000 |
| H | 2.580900 | -0.522300 | 5.691600 |
| H | 1.359600 | -2.930200 | 5.135300 |
| H | 2.012800 | 0.128500 | 3.189500 |
| H | 5.273800 | -2.632200 | 1.746300 |
| H | 3.206900 | -3.814100 | 5.825500 |
| H | 5.099000 | -5.280800 | 6.483600 |
| H | 5.839400 | -6.036600 | 5.072400 |
| H | 6.630500 | -4.672700 | 5.853600 |
| H | -1.157900 | 0.521500 | 2.511900 |
| H | -0.062400 | -0.425800 | 1.520600 |
| H | -1.290200 | -2.269200 | 2.027600 |
| H | 0.524000 | 1.364100 | 5.238600 |
| H | -1.360100 | -0.090400 | 5.110500 |
| H | -1.183900 | -1.852600 | 5.105300 |

LNA-LockedmonoCl-Diol_16
Eopt -988.140927

| | | | |
|---|---|---|---|
| C | 1.956100 | -0.166600 | 4.478800 |
| C | 2.076500 | -1.674600 | 4.760300 |
| O | 0.960200 | -2.198500 | 4.088200 |
| C | 0.147600 | -1.081600 | 3.686200 |
| C | 1.245500 | -0.197900 | 3.129400 |
| N | 3.299200 | -2.413200 | 4.434400 |
| C | 3.535000 | -3.594800 | 5.118600 |
| N | 4.608100 | -4.301800 | 4.666000 |
| C | 5.477400 | -4.025700 | 3.662200 |
| C | 5.191800 | -2.761600 | 2.976900 |
| C | 4.131300 | -2.036200 | 3.396200 |
| C | 6.104400 | -2.332400 | 1.833500 |
| O | 6.396700 | -4.797400 | 3.403200 |
| O | 2.894000 | -4.043200 | 6.067100 |
| C | -0.949100 | -1.522500 | 2.691400 |
| O | -2.075400 | -2.069200 | 3.361200 |
| O | 0.879600 | 1.091000 | 2.658600 |
| C | -0.319300 | -0.308300 | 4.939500 |
| O | 0.902300 | 0.365500 | 5.305900 |
| H | 2.903700 | 0.370400 | 4.543400 |
| H | 1.935600 | -1.820600 | 5.838200 |
| H | 1.793200 | -0.721400 | 2.343200 |
| H | 4.783600 | -5.163800 | 5.152300 |
| H | 3.888500 | -1.097900 | 2.927600 |
| H | 5.808400 | -1.380400 | 1.392900 |
| H | 7.133500 | -2.234900 | 2.184600 |
| H | 6.098900 | -3.084200 | 1.042000 |
| H | -1.284900 | -0.668800 | 2.100100 |
| H | -0.542200 | -2.249900 | 1.986900 |
| H | -2.576900 | -2.613100 | 2.747200 |
| H | 0.585200 | 1.596400 | 3.426000 |
| H | -1.109200 | 0.410300 | 4.717900 |
| H | -0.673400 | -0.984000 | 5.720100 |

LNA-LockedmonoCl-Diol_2
Eopt -988.145653

| | | | |
|---|---|---|---|
| C | 1.620200 | -0.770100 | 4.913100 |
| C | 1.778100 | -2.304500 | 4.924700 |
| O | 0.714400 | -2.715300 | 4.094100 |
| C | -0.015000 | -1.539100 | 3.719500 |
| C | 1.136700 | -0.574800 | 3.487400 |
| N | 3.066700 | -2.867500 | 4.514500 |
| C | 4.153300 | -2.583900 | 5.321900 |
| N | 5.356100 | -2.955800 | 4.800700 |
| C | 5.651600 | -3.516400 | 3.601000 |
| C | 4.471600 | -3.762000 | 2.766000 |
| C | 3.260900 | -3.423800 | 3.262900 |
| C | 4.670900 | -4.388000 | 1.390600 |
| O | 6.816000 | -3.765100 | 3.302200 |
| O | 4.116400 | -2.025800 | 6.416600 |
| C | -0.932300 | -1.825800 | 2.513100 |
| O | -1.548700 | -0.635000 | 2.054400 |
| O | 0.793200 | 0.769000 | 3.174500 |
| C | -0.695900 | -0.989700 | 4.988400 |
| O | 0.439100 | -0.416100 | 5.659900 |
| H | 2.526000 | -0.235900 | 5.208300 |
| H | 1.622200 | -2.708500 | 5.931200 |
| H | 1.842900 | -0.953500 | 2.743300 |
| H | 6.153200 | -2.767600 | 5.383700 |
| H | 2.354800 | -3.597000 | 2.700300 |
| H | 3.731600 | -4.536000 | 0.857600 |
| H | 5.311800 | -3.755300 | 0.773800 |
| H | 5.160300 | -5.359500 | 1.482800 |
| H | -0.352900 | -2.264200 | 1.699000 |
| H | -1.700100 | -2.553400 | 2.781000 |
| H | -0.857400 | 0.031600 | 1.932800 |
| H | 0.474400 | 1.172400 | 3.992600 |
| H | -1.439600 | -0.221600 | 4.770100 |
| H | -1.168700 | -1.776600 | 5.577900 |

LNA-LockedmonoCl-Diol_3
Eopt -988.140926

| | | | |
|---|---|---|---|
| C | 1.752600 | -0.649300 | 4.987100 |
| C | 1.779000 | -2.181500 | 4.813300 |
| O | 0.769900 | -2.384100 | 3.849900 |
| C | 0.168400 | -1.108300 | 3.580300 |
| C | 1.411900 | -0.236400 | 3.566500 |
| N | 3.048100 | -2.811900 | 4.443300 |
| C | 4.075800 | -2.727600 | 5.365000 |
| N | 5.286300 | -3.146500 | 4.900200 |
| C | 5.642900 | -3.588700 | 3.668100 |
| C | 4.527700 | -3.625500 | 2.716800 |
| C | 3.309000 | -3.235800 | 3.152500 |
| C | 4.798700 | -4.105600 | 1.295700 |
| O | 6.805400 | -3.907700 | 3.435200 |
| O | 3.987200 | -2.301200 | 6.514600 |
| C | -0.657200 | -1.136600 | 2.274900 |
| O | -1.917700 | -1.763000 | 2.462500 |
| O | 1.219400 | 1.162700 | 3.417400 |
| C | -0.568800 | -0.655300 | 4.855600 |
| O | 0.545900 | -0.276000 | 5.681800 |
| H | 2.671100 | -0.242400 | 5.415100 |
| H | 1.492600 | -2.688500 | 5.741100 |
| H | 2.146500 | -0.583900 | 2.835100 |
| H | 6.039300 | -3.102200 | 5.564700 |
| H | 2.445500 | -3.258500 | 2.503200 |
| H | 3.901900 | -4.105400 | 0.676100 |
| H | 5.541500 | -3.467200 | 0.813900 |
| H | 5.197300 | -5.121800 | 1.307800 |
| H | -0.828900 | -0.118100 | 1.921800 |
| H | -0.098600 | -1.649300 | 1.490300 |
| H | -2.343000 | -1.886700 | 1.609200 |
| H | 0.828700 | 1.479200 | 4.240900 |
| H | -1.226800 | 0.196100 | 4.678000 |
| H | -1.155600 | -1.463600 | 5.295100 |

LNA-LockedmonoCl-Diol_4
Eopt -988.144502

| | | | |
|---|---|---|---|
| C | 1.648900 | -0.596100 | 5.046100 |
| C | 1.719000 | -2.137200 | 5.076600 |
| O | 0.573800 | -2.498600 | 4.341700 |
| C | -0.072900 | -1.288400 | 3.927000 |
| C | 1.124000 | -0.396400 | 3.636800 |
| N | 2.932500 | -2.780800 | 4.567700 |
| C | 4.094500 | -2.570400 | 5.286800 |
| N | 5.225100 | -3.023900 | 4.676100 |
| C | 5.388700 | -3.600300 | 3.458800 |
| C | 4.134700 | -3.759500 | 2.715900 |
| C | 2.991500 | -3.341900 | 3.303700 |
| C | 4.185200 | -4.383800 | 1.326500 |
| O | 6.507700 | -3.927900 | 3.073700 |
| O | 4.180500 | -2.009000 | 6.377500 |
| C | -1.063500 | -1.555100 | 2.769700 |
| O | -0.398400 | -1.790200 | 1.537600 |
| O | 0.848200 | 0.957700 | 3.307600 |
| C | -0.672300 | -0.665900 | 5.202000 |
| O | 0.518600 | -0.158300 | 5.827700 |
| H | 2.596800 | -0.114800 | 5.296600 |
| H | 1.621700 | -2.519100 | 6.098600 |
| H | 1.782200 | -0.828600 | 2.878600 |
| H | 6.075800 | -2.892200 | 5.195200 |
| H | 2.034700 | -3.443000 | 2.806900 |
| H | 3.199900 | -4.449300 | 0.863700 |
| H | 4.827400 | -3.796900 | 0.667400 |
| H | 4.599200 | -5.392400 | 1.377800 |
| H | -1.697900 | -2.410100 | 3.009100 |

| | | | |
|---|---|---|---|
| H | -1.725800 | -0.698100 | 2.637300 |
| H | -0.003000 | -0.962800 | 1.242700 |
| H | 0.607300 | 1.403100 | 4.128900 |
| H | -1.365000 | 0.149600 | 4.989800 |
| H | -1.178500 | -1.404300 | 5.826200 |

LNA-LockedmonoCl-Diol_5
Eopt -988.139458

| | | | |
|---|---|---|---|
| C | 1.435200 | -0.888900 | 5.316600 |
| C | 1.723300 | -2.210800 | 4.562000 |
| O | 0.898100 | -2.076800 | 3.428900 |
| C | 0.180800 | -0.839500 | 3.556600 |
| C | 1.261800 | 0.053300 | 4.137100 |
| N | 3.058000 | -2.630400 | 4.124600 |
| C | 4.197100 | -1.911100 | 4.446900 |
| N | 5.364100 | -2.528400 | 4.107200 |
| C | 5.581700 | -3.734300 | 3.525100 |
| C | 4.347300 | -4.460300 | 3.214100 |
| C | 3.172600 | -3.873400 | 3.531300 |
| C | 4.452900 | -5.826000 | 2.545800 |
| O | 6.725800 | -4.123100 | 3.308000 |
| O | 4.275000 | -0.813800 | 4.990300 |
| C | -0.408000 | -0.407100 | 2.200200 |
| O | -1.378600 | -1.345000 | 1.770000 |
| O | 0.873000 | 1.368800 | 4.509100 |
| C | -0.813200 | -0.994100 | 4.722000 |
| O | 0.091300 | -0.945900 | 5.838700 |
| H | 2.175100 | -0.626600 | 6.073600 |
| H | 1.402100 | -3.043700 | 5.193500 |
| H | 2.145100 | 0.096600 | 3.493600 |
| H | 6.198400 | -2.013600 | 4.330000 |
| H | 2.219800 | -4.340700 | 3.314400 |
| H | 3.478100 | -6.272100 | 2.348400 |
| H | 4.982800 | -5.743800 | 1.594900 |
| H | 5.017100 | -6.515400 | 3.176800 |
| H | -0.870300 | 0.578300 | 2.281500 |
| H | 0.382000 | -0.326000 | 1.451700 |
| H | -0.934900 | -2.193600 | 1.670300 |
| H | 0.367200 | 1.294600 | 5.325900 |
| H | -1.529700 | -0.172700 | 4.769100 |
| H | -1.363600 | -1.934700 | 4.674300 |

LNA-LockedmonoCl-Diol_6
Eopt -988.138471

| | | | |
|---|---|---|---|
| C | 1.913500 | -0.139200 | 4.506400 |
| C | 1.996700 | -1.659100 | 4.736300 |
| O | 0.880500 | -2.135200 | 4.027700 |
| C | 0.116000 | -0.985300 | 3.629900 |
| C | 1.248400 | -0.109300 | 3.134600 |
| N | 3.206500 | -2.414400 | 4.402600 |
| C | 3.363000 | -3.655600 | 4.997800 |
| N | 4.429900 | -4.368300 | 4.539500 |
| C | 5.352200 | -4.052700 | 3.596400 |
| C | 5.142200 | -2.731800 | 2.996000 |
| C | 4.092900 | -1.996500 | 3.426900 |
| C | 6.115600 | -2.258500 | 1.922500 |
| O | 6.253600 | -4.838800 | 3.319000 |
| O | 2.653800 | -4.149700 | 5.872500 |
| C | -0.953700 | -1.388200 | 2.597800 |
| O | -1.910300 | -2.235100 | 3.207200 |
| O | 0.924500 | 1.201000 | 2.694700 |
| C | -0.380200 | -0.241300 | 4.888600 |
| O | 0.845000 | 0.383200 | 5.321200 |
| H | 2.867900 | 0.377100 | 4.622000 |
| H | 1.844100 | -1.838600 | 5.807800 |
| H | 1.812800 | -0.619800 | 2.351100 |
| H | 4.552300 | -5.271400 | 4.963700 |
| H | 3.904800 | -1.018000 | 3.018300 |
| H | 5.872800 | -1.266800 | 1.540600 |
| H | 7.132400 | -2.226500 | 2.318500 |
| H | 6.115500 | -2.951400 | 1.078800 |
| H | -1.455200 | -0.505000 | 2.198400 |
| H | -0.493700 | -1.903600 | 1.752900 |
| H | -1.439200 | -3.004800 | 3.547800 |
| H | 0.619300 | 1.693900 | 3.465700 |
| H | -1.138600 | 0.508900 | 4.660600 |
| H | -0.782300 | -0.927100 | 5.636200 |

LNA-LockedmonoCl-Diol_7
Eopt -988.141167

| | | | |
|---|---|---|---|
| C | 1.354400 | -1.081700 | 5.181700 |
| C | 1.787900 | -2.447500 | 4.592200 |
| O | 0.907400 | -2.575000 | 3.500600 |
| C | 0.045800 | -1.429900 | 3.494800 |
| C | 1.024500 | -0.339800 | 3.897500 |
| N | 3.147600 | -2.747500 | 4.133800 |
| C | 4.199100 | -1.867100 | 4.329000 |
| N | 5.423000 | -2.376900 | 4.012100 |
| C | 5.770100 | -3.606800 | 3.557200 |
| C | 4.625400 | -4.503700 | 3.376700 |
| C | 3.396900 | -4.027300 | 3.675200 |
| C | 4.878200 | -5.913700 | 2.856300 |
| O | 6.947000 | -3.879800 | 3.338900 |
| O | 4.157000 | -0.713500 | 4.745000 |
| C | -0.639600 | -1.268100 | 2.122800 |
| O | -1.406400 | -0.076900 | 2.082500 |
| O | 0.477000 | 0.958500 | 4.099000 |
| C | -0.879500 | -1.552800 | 4.719200 |
| O | 0.046600 | -1.232700 | 5.771600 |
| H | 2.076500 | -0.629300 | 5.862000 |
| H | 1.607800 | -3.223000 | 5.341700 |
| H | 1.874300 | -0.280900 | 3.211300 |
| H | 6.194400 | -1.745700 | 4.143400 |
| H | 2.502000 | -4.624600 | 3.547700 |
| H | 3.959200 | -6.489700 | 2.745500 |
| H | 5.369800 | -5.876900 | 1.882300 |
| H | 5.536800 | -6.457600 | 3.535900 |
| H | 0.111700 | -1.237700 | 1.331900 |
| H | -1.284100 | -2.123100 | 1.913400 |
| H | -0.838500 | 0.648200 | 2.382100 |
| H | 0.046500 | 0.948300 | 4.962800 |
| H | -1.700100 | -0.833700 | 4.699800 |
| H | -1.295900 | -2.555400 | 4.825600 |

LNA-LockedmonoCl-Diol_8
Eopt -988.136202

| | | | |
|---|---|---|---|
| C | 1.498400 | -0.896900 | 5.314900 |
| C | 1.815900 | -2.246700 | 4.624500 |
| O | 0.956100 | -2.202200 | 3.511800 |
| C | 0.191900 | -0.990600 | 3.590600 |
| C | 1.256800 | -0.030500 | 4.090600 |
| N | 3.150800 | -2.639300 | 4.164600 |
| C | 4.278500 | -1.890800 | 4.458200 |
| N | 5.453900 | -2.491800 | 4.118100 |
| C | 5.689500 | -3.705100 | 3.558900 |
| C | 4.466900 | -4.462200 | 3.277400 |
| C | 3.284300 | -3.892200 | 3.596100 |
| C | 4.592300 | -5.838500 | 2.634900 |
| O | 6.839200 | -4.074700 | 3.336700 |
| O | 4.342000 | -0.780400 | 4.976700 |
| C | -0.452000 | -0.642900 | 2.230700 |
| O | -1.602000 | -1.436200 | 1.972300 |
| O | 0.831000 | 1.289300 | 4.403100 |
| C | -0.758700 | -1.122500 | 4.794100 |
| O | 0.172600 | -0.969700 | 5.879300 |
| H | 2.245900 | -0.564900 | 6.035300 |
| H | 1.543000 | -3.059300 | 5.303100 |
| H | 2.120800 | 0.006900 | 3.421000 |
| H | 6.279700 | -1.955700 | 4.320500 |
| H | 2.337200 | -4.380800 | 3.398600 |
| H | 3.623400 | -6.304700 | 2.454700 |
| H | 5.112200 | -5.765300 | 1.677900 |
| H | 5.173300 | -6.505200 | 3.274600 |
| H | -0.748500 | 0.407500 | 2.217900 |
| H | 0.278200 | -0.770800 | 1.430000 |
| H | -1.882400 | -1.297800 | 1.063200 |
| H | 0.345600 | 1.235800 | 5.234000 |
| H | -1.512900 | -0.334900 | 4.816100 |
| H | -1.263700 | -2.089500 | 4.811000 |

LNA-LockedmonoCl-Diol_9
Eopt -988.145330

| | | | |
|---|---|---|---|
| C | 1.593800 | -0.585500 | 5.121700 |
| C | 1.604100 | -2.119600 | 4.984100 |
| O | 0.492600 | -2.351200 | 4.148300 |
| C | -0.082200 | -1.066300 | 3.847100 |
| C | 1.158100 | -0.201800 | 3.711300 |
| N | 2.828800 | -2.753100 | 4.490500 |
| C | 3.926400 | -2.724300 | 5.331400 |
| N | 5.082700 | -3.169900 | 4.764400 |
| C | 5.331000 | -3.579300 | 3.494900 |
| C | 4.149800 | -3.535500 | 2.627100 |
| C | 2.980900 | -3.125100 | 3.167200 |
| C | 4.302900 | -3.945000 | 1.167000 |
| O | 6.459900 | -3.932500 | 3.166600 |
| O | 3.940200 | -2.322700 | 6.493000 |
| C | -1.021700 | -1.155000 | 2.625800 |
| O | -0.407200 | -1.867800 | 1.564900 |
| O | 0.935900 | 1.189300 | 3.490500 |
| C | -0.720100 | -0.528800 | 5.143700 |
| O | 0.451400 | -0.222700 | 5.915800 |
| H | 2.541100 | -0.183200 | 5.484800 |
| H | 1.420400 | -2.601000 | 5.951200 |
| H | 1.830200 | -0.584400 | 2.939300 |
| H | 5.884200 | -3.174700 | 5.371300 |
| H | 2.077000 | -3.073500 | 2.578300 |
| H | 3.372000 | -3.853300 | 0.606900 |
| H | 5.052500 | -3.323900 | 0.673100 |
| H | 4.637300 | -4.981700 | 1.096400 |
| H | -1.946700 | -1.664400 | 2.900000 |
| H | -1.297100 | -0.155800 | 2.283200 |
| H | -0.271700 | -2.768600 | 1.873600 |
| H | 1.734800 | 1.679700 | 3.702200 |
| H | -1.300500 | 0.379600 | 4.973500 |
| H | -1.352400 | -1.267200 | 5.638200 |

**Cartesian coordinates for compound 37**

LNA-LockedmonoCl-Acet_1
Eopt -1104.850607

| | | | |
|---|---|---|---|
| C | -6.625000 | 8.153000 | -1.921500 |
| C | -6.041700 | 6.952300 | -2.712300 |
| O | -5.348400 | 6.234200 | -1.705500 |
| C | -5.526600 | 6.989500 | -0.504600 |
| C | -6.947100 | 7.425500 | -0.627500 |
| N | -6.968800 | 6.110500 | -3.473100 |

C -7.631500 6.711700 -4.528300
N -8.635800 5.962400 -5.064500
C -9.108100 4.744600 -4.697500
C -8.395000 4.162900 -3.555900
C -7.380600 4.872100 -3.013100
C -8.828400 2.792600 -3.048500
O -10.048000 4.234600 -5.300500
O -7.408800 7.830200 -4.985900
C -5.355400 6.227500 0.811600
O -5.888000 6.988300 1.908700
C -7.179000 7.537700 1.735700
O -7.314400 8.216100 0.504500
C -4.712500 8.291900 -0.634600
O -5.531300 9.012200 -1.562900
C -8.221600 6.424900 1.852100
C -7.303000 8.607100 2.796700
H -7.450100 8.658200 -2.426200
H -5.318400 7.290100 -3.461600
H -7.674400 6.624000 -0.765500
H -9.118300 6.384100 -5.839400
H -6.807500 4.485100 -2.183500
H -8.226700 2.447200 -2.207900
H -9.871900 2.817800 -2.729100
H -8.747700 2.050500 -3.845200
H -5.823000 5.243600 0.772400
H -4.298600 6.036400 0.998600
H -4.652500 8.843700 0.305400
H -3.704100 8.125900 -1.015300
H -8.045300 5.613400 1.150900
H -8.195300 6.009900 2.859500
H -9.214600 6.837200 1.672800
H -6.529800 9.361700 2.650300
H -8.278700 9.087900 2.728900
H -7.186100 8.169500 3.787800

LNA-LockedmonoCl-Acet_2
Eopt -1104.846441
C -6.247000 8.011200 -1.948500
C -6.148000 6.567100 -2.520700
O -5.847600 5.808500 -1.365900
C -5.728400 6.753400 -0.298900
C -6.852400 7.684600 -0.591800
N -7.219100 5.908400 -3.272400
C -8.456200 6.501500 -3.462300
N -9.288600 5.805900 -4.288300
C -9.094100 4.636000 -4.947400
C -7.772900 4.047600 -4.710000
C -6.922500 4.711800 -3.897000
C -7.433800 2.721400 -5.380000
O -9.979900 4.168100 -5.657200
O -8.877700 7.551000 -2.985300
C -5.904700 6.202200 1.118200
O -6.100500 7.273300 2.057000
C -7.055800 8.255400 1.707900
O -6.883500 8.726300 0.388000
C -4.462500 7.600300 -0.538900
O -4.908100 8.424200 -1.624400
C -8.461900 7.683700 1.894000
C -6.753800 9.440000 2.597000
H -6.779900 8.718900 -2.584100
H -5.313600 6.548800 -3.227000
H -7.834300 7.215200 -0.665500
H -10.190300 6.224900 -4.437200
H -5.937300 4.329400 -3.659000
H -6.435000 2.367500 -5.124400
H -8.148900 1.951700 -5.083500
H -7.488600 2.819600 -6.465800
H -6.731700 5.494100 1.174800
H -5.020900 5.636200 1.412300
H -4.211600 8.228000 0.318300
H -3.593500 6.997700 -0.804900
H -8.616800 6.766600 1.331400
H -8.626700 7.463300 2.948400
H -9.198400 8.420400 1.573000
H -5.739000 9.791300 2.409400
H -7.450300 10.250900 2.384700
H -6.841800 9.155600 3.645200

LNA-LockedmonoCl-Acet_3
Eopt -1104.843946
C -7.315200 8.268800 -1.590100
C -6.646000 7.390400 -2.676100
O -5.614500 6.742100 -1.959200
C -5.626000 7.333800 -0.657100
C -7.088200 7.360900 -0.386100
N -7.424200 6.441200 -3.475400
C -6.814900 5.925700 -4.608100
N -7.508100 4.919400 -5.211000
C -8.690400 4.347100 -4.873000
C -9.297500 4.931100 -3.673000
C -8.637700 5.932000 -3.048700
C -10.639500 4.385200 -3.198400
O -9.156100 3.433000 -5.547600
O -5.745200 6.280600 -5.099700
C -4.932200 6.540100 0.452000
O -5.333700 7.026400 1.744300
C -6.720300 7.198200 1.957800
O -7.344000 7.909200 0.908300
C -5.228700 8.821700 -0.767500
O -6.431500 9.369300 -1.328100
C -7.376200 5.829000 2.141300
C -6.829100 8.096600 3.168600
H -8.338000 8.566900 -1.821500
H -6.202100 8.064000 -3.418400
H -7.571700 6.387700 -0.470100
H -7.072700 4.535200 -6.031700
H -9.054900 6.400200 -2.173300
H -11.009200 4.891500 -2.306800
H -11.391900 4.493800 -3.982000
H -10.556700 3.320700 -2.970900
H -5.131500 5.471400 0.369300
H -3.850300 6.637300 0.360000
H -5.036600 9.277800 0.205500
H -4.362600 8.978100 -1.411800
H -7.188100 5.160900 1.304700
H -6.980000 5.357600 3.040600
H -8.452300 5.953400 2.260700
H -6.339800 9.049300 2.964500
H -7.877000 8.283300 3.401900
H -6.347300 7.629900 4.027400

LNA-LockedmonoCl-Acet_4
Eopt -1104.843619
C -6.632800 8.045000 -1.930400
C -6.235200 6.683100 -2.541300
O -5.634300 5.991300 -1.469700
C -5.615600 6.945000 -0.398800
C -6.955300 7.591400 -0.517400
N -7.207800 5.909100 -3.315100
C -8.082400 5.044300 -2.676000
N -8.929300 4.386500 -3.517400
C -9.054200 4.453500 -4.866500
C -8.117300 5.391700 -5.491500
C -7.258000 6.058600 -4.689700
C -8.166500 5.570000 -7.004500
O -9.893100 3.771300 -5.448000
O -8.179700 4.806400 -1.474300
C -5.479300 6.367600 1.011000
O -5.788700 7.368900 1.996600
C -6.984300 8.098600 1.803200
O -7.101500 8.599600 0.487000
C -4.636200 8.083300 -0.766200
O -5.413600 8.786200 -1.752800
C -8.181000 7.210800 2.150100
C -6.852800 9.319700 2.684600
H -7.422400 8.561200 -2.477300
H -5.414600 6.834400 -3.246400
H -7.813400 6.926900 -0.470400
H -9.563100 3.747900 -3.069000
H -6.551100 6.771900 -5.092900
H -7.437500 6.295400 -7.365700
H -7.970400 4.619600 -7.504600
H -9.157400 5.905600 -7.316300
H -6.111500 5.489100 1.146000
H -4.460700 6.016400 1.174300
H -4.428800 8.744400 0.077400
H -3.691800 7.713200 -1.166600
H -8.193400 6.285000 1.579000
H -8.144000 6.950300 3.207500
H -9.105800 7.753900 1.957200
H -5.981300 9.900000 2.381200
H -7.741000 9.943900 2.590200
H -6.733000 9.020000 3.725400

LNA-LockedmonoCl-Acet_5
Eopt -1104.844994
C -6.660300 8.206600 -1.929000
C -6.046100 7.022200 -2.719300
O -5.310900 6.336200 -1.719800
C -5.492300 7.090500 -0.517500
C -6.932400 7.472100 -0.629700
N -6.956200 6.142200 -3.456400
C -7.630200 6.701400 -4.527300
N -8.610700 5.912600 -5.050700
C -9.053400 4.692400 -4.656000
C -8.336300 4.160600 -3.492700
C -7.343100 4.908600 -2.963100
C -8.745700 2.798300 -2.945400
O -9.973100 4.141900 -5.254600
O -7.435600 7.815300 -5.009100
C -5.272300 6.337000 0.808100
O -6.469700 6.257100 1.601300
C -7.245300 7.439000 1.706000
O -7.271900 8.224600 0.532800
C -4.729700 8.421900 -0.678400
O -5.595000 9.108500 -1.589100
C -8.663300 6.982700 1.981300
C -6.625600 8.310100 2.781300
H -7.512800 8.674300 -2.424100
H -5.346200 7.379100 -3.481900
H -7.627100 6.634600 -0.733300
H -9.099000 6.301600 -5.838900
H -6.772000 4.562500 -2.113600
H -8.150500 2.498200 -2.082800
H -9.794500 2.809200 -2.642900
H -8.633600 2.031300 -3.713900
H -4.944700 5.318000 0.601700
H -4.470100 6.779100 1.399700

| | | | |
|---|---|---|---|
| H | -4.662900 | 8.987800 | 0.251700 |
| H | -3.727100 | 8.288200 | -1.086600 |
| H | -9.020000 | 6.344300 | 1.174200 |
| H | -8.697000 | 6.420100 | 2.914000 |
| H | -9.320700 | 7.848000 | 2.064200 |
| H | -5.627900 | 8.630500 | 2.487000 |
| H | -7.241400 | 9.196200 | 2.935300 |
| H | -6.561700 | 7.755700 | 3.717400 |

LNA-LockedmonoCl-Acet_6
Eopt -1104.840158

| | | | |
|---|---|---|---|
| C | -6.256600 | 8.077300 | -1.971100 |
| C | -6.122800 | 6.639200 | -2.551300 |
| O | -5.743900 | 5.893100 | -1.410000 |
| C | -5.644700 | 6.832600 | -0.335300 |
| C | -6.817700 | 7.713500 | -0.607600 |
| N | -7.201200 | 5.933900 | -3.249400 |
| C | -8.444900 | 6.509000 | -3.453200 |
| N | -9.276100 | 5.776600 | -4.248100 |
| C | -9.072800 | 4.588900 | -4.871600 |
| C | -7.743000 | 4.023700 | -4.626100 |
| C | -6.894900 | 4.722800 | -3.840500 |
| C | -7.392500 | 2.681900 | -5.258000 |
| O | -9.957500 | 4.089100 | -5.560900 |
| O | -8.873700 | 7.571600 | -3.013500 |
| C | -5.765600 | 6.263500 | 1.091700 |
| O | -6.903900 | 6.780800 | 1.803100 |
| C | -7.160900 | 8.168900 | 1.679300 |
| O | -6.846400 | 8.709700 | 0.413900 |
| C | -4.422100 | 7.732600 | -0.608000 |
| O | -4.927500 | 8.541100 | -1.676700 |
| C | -8.651600 | 8.337800 | 1.890300 |
| C | -6.278500 | 8.894300 | 2.675900 |
| H | -6.829100 | 8.770200 | -2.587800 |
| H | -5.322400 | 6.650000 | -3.295700 |
| H | -7.780400 | 7.196600 | -0.647300 |
| H | -10.183500 | 6.180800 | -4.403100 |
| H | -5.903300 | 4.360400 | -3.598400 |
| H | -6.387100 | 2.348800 | -5.000300 |
| H | -8.095000 | 1.912900 | -4.931100 |
| H | -7.458100 | 2.745600 | -6.345700 |
| H | -5.873500 | 5.179700 | 1.046000 |
| H | -4.861200 | 6.436700 | 1.675500 |
| H | -4.169900 | 8.371100 | 0.239800 |
| H | -3.537700 | 7.166500 | -0.902300 |
| H | -9.206300 | 7.767800 | 1.146000 |
| H | -8.928800 | 7.982600 | 2.882400 |
| H | -8.920900 | 9.389900 | 1.799600 |
| H | -5.227700 | 8.751500 | 2.430600 |
| H | -6.498600 | 9.961600 | 2.651000 |
| H | -6.465800 | 8.516600 | 3.680800 |

**Cartesian coordinates for compound 17**

LNA-OpenAlkynyl-Acet_1
Eopt -1142.869539

| | | | |
|---|---|---|---|
| C | -5.872200 | 2.867900 | -1.072500 |
| C | -4.729000 | 3.924500 | -1.024000 |
| O | -4.541000 | 4.262700 | 0.345900 |
| C | -5.750700 | 4.031500 | 1.073300 |
| C | -6.073500 | 2.694500 | 0.429900 |
| N | -3.445200 | 3.606900 | -1.647700 |
| C | -2.777700 | 2.458800 | -1.254800 |
| N | -1.578900 | 2.270100 | -1.875200 |
| C | -0.950700 | 3.020300 | -2.814800 |
| C | -1.701000 | 4.220000 | -3.199300 |
| C | -2.892500 | 4.443500 | -2.601300 |
| C | -1.099100 | 5.149600 | -4.246500 |
| O | 0.138900 | 2.671400 | -3.260800 |
| O | -3.149800 | 1.617000 | -0.439100 |
| C | -5.620300 | 3.705000 | 2.568000 |
| O | -6.836500 | 3.090600 | 3.048700 |
| C | -7.368000 | 2.010300 | 2.298600 |
| O | -7.336800 | 2.214700 | 0.894100 |
| C | -6.710800 | 5.154300 | 0.851400 |
| O | -7.005500 | 3.391000 | -1.747800 |
| C | -6.592700 | 0.743800 | 2.659700 |
| C | -8.834000 | 1.952900 | 2.662400 |
| C | -7.487700 | 6.081100 | 0.676200 |
| H | -5.541600 | 1.931700 | -1.527500 |
| H | -5.074900 | 4.834600 | -1.518600 |
| H | -5.336300 | 1.946800 | 0.723100 |
| H | -1.080500 | 1.442500 | -1.597900 |
| H | -3.484800 | 5.315300 | -2.842300 |
| H | -1.734200 | 6.010300 | -4.456400 |
| H | -0.129300 | 5.522100 | -3.911200 |
| H | -0.939100 | 4.613600 | -5.183800 |
| H | -4.767900 | 3.053800 | 2.765300 |
| H | -5.422000 | 4.605900 | 3.150100 |
| H | -7.774700 | 2.908200 | -1.422900 |
| H | -5.517400 | 0.870400 | 2.556700 |
| H | -6.794200 | 0.479500 | 3.697500 |
| H | -6.915300 | -0.078800 | 2.021900 |
| H | -9.317200 | 2.889000 | 2.381800 |
| H | -9.318100 | 1.131900 | 2.134200 |
| H | -8.948200 | 1.806300 | 3.736200 |
| H | -8.174300 | 6.882700 | 0.520900 |

LNA-OpenAlkynyl-Acet_2
Eopt -1142.866630

| | | | |
|---|---|---|---|
| C | -5.873700 | 2.872000 | -1.072600 |
| C | -4.738600 | 3.932800 | -1.029400 |
| O | -4.543200 | 4.253600 | 0.345300 |
| C | -5.756500 | 4.036600 | 1.072400 |
| C | -6.063900 | 2.694500 | 0.431400 |
| N | -3.461000 | 3.560300 | -1.631400 |
| C | -2.937000 | 4.330000 | -2.656100 |
| N | -1.732000 | 3.892400 | -3.120500 |
| C | -0.972000 | 2.838600 | -2.727600 |
| C | -1.558700 | 2.078700 | -1.618600 |
| C | -2.756200 | 2.480500 | -1.138600 |
| C | -0.784500 | 0.892000 | -1.057600 |
| O | 0.094900 | 2.597900 | -3.285600 |
| O | -3.439500 | 5.325300 | -3.173600 |
| C | -5.631800 | 3.725000 | 2.570800 |
| O | -6.837700 | 3.090100 | 3.048500 |
| C | -7.350000 | 1.997900 | 2.303700 |
| O | -7.315900 | 2.192400 | 0.898400 |
| C | -6.718100 | 5.155400 | 0.838200 |
| O | -7.017500 | 3.379200 | -1.738900 |
| C | -6.556100 | 0.746800 | 2.676000 |
| C | -8.816300 | 1.920600 | 2.662200 |
| C | -7.491900 | 6.079900 | 0.639200 |
| H | -5.532900 | 1.942900 | -1.534700 |
| H | -5.081300 | 4.849900 | -1.524100 |
| H | -5.315000 | 1.956300 | 0.721600 |
| H | -1.344100 | 4.434000 | -3.873400 |
| H | -3.236600 | 1.987300 | -0.307500 |
| H | -1.297700 | 0.414100 | -0.223300 |
| H | -0.628400 | 0.140100 | -1.833400 |
| H | 0.199100 | 1.211500 | -0.707900 |
| H | -4.769100 | 3.092800 | 2.781800 |
| H | -5.456100 | 4.634500 | 3.147500 |
| H | -7.787900 | 2.910500 | -1.398300 |
| H | -5.483200 | 0.890300 | 2.576500 |
| H | -6.757700 | 0.488500 | 3.715500 |
| H | -6.866500 | -0.086300 | 2.045600 |
| H | -9.312600 | 2.847500 | 2.373500 |
| H | -9.286900 | 1.089400 | 2.137800 |
| H | -8.933400 | 1.780100 | 3.736500 |
| H | -8.165900 | 6.885000 | 0.449800 |

LNA-OpenAlkynyl-Acet_3
Eopt -1142.865566

| | | | |
|---|---|---|---|
| C | -5.875200 | 2.871400 | -1.074500 |
| C | -4.730200 | 3.925000 | -1.027800 |
| O | -4.541900 | 4.254200 | 0.346800 |
| C | -5.757300 | 4.036100 | 1.071200 |
| C | -6.062900 | 2.694900 | 0.432900 |
| N | -3.481700 | 3.565800 | -1.693800 |
| C | -3.547200 | 3.395000 | -3.066000 |
| N | -2.418900 | 2.868900 | -3.621200 |
| C | -1.280100 | 2.427500 | -3.029600 |
| C | -1.290200 | 2.564600 | -1.569200 |
| C | -2.381300 | 3.113900 | -0.990300 |
| C | -0.075700 | 2.083500 | -0.784600 |
| O | -0.370300 | 1.961800 | -3.709300 |
| O | -4.519900 | 3.643400 | -3.780000 |
| C | -5.616800 | 3.734600 | 2.570900 |
| O | -6.804000 | 3.084500 | 3.069200 |
| C | -7.301100 | 1.977500 | 2.337000 |
| O | -7.291600 | 2.166400 | 0.932500 |
| C | -6.720900 | 5.151500 | 0.833200 |
| O | -6.968200 | 3.405900 | -1.808700 |
| C | -6.474300 | 0.745700 | 2.701600 |
| C | -8.760200 | 1.868600 | 2.715500 |
| C | -7.500300 | 6.072600 | 0.640600 |
| H | -5.529200 | 1.935600 | -1.519100 |
| H | -5.020300 | 4.860600 | -1.521600 |
| H | -5.295700 | 1.969100 | 0.710700 |
| H | -2.439100 | 2.759400 | -4.620600 |
| H | -2.446300 | 3.250900 | 0.079800 |
| H | -0.186200 | 2.234000 | 0.289400 |
| H | 0.094400 | 1.019600 | -0.959700 |
| H | 0.820500 | 2.616900 | -1.107000 |
| H | -4.743100 | 3.116200 | 2.776500 |
| H | -5.448900 | 4.649600 | 3.140800 |
| H | -6.730100 | 3.431000 | -2.743300 |
| H | -5.407300 | 0.908900 | 2.574700 |
| H | -6.648500 | 0.490900 | 3.746800 |
| H | -6.782600 | -0.096700 | 2.082600 |
| H | -9.280400 | 2.782000 | 2.425700 |
| H | -9.217400 | 1.025400 | 2.198400 |
| H | -8.861300 | 1.730600 | 3.791600 |
| H | -8.197700 | 6.859600 | 0.461200 |

LNA-OpenAlkynyl-Acet_4
Eopt -1142.864271

| | | | |
|---|---|---|---|
| C | -5.871400 | 2.865600 | -1.075000 |
| C | -4.731500 | 3.924800 | -1.027000 |
| O | -4.543500 | 4.259200 | 0.343800 |
| C | -5.751300 | 4.034900 | 1.080300 |
| C | -6.071100 | 2.696800 | 0.427300 |
| N | -3.445400 | 3.615800 | -1.651200 |
| C | -2.785500 | 2.454200 | -1.286400 |
| N | -1.577300 | 2.283700 | -1.893600 |

| | | | |
|---|---|---|---|
| C | -0.932600 | 3.063700 | -2.797000 |
| C | -1.673800 | 4.278300 | -3.151200 |
| C | -2.874400 | 4.484000 | -2.565300 |
| C | -1.052000 | 5.243300 | -4.153800 |
| O | 0.162900 | 2.727700 | -3.238400 |
| O | -3.170900 | 1.585800 | -0.506400 |
| C | -5.594300 | 3.725800 | 2.589200 |
| O | -6.164000 | 2.455100 | 2.979200 |
| C | -7.335800 | 2.016800 | 2.305200 |
| O | -7.332400 | 2.225500 | 0.901400 |
| C | -6.703900 | 5.161000 | 0.844700 |
| O | -7.006800 | 3.385200 | -1.750800 |
| C | -7.382300 | 0.519400 | 2.514000 |
| C | -8.521400 | 2.780400 | 2.866100 |
| C | -7.471700 | 6.092400 | 0.653800 |
| H | -5.540200 | 1.928500 | -1.526900 |
| H | -5.082400 | 4.832300 | -1.522400 |
| H | -5.346200 | 1.937900 | 0.739500 |
| H | -1.085800 | 1.445700 | -1.635600 |
| H | -3.459500 | 5.366400 | -2.783700 |
| H | -1.680500 | 6.113800 | -4.342000 |
| H | -0.086300 | 5.599000 | -3.789800 |
| H | -0.878900 | 4.741100 | -5.107400 |
| H | -4.532400 | 3.680600 | 2.831600 |
| H | -5.997400 | 4.510100 | 3.232700 |
| H | -7.766000 | 2.862600 | -1.468000 |
| H | -6.493600 | 0.058700 | 2.082400 |
| H | -7.414000 | 0.292500 | 3.579400 |
| H | -8.266200 | 0.102500 | 2.032200 |
| H | -8.430200 | 3.844700 | 2.661300 |
| H | -9.441700 | 2.416800 | 2.409700 |
| H | -8.578200 | 2.632500 | 3.944200 |
| H | -8.145000 | 6.900500 | 0.473800 |

LNA-OpenAlkynyl-Acet_5
Eopt -1142.862237

| | | | |
|---|---|---|---|
| C | -5.873200 | 2.868400 | -1.075000 |
| C | -4.735000 | 3.924000 | -1.030400 |
| O | -4.545600 | 4.251500 | 0.343800 |
| C | -5.756000 | 4.039500 | 1.079900 |
| C | -6.062600 | 2.697200 | 0.427700 |
| N | -3.455900 | 3.538500 | -1.620700 |
| C | -2.933600 | 4.280600 | -2.666200 |
| N | -1.729400 | 3.830500 | -3.120600 |
| C | -0.969300 | 2.787000 | -2.701400 |
| C | -1.554200 | 2.057100 | -1.571300 |
| C | -2.750700 | 2.472000 | -1.100200 |
| C | -0.778900 | 0.886400 | -0.979300 |
| O | 0.096200 | 2.531200 | -3.255500 |
| O | -3.437800 | 5.261300 | -3.209700 |
| C | -5.599800 | 3.742000 | 2.591800 |
| O | -6.121600 | 2.448900 | 2.974700 |
| C | -7.298900 | 1.997800 | 2.318300 |
| O | -7.304000 | 2.187700 | 0.911700 |
| C | -6.708900 | 5.162300 | 0.833500 |
| O | -7.014500 | 3.379500 | -1.743700 |
| C | -7.338100 | 0.503000 | 2.546100 |
| C | -8.486300 | 2.760800 | 2.878100 |
| C | -7.474300 | 6.091500 | 0.624500 |
| H | -5.533400 | 1.936800 | -1.532600 |
| H | -5.068600 | 4.839700 | -1.533300 |
| H | -5.322600 | 1.949800 | 0.732200 |
| H | -1.342400 | 4.351400 | -3.888300 |
| H | -3.229800 | 2.002200 | -0.254100 |
| H | -1.290500 | 0.433700 | -0.129900 |
| H | -0.625900 | 0.112200 | -1.733300 |
| H | 0.205700 | 1.214900 | -0.641100 |
| H | -4.540600 | 3.748400 | 2.849200 |
| H | -6.045200 | 4.507700 | 3.230300 |
| H | -7.776200 | 2.858500 | -1.467200 |
| H | -6.452000 | 0.038000 | 2.115000 |
| H | -7.363300 | 0.290100 | 3.614800 |
| H | -8.225000 | 0.077600 | 2.076900 |
| H | -8.413800 | 3.821200 | 2.646800 |
| H | -9.408400 | 2.376000 | 2.443000 |
| H | -8.527700 | 2.637700 | 3.960100 |
| H | -8.136400 | 6.902500 | 0.417200 |

**Cartesian coordinates for compound 18**

LNA-LockedAlkynyl-Acet_1
Eopt -1142.922372

| | | | |
|---|---|---|---|
| C | -3.831800 | 5.835700 | 3.824900 |
| C | -5.358200 | 5.945000 | 4.087800 |
| O | -5.705800 | 4.592000 | 4.344800 |
| C | -4.489900 | 3.842800 | 4.217600 |
| C | -3.516900 | 4.769000 | 4.862700 |
| N | -5.820200 | 6.859900 | 5.134200 |
| C | -5.554000 | 8.203500 | 4.944300 |
| N | -5.809000 | 8.988300 | 6.028700 |
| C | -6.239400 | 8.648800 | 7.269800 |
| C | -6.475400 | 7.210700 | 7.431800 |
| C | -6.251600 | 6.407300 | 6.368200 |
| C | -6.964800 | 6.700900 | 8.782200 |
| O | -6.391900 | 9.509500 | 8.131800 |
| O | -5.102800 | 8.714900 | 3.921900 |
| C | -4.409500 | 2.478900 | 4.911700 |
| O | -3.047200 | 2.027300 | 5.002500 |
| C | -2.099900 | 2.961600 | 5.480900 |
| O | -2.199400 | 4.214600 | 4.835500 |
| C | -4.069400 | 3.873800 | 2.754400 |
| O | -3.661300 | 5.152600 | 2.569500 |
| C | -2.270700 | 3.124600 | 6.991700 |
| C | -0.754200 | 2.407800 | 5.072100 |
| C | -4.018500 | 2.955100 | 1.779300 |
| H | -3.289200 | 6.778100 | 3.908200 |
| H | -5.886400 | 6.283000 | 3.190000 |
| H | -3.762200 | 5.073700 | 5.881500 |
| H | -5.630200 | 9.969200 | 5.899400 |
| H | -6.425800 | 5.342200 | 6.422000 |
| H | -7.119600 | 5.621900 | 8.791900 |
| H | -6.244300 | 6.945100 | 9.565100 |
| H | -7.910700 | 7.177000 | 9.047500 |
| H | -4.861500 | 2.508700 | 5.902900 |
| H | -4.988700 | 1.740400 | 4.357800 |
| H | -3.277600 | 3.429200 | 7.265200 |
| H | -2.060700 | 2.174800 | 7.483300 |
| H | -1.565200 | 3.869700 | 7.358900 |
| H | -0.710200 | 2.318800 | 3.986300 |
| H | 0.040500 | 3.076500 | 5.401900 |
| H | -0.603000 | 1.423100 | 5.513200 |
| H | -3.658200 | 3.221100 | 0.797300 |
| H | -4.328900 | 1.936100 | 1.947400 |

LNA-LockedAlkynyl-Acet_2
Eopt -1142.918551

| | | | |
|---|---|---|---|
| C | -4.011200 | 5.711200 | 3.655800 |
| C | -5.384100 | 5.833200 | 4.385600 |
| O | -5.555000 | 4.514100 | 4.878600 |
| C | -4.400300 | 3.775300 | 4.462900 |
| C | -3.322600 | 4.786800 | 4.648000 |
| N | -5.657000 | 6.779900 | 5.470200 |
| C | -4.777600 | 7.802300 | 5.785000 |
| N | -5.267400 | 8.708500 | 6.677800 |
| C | -6.479900 | 8.776800 | 7.282500 |
| C | -7.381200 | 7.686100 | 6.901300 |
| C | -6.923000 | 6.765500 | 6.025200 |
| C | -8.775900 | 7.644100 | 7.514300 |
| O | -6.735400 | 9.689900 | 8.062400 |
| O | -3.639700 | 7.981400 | 5.362900 |
| C | -4.031500 | 2.513600 | 5.250600 |
| O | -2.692000 | 2.088800 | 4.944100 |
| C | -1.690600 | 3.087100 | 4.973200 |
| O | -2.058700 | 4.245100 | 4.252800 |
| C | -4.469100 | 3.625200 | 2.950400 |
| O | -4.222900 | 4.878500 | 2.499100 |
| C | -1.372000 | 3.441500 | 6.426200 |
| C | -0.522000 | 2.500100 | 4.215200 |
| C | -4.672800 | 2.589300 | 2.124100 |
| H | -3.514200 | 6.650100 | 3.417000 |
| H | -6.153400 | 6.077200 | 3.648400 |
| H | -3.245700 | 5.213600 | 5.649900 |
| H | -4.634900 | 9.450900 | 6.921300 |
| H | -7.525500 | 5.921800 | 5.709600 |
| H | -9.357600 | 6.788100 | 7.171700 |
| H | -8.711500 | 7.591600 | 8.602800 |
| H | -9.329800 | 8.550200 | 7.261700 |
| H | -4.140200 | 2.669000 | 6.323900 |
| H | -4.720700 | 1.704800 | 5.009500 |
| H | -2.251500 | 3.766000 | 6.976400 |
| H | -0.961700 | 2.567200 | 6.930700 |
| H | -0.629600 | 4.239100 | 6.450600 |
| H | -0.826500 | 2.272900 | 3.193200 |
| H | 0.299700 | 3.215200 | 4.186100 |
| H | -0.184800 | 1.582400 | 4.696000 |
| H | -4.660400 | 2.732400 | 1.054600 |
| H | -4.847900 | 1.594100 | 2.501500 |

LNA-LockedAlkynyl-Acet_3
Eopt -1142.919589

| | | | |
|---|---|---|---|
| C | -3.784300 | 5.859800 | 3.821900 |
| C | -5.316000 | 5.957600 | 4.051700 |
| O | -5.667500 | 4.598100 | 4.265600 |
| C | -4.450500 | 3.848300 | 4.142500 |
| C | -3.498900 | 4.767300 | 4.836500 |
| N | -5.801500 | 6.843400 | 5.112000 |
| C | -5.566300 | 8.195600 | 4.945000 |
| N | -5.854500 | 8.957400 | 6.037500 |
| C | -6.287700 | 8.588300 | 7.269200 |
| C | -6.479300 | 7.141300 | 7.410300 |
| C | -6.224500 | 6.360000 | 6.337300 |
| C | -6.954300 | 6.597300 | 8.752400 |
| O | -6.476700 | 9.433200 | 8.139700 |
| O | -5.115500 | 8.732800 | 3.935400 |
| C | -4.386000 | 2.459800 | 4.810300 |
| O | -3.406200 | 2.401600 | 5.862300 |
| C | -2.144200 | 2.985300 | 5.585500 |
| O | -2.200900 | 4.177800 | 4.830900 |
| C | -4.003000 | 3.926900 | 2.687600 |
| O | -3.576200 | 5.206800 | 2.555600 |
| C | -1.547200 | 3.333600 | 6.932500 |
| C | -1.344200 | 2.001100 | 4.754000 |
| C | -3.956500 | 3.051400 | 1.672700 |
| H | -3.247800 | 6.801000 | 3.947500 |
| H | -5.825600 | 6.316400 | 3.151100 |
| H | -3.767200 | 5.023500 | 5.865000 |

| | | | |
|---|---|---|---|
| H | -5.702400 | 9.944700 | 5.924100 |
| H | -6.358500 | 5.288000 | 6.377300 |
| H | -7.062400 | 5.512500 | 8.749100 |
| H | -6.248500 | 6.862000 | 9.541900 |
| H | -7.920900 | 7.030000 | 9.017200 |
| H | -5.355000 | 2.225100 | 5.250500 |
| H | -4.198900 | 1.658100 | 4.096700 |
| H | -2.194900 | 4.032200 | 7.460700 |
| H | -1.437500 | 2.431500 | 7.533900 |
| H | -0.568200 | 3.792700 | 6.796200 |
| H | -1.805800 | 1.867300 | 3.776500 |
| H | -0.333600 | 2.381600 | 4.607100 |
| H | -1.292300 | 1.039000 | 5.262800 |
| H | -3.575200 | 3.357900 | 0.710200 |
| H | -4.293500 | 2.032900 | 1.780500 |

LNA-LockedAlkynyl-Acet_4
Eopt -1142.914899

| | | | |
|---|---|---|---|
| C | -3.286300 | 5.877700 | 4.243600 |
| C | -4.789400 | 6.250700 | 4.199700 |
| O | -5.410700 | 4.979200 | 4.168100 |
| C | -4.345000 | 4.024100 | 4.055600 |
| C | -3.397500 | 4.586700 | 5.052700 |
| N | -5.392400 | 7.126700 | 5.207400 |
| C | -6.629000 | 7.674100 | 4.907200 |
| N | -7.219700 | 8.331900 | 5.943700 |
| C | -6.783000 | 8.538000 | 7.211100 |
| C | -5.463700 | 7.955000 | 7.473800 |
| C | -4.850400 | 7.289900 | 6.469500 |
| C | -4.852500 | 8.134700 | 8.858800 |
| O | -7.469600 | 9.163600 | 8.014100 |
| O | -7.215800 | 7.627200 | 3.828000 |
| C | -4.646300 | 2.568400 | 4.426100 |
| O | -3.428700 | 1.834700 | 4.647800 |
| C | -2.467000 | 2.440600 | 5.488300 |
| O | -2.215100 | 3.788500 | 5.144900 |
| C | -3.612700 | 4.241600 | 2.734900 |
| O | -2.938300 | 5.403600 | 2.930300 |
| C | -2.932400 | 2.338400 | 6.941300 |
| C | -1.175100 | 1.708500 | 5.205800 |
| C | -3.521200 | 3.540800 | 1.595700 |
| H | -2.633800 | 6.661600 | 4.626600 |
| H | -4.977900 | 6.758600 | 3.245900 |
| H | -3.826200 | 4.709900 | 6.047200 |
| H | -8.121400 | 8.725400 | 5.737400 |
| H | -3.874400 | 6.859100 | 6.615000 |
| H | -3.869500 | 7.672900 | 8.949900 |
| H | -4.747800 | 9.195900 | 9.093200 |
| H | -5.501500 | 7.695500 | 9.619000 |
| H | -5.290700 | 2.505000 | 5.302800 |
| H | -5.208600 | 2.086400 | 3.626700 |
| H | -3.911800 | 2.784000 | 7.096100 |
| H | -2.993500 | 1.288300 | 7.226900 |
| H | -2.211400 | 2.833800 | 7.590900 |
| H | -0.912100 | 1.824100 | 4.153900 |
| H | -0.371400 | 2.118600 | 5.816600 |
| H | -1.289100 | 0.647200 | 5.425400 |
| H | -2.910600 | 3.897600 | 0.780600 |
| H | -4.048500 | 2.608900 | 1.464600 |

**Cartesian coordinates for compound 17'**

LNA-OpenAlkynyl-Diol_1
Eopt -1026.170916

| | | | |
|---|---|---|---|
| C | 1.922200 | 0.697400 | 4.519200 |
| C | 1.027600 | 0.785900 | 5.755500 |
| O | 0.824800 | -0.525700 | 6.196100 |
| C | 0.951100 | -1.470400 | 5.128400 |
| C | 1.370800 | -0.592900 | 3.899400 |
| N | 1.442300 | 1.662300 | 6.846200 |
| C | 2.592900 | 1.333800 | 7.543800 |
| N | 2.925500 | 2.227700 | 8.518000 |
| C | 2.318200 | 3.382600 | 8.891100 |
| C | 1.118000 | 3.691300 | 8.106200 |
| C | 0.751800 | 2.823400 | 7.136800 |
| C | 0.353800 | 4.972300 | 8.419300 |
| O | 2.782900 | 4.059700 | 9.803600 |
| O | 3.313300 | 0.348000 | 7.369000 |
| C | 2.040600 | -2.483800 | 5.588700 |
| O | 3.335500 | -1.905600 | 5.713800 |
| O | 0.383800 | -0.278500 | 2.914800 |
| C | -0.366400 | -2.143800 | 5.002400 |
| O | 1.813200 | 1.895500 | 3.753400 |
| C | -1.448300 | -2.705400 | 4.913700 |
| H | 2.953100 | 0.540000 | 4.841300 |
| H | 0.036500 | 1.139400 | 5.460500 |
| H | 2.180600 | -1.096500 | 3.367200 |
| H | 3.752200 | 2.003100 | 9.044100 |
| H | -0.117300 | 2.999100 | 6.518300 |
| H | -0.513100 | 5.110400 | 7.772900 |
| H | 0.003700 | 4.962500 | 9.453100 |
| H | 1.003000 | 5.841800 | 8.300400 |
| H | 1.766300 | -2.930800 | 6.546800 |
| H | 2.115000 | -3.305300 | 4.873300 |
| H | 3.310700 | -1.231200 | 6.418500 |
| H | 0.418300 | 0.679900 | 2.801100 |
| H | 2.579100 | 1.961800 | 3.170600 |
| H | -2.396500 | -3.187000 | 4.827200 |

LNA-OpenAlkynyl-Diol_10
Eopt -1026.167910

| | | | |
|---|---|---|---|
| C | 1.913800 | 0.677600 | 4.533300 |
| C | 1.021200 | 0.801500 | 5.762500 |
| O | 0.839600 | -0.521400 | 6.175000 |
| C | 0.628600 | -1.355700 | 5.025900 |
| C | 1.221300 | -0.507600 | 3.838700 |
| N | 1.470100 | 1.649200 | 6.865500 |
| C | 0.624900 | 2.628000 | 7.361700 |
| N | 1.139800 | 3.327700 | 8.412700 |
| C | 2.326000 | 3.192600 | 9.058000 |
| C | 3.164400 | 2.117800 | 8.516800 |
| C | 2.691300 | 1.414000 | 7.464800 |
| C | 4.513900 | 1.846200 | 9.171200 |
| O | 2.607200 | 3.926300 | 10.001800 |
| O | -0.504500 | 2.912800 | 6.964500 |
| C | 1.397500 | -2.678900 | 5.300700 |
| O | 2.784000 | -2.464800 | 5.528400 |
| O | 0.325300 | -0.036900 | 2.817800 |
| C | -0.831200 | -1.623800 | 4.931800 |
| O | 1.958700 | 1.902600 | 3.824300 |
| C | -2.033900 | -1.829300 | 4.864200 |
| H | 2.916800 | 0.374100 | 4.836100 |
| H | 0.043700 | 1.185500 | 5.444000 |
| H | 1.993900 | -1.089600 | 3.330800 |
| H | 0.545000 | 4.052400 | 8.774900 |
| H | 3.246000 | 0.600000 | 7.024700 |
| H | 5.050100 | 1.024300 | 8.696800 |
| H | 5.145900 | 2.734600 | 9.116700 |
| H | 4.383000 | 1.599800 | 10.226400 |
| H | 0.983500 | -3.194600 | 6.170600 |
| H | 1.291200 | -3.361500 | 4.455100 |
| H | 2.878400 | -2.053200 | 6.394200 |
| H | -0.208900 | -0.772700 | 2.501000 |
| H | 1.371200 | 1.786500 | 3.064200 |
| H | -3.089200 | -1.990400 | 4.827400 |

LNA-OpenAlkynyl-Diol_11
Eopt -1026.174306

| | | | |
|---|---|---|---|
| C | 1.911800 | 0.664100 | 4.537700 |
| C | 1.013600 | 0.813700 | 5.771600 |
| O | 0.849100 | -0.520100 | 6.161500 |
| C | 0.581800 | -1.353700 | 5.024900 |
| C | 1.201100 | -0.516300 | 3.851600 |
| N | 1.363400 | 1.628600 | 6.938700 |
| C | 2.401100 | 2.548400 | 6.903700 |
| N | 2.493100 | 3.331500 | 8.016600 |
| C | 1.707100 | 3.382300 | 9.120800 |
| C | 0.596700 | 2.426800 | 9.088200 |
| C | 0.489300 | 1.619900 | 8.010100 |
| C | -0.370500 | 2.391600 | 10.265300 |
| O | 1.955800 | 4.178600 | 10.021200 |
| O | 3.216500 | 2.747100 | 6.003600 |
| C | 1.370200 | -2.672000 | 5.290500 |
| O | 0.908900 | -3.424100 | 6.407800 |
| O | 0.327800 | -0.016400 | 2.841000 |
| C | -0.893100 | -1.556900 | 4.960900 |
| O | 1.990300 | 1.870800 | 3.783700 |
| C | -2.104100 | -1.717800 | 4.924200 |
| H | 2.900000 | 0.346300 | 4.881400 |
| H | 0.050600 | 1.243900 | 5.472300 |
| H | 1.959500 | -1.106400 | 3.333500 |
| H | 3.250700 | 3.992700 | 8.017200 |
| H | -0.299800 | 0.882400 | 7.922300 |
| H | -1.153500 | 1.643400 | 10.139400 |
| H | 0.163500 | 2.165500 | 11.190200 |
| H | -0.851500 | 3.363100 | 10.392600 |
| H | 1.319400 | -3.316100 | 4.410400 |
| H | 2.427100 | -2.446700 | 5.442700 |
| H | 0.826500 | -2.822200 | 7.154900 |
| H | 0.680100 | 0.858800 | 2.619200 |
| H | 2.572100 | 2.470800 | 4.283800 |
| H | -3.159400 | -1.867800 | 4.876700 |

LNA-OpenAlkynyl-Diol_2 Eopt
-1026.175992

| | | | |
|---|---|---|---|
| C | 1.922800 | 0.701400 | 4.517800 |
| C | 1.029800 | 0.783300 | 5.752700 |
| O | 0.821800 | -0.527000 | 6.200400 |
| C | 1.028300 | -1.488500 | 5.163400 |
| C | 1.442200 | -0.627200 | 3.919900 |
| N | 1.439400 | 1.672100 | 6.835100 |
| C | 2.580000 | 1.346200 | 7.549800 |
| N | 2.907800 | 2.252000 | 8.514800 |
| C | 2.302400 | 3.414500 | 8.866700 |
| C | 1.118700 | 3.724500 | 8.057900 |
| C | 0.758100 | 2.845600 | 7.096400 |
| C | 0.367000 | 5.021100 | 8.335600 |
| O | 2.754100 | 4.095100 | 9.783500 |
| O | 3.294000 | 0.351300 | 7.402000 |
| C | 2.139800 | -2.449100 | 5.684300 |
| O | 3.425500 | -1.847900 | 5.755900 |
| O | 0.471400 | -0.409500 | 2.881600 |
| C | -0.253200 | -2.222100 | 5.005500 |
| O | 1.733100 | 1.858000 | 3.719200 |
| C | -1.302600 | -2.835600 | 4.878600 |
| H | 2.966400 | 0.611700 | 4.825500 |

| | | | |
|---|---|---|---|
| H | 0.038400 | 1.131600 | 5.453800 |
| H | 2.297900 | -1.103800 | 3.435900 |
| H | 3.727000 | 2.029300 | 9.052900 |
| H | -0.094200 | 3.024200 | 6.456000 |
| H | -0.483800 | 5.161200 | 7.668700 |
| H | -0.003600 | 5.033200 | 9.362200 |
| H | 1.031700 | 5.878600 | 8.215300 |
| H | 1.878700 | -2.836300 | 6.671800 |
| H | 2.229700 | -3.315800 | 5.026300 |
| H | 3.404500 | -1.141000 | 6.430700 |
| H | 0.084000 | -1.257300 | 2.639100 |
| H | 1.301800 | 1.545100 | 2.911700 |
| H | -2.224900 | -3.367000 | 4.790800 |

LNA-OpenAlkynyl-Diol_3 Eopt
-1026.172414

| | | | |
|---|---|---|---|
| C | 1.913700 | 0.667400 | 4.534600 |
| C | 1.014300 | 0.811800 | 5.770900 |
| O | 0.846500 | -0.521500 | 6.165400 |
| C | 0.606300 | -1.356400 | 5.023400 |
| C | 1.212200 | -0.516200 | 3.846400 |
| N | 1.359700 | 1.630600 | 6.936700 |
| C | 2.392900 | 2.555500 | 6.900900 |
| N | 2.479700 | 3.341600 | 8.012100 |
| C | 1.693300 | 3.389900 | 9.116200 |
| C | 0.588700 | 2.427700 | 9.085300 |
| C | 0.486100 | 1.618300 | 8.008600 |
| C | -0.377800 | 2.388100 | 10.262800 |
| O | 1.937100 | 4.188800 | 10.015500 |
| O | 3.209600 | 2.756600 | 6.002900 |
| C | 1.357400 | -2.691300 | 5.288300 |
| O | 2.745800 | -2.495700 | 5.528300 |
| O | 0.327000 | -0.021200 | 2.842100 |
| C | -0.860500 | -1.594300 | 4.954700 |
| O | 1.983200 | 1.876900 | 3.782900 |
| C | -2.066400 | -1.789200 | 4.915300 |
| H | 2.904400 | 0.353400 | 4.874500 |
| H | 0.051100 | 1.239400 | 5.469400 |
| H | 1.974400 | -1.102600 | 3.328800 |
| H | 3.233700 | 4.006800 | 8.011100 |
| H | -0.298200 | 0.875600 | 7.922400 |
| H | -1.155200 | 1.633900 | 10.138800 |
| H | 0.158200 | 2.168100 | 11.188000 |
| H | -0.865700 | 3.356500 | 10.387900 |
| H | 0.933000 | -3.210400 | 6.150700 |
| H | 1.244900 | -3.362700 | 4.434300 |
| H | 2.835800 | -1.991800 | 6.344400 |
| H | 0.690600 | 0.844100 | 2.601800 |
| H | 2.572500 | 2.473200 | 4.277900 |
| H | -3.119700 | -1.952400 | 4.861200 |

LNA-OpenAlkynyl-Diol_4 Eopt
-1026.174018

| | | | |
|---|---|---|---|
| C | 1.929900 | 0.709500 | 4.507100 |
| C | 1.029500 | 0.779200 | 5.753700 |
| O | 0.815200 | -0.531000 | 6.210100 |
| C | 1.234100 | -1.519200 | 5.271300 |
| C | 2.342300 | -0.773500 | 4.464800 |
| N | 1.462700 | 1.640900 | 6.849700 |
| C | 2.589100 | 1.276300 | 7.568600 |
| N | 2.950500 | 2.174100 | 8.528900 |
| C | 2.390300 | 3.361300 | 8.872700 |
| C | 1.215100 | 3.707600 | 8.066200 |
| C | 0.822500 | 2.837800 | 7.108900 |
| C | 0.505900 | 5.027800 | 8.343500 |
| O | 2.872900 | 4.033700 | 9.779300 |
| O | 3.263300 | 0.254600 | 7.425700 |
| C | 1.739100 | -2.724000 | 6.120400 |
| O | 2.974600 | -2.490600 | 6.786500 |
| O | 2.590100 | -1.202600 | 3.118200 |
| C | 0.051900 | -1.923200 | 4.462700 |
| O | 1.184100 | 1.120700 | 3.371300 |
| C | -0.927200 | -2.244600 | 3.805600 |
| H | 2.812900 | 1.341400 | 4.627500 |
| H | 0.033600 | 1.137800 | 5.482600 |
| H | 3.284900 | -0.832500 | 5.010500 |
| H | 3.760200 | 1.924300 | 9.070100 |
| H | -0.030300 | 3.040600 | 6.475100 |
| H | -0.344900 | 5.192300 | 7.682000 |
| H | 0.141600 | 5.054700 | 9.372200 |
| H | 1.195500 | 5.864600 | 8.217200 |
| H | 0.988100 | -2.999500 | 6.864000 |
| H | 1.881900 | -3.604100 | 5.490100 |
| H | 2.946100 | -1.615300 | 7.212400 |
| H | 2.958900 | -2.091600 | 3.144300 |
| H | 1.548000 | 0.616800 | 2.629800 |
| H | -1.785200 | -2.509900 | 3.229000 |

LNA-OpenAlkynyl-Diol_5 Eopt
-1026.171964

| | | | |
|---|---|---|---|
| C | 1.920200 | 0.688800 | 4.523100 |
| C | 1.023600 | 0.794600 | 5.760100 |
| O | 0.830800 | -0.525800 | 6.187800 |
| C | 0.882000 | -1.463200 | 5.109000 |
| C | 1.399700 | -0.615200 | 3.901700 |
| N | 1.492400 | 1.652300 | 6.850200 |
| C | 1.441200 | 3.020900 | 6.656000 |
| N | 2.003200 | 3.754100 | 7.658700 |
| C | 2.676200 | 3.347300 | 8.764400 |
| C | 2.794300 | 1.889700 | 8.871000 |
| C | 2.208400 | 1.134800 | 7.914900 |
| C | 3.575600 | 1.305800 | 10.042000 |
| O | 3.125700 | 4.173500 | 9.553300 |
| O | 0.980700 | 3.598000 | 5.670800 |
| C | 1.863800 | -2.588000 | 5.552300 |
| O | 3.209700 | -2.141800 | 5.713100 |
| O | 0.493200 | -0.319300 | 2.839100 |
| C | -0.491700 | -2.010000 | 4.959800 |
| O | 1.814600 | 1.848400 | 3.692400 |
| C | -1.622700 | -2.460900 | 4.853000 |
| H | 2.943800 | 0.548800 | 4.880300 |
| H | 0.032200 | 1.185400 | 5.501100 |
| H | 2.246800 | -1.123800 | 3.437700 |
| H | 1.944900 | 4.752300 | 7.552300 |
| H | 2.266800 | 0.054700 | 7.931700 |
| H | 3.612500 | 0.216600 | 10.014300 |
| H | 4.602000 | 1.677000 | 10.038700 |
| H | 3.120100 | 1.602900 | 10.988400 |
| H | 1.529200 | -3.029400 | 6.493900 |
| H | 1.861800 | -3.394000 | 4.815200 |
| H | 3.754300 | -2.875600 | 6.014600 |
| H | 0.723700 | 0.582400 | 2.570100 |
| H | 1.708200 | 2.639000 | 4.249300 |
| H | -2.615600 | -2.836600 | 4.745200 |

LNA-OpenAlkynyl-Diol_6 Eopt
-1026.171960

| | | | |
|---|---|---|---|
| C | 1.914600 | 0.669700 | 4.533100 |
| C | 1.015100 | 0.809900 | 5.770000 |
| O | 0.844800 | -0.521900 | 6.167800 |
| C | 0.670700 | -1.382900 | 5.035600 |
| C | 1.224300 | -0.520800 | 3.850400 |
| N | 1.364000 | 1.628600 | 6.935500 |
| C | 2.365800 | 2.587200 | 6.882600 |
| N | 2.459100 | 3.362400 | 8.000800 |
| C | 1.698700 | 3.378300 | 9.123800 |
| C | 0.620900 | 2.386200 | 9.109300 |
| C | 0.515900 | 1.583900 | 8.027500 |
| C | -0.316100 | 2.308800 | 10.308700 |
| O | 1.940900 | 4.176300 | 10.024800 |
| O | 3.148700 | 2.832400 | 5.965300 |
| C | 1.497400 | -2.671500 | 5.322800 |
| O | 2.907800 | -2.460900 | 5.324600 |
| O | 0.305900 | -0.039600 | 2.868200 |
| C | -0.778800 | -1.708000 | 4.963800 |
| O | 1.967100 | 1.874700 | 3.771900 |
| C | -1.970800 | -1.974900 | 4.921300 |
| H | 2.907900 | 0.366800 | 4.876300 |
| H | 0.048600 | 1.231600 | 5.473300 |
| H | 1.985100 | -1.083800 | 3.306700 |
| H | 3.193400 | 4.048900 | 7.987000 |
| H | -0.248100 | 0.819400 | 7.953300 |
| H | -1.073700 | 1.533200 | 10.195200 |
| H | 0.248000 | 2.096100 | 11.218600 |
| H | -0.828600 | 3.261500 | 10.453800 |
| H | 1.204100 | -3.096400 | 6.285200 |
| H | 1.278400 | -3.431500 | 4.569400 |
| H | 3.349600 | -3.253100 | 5.644700 |
| H | 0.649800 | 0.833600 | 2.627900 |
| H | 2.537700 | 2.484300 | 4.273300 |
| H | -3.013500 | -2.195200 | 4.866900 |

LNA-OpenAlkynyl-Diol_7 Eopt
-1026.163066

| | | | |
|---|---|---|---|
| C | 1.915700 | 0.681400 | 4.530100 |
| C | 1.022000 | 0.798000 | 5.761900 |
| O | 0.836800 | -0.521700 | 6.178900 |
| C | 0.729400 | -1.398300 | 5.050800 |
| C | 1.259800 | -0.531600 | 3.853700 |
| N | 1.512500 | 1.623600 | 6.860100 |
| C | 0.797200 | 2.742100 | 7.250200 |
| N | 1.361100 | 3.426300 | 8.286600 |
| C | 2.490100 | 3.169000 | 8.995600 |
| C | 3.184700 | 1.950800 | 8.564900 |
| C | 2.659500 | 1.254100 | 7.533200 |
| C | 4.445600 | 1.527700 | 9.308800 |
| O | 2.840000 | 3.921400 | 9.901000 |
| O | -0.252900 | 3.163100 | 6.766500 |
| C | 1.621100 | -2.632400 | 5.378000 |
| O | 3.016300 | -2.333300 | 5.429300 |
| O | 0.336700 | -0.104100 | 2.849400 |
| C | -0.698000 | -1.801500 | 4.953900 |
| O | 1.924400 | 1.914000 | 3.817100 |
| C | -1.874600 | -2.125700 | 4.890700 |
| H | 2.922100 | 0.410700 | 4.856900 |
| H | 0.040100 | 1.188600 | 5.469600 |
| H | 2.033400 | -1.090300 | 3.324000 |
| H | 0.863800 | 4.251100 | 8.573700 |
| H | 3.095000 | 0.331600 | 7.170900 |
| H | 4.864200 | 0.599000 | 8.920700 |
| H | 5.210500 | 2.302100 | 9.229800 |
| H | 4.230800 | 1.382500 | 10.369100 |
| H | 1.320000 | -3.067600 | 6.333700 |
| H | 1.473800 | -3.409700 | 4.624800 |
| H | 3.492700 | -3.110000 | 5.739500 |
| H | 0.187600 | 0.838700 | 2.996900 |
| H | 2.594900 | 1.875100 | 3.126400 |
| H | -2.906900 | -2.389600 | 4.830700 |

LNA-OpenAlkynyl-Diol_8 Eopt
-1026.173089

| | | | |
|---|---|---|---|
| C | 1.929700 | 0.705200 | 4.507800 |
| C | 1.026700 | 0.783800 | 5.757000 |
| O | 0.818100 | -0.531300 | 6.206100 |
| C | 1.128600 | -1.505800 | 5.207400 |
| C | 2.277800 | -0.796200 | 4.444100 |
| N | 1.541600 | 1.593800 | 6.856200 |
| C | 0.905300 | 2.777700 | 7.181100 |
| N | 1.450800 | 3.426000 | 8.249900 |
| C | 2.526600 | 3.103600 | 9.013000 |
| C | 3.176800 | 1.852600 | 8.606800 |
| C | 2.652800 | 1.176900 | 7.560600 |
| C | 4.398900 | 1.378900 | 9.383800 |
| O | 2.871700 | 3.834600 | 9.936600 |
| O | -0.050500 | 3.283100 | 6.595000 |
| C | 1.584800 | -2.796900 | 5.945400 |
| O | 2.517400 | -2.532200 | 6.989300 |
| O | 2.493400 | -1.184000 | 3.091300 |
| C | -0.094500 | -1.772300 | 4.399700 |
| O | 1.206300 | 1.182700 | 3.370700 |
| C | -1.105100 | -1.977300 | 3.744000 |
| H | 2.838700 | 1.293900 | 4.653500 |
| H | 0.032500 | 1.165300 | 5.496800 |
| H | 3.204000 | -0.950900 | 5.001300 |
| H | 0.998600 | 4.286000 | 8.508900 |
| H | 3.069800 | 0.242800 | 7.207600 |
| H | 4.794600 | 0.436100 | 9.004500 |
| H | 5.195900 | 2.122900 | 9.331300 |
| H | 4.149900 | 1.238000 | 10.437200 |
| H | 0.732100 | -3.312800 | 6.392800 |
| H | 2.032400 | -3.495200 | 5.234900 |
| H | 2.044200 | -2.059600 | 7.682600 |
| H | 1.887300 | -0.633800 | 2.575200 |
| H | 1.094300 | 2.138200 | 3.434100 |
| H | -1.987100 | -2.147700 | 3.167400 |

LNA-OpenAlkynyl-Diol_9 Eopt
-1026.171038

| | | | |
|---|---|---|---|
| C | 1.927000 | 0.697700 | 4.512400 |
| C | 1.023800 | 0.789300 | 5.760400 |
| O | 0.823700 | -0.529400 | 6.198100 |
| C | 0.919800 | -1.466600 | 5.126500 |
| C | 2.076200 | -0.824800 | 4.310900 |
| N | 1.481100 | 1.666200 | 6.834300 |
| C | 1.527400 | 3.018600 | 6.542600 |
| N | 2.134300 | 3.778300 | 7.498200 |
| C | 2.762200 | 3.403600 | 8.641400 |
| C | 2.760200 | 1.953200 | 8.860000 |
| C | 2.132800 | 1.174800 | 7.950000 |
| C | 3.467100 | 1.401300 | 10.092100 |
| O | 3.269600 | 4.249500 | 9.371900 |
| O | 1.108400 | 3.551200 | 5.512400 |
| C | 1.250100 | -2.860900 | 5.742400 |
| O | 2.527000 | -2.956200 | 6.370700 |
| O | 2.137700 | -1.129900 | 2.922700 |
| C | -0.388900 | -1.530800 | 4.414000 |
| O | 1.291600 | 1.394100 | 3.440500 |
| C | -1.464700 | -1.567400 | 3.835400 |
| H | 2.904500 | 1.137000 | 4.726700 |
| H | 0.024500 | 1.173800 | 5.522700 |
| H | 3.025400 | -1.104800 | 4.768400 |
| H | 2.153500 | 4.767700 | 7.319700 |
| H | 2.095000 | 0.097900 | 8.054500 |
| H | 3.413300 | 0.313600 | 10.148700 |
| H | 4.521000 | 1.685200 | 10.087600 |
| H | 3.019600 | 1.806900 | 11.001200 |
| H | 0.492500 | -3.122000 | 6.484600 |
| H | 1.202600 | -3.633900 | 4.972100 |
| H | 3.188500 | -3.175800 | 5.707400 |
| H | 1.652600 | -0.409000 | 2.493900 |
| H | 1.231400 | 2.331400 | 3.682000 |
| H | -2.402000 | -1.591600 | 3.325400 |

**Cartesian coordinates for compound 19**

LNA-OpenmonoCl-Acet_1
Eopt -1565.650817

| | | | |
|---|---|---|---|
| C | 2.282800 | -3.115800 | 2.089100 |
| C | 1.056900 | -4.000400 | 2.467800 |
| O | -0.102000 | -3.209100 | 2.263900 |
| C | 0.240000 | -1.828800 | 2.359900 |
| C | 1.528300 | -1.900000 | 1.567000 |
| N | 0.892000 | -5.301600 | 1.818600 |
| C | 0.889800 | -5.364400 | 0.434900 |
| N | 0.691400 | -6.615700 | -0.067500 |
| C | 0.505200 | -7.797400 | 0.572000 |
| C | 0.513000 | -7.676100 | 2.033200 |
| C | 0.703600 | -6.449600 | 2.568300 |
| C | 0.300000 | -8.933200 | 2.868200 |
| O | 0.353500 | -8.833000 | -0.070000 |
| O | 1.055800 | -4.438800 | -0.357700 |
| C | -0.631000 | -0.849400 | 1.560100 |
| O | 0.051100 | 0.419200 | 1.462100 |
| C | 1.397700 | 0.404500 | 1.018600 |
| O | 2.176400 | -0.628200 | 1.603000 |
| C | 0.351400 | -1.402300 | 3.854500 |
| O | 3.083800 | -2.850500 | 3.231500 |
| Cl | -1.216400 | -1.535900 | 4.701900 |
| C | 1.407000 | 0.272300 | -0.504200 |
| C | 2.000600 | 1.696700 | 1.521100 |
| H | 2.877700 | -3.571100 | 1.294300 |
| H | 1.099000 | -4.218400 | 3.536800 |
| H | 1.324700 | -2.121500 | 0.519200 |
| H | 0.685800 | -6.676900 | -1.070800 |
| H | 0.707100 | -6.301800 | 3.639400 |
| H | 0.314300 | -8.731600 | 3.939400 |
| H | -0.661000 | -9.391300 | 2.626700 |
| H | 1.077600 | -9.668700 | 2.653900 |
| H | -0.879100 | -1.231800 | 0.569200 |
| H | -1.592300 | -0.696900 | 2.050200 |
| H | 1.057500 | -2.005800 | 4.418800 |
| H | 0.685100 | -0.367900 | 3.937900 |
| H | 3.714400 | -2.161800 | 2.990500 |
| H | 0.815600 | -0.570700 | -0.854100 |
| H | 0.985900 | 1.174100 | -0.948300 |
| H | 2.431500 | 0.156900 | -0.857200 |
| H | 1.955500 | 1.726600 | 2.609700 |
| H | 3.041700 | 1.769800 | 1.207600 |
| H | 1.447000 | 2.547300 | 1.123800 |

LNA-OpenmonoCl-Acet_2
Eopt -1565.647687

| | | | |
|---|---|---|---|
| C | 2.266503 | -3.126928 | 2.145839 |
| C | 1.084330 | -3.994168 | 2.691156 |
| O | -0.123101 | -3.248184 | 2.510738 |
| C | 0.235958 | -1.885657 | 2.396105 |
| C | 1.483347 | -1.989918 | 1.513020 |
| N | 0.915972 | -5.270315 | 2.023092 |
| C | 1.325752 | -6.417703 | 2.699576 |
| N | 1.167530 | -7.574750 | 1.963882 |
| C | 0.666063 | -7.722910 | 0.666318 |
| C | 0.244727 | -6.466264 | 0.044214 |
| C | 0.385651 | -5.329273 | 0.747828 |
| C | -0.339383 | -6.531487 | -1.333690 |
| O | 0.600976 | -8.820487 | 0.147572 |
| O | 1.787917 | -6.407492 | 3.821830 |
| C | -0.717087 | -1.013887 | 1.584570 |
| O | -0.028038 | 0.217372 | 1.380526 |
| C | 1.240747 | 0.198843 | 0.748824 |
| O | 2.128997 | -0.765124 | 1.355721 |
| C | 0.502913 | -1.260270 | 3.770418 |
| O | 3.074812 | -2.715233 | 3.220338 |
| Cl | -0.972603 | -1.296079 | 4.796894 |
| C | 1.110067 | -0.065979 | -0.751999 |
| C | 1.861397 | 1.553284 | 1.031028 |
| H | 2.854281 | -3.695202 | 1.414122 |
| H | 1.267191 | -4.217319 | 3.744285 |
| H | 1.131130 | -2.366559 | 0.540330 |
| H | 1.460391 | -8.423220 | 2.431819 |
| H | 0.051795 | -4.373997 | 0.360505 |
| H | -0.617743 | -5.536612 | -1.692980 |
| H | 0.376055 | -6.971489 | -2.035691 |
| H | -1.228718 | -7.169558 | -1.346540 |
| H | -0.975484 | -1.512536 | 0.639959 |
| H | -1.636079 | -0.756536 | 2.112318 |
| H | 1.280223 | -1.798356 | 4.308630 |
| H | 0.793791 | -0.217272 | 3.659747 |
| H | 3.542869 | -1.917714 | 2.937109 |
| H | 0.617713 | -1.015697 | -0.976590 |
| H | 0.513023 | 0.730215 | -1.203424 |
| H | 2.100660 | -0.075759 | -1.213647 |
| H | 1.949765 | 1.695527 | 2.110577 |
| H | 2.854966 | 1.610908 | 0.579430 |
| H | 1.231921 | 2.345187 | 0.619043 |

LNA-OpenmonoCl-Acet_3
Eopt -1565.650451

| | | | |
|---|---|---|---|
| C | 2.185800 | -3.024100 | 1.749700 |
| C | 1.193500 | -3.909600 | 2.558000 |
| O | -0.010900 | -3.165100 | 2.674200 |
| C | 0.277600 | -1.771300 | 2.575400 |
| C | 1.234300 | -1.881400 | 1.409900 |
| N | 0.968800 | -5.264900 | 2.061400 |
| C | 2.070400 | -6.103600 | 2.053100 |
| N | 1.871900 | -7.286100 | 1.405000 |
| C | 0.792100 | -7.730900 | 0.713700 |
| C | -0.322500 | -6.777400 | 0.695400 |
| C | -0.169800 | -5.608700 | 1.357500 |
| C | -1.589400 | -7.153600 | -0.063300 |
| O | 0.815200 | -8.832100 | 0.172000 |
| O | 3.174500 | -5.850200 | 2.538100 |
| C | -0.863600 | -0.886400 | 2.050700 |
| O | -0.332200 | 0.380900 | 1.616100 |
| C | 0.779300 | 0.354700 | 0.738000 |
| O | 1.766600 | -0.594100 | 1.101800 |
| C | 0.856800 | -1.223100 | 3.913000 |
| O | 3.304600 | -2.713000 | 2.568000 |
| Cl | -0.333900 | -1.330400 | 5.241700 |
| C | 0.275800 | 0.069600 | -0.676300 |
| C | 1.443800 | 1.704100 | 0.888700 |
| H | 2.503300 | -3.526600 | 0.832800 |
| H | 1.538900 | -4.079600 | 3.584900 |
| H | 0.707700 | -2.223100 | 0.516200 |
| H | 2.654000 | -7.918100 | 1.408400 |

H -0.958800 -4.870800 1.391800
H -2.354600 -6.379100 -0.007700
H -1.365100 -7.330600 -1.116700
H -2.015100 -8.073900 0.341100
H -1.418000 -1.364200 1.242700
H -1.604200 -0.707300 2.829800
H 1.742500 -1.757000 4.247100
H 1.146600 -0.177200 3.807500
H 3.856400 -3.502500 2.645000
H -0.342100 -0.823100 -0.727100
H -0.328300 0.909100 -1.020100
H 1.124700 -0.045500 -1.350000
H 1.773700 1.838500 1.919000
H 2.310800 1.768000 0.231500
H 0.742400 2.499200 0.636800

LNA-OpenmonoCl-Acet_4
Eopt -1565.641515
C 2.160200 -3.163400 2.262800
C 0.928100 -4.086200 2.501300
O -0.231100 -3.300400 2.246100
C 0.079800 -1.914200 2.411700
C 1.417700 -1.963900 1.700200
N 0.844800 -5.365900 1.793000
C 0.938900 -5.378400 0.411100
N 0.815700 -6.615300 -0.147600
C 0.625700 -7.825200 0.434900
C 0.537800 -7.759500 1.896800
C 0.651000 -6.548700 2.486800
C 0.321300 -9.054500 2.670900
O 0.545000 -8.839500 -0.252200
O 1.126000 -4.422800 -0.338500
C -0.756200 -0.937800 1.566700
O -0.077900 0.328200 1.422600
C 1.283500 0.302200 1.027100
O 2.048800 -0.685300 1.703600
C 0.012500 -1.609700 3.945200
O 2.824800 -2.893200 3.487300
Cl 0.600000 0.007000 4.423900
C 1.346400 0.081500 -0.486100
C 1.861500 1.632500 1.457500
H 2.843800 -3.581400 1.520300
H 0.898700 -4.353800 3.558600
H 1.272500 -2.208300 0.649500
H 0.879100 -6.638200 -1.150600
H 0.586800 -6.445900 3.561000
H 0.267200 -8.895300 3.747900
H -0.606900 -9.534900 2.355800
H 1.135600 -9.754800 2.475700
H -0.987100 -1.350900 0.584000
H -1.725000 -0.763700 2.034300
H -1.019300 -1.682600 4.286700
H 0.574900 -2.324200 4.541000
H 3.340700 -2.086600 3.361500
H 0.749900 -0.767400 -0.812100
H 0.961000 0.963800 -0.996600
H 2.380900 -0.072600 -0.792100
H 1.789800 1.737700 2.539300
H 2.909900 1.694800 1.167800
H 1.310800 2.448900 0.991200

**Cartesian coordinates for compound 19’**

LNA-OpenmonoCl-Diol_1
Eopt -1448.952948
C -2.455100 0.800000 -0.711400
C -2.013300 0.635200 0.745300
O -2.249100 1.856300 1.387300
C -2.339500 2.943600 0.461300
C -2.082500 2.272400 -0.922800
N -2.542700 -0.489700 1.511000
C -3.902500 -0.519500 1.772300
N -4.310700 -1.618800 2.467600
C -3.600700 -2.684400 2.916700
C -2.171600 -2.611300 2.594600
C -1.727600 -1.530200 1.915500
C -1.267000 -3.755600 3.036500
O -4.162600 -3.592100 3.523100
O -4.744100 0.318600 1.441400
C -3.769800 3.549600 0.606300
O -4.807500 2.718500 0.103700
O -0.752900 2.370800 -1.431300
C -1.236800 3.956300 0.871100
O -1.797300 -0.160600 -1.534900
Cl -1.477500 4.648400 2.503800
H -3.537100 0.674100 -0.770100
H -0.927700 0.516500 0.775500
H -2.750700 2.706100 -1.670200
H -5.293200 -1.654300 2.677900
H -0.686700 -1.422400 1.644500
H -0.228400 -3.605600 2.741500
H -1.294900 -3.863600 4.122500
H -1.606400 -4.698600 2.603900
H -3.984200 3.774600 1.652400
H -3.819600 4.499100 0.071400
H -4.886300 1.939200 0.687700
H -0.538400 1.495100 -1.781300
H -0.257400 3.478200 0.872800
H -1.188600 4.783700 0.163800
H -2.327200 -0.308300 -2.327400

LNA-OpenmonoCl-Diol_10
Eopt -1448.951828
C -2.472000 0.794800 -0.706700
C -1.983900 0.649500 0.738500
O -2.282400 1.851000 1.387500
C -2.318600 2.939700 0.463200
C -2.153300 2.276600 -0.939900
N -2.435000 -0.499600 1.515100
C -3.784900 -0.595200 1.808800
N -4.124100 -1.720100 2.500200
C -3.352600 -2.753400 2.923200
C -1.934700 -2.603600 2.577900
C -1.559100 -1.496900 1.898700
C -0.963300 -3.698900 3.001400
O -3.857200 -3.695800 3.526400
O -4.668700 0.209600 1.505800
C -3.719000 3.589700 0.665000
O -4.801800 2.746300 0.291600
O -0.894400 2.424500 -1.584400
C -1.188800 3.921600 0.886500
O -1.814800 -0.147400 -1.553300
Cl 0.389100 3.131600 1.205600
H -3.554100 0.655600 -0.740400
H -0.892000 0.591800 0.740900
H -2.899100 2.683100 -1.626800
H -5.098900 -1.806300 2.731000
H -0.528100 -1.327100 1.618200
H 0.062200 -3.489600 2.696200
H -0.971700 -3.814700 4.086900
H -1.254600 -4.657000 2.567000
H -3.852100 3.882600 1.708100
H -3.789000 4.504700 0.074700
H -4.800500 1.955500 0.862800
H -0.709600 1.562500 -1.983300
H -1.026600 4.675200 0.115900
H -1.455800 4.442600 1.805300
H -2.331000 -0.960500 -1.586100

LNA-OpenmonoCl-Diol_11
Eopt -1448.956235
C -2.471100 0.801700 -0.698400
C -1.982700 0.639200 0.740400
O -2.282800 1.848500 1.379300
C -2.320400 2.945500 0.461100
C -2.115800 2.273700 -0.928600
N -2.538100 -0.484100 1.486600
C -1.741700 -1.580600 1.759700
N -2.367900 -2.556200 2.478000
C -3.646600 -2.626600 2.929400
C -4.449300 -1.444500 2.596300
C -3.854600 -0.451400 1.899200
C -5.900700 -1.394200 3.057400
O -4.032900 -3.616300 3.544600
O -0.580400 -1.756900 1.397100
C -3.715400 3.625100 0.621200
O -4.806800 2.855300 0.121400
O -0.813000 2.381500 -1.496300
C -1.157700 3.889800 0.869500
O -1.832300 -0.134900 -1.565600
Cl -1.370500 4.620000 2.490000
H -3.556200 0.676100 -0.711300
H -0.890400 0.540700 0.755700
H -2.816100 2.691500 -1.654700
H -1.803200 -3.359900 2.693100
H -4.370400 0.461900 1.628000
H -6.397000 -0.468600 2.765000
H -6.464400 -2.227800 2.635100
H -5.956400 -1.475400 4.144600
H -3.903300 3.846600 1.673200
H -3.712300 4.585300 0.102800
H -5.629400 3.316200 0.317700
H -0.665900 1.532800 -1.941400
H -0.212400 3.346600 0.885400
H -1.046500 4.701700 0.151400
H -2.127800 -1.028500 -1.359000

LNA-OpenmonoCl-Diol_12
Eopt -1448.952123
C -2.424300 0.800300 -0.728400
C -2.070000 0.621200 0.757300
O -2.186300 1.870200 1.387400
C -2.376400 2.943300 0.466200
C -2.989300 2.228200 -0.771500
N -2.780900 -0.413100 1.506000
C -4.129500 -0.233800 1.762100
N -4.717700 -1.278500 2.411100
C -4.192300 -2.459100 2.825000
C -2.767900 -2.606700 2.508400
C -2.149200 -1.587200 1.871900
C -2.064900 -3.899500 2.905000
O -4.897300 -3.282700 3.400600
O -4.815500 0.746100 1.469900
C -3.323700 3.950400 1.187100
O -4.671300 3.514200 1.306000

O -2.767800 2.842300 -2.043900
C -0.954900 3.530000 0.250800
O -1.247500 0.638100 -1.507200
Cl -0.954400 5.032000 -0.722800
H -3.188500 0.083000 -1.037300
H -1.013300 0.365000 0.863600
H -4.067200 2.132300 -0.631700
H -5.695400 -1.163600 2.616100
H -1.102900 -1.642700 1.603500
H -1.014500 -3.911600 2.613400
H -2.114900 -4.043100 3.986000
H -2.552300 -4.757200 2.437500
H -2.937400 4.174600 2.182800
H -3.347700 4.899700 0.651300
H -4.688200 2.583000 1.593200
H -3.051300 3.763100 -2.000200
H -0.490500 3.767600 1.207900
H -0.302900 2.814500 -0.250000
H -1.413000 1.112400 -2.334400

LNA-OpenmonoCl-Diol_13
Eopt -1448.956786
C -2.391100 0.816500 -0.729100
C -2.127600 0.601400 0.774000
O -2.122200 1.875300 1.371700
C -2.416100 2.941800 0.465900
C -2.042500 2.300000 -0.892400
N -2.977000 -0.359000 1.487000
C -2.959300 -1.669300 1.037700
N -3.901100 -2.475000 1.605500
C -4.885500 -2.177400 2.489900
C -4.899000 -0.768300 2.893000
C -3.958700 0.050100 2.370800
C -5.969200 -0.299800 3.871400
O -5.659200 -3.052000 2.868200
O -2.210600 -2.136100 0.177400
C -3.940900 3.284800 0.529800
O -4.434700 3.521600 1.839300
O -0.690000 2.454900 -1.307900
C -1.493300 4.131500 0.856100
O -1.597800 -0.072200 -1.513200
Cl -1.817000 4.807200 2.482400
H -3.456500 0.654400 -0.909600
H -1.119000 0.208500 0.943500
H -2.660200 2.722000 -1.687900
H -3.890000 -3.434800 1.305800
H -3.914800 1.094100 2.645500
H -5.900900 0.769300 4.077300
H -6.965600 -0.499200 3.473300
H -5.878500 -0.831800 4.819900
H -4.145200 4.163400 -0.084300
H -4.541300 2.476700 0.110900
H -3.791800 4.067400 2.314500
H -0.457400 1.603800 -1.711600
H -0.449100 3.816700 0.855300
H -1.579100 4.944000 0.135100
H -1.768000 -0.983600 -1.211300

LNA-OpenmonoCl-Diol_14
Eopt -1448.952049
C -2.466300 0.792500 -0.715400
C -1.995100 0.641900 0.745200
O -2.268500 1.856400 1.385400
C -2.327100 2.944300 0.467300
C -2.930200 2.258100 -0.780400
N -2.523700 -0.501900 1.482600
C -2.131400 -1.752100 1.036000
N -2.785600 -2.790400 1.629700
C -3.809900 -2.780600 2.520000
C -4.249900 -1.430800 2.889000
C -3.593600 -0.379500 2.349100
C -5.423200 -1.284600 3.850300
O -4.282400 -3.837400 2.928500
O -1.298000 -1.975600 0.155000
C -3.247000 4.034900 1.092400
O -4.587700 3.607200 1.325200
O -2.567300 2.838600 -2.027100
C -0.877100 3.461100 0.242100
O -1.378600 0.462900 -1.579400
Cl -0.083700 4.000800 1.751800
H -3.317400 0.131900 -0.900300
H -0.908800 0.514400 0.823500
H -4.018500 2.256000 -0.694000
H -2.493900 -3.708800 1.342600
H -3.869400 0.642100 2.578400
H -5.674400 -0.241000 4.041800
H -6.311100 -1.774900 3.447100
H -5.191600 -1.755400 4.807400
H -2.829300 4.374100 2.041400
H -3.271700 4.906700 0.436500
H -5.070000 4.310400 1.770500
H -1.951200 2.201700 -2.421400
H -0.245000 2.689000 -0.195400
H -0.872800 4.302300 -0.451200
H -1.160600 -0.473500 -1.444200

LNA-OpenmonoCl-Diol_15
Eopt -1448.957039
C -2.374600 0.822600 -0.732700
C -2.158100 0.601100 0.780700
O -2.089500 1.878900 1.368000
C -2.434800 2.932400 0.466400
C -2.003700 2.303800 -0.879200
N -3.076300 -0.301600 1.483200
C -3.153200 -1.604400 1.018700
N -4.150500 -2.346100 1.578800
C -5.106200 -1.989900 2.473100
C -5.012900 -0.590400 2.898700
C -4.018100 0.165100 2.382800
C -6.039000 -0.060700 3.892900
O -5.941500 -2.810000 2.842300
O -2.438700 -2.114400 0.153900
C -3.976600 3.187500 0.544200
O -4.409400 3.731100 1.785000
O -0.626900 2.433600 -1.218000
C -1.597900 4.156500 0.913100
O -1.571800 -0.079900 -1.490500
Cl -1.989600 5.620800 -0.036000
H -3.435100 0.676300 -0.950700
H -1.179400 0.149100 0.975200
H -2.576500 2.744600 -1.697500
H -4.210600 -3.299900 1.265900
H -3.896000 1.199300 2.675100
H -5.890600 0.996600 4.116600
H -7.050000 -0.179100 3.499100
H -5.982400 -0.614700 4.831400
H -4.281500 3.868400 -0.252400
H -4.532300 2.265100 0.375400
H -4.176200 4.665500 1.817400
H -0.394100 1.592800 -1.639800
H -1.775500 4.383100 1.963800
H -0.528800 3.967000 0.813200
H -1.800600 -0.988400 -1.223400

LNA-OpenmonoCl-Diol_16
Eopt -1448.954443
C -2.488900 0.809300 -0.685700
C -1.948800 0.639000 0.743300
O -2.317800 1.838400 1.365200
C -2.301400 2.948400 0.459700
C -2.087200 2.267800 -0.924000
N -2.296800 -0.492900 1.609000
C -2.861400 -1.652600 1.098100
N -2.972300 -2.667800 2.001900
C -2.577100 -2.736100 3.297500
C -1.942000 -1.505900 3.776600
C -1.836300 -0.472000 2.913400
C -1.441400 -1.455400 5.215000
O -2.760500 -3.761600 3.946700
O -3.248800 -1.874100 -0.048800
C -3.700400 3.614600 0.590500
O -3.889500 4.670900 -0.342700
O -0.765800 2.323800 -1.459300
C -1.143800 3.884600 0.897000
O -1.921400 -0.134400 -1.589000
Cl -1.299300 4.450600 2.588900
H -3.578200 0.723800 -0.649300
H -0.855700 0.574100 0.715600
H -2.756000 2.702400 -1.670000
H -3.395600 -3.510500 1.653100
H -1.389000 0.471600 3.204000
H -0.996500 -0.492300 5.467000
H -2.262700 -1.636700 5.910800
H -0.689200 -2.227900 5.384600
H -4.484700 2.871400 0.438200
H -3.846400 3.995700 1.602300
H -4.683800 5.163400 -0.111900
H -0.683800 1.527100 -2.004000
H -0.182600 3.377800 0.820100
H -1.100200 4.761500 0.251000
H -2.345300 -0.989500 -1.393800

LNA-OpenmonoCl-Diol_17
Eopt -1448.954442
C -2.381000 0.823800 -0.731600
C -2.146100 0.599600 0.779900
O -2.101500 1.877900 1.370200
C -2.428200 2.933700 0.463900
C -2.003400 2.300800 -0.881700
N -3.035400 -0.323900 1.495300
C -3.150700 -1.610600 0.994300
N -4.135500 -2.355800 1.571200
C -5.038500 -2.020700 2.526100
C -4.888800 -0.646200 3.012700
C -3.910400 0.114800 2.474200
C -5.830200 -0.154100 4.105500
O -5.876900 -2.836200 2.897900
O -2.480800 -2.107800 0.088200
C -3.962400 3.203800 0.560900
O -4.421300 4.091800 -0.449600
O -0.626200 2.429100 -1.225300
C -1.581300 4.165400 0.874500
O -1.592400 -0.078800 -1.504600
Cl -1.950400 4.749800 2.527000
H -3.445800 0.688700 -0.936100
H -1.157000 0.165100 0.959800
H -2.578000 2.738500 -1.700500
H -4.228100 -3.295000 1.223600

| | | | |
|---|---|---|---|
| H | -3.737900 | 1.124700 | 2.818800 |
| H | -5.616400 | 0.870500 | 4.411200 |
| H | -6.866200 | -0.195400 | 3.764500 |
| H | -5.750500 | -0.788700 | 4.990300 |
| H | -4.525000 | 2.273900 | 0.474900 |
| H | -4.218500 | 3.607200 | 1.541300 |
| H | -5.306800 | 4.400700 | -0.231000 |
| H | -0.403900 | 1.593100 | -1.661600 |
| H | -0.517400 | 3.929200 | 0.853000 |
| H | -1.745300 | 4.990500 | 0.181700 |
| H | -1.833500 | -0.987200 | -1.247300 |

LNA-OpenmonoCl-Diol_18
Eopt -1448.946694

| | | | |
|---|---|---|---|
| C | -2.480600 | 0.802300 | -0.692700 |
| C | -1.965500 | 0.646600 | 0.737200 |
| O | -2.302100 | 1.843500 | 1.378100 |
| C | -2.308600 | 2.942600 | 0.460000 |
| C | -2.099700 | 2.266900 | -0.930500 |
| N | -2.456900 | -0.499800 | 1.493600 |
| C | -1.569700 | -1.471500 | 1.921600 |
| N | -2.155500 | -2.489100 | 2.615400 |
| C | -3.455500 | -2.683300 | 2.955800 |
| C | -4.343100 | -1.604100 | 2.509000 |
| C | -3.797300 | -0.583700 | 1.811500 |
| C | -5.822600 | -1.680900 | 2.865900 |
| O | -3.791700 | -3.686600 | 3.579100 |
| O | -0.354400 | -1.507200 | 1.733800 |
| C | -3.694800 | 3.643200 | 0.606000 |
| O | -4.788200 | 2.907300 | 0.061000 |
| O | -0.787600 | 2.350300 | -1.483300 |
| C | -1.135700 | 3.868100 | 0.882200 |
| O | -1.881300 | -0.165700 | -1.549400 |
| Cl | -1.362800 | 4.611500 | 2.494500 |
| H | -3.567100 | 0.694700 | -0.681300 |
| H | -0.870900 | 0.588400 | 0.727100 |
| H | -2.784800 | 2.698900 | -1.662700 |
| H | -1.527900 | -3.211700 | 2.922300 |
| H | -4.376700 | 0.264200 | 1.468700 |
| H | -6.382100 | -0.818100 | 2.504400 |
| H | -6.270700 | -2.578900 | 2.437000 |
| H | -5.949600 | -1.733800 | 3.948800 |
| H | -3.901300 | 3.841300 | 1.659300 |
| H | -3.664000 | 4.616500 | 0.113300 |
| H | -5.610100 | 3.347100 | 0.303900 |
| H | -0.542600 | 1.443200 | -1.718500 |
| H | -0.200400 | 3.308800 | 0.917600 |
| H | -0.998600 | 4.674400 | 0.162200 |
| H | -2.423900 | -0.276300 | -2.338000 |

LNA-OpenmonoCl-Diol_19
Eopt -1448.955902

| | | | |
|---|---|---|---|
| C | -2.468100 | 0.805200 | -0.700800 |
| C | -1.988300 | 0.637400 | 0.742100 |
| O | -2.276000 | 1.850200 | 1.383400 |
| C | -2.324500 | 2.942200 | 0.457800 |
| C | -2.075900 | 2.269300 | -0.924200 |
| N | -2.546000 | -0.483000 | 1.491900 |
| C | -1.722600 | -1.522200 | 1.885300 |
| N | -2.357000 | -2.491800 | 2.604500 |
| C | -3.658700 | -2.595500 | 2.976600 |
| C | -4.482700 | -1.463500 | 2.538600 |
| C | -3.884100 | -0.482200 | 1.828500 |
| C | -5.959100 | -1.446500 | 2.915100 |
| O | -4.045700 | -3.570400 | 3.614400 |
| O | -0.526700 | -1.655400 | 1.633800 |
| C | -3.736700 | 3.589700 | 0.604400 |
| O | -4.818900 | 2.756600 | 0.195500 |
| O | -0.746400 | 2.335100 | -1.433500 |
| C | -1.206800 | 3.903000 | 0.936200 |
| O | -1.850400 | -0.150200 | -1.562500 |
| Cl | -1.171300 | 5.432500 | 0.008700 |
| H | -3.554700 | 0.703700 | -0.725600 |
| H | -0.896400 | 0.534600 | 0.751400 |
| H | -2.736700 | 2.706800 | -1.675100 |
| H | -1.774900 | -3.257200 | 2.898100 |
| H | -4.415700 | 0.397700 | 1.488300 |
| H | -6.469400 | -0.553800 | 2.552900 |
| H | -6.468200 | -2.318200 | 2.500200 |
| H | -6.075200 | -1.482500 | 3.999900 |
| H | -3.896600 | 3.891000 | 1.641200 |
| H | -3.784700 | 4.504100 | 0.011100 |
| H | -5.643500 | 3.243800 | 0.297400 |
| H | -0.617000 | 1.504200 | -1.913200 |
| H | -1.340900 | 4.161000 | 1.986300 |
| H | -0.224300 | 3.438300 | 0.848400 |
| H | -2.217400 | -1.025400 | -1.398300 |

LNA-OpenmonoCl-Diol_2
Eopt -1448.953213

| | | | |
|---|---|---|---|
| C | -2.456600 | 0.803200 | -0.709900 |
| C | -2.010200 | 0.635100 | 0.744200 |
| O | -2.252200 | 1.856000 | 1.390300 |
| C | -2.338000 | 2.940600 | 0.457900 |
| C | -2.046100 | 2.266500 | -0.917800 |
| N | -2.520900 | -0.502200 | 1.504100 |
| C | -3.873800 | -0.538200 | 1.795700 |
| N | -4.266800 | -1.652900 | 2.475100 |
| C | -3.546600 | -2.727100 | 2.886100 |
| C | -2.126200 | -2.648600 | 2.529400 |
| C | -1.697600 | -1.552800 | 1.863900 |
| C | -1.213300 | -3.805100 | 2.919300 |
| O | -4.093600 | -3.644800 | 3.490700 |
| O | -4.720800 | 0.309300 | 1.506700 |
| C | -3.774400 | 3.535300 | 0.592600 |
| O | -4.813700 | 2.675200 | 0.146500 |
| O | -0.692900 | 2.321700 | -1.369500 |
| C | -1.265500 | 3.950300 | 0.937500 |
| O | -1.828500 | -0.175300 | -1.535500 |
| Cl | -1.236800 | 5.442300 | -0.048500 |
| H | -3.541100 | 0.699000 | -0.760800 |
| H | -0.923300 | 0.525500 | 0.768300 |
| H | -2.676100 | 2.718900 | -1.686800 |
| H | -5.244300 | -1.693400 | 2.706800 |
| H | -0.664600 | -1.442300 | 1.564700 |
| H | -0.183100 | -3.652000 | 2.597400 |
| H | -1.210300 | -3.938000 | 4.002800 |
| H | -1.568400 | -4.737000 | 2.475400 |
| H | -3.969100 | 3.812900 | 1.629900 |
| H | -3.850400 | 4.454900 | 0.010800 |
| H | -4.860400 | 1.903100 | 0.743300 |
| H | -0.517200 | 1.477200 | -1.803400 |
| H | -1.445900 | 4.244900 | 1.971100 |
| H | -0.268300 | 3.512100 | 0.901200 |
| H | -2.354200 | -0.292700 | -2.335800 |

LNA-OpenmonoCl-Diol_20
Eopt -1448.954416

| | | | |
|---|---|---|---|
| C | -2.428500 | 0.813700 | -0.714100 |
| C | -2.059500 | 0.621000 | 0.753100 |
| O | -2.198000 | 1.863000 | 1.388600 |
| C | -2.370800 | 2.937200 | 0.454800 |
| C | -1.994700 | 2.271800 | -0.900000 |
| N | -2.691300 | -0.469000 | 1.489700 |
| C | -4.024900 | -0.340400 | 1.837700 |
| N | -4.540100 | -1.429700 | 2.475600 |
| C | -3.956500 | -2.610200 | 2.803300 |
| C | -2.554100 | -2.703900 | 2.384300 |
| C | -2.006500 | -1.639700 | 1.756100 |
| C | -1.795300 | -3.995400 | 2.665600 |
| O | -4.597900 | -3.475800 | 3.392200 |
| O | -4.758100 | 0.629700 | 1.639400 |
| C | -3.861000 | 3.398800 | 0.485500 |
| O | -4.355300 | 3.547800 | 1.805300 |
| O | -0.621300 | 2.371900 | -1.297500 |
| C | -1.407400 | 4.046600 | 0.936200 |
| O | -1.748500 | -0.137900 | -1.513900 |
| Cl | -1.228100 | 5.349900 | -0.276500 |
| H | -3.510900 | 0.718100 | -0.825800 |
| H | -0.988300 | 0.418600 | 0.827300 |
| H | -2.590900 | 2.714400 | -1.701300 |
| H | -5.504600 | -1.352300 | 2.748000 |
| H | -0.984200 | -1.653900 | 1.404100 |
| H | -0.770400 | -3.966800 | 2.295400 |
| H | -1.760500 | -4.189600 | 3.739100 |
| H | -2.297900 | -4.842100 | 2.194200 |
| H | -3.976800 | 4.344600 | -0.045900 |
| H | -4.506600 | 2.684700 | -0.026900 |
| H | -4.488100 | 2.651000 | 2.150600 |
| H | -0.390300 | 3.307700 | -1.368100 |
| H | -1.768100 | 4.493700 | 1.863900 |
| H | -0.415800 | 3.642500 | 1.140800 |
| H | -1.013800 | 0.338300 | -1.925700 |

LNA-OpenmonoCl-Diol_21
Eopt -1448.948788

| | | | |
|---|---|---|---|
| C | -2.485600 | 0.788700 | -0.704400 |
| C | -1.959600 | 0.651900 | 0.738600 |
| O | -2.308300 | 1.847600 | 1.378500 |
| C | -2.303400 | 2.946700 | 0.469800 |
| C | -2.951700 | 2.255900 | -0.751000 |
| N | -2.371900 | -0.532900 | 1.490200 |
| C | -1.987700 | -1.756000 | 0.966600 |
| N | -2.547500 | -2.833600 | 1.586000 |
| C | -3.466300 | -2.886400 | 2.583000 |
| C | -3.888600 | -1.564700 | 3.058400 |
| C | -3.328300 | -0.475100 | 2.486900 |
| C | -4.929600 | -1.489100 | 4.168700 |
| O | -3.868300 | -3.970700 | 2.994100 |
| O | -1.236700 | -1.926300 | 0.003500 |
| C | -3.215600 | 4.067900 | 1.051500 |
| O | -2.835900 | 4.506500 | 2.348200 |
| O | -2.650000 | 2.846400 | -2.006500 |
| C | -0.839400 | 3.417500 | 0.211100 |
| O | -1.432400 | 0.465200 | -1.610200 |
| Cl | 0.052900 | 3.815300 | 1.709700 |
| H | -3.341100 | 0.124000 | -0.850800 |
| H | -0.865200 | 0.593400 | 0.780300 |
| H | -4.035500 | 2.252100 | -0.614100 |
| H | -2.264300 | -3.732600 | 1.235400 |
| H | -3.589600 | 0.525700 | 2.804100 |
| H | -5.165100 | -0.461900 | 4.449100 |
| H | -5.856800 | -1.973500 | 3.857300 |
| H | -4.572100 | -2.005300 | 5.061700 |
| H | -3.221000 | 4.923900 | 0.374700 |
| H | -4.249800 | 3.727900 | 1.121300 |
| H | -1.873700 | 4.454000 | 2.423100 |
| H | -2.046900 | 2.220800 | -2.438000 |

| | | | |
|---|---|---|---|
| H | -0.260700 | 2.655200 | -0.309800 |
| H | -0.829600 | 4.302300 | -0.426000 |
| H | -1.197900 | -0.468900 | -1.475500 |

LNA-OpenmonoCl-Diol_22
Eopt -1448.955334

| | | | |
|---|---|---|---|
| C | -2.486400 | 0.808200 | -0.691500 |
| C | -1.954200 | 0.638200 | 0.742900 |
| O | -2.311600 | 1.841900 | 1.373400 |
| C | -2.304800 | 2.946700 | 0.457700 |
| C | -2.096900 | 2.270800 | -0.928500 |
| N | -2.305700 | -0.498300 | 1.602300 |
| C | -2.888600 | -1.647900 | 1.088400 |
| N | -3.012200 | -2.665100 | 1.988200 |
| C | -2.613100 | -2.745000 | 3.281900 |
| C | -1.953700 | -1.528100 | 3.762300 |
| C | -1.836200 | -0.491900 | 2.903500 |
| C | -1.439700 | -1.494800 | 5.196600 |
| O | -2.812000 | -3.768200 | 3.929800 |
| O | -3.283000 | -1.859700 | -0.057400 |
| C | -3.685300 | 3.650700 | 0.611100 |
| O | -4.789400 | 2.781800 | 0.396500 |
| O | -0.778200 | 2.328200 | -1.467400 |
| C | -1.145800 | 3.858900 | 0.934600 |
| O | -1.905100 | -0.135600 | -1.586900 |
| Cl | -1.066100 | 5.396000 | 0.024400 |
| H | -3.574600 | 0.711200 | -0.669500 |
| H | -0.861700 | 0.564500 | 0.711100 |
| H | -2.777200 | 2.706100 | -1.663900 |
| H | -3.450600 | -3.499200 | 1.636800 |
| H | -1.367100 | 0.439500 | 3.195000 |
| H | -0.970500 | -0.544100 | 5.450500 |
| H | -2.258800 | -1.662500 | 5.898500 |
| H | -0.704200 | -2.285500 | 5.356100 |
| H | -3.776900 | 4.088400 | 1.606500 |
| H | -3.761800 | 4.478600 | -0.096000 |
| H | -4.823600 | 2.153000 | 1.124800 |
| H | -0.701600 | 1.541400 | -2.026700 |
| H | -1.259900 | 4.113000 | 1.987900 |
| H | -0.181300 | 3.362300 | 0.828700 |
| H | -2.349300 | -0.984900 | -1.415200 |

35
LNA-OpenmonoCl-Diol_23
Eopt -1448.950502

| | | | |
|---|---|---|---|
| C | -2.460200 | 0.792700 | -0.709100 |
| C | -2.004200 | 0.640200 | 0.741000 |
| O | -2.260400 | 1.854200 | 1.384300 |
| C | -2.332200 | 2.947800 | 0.466300 |
| C | -2.194300 | 2.287700 | -0.941100 |
| N | -2.508000 | -0.496100 | 1.506800 |
| C | -3.864300 | -0.546400 | 1.780700 |
| N | -4.251100 | -1.657000 | 2.470100 |
| C | -3.521600 | -2.714100 | 2.908200 |
| C | -2.097400 | -2.620600 | 2.570100 |
| C | -1.674900 | -1.528900 | 1.894100 |
| C | -1.173900 | -3.757900 | 2.990400 |
| O | -4.064200 | -3.629500 | 3.520800 |
| O | -4.717500 | 0.286200 | 1.469600 |
| C | -3.746800 | 3.565200 | 0.662600 |
| O | -3.967900 | 4.011600 | 1.991100 |
| O | -0.990200 | 2.544700 | -1.659600 |
| C | -1.197100 | 3.934000 | 0.865100 |
| O | -1.752000 | -0.144800 | -1.509900 |
| Cl | 0.421600 | 3.164600 | 0.950500 |
| H | -3.535800 | 0.613000 | -0.766700 |
| H | -0.916900 | 0.537900 | 0.764300 |
| H | -2.988200 | 2.661700 | -1.591200 |
| H | -5.231300 | -1.707300 | 2.686900 |
| H | -0.639800 | -1.407300 | 1.606700 |
| H | -0.141600 | -3.593800 | 2.680600 |
| H | -1.184600 | -3.874900 | 4.075600 |
| H | -1.508900 | -4.701000 | 2.554400 |
| H | -3.885900 | 4.409600 | -0.014400 |
| H | -4.519600 | 2.834700 | 0.414600 |
| H | -3.967600 | 3.230400 | 2.558600 |
| H | -0.243600 | 2.291200 | -1.101800 |
| H | -1.137000 | 4.762600 | 0.159600 |
| H | -1.389700 | 4.363100 | 1.848900 |
| H | -1.902800 | 0.065900 | -2.438000 |

LNA-OpenmonoCl-Diol_24
Eopt -1448.951286

| | | | |
|---|---|---|---|
| C | -2.449000 | 0.797900 | -0.712600 |
| C | -2.024100 | 0.633800 | 0.745300 |
| O | -2.238100 | 1.858700 | 1.385700 |
| C | -2.345800 | 2.944700 | 0.464100 |
| C | -2.131300 | 2.282700 | -0.936200 |
| N | -2.581100 | -0.482600 | 1.503600 |
| C | -3.940100 | -0.477800 | 1.770500 |
| N | -4.375500 | -1.573300 | 2.455300 |
| C | -3.692600 | -2.661100 | 2.893100 |
| C | -2.264900 | -2.627400 | 2.558600 |
| C | -1.794500 | -1.552100 | 1.888300 |
| C | -1.392300 | -3.805700 | 2.975100 |
| O | -4.274500 | -3.554100 | 3.502900 |
| O | -4.759700 | 0.389200 | 1.458000 |
| C | -3.766000 | 3.560700 | 0.657000 |
| O | -4.815600 | 2.767500 | 0.122900 |
| O | -0.843700 | 2.459300 | -1.547700 |
| C | -1.221300 | 3.937500 | 0.864100 |
| O | -1.735100 | -0.124900 | -1.519400 |
| Cl | -1.419800 | 4.610000 | 2.509700 |
| H | -3.524400 | 0.633300 | -0.802000 |
| H | -0.941400 | 0.495500 | 0.786100 |
| H | -2.871700 | 2.673600 | -1.638700 |
| H | -5.357700 | -1.583800 | 2.668900 |
| H | -0.755000 | -1.474700 | 1.602400 |
| H | -0.353700 | -3.685800 | 2.666600 |
| H | -1.409600 | -3.926400 | 4.059800 |
| H | -1.768200 | -4.731200 | 2.534900 |
| H | -3.966200 | 3.735700 | 1.715700 |
| H | -3.812900 | 4.535700 | 0.170400 |
| H | -4.900200 | 1.961000 | 0.670300 |
| H | -0.804000 | 3.324300 | -1.969300 |
| H | -0.249600 | 3.443300 | 0.843100 |
| H | -1.178000 | 4.780300 | 0.175800 |
| H | -1.094400 | 0.400700 | -2.017100 |

LNA-OpenmonoCl-Diol_25
Eopt -1448.949686

| | | | |
|---|---|---|---|
| C | -2.503800 | 0.798400 | -0.679400 |
| C | -1.922700 | 0.654400 | 0.733300 |
| O | -2.349100 | 1.832500 | 1.363000 |
| C | -2.281200 | 2.949700 | 0.465500 |
| C | -2.175700 | 2.274800 | -0.936300 |
| N | -2.182000 | -0.494100 | 1.605200 |
| C | -2.764000 | -1.657900 | 1.125200 |
| N | -2.804200 | -2.677600 | 2.030000 |
| C | -2.323100 | -2.746000 | 3.296500 |
| C | -1.664800 | -1.512700 | 3.735500 |
| C | -1.629400 | -0.473900 | 2.872500 |
| C | -1.057300 | -1.466900 | 5.132300 |
| O | -2.457100 | -3.773600 | 3.954200 |
| O | -3.221000 | -1.876200 | 0.003500 |
| C | -3.643700 | 3.682500 | 0.624800 |
| O | -3.865100 | 4.145800 | 1.948600 |
| O | -0.935500 | 2.404700 | -1.617100 |
| C | -1.083100 | 3.856400 | 0.886000 |
| O | -1.923900 | -0.128300 | -1.591100 |
| Cl | 0.437700 | 2.970200 | 1.223900 |
| H | -3.587300 | 0.668000 | -0.611600 |
| H | -0.829600 | 0.634100 | 0.666700 |
| H | -2.934300 | 2.676000 | -1.611500 |
| H | -3.241200 | -3.523500 | 1.706800 |
| H | -1.163500 | 0.470800 | 3.129200 |
| H | -0.590600 | -0.506200 | 5.351900 |
| H | -1.823900 | -1.648500 | 5.887600 |
| H | -0.296400 | -2.241800 | 5.242500 |
| H | -3.694600 | 4.535000 | -0.054800 |
| H | -4.466500 | 3.018200 | 0.353800 |
| H | -3.797000 | 3.389400 | 2.543000 |
| H | -0.790100 | 1.547400 | -2.045300 |
| H | -0.868800 | 4.590000 | 0.109100 |
| H | -1.314300 | 4.413100 | 1.793500 |
| H | -2.317500 | -0.995900 | -1.390100 |

LNA-OpenmonoCl-Diol_26
Eopt -1448.949928

| | | | |
|---|---|---|---|
| C | -2.441100 | 0.801400 | -0.725500 |
| C | -2.040000 | 0.626000 | 0.754300 |
| O | -2.217800 | 1.866000 | 1.391600 |
| C | -2.358000 | 2.941600 | 0.462000 |
| C | -3.036800 | 2.216400 | -0.726200 |
| N | -2.687000 | -0.437400 | 1.523000 |
| C | -4.063100 | -0.401500 | 1.673800 |
| N | -4.573000 | -1.438800 | 2.396100 |
| C | -3.947300 | -2.497100 | 2.969900 |
| C | -2.495200 | -2.496700 | 2.765300 |
| C | -1.952500 | -1.479000 | 2.060300 |
| C | -1.677800 | -3.640500 | 3.354300 |
| O | -4.593200 | -3.340300 | 3.585300 |
| O | -4.837900 | 0.443200 | 1.227600 |
| C | -3.292900 | 3.984600 | 1.133700 |
| O | -2.758400 | 4.476400 | 2.355200 |
| O | -2.953300 | 2.860500 | -1.996700 |
| C | -0.921700 | 3.477800 | 0.194000 |
| O | -1.287700 | 0.663700 | -1.546600 |
| Cl | -0.877000 | 4.801900 | -1.008100 |
| H | -3.202200 | 0.072000 | -1.013100 |
| H | -0.970200 | 0.417600 | 0.822500 |
| H | -4.098100 | 2.107800 | -0.493200 |
| H | -5.570500 | -1.424300 | 2.521800 |
| H | -0.888000 | -1.429000 | 1.877800 |
| H | -0.612500 | -3.547200 | 3.142400 |
| H | -1.802200 | -3.677800 | 4.438200 |
| H | -2.017100 | -4.597100 | 2.952500 |
| H | -3.467800 | 4.824000 | 0.458700 |
| H | -4.269700 | 3.542800 | 1.340600 |
| H | -2.697800 | 3.734700 | 2.966900 |
| H | -2.187600 | 3.451700 | -1.992800 |
| H | -0.482600 | 3.863600 | 1.114400 |
| H | -0.258800 | 2.690900 | -0.163900 |
| H | -1.559500 | 0.852300 | -2.453500 |

LNA-OpenmonoCl-Diol_27
Eopt -1448.950323

| | | | |
|---|---|---|---|
| C | -2.501900 | 0.798100 | -0.684100 |
| C | -1.927100 | 0.654700 | 0.733700 |

| | | | |
|---|---|---|---|
| O | -2.344100 | 1.835700 | 1.368500 |
| C | -2.283900 | 2.946400 | 0.464400 |
| C | -2.190500 | 2.278400 | -0.940700 |
| N | -2.190400 | -0.494900 | 1.603100 |
| C | -2.761200 | -1.661800 | 1.117000 |
| N | -2.804900 | -2.682300 | 2.020700 |
| C | -2.336200 | -2.748900 | 3.291900 |
| C | -1.688300 | -1.512600 | 3.737900 |
| C | -1.650100 | -0.473200 | 2.875900 |
| C | -1.094200 | -1.464800 | 5.140300 |
| O | -2.471700 | -3.777200 | 3.948400 |
| O | -3.206800 | -1.883400 | -0.008400 |
| C | -3.615900 | 3.724700 | 0.664300 |
| O | -4.769600 | 2.935600 | 0.409300 |
| O | -0.956400 | 2.420500 | -1.632100 |
| C | -1.076800 | 3.840000 | 0.883000 |
| O | -1.906000 | -0.124400 | -1.591300 |
| Cl | 0.440700 | 2.947100 | 1.215500 |
| H | -3.584500 | 0.657600 | -0.626500 |
| H | -0.834400 | 0.628300 | 0.667800 |
| H | -2.965700 | 2.675900 | -1.599700 |
| H | -3.234200 | -3.530000 | 1.691900 |
| H | -1.191300 | 0.473400 | 3.137600 |
| H | -0.634800 | -0.501800 | 5.364900 |
| H | -1.867000 | -1.651200 | 5.888200 |
| H | -0.330400 | -2.235800 | 5.256900 |
| H | -3.682400 | 4.108800 | 1.683800 |
| H | -3.640600 | 4.592800 | 0.003200 |
| H | -4.817500 | 2.247900 | 1.082700 |
| H | -0.820000 | 1.568100 | -2.072800 |
| H | -0.857700 | 4.572300 | 0.105400 |
| H | -1.302000 | 4.392500 | 1.794400 |
| H | -2.306200 | -0.991200 | -1.402000 |

LNA-OpenmonoCl-Diol_28
Eopt -1448.949559

| | | | |
|---|---|---|---|
| C | -2.427600 | 0.781900 | -0.736200 |
| C | -2.065900 | 0.624900 | 0.755800 |
| O | -2.191700 | 1.871500 | 1.383900 |
| C | -2.371800 | 2.956700 | 0.478900 |
| C | -2.784700 | 2.275800 | -0.854100 |
| N | -2.784300 | -0.388700 | 1.524900 |
| C | -4.162300 | -0.286600 | 1.617800 |
| N | -4.745000 | -1.271800 | 2.358000 |
| C | -4.188800 | -2.333500 | 2.994300 |
| C | -2.731100 | -2.403900 | 2.847600 |
| C | -2.117000 | -1.438400 | 2.127800 |
| C | -1.988400 | -3.557600 | 3.511000 |
| O | -4.895500 | -3.123600 | 3.613400 |
| O | -4.876800 | 0.574100 | 1.102600 |
| C | -3.532800 | 3.808300 | 1.067900 |
| O | -3.220000 | 4.352000 | 2.342900 |
| O | -2.236100 | 2.874300 | -2.026000 |
| C | -1.039200 | 3.762800 | 0.444900 |
| O | -1.331700 | 0.343800 | -1.533800 |
| Cl | 0.363200 | 2.842300 | -0.179800 |
| H | -3.304900 | 0.177900 | -0.978500 |
| H | -1.007500 | 0.370700 | 0.850500 |
| H | -3.873400 | 2.319800 | -0.939200 |
| H | -5.744900 | -1.211000 | 2.443200 |
| H | -1.045000 | -1.436500 | 1.984700 |
| H | -0.912600 | -3.516400 | 3.339000 |
| H | -2.156100 | -3.547900 | 4.589600 |
| H | -2.352500 | -4.513800 | 3.130700 |
| H | -3.773900 | 4.628500 | 0.389600 |
| H | -4.439900 | 3.208700 | 1.163200 |
| H | -3.095200 | 3.613600 | 2.949900 |
| H | -1.278500 | 2.748500 | -1.974900 |
| H | -1.148000 | 4.654500 | -0.172600 |
| H | -0.773000 | 4.103300 | 1.445600 |
| H | -1.590800 | 0.400200 | -2.460400 |

LNA-OpenmonoCl-Diol_29
Eopt -1448.954475

| | | | |
|---|---|---|---|
| C | -2.386100 | 0.825500 | -0.730800 |
| C | -2.136700 | 0.599200 | 0.777600 |
| O | -2.111200 | 1.878300 | 1.375200 |
| C | -2.422900 | 2.932000 | 0.460500 |
| C | -1.982300 | 2.294300 | -0.878400 |
| N | -3.002400 | -0.347900 | 1.492100 |
| C | -3.066400 | -1.643600 | 1.005000 |
| N | -4.023500 | -2.420500 | 1.586600 |
| C | -4.942500 | -2.110700 | 2.534600 |
| C | -4.856300 | -0.722500 | 2.997200 |
| C | -3.905400 | 0.069700 | 2.454400 |
| C | -5.832400 | -0.250700 | 4.068400 |
| O | -5.741600 | -2.957800 | 2.921800 |
| O | -2.371900 | -2.125700 | 0.109600 |
| C | -3.957300 | 3.198400 | 0.573700 |
| O | -4.484800 | 3.937800 | -0.517200 |
| O | -0.593900 | 2.396100 | -1.184400 |
| C | -1.580000 | 4.148500 | 0.918500 |
| O | -1.619500 | -0.084300 | -1.517700 |
| Cl | -1.889100 | 5.609400 | -0.067300 |
| H | -3.456000 | 0.709700 | -0.922100 |
| H | -1.137200 | 0.183000 | 0.944400 |
| H | -2.530600 | 2.738700 | -1.711300 |
| H | -4.075800 | -3.367400 | 1.251500 |
| H | -3.784500 | 1.092700 | 2.778000 |
| H | -5.672800 | 0.789900 | 4.351600 |
| H | -6.860900 | -0.348600 | 3.715700 |
| H | -5.735000 | -0.861100 | 4.968200 |
| H | -4.507700 | 2.258000 | 0.610200 |
| H | -4.186500 | 3.712700 | 1.508400 |
| H | -4.051200 | 4.801200 | -0.552200 |
| H | -0.388800 | 1.586300 | -1.674300 |
| H | -1.795500 | 4.401300 | 1.956100 |
| H | -0.512700 | 3.931300 | 0.863800 |
| H | -1.847600 | -0.991600 | -1.244900 |

LNA-OpenmonoCl-Diol_3
Eopt -1448.953109

| | | | |
|---|---|---|---|
| C | -2.457900 | 0.793900 | -0.712000 |
| C | -2.009000 | 0.640900 | 0.741600 |
| O | -2.255100 | 1.856900 | 1.387800 |
| C | -2.335000 | 2.943200 | 0.465200 |
| C | -2.191500 | 2.289700 | -0.945400 |
| N | -2.519100 | -0.495000 | 1.503400 |
| C | -3.868700 | -0.518700 | 1.812300 |
| N | -4.263700 | -1.633200 | 2.490900 |
| C | -3.548200 | -2.715300 | 2.889200 |
| C | -2.132300 | -2.650300 | 2.511800 |
| C | -1.701900 | -1.555800 | 1.845500 |
| C | -1.226600 | -3.819400 | 2.880600 |
| O | -4.095500 | -3.628100 | 3.501400 |
| O | -4.709400 | 0.341600 | 1.540700 |
| C | -3.730800 | 3.591400 | 0.703000 |
| O | -4.819500 | 2.777100 | 0.289800 |
| O | -0.978900 | 2.544200 | -1.653100 |
| C | -1.198300 | 3.922000 | 0.871900 |
| O | -1.745400 | -0.143600 | -1.509400 |
| Cl | 0.415000 | 3.145300 | 0.985500 |
| H | -3.532900 | 0.611600 | -0.773600 |
| H | -0.922500 | 0.532500 | 0.770100 |
| H | -2.984100 | 2.669800 | -1.594000 |
| H | -5.238300 | -1.665500 | 2.734800 |
| H | -0.673400 | -1.454600 | 1.527400 |
| H | -0.200600 | -3.675800 | 2.541300 |
| H | -1.206700 | -3.957800 | 3.963200 |
| H | -1.599900 | -4.744500 | 2.437400 |
| H | -3.859100 | 3.838600 | 1.758500 |
| H | -3.797600 | 4.531900 | 0.153800 |
| H | -4.840200 | 1.969200 | 0.840200 |
| H | -0.243400 | 2.224200 | -1.116900 |
| H | -1.119900 | 4.742200 | 0.157800 |
| H | -1.400400 | 4.357400 | 1.850700 |
| H | -1.928800 | 0.044800 | -2.436600 |

LNA-OpenmonoCl-Diol_30
Eopt -1448.952696

| | | | |
|---|---|---|---|
| C | -2.488400 | 0.791700 | -0.704700 |
| C | -1.955000 | 0.654000 | 0.737600 |
| O | -2.313100 | 1.848200 | 1.383400 |
| C | -2.300500 | 2.941100 | 0.466200 |
| C | -2.999300 | 2.241100 | -0.718500 |
| N | -2.350700 | -0.539200 | 1.483900 |
| C | -1.957200 | -1.754500 | 0.948900 |
| N | -2.502800 | -2.842400 | 1.563000 |
| C | -3.415900 | -2.911600 | 2.564300 |
| C | -3.848000 | -1.597800 | 3.052500 |
| C | -3.301500 | -0.498400 | 2.486300 |
| C | -4.882800 | -1.541100 | 4.169700 |
| O | -3.806300 | -4.002500 | 2.969000 |
| O | -1.210200 | -1.909400 | -0.019900 |
| C | -3.168700 | 4.085000 | 1.052800 |
| O | -3.393900 | 5.115400 | 0.097100 |
| O | -2.809900 | 2.864500 | -1.979900 |
| C | -0.836200 | 3.392100 | 0.187300 |
| O | -1.429500 | 0.529100 | -1.621500 |
| Cl | 0.059900 | 3.754900 | 1.691300 |
| H | -3.321300 | 0.101900 | -0.864000 |
| H | -0.859700 | 0.605000 | 0.774300 |
| H | -4.070400 | 2.204700 | -0.503700 |
| H | -2.212600 | -3.735700 | 1.203600 |
| H | -3.569000 | 0.496900 | 2.812800 |
| H | -5.127100 | -0.519200 | 4.460900 |
| H | -5.806900 | -2.032500 | 3.859900 |
| H | -4.514900 | -2.061900 | 5.055800 |
| H | -4.133700 | 3.696000 | 1.381500 |
| H | -2.698300 | 4.512600 | 1.939600 |
| H | -3.589000 | 4.717800 | -0.762500 |
| H | -2.151700 | 2.316700 | -2.436000 |
| H | -0.268100 | 2.634300 | -0.349800 |
| H | -0.822600 | 4.291500 | -0.429200 |
| H | -1.167900 | -0.401300 | -1.519000 |

LNA-OpenmonoCl-Diol_31
Eopt -1448.944010

| | | | |
|---|---|---|---|
| C | -2.458900 | 0.809800 | -0.707000 |
| C | -2.005800 | 0.634500 | 0.747600 |
| O | -2.255900 | 1.852800 | 1.387500 |
| C | -2.336400 | 2.937900 | 0.454400 |
| C | -2.012800 | 2.258700 | -0.910600 |
| N | -2.503300 | -0.496900 | 1.526700 |
| C | -3.870800 | -0.627000 | 1.701400 |
| N | -4.236800 | -1.709300 | 2.444800 |
| C | -3.477200 | -2.678700 | 3.014100 |
| C | -2.039000 | -2.497300 | 2.792900 |

| | | | |
|---|---|---|---|
| C | -1.636900 | -1.429000 | 2.068800 |
| C | -1.076900 | -3.516400 | 3.391900 |
| O | -4.006300 | -3.596000 | 3.635000 |
| O | -4.755900 | 0.101600 | 1.254200 |
| C | -3.801200 | 3.458800 | 0.533400 |
| O | -4.073500 | 4.468200 | -0.431900 |
| O | -0.651000 | 2.298300 | -1.337300 |
| C | -1.294800 | 4.003200 | 0.885700 |
| O | -1.859500 | -0.172900 | -1.548800 |
| Cl | -1.568700 | 4.634300 | 2.538900 |
| H | -3.547000 | 0.765000 | -0.770700 |
| H | -0.917700 | 0.532500 | 0.756300 |
| H | -2.611400 | 2.710800 | -1.704200 |
| H | -5.226200 | -1.814900 | 2.588900 |
| H | -0.588800 | -1.240300 | 1.882100 |
| H | -0.033900 | -3.286700 | 3.173300 |
| H | -1.190600 | -3.553100 | 4.477100 |
| H | -1.289700 | -4.515100 | 3.006000 |
| H | -4.505200 | 2.638900 | 0.385200 |
| H | -4.012800 | 3.844700 | 1.531600 |
| H | -4.932700 | 4.858500 | -0.241600 |
| H | -0.531300 | 1.514900 | -1.891200 |
| H | -0.287500 | 3.588600 | 0.868900 |
| H | -1.310600 | 4.848800 | 0.198500 |
| H | -2.418800 | -0.957600 | -1.566600 |

LNA-OpenmonoCl-Diol_32
Eopt -1448.953106

| | | | |
|---|---|---|---|
| C | -2.469700 | 0.819200 | -0.696000 |
| C | -1.983100 | 0.622700 | 0.751100 |
| O | -2.277800 | 1.847300 | 1.376100 |
| C | -2.326400 | 2.945800 | 0.451200 |
| C | -2.013800 | 2.263900 | -0.909000 |
| N | -2.419000 | -0.491700 | 1.600500 |
| C | -3.037900 | -1.612900 | 1.066300 |
| N | -3.245000 | -2.620200 | 1.961900 |
| C | -2.896600 | -2.716500 | 3.269100 |
| C | -2.193300 | -1.533600 | 3.771500 |
| C | -1.993900 | -0.506400 | 2.916800 |
| C | -1.726300 | -1.524000 | 5.222100 |
| O | -3.170200 | -3.726900 | 3.909700 |
| O | -3.397700 | -1.806100 | -0.094400 |
| C | -3.784600 | 3.488500 | 0.527500 |
| O | -4.146400 | 3.881700 | 1.844200 |
| O | -0.651000 | 2.280600 | -1.328000 |
| C | -1.276300 | 3.984100 | 0.920100 |
| O | -1.901400 | -0.137300 | -1.583600 |
| Cl | -1.238100 | 5.426000 | -0.137700 |
| H | -3.561100 | 0.763000 | -0.702900 |
| H | -0.895900 | 0.488600 | 0.748400 |
| H | -2.605500 | 2.722000 | -1.704400 |
| H | -3.711400 | -3.432800 | 1.596600 |
| H | -1.485900 | 0.399600 | 3.223600 |
| H | -1.216000 | -0.598900 | 5.490900 |
| H | -2.576000 | -1.646200 | 5.896500 |
| H | -1.039400 | -2.352000 | 5.406900 |
| H | -3.908700 | 4.342500 | -0.140500 |
| H | -4.496700 | 2.732700 | 0.193900 |
| H | -4.003500 | 3.130900 | 2.430100 |
| H | -0.546900 | 1.508600 | -1.902300 |
| H | -1.491100 | 4.328100 | 1.931400 |
| H | -0.274600 | 3.555200 | 0.929100 |
| H | -2.367900 | -0.976600 | -1.422200 |

LNA-OpenmonoCl-Diol_33
Eopt -1448.949466

| | | | |
|---|---|---|---|
| C | -2.421500 | 0.807800 | -0.728200 |
| C | -2.074200 | 0.615000 | 0.761600 |
| O | -2.180100 | 1.871400 | 1.386500 |
| C | -2.381000 | 2.940800 | 0.462500 |
| C | -3.044800 | 2.206800 | -0.726200 |
| N | -2.835600 | -0.409800 | 1.470500 |
| C | -2.555600 | -1.724600 | 1.141100 |
| N | -3.413500 | -2.634100 | 1.684600 |
| C | -4.531000 | -2.441600 | 2.430400 |
| C | -4.829500 | -1.027300 | 2.679800 |
| C | -3.977200 | -0.102200 | 2.185000 |
| C | -6.082900 | -0.675100 | 3.471600 |
| O | -5.187100 | -3.403500 | 2.819400 |
| O | -1.647400 | -2.106000 | 0.401200 |
| C | -3.304700 | 3.991400 | 1.147300 |
| O | -4.569400 | 3.479700 | 1.563700 |
| O | -2.929700 | 2.823700 | -2.000000 |
| C | -0.952600 | 3.500900 | 0.210000 |
| O | -1.233800 | 0.682500 | -1.508200 |
| Cl | -0.933900 | 4.988300 | -0.782700 |
| H | -3.165200 | 0.074500 | -1.050600 |
| H | -1.030800 | 0.311700 | 0.905600 |
| H | -4.106200 | 2.085500 | -0.500100 |
| H | -3.210900 | -3.597400 | 1.479700 |
| H | -4.139700 | 0.957400 | 2.332100 |
| H | -6.215400 | 0.402100 | 3.577700 |
| H | -6.970900 | -1.075100 | 2.978900 |
| H | -6.035600 | -1.109000 | 4.471900 |
| H | -2.800300 | 4.416900 | 2.016300 |
| H | -3.483000 | 4.818700 | 0.458000 |
| H | -5.108100 | 4.205800 | 1.893500 |
| H | -2.061300 | 2.575200 | -2.342200 |
| H | -0.471800 | 3.745800 | 1.156900 |
| H | -0.313100 | 2.770500 | -0.283000 |
| H | -0.995200 | -0.253100 | -1.553900 |

LNA-OpenmonoCl-Diol_34
Eopt -1448.948922

| | | | |
|---|---|---|---|
| C | -2.436100 | 0.791100 | -0.730400 |
| C | -2.050300 | 0.629600 | 0.755000 |
| O | -2.208000 | 1.867600 | 1.386300 |
| C | -2.362500 | 2.946600 | 0.471600 |
| C | -2.926300 | 2.249800 | -0.796700 |
| N | -2.725800 | -0.414100 | 1.522500 |
| C | -4.088100 | -0.291300 | 1.736800 |
| N | -4.642300 | -1.336700 | 2.413800 |
| C | -4.071400 | -2.473100 | 2.887500 |
| C | -2.631100 | -2.557200 | 2.622500 |
| C | -2.045400 | -1.534600 | 1.960200 |
| C | -1.873900 | -3.789100 | 3.104100 |
| O | -4.752400 | -3.313200 | 3.468000 |
| O | -4.809500 | 0.639900 | 1.377800 |
| C | -3.344000 | 3.953300 | 1.142800 |
| O | -4.678300 | 3.475400 | 1.271000 |
| O | -2.577700 | 2.868600 | -2.034500 |
| C | -0.946400 | 3.551700 | 0.262500 |
| O | -1.293100 | 0.513900 | -1.539300 |
| Cl | -0.190500 | 4.118000 | 1.781500 |
| H | -3.252700 | 0.108400 | -0.978700 |
| H | -0.984100 | 0.411700 | 0.851000 |
| H | -4.015600 | 2.217900 | -0.732800 |
| H | -5.630100 | -1.262600 | 2.587000 |
| H | -0.987500 | -1.540000 | 1.735800 |
| H | -0.813300 | -3.753100 | 2.854900 |
| H | -1.959800 | -3.887400 | 4.188000 |
| H | -2.293500 | -4.693100 | 2.658900 |
| H | -2.982300 | 4.223300 | 2.136100 |
| H | -3.376900 | 4.878300 | 0.565500 |
| H | -4.661600 | 2.550000 | 1.569000 |
| H | -1.670500 | 2.614200 | -2.233000 |
| H | -0.268900 | 2.817500 | -0.172400 |
| H | -0.987700 | 4.403700 | -0.416100 |
| H | -1.595100 | 0.279200 | -2.425000 |

LNA-OpenmonoCl-Diol_35
Eopt -1448.944268

| | | | |
|---|---|---|---|
| C | -2.450100 | 0.801600 | -0.712700 |
| C | -2.022300 | 0.634700 | 0.745800 |
| O | -2.239500 | 1.858800 | 1.388400 |
| C | -2.345000 | 2.939800 | 0.460900 |
| C | -2.118800 | 2.283300 | -0.934800 |
| N | -2.566800 | -0.486800 | 1.506300 |
| C | -3.931100 | -0.516500 | 1.741000 |
| N | -4.351300 | -1.610200 | 2.437700 |
| C | -3.648900 | -2.668800 | 2.914300 |
| C | -2.214900 | -2.599600 | 2.614700 |
| C | -1.759200 | -1.524700 | 1.933500 |
| C | -1.318800 | -3.741700 | 3.079200 |
| O | -4.220600 | -3.565900 | 3.527600 |
| O | -4.769200 | 0.316100 | 1.391500 |
| C | -3.792500 | 3.479000 | 0.634400 |
| O | -4.028700 | 4.646600 | -0.141700 |
| O | -0.846100 | 2.485400 | -1.548500 |
| C | -1.285700 | 3.989100 | 0.895900 |
| O | -1.768100 | -0.161600 | -1.504800 |
| Cl | 0.361100 | 3.305100 | 1.097400 |
| H | -3.530600 | 0.667200 | -0.792100 |
| H | -0.938900 | 0.500000 | 0.778600 |
| H | -2.845900 | 2.691200 | -1.640400 |
| H | -5.337800 | -1.644300 | 2.626500 |
| H | -0.714700 | -1.421800 | 1.674500 |
| H | -0.276100 | -3.595500 | 2.796500 |
| H | -1.361900 | -3.841000 | 4.165300 |
| H | -1.653600 | -4.687100 | 2.648400 |
| H | -4.521500 | 2.714400 | 0.361500 |
| H | -3.979900 | 3.708500 | 1.685000 |
| H | -4.939600 | 4.932200 | -0.013900 |
| H | -0.163000 | 2.351500 | -0.879500 |
| H | -1.228700 | 4.798200 | 0.166800 |
| H | -1.561000 | 4.433700 | 1.851900 |
| H | -1.785600 | 0.135200 | -2.421500 |

LNA-OpenmonoCl-Diol_4
Eopt -1448.956241

| | | | |
|---|---|---|---|
| C | -2.386100 | 0.810900 | -0.734900 |
| C | -2.138000 | 0.604700 | 0.774400 |
| O | -2.111700 | 1.878300 | 1.372800 |
| C | -2.421100 | 2.941000 | 0.470200 |
| C | -2.107400 | 2.309100 | -0.908100 |
| N | -3.009800 | -0.337600 | 1.483000 |
| C | -3.021300 | -1.646100 | 1.028600 |
| N | -3.982700 | -2.431300 | 1.592200 |
| C | -4.955400 | -2.116000 | 2.483600 |
| C | -4.932700 | -0.710100 | 2.897800 |
| C | -3.975500 | 0.088900 | 2.376600 |
| C | -5.982700 | -0.226000 | 3.890800 |
| O | -5.749400 | -2.972700 | 2.861500 |
| O | -2.278400 | -2.130200 | 0.172300 |
| C | -3.916300 | 3.360600 | 0.653800 |
| O | -4.872900 | 2.436000 | 0.146000 |
| O | -0.788300 | 2.512800 | -1.405000 |
| C | -1.456600 | 4.096100 | 0.850500 |

| | | | |
|---|---|---|---|
| O | -1.542300 | -0.055500 | -1.494700 |
| Cl | -1.780600 | 4.784900 | 2.471400 |
| H | -3.439100 | 0.601200 | -0.936700 |
| H | -1.139400 | 0.192900 | 0.954700 |
| H | -2.793700 | 2.707800 | -1.657800 |
| H | -3.994800 | -3.388600 | 1.285100 |
| H | -3.903800 | 1.130900 | 2.650100 |
| H | -5.868600 | 0.830600 | 4.134100 |
| H | -6.985800 | -0.373400 | 3.486900 |
| H | -5.915300 | -0.792800 | 4.821200 |
| H | -4.127900 | 3.530200 | 1.710700 |
| H | -4.083900 | 4.316700 | 0.155200 |
| H | -5.760200 | 2.757300 | 0.338800 |
| H | -0.540200 | 1.649700 | -1.773100 |
| H | -0.423700 | 3.746100 | 0.854500 |
| H | -1.514800 | 4.907300 | 0.125400 |
| H | -1.732400 | -0.971100 | -1.222600 |

LNA-OpenmonoCl-Diol_5
Eopt -1448.956161

| | | | |
|---|---|---|---|
| C | -2.503900 | 0.797600 | -0.680900 |
| C | -1.923100 | 0.655000 | 0.735100 |
| O | -2.348500 | 1.832500 | 1.361000 |
| C | -2.281500 | 2.949900 | 0.467200 |
| C | -2.196500 | 2.279400 | -0.937200 |
| N | -2.193300 | -0.492200 | 1.608400 |
| C | -2.703000 | -1.682300 | 1.109700 |
| N | -2.742300 | -2.699700 | 2.016900 |
| C | -2.319200 | -2.743400 | 3.304700 |
| C | -1.742200 | -1.479600 | 3.769800 |
| C | -1.709200 | -0.443500 | 2.903500 |
| C | -1.216800 | -1.399000 | 5.197900 |
| O | -2.434100 | -3.775500 | 3.959000 |
| O | -3.097800 | -1.927400 | -0.029700 |
| C | -3.587600 | 3.769600 | 0.672100 |
| O | -4.759700 | 3.017100 | 0.387800 |
| O | -0.949600 | 2.406800 | -1.615800 |
| C | -1.013500 | 3.756800 | 0.860300 |
| O | -1.906400 | -0.117400 | -1.595300 |
| Cl | -1.038800 | 4.323800 | 2.557500 |
| H | -3.585800 | 0.652300 | -0.617800 |
| H | -0.829100 | 0.643800 | 0.682600 |
| H | -2.969300 | 2.680000 | -1.597600 |
| H | -3.125900 | -3.564800 | 1.677100 |
| H | -1.307200 | 0.523100 | 3.184700 |
| H | -0.818700 | -0.413200 | 5.440200 |
| H | -2.013400 | -1.622200 | 5.910100 |
| H | -0.421600 | -2.130300 | 5.353800 |
| H | -3.656900 | 4.139100 | 1.696200 |
| H | -3.575900 | 4.649000 | 0.026200 |
| H | -4.873700 | 2.369300 | 1.091800 |
| H | -0.866000 | 1.587600 | -2.128500 |
| H | -0.115500 | 3.150600 | 0.743300 |
| H | -0.893700 | 4.628200 | 0.216600 |
| H | -2.287300 | -0.991600 | -1.394600 |

LNA-OpenmonoCl-Diol_6
Eopt -1448.952708

| | | | |
|---|---|---|---|
| C | -2.426900 | 0.802000 | -0.728800 |
| C | -2.065700 | 0.623300 | 0.757700 |
| O | -2.190600 | 1.870000 | 1.389000 |
| C | -2.373700 | 2.939600 | 0.464700 |
| C | -3.017900 | 2.218100 | -0.750200 |
| N | -2.774100 | -0.409400 | 1.510300 |
| C | -4.122700 | -0.229700 | 1.771200 |
| N | -4.707200 | -1.274500 | 2.423500 |
| C | -4.179600 | -2.454600 | 2.836000 |
| C | -2.755000 | -2.599400 | 2.519500 |
| C | -2.139300 | -1.580300 | 1.879600 |
| C | -2.047900 | -3.888200 | 2.921800 |
| O | -4.884000 | -3.280400 | 3.409500 |
| O | -4.814100 | 0.747800 | 1.480000 |
| C | -3.300300 | 3.978900 | 1.164300 |
| O | -4.664400 | 3.585300 | 1.246900 |
| O | -2.864100 | 2.870600 | -2.013600 |
| C | -0.963600 | 3.518600 | 0.159100 |
| O | -1.258000 | 0.663100 | -1.523600 |
| Cl | -0.152300 | 4.184900 | 1.607500 |
| H | -3.181700 | 0.073300 | -1.034200 |
| H | -1.007100 | 0.374000 | 0.861800 |
| H | -4.086300 | 2.087200 | -0.574900 |
| H | -5.684200 | -1.160400 | 2.631800 |
| H | -1.091900 | -1.631300 | 1.613900 |
| H | -0.996700 | -3.896400 | 2.632900 |
| H | -2.099300 | -4.027900 | 4.003300 |
| H | -2.530600 | -4.749500 | 2.456200 |
| H | -2.935900 | 4.186300 | 2.172100 |
| H | -3.264800 | 4.927500 | 0.627100 |
| H | -4.710300 | 2.684000 | 1.611500 |
| H | -3.494000 | 3.599400 | -2.058500 |
| H | -0.303500 | 2.755200 | -0.250800 |
| H | -1.021700 | 4.317600 | -0.579100 |
| H | -1.452100 | 1.086000 | -2.370600 |

LNA-OpenmonoCl-Diol_7
Eopt -1448.951493

| | | | |
|---|---|---|---|
| C | -2.451000 | 0.800600 | -0.723700 |
| C | -2.022800 | 0.632400 | 0.751300 |
| O | -2.236800 | 1.864600 | 1.392900 |
| C | -2.346400 | 2.937400 | 0.461900 |
| C | -3.039700 | 2.216800 | -0.714300 |
| N | -2.630600 | -0.446600 | 1.528200 |
| C | -4.008900 | -0.469300 | 1.663800 |
| N | -4.478700 | -1.510000 | 2.408100 |
| C | -3.811800 | -2.521600 | 3.018600 |
| C | -2.358400 | -2.456300 | 2.834800 |
| C | -1.854900 | -1.434200 | 2.107400 |
| C | -1.495400 | -3.539300 | 3.471800 |
| O | -4.426800 | -3.378800 | 3.646800 |
| O | -4.818900 | 0.325500 | 1.186900 |
| C | -3.264900 | 4.021200 | 1.086100 |
| O | -3.671700 | 4.988700 | 0.118900 |
| O | -2.941800 | 2.899000 | -1.964500 |
| C | -0.919900 | 3.468200 | 0.141300 |
| O | -1.321400 | 0.668200 | -1.574700 |
| Cl | -0.049200 | 4.036400 | 1.597500 |
| H | -3.219600 | 0.075500 | -1.000300 |
| H | -0.945800 | 0.458200 | 0.802100 |
| H | -4.098900 | 2.116600 | -0.465400 |
| H | -5.477000 | -1.539200 | 2.521100 |
| H | -0.791300 | -1.333100 | 1.941000 |
| H | -0.432700 | -3.396300 | 3.275200 |
| H | -1.637600 | -3.550800 | 4.554000 |
| H | -1.777700 | -4.523100 | 3.092500 |
| H | -4.156600 | 3.550400 | 1.505600 |
| H | -2.767900 | 4.514300 | 1.922700 |
| H | -4.166400 | 5.683400 | 0.568300 |
| H | -3.308700 | 3.783900 | -1.822600 |
| H | -0.300100 | 2.701100 | -0.319500 |
| H | -0.969200 | 4.301600 | -0.558700 |
| H | -1.565700 | 1.086600 | -2.411000 |

LNA-OpenmonoCl-Diol_8
Eopt -1448.955896

| | | | |
|---|---|---|---|
| C | -2.397000 | 0.812900 | -0.732600 |
| C | -2.117900 | 0.605300 | 0.770400 |
| O | -2.132700 | 1.877000 | 1.379800 |
| C | -2.409300 | 2.939800 | 0.464900 |
| C | -2.072900 | 2.301500 | -0.905100 |
| N | -2.943100 | -0.374600 | 1.484200 |
| C | -2.873100 | -1.690500 | 1.057400 |
| N | -3.781600 | -2.523000 | 1.640600 |
| C | -4.775000 | -2.249800 | 2.523100 |
| C | -4.851400 | -0.833700 | 2.893900 |
| C | -3.945100 | 0.012000 | 2.355600 |
| C | -5.946000 | -0.391100 | 3.857500 |
| O | -5.507700 | -3.147900 | 2.927700 |
| O | -2.102800 | -2.145500 | 0.210300 |
| C | -3.901900 | 3.372900 | 0.635100 |
| O | -4.866400 | 2.390400 | 0.272800 |
| O | -0.728500 | 2.450700 | -1.352500 |
| C | -1.451700 | 4.074700 | 0.908300 |
| O | -1.600200 | -0.075900 | -1.517800 |
| Cl | -1.662400 | 5.567900 | -0.054500 |
| H | -3.459900 | 0.633100 | -0.906900 |
| H | -1.099800 | 0.233000 | 0.927100 |
| H | -2.721500 | 2.723200 | -1.675200 |
| H | -3.729600 | -3.486600 | 1.358300 |
| H | -3.952600 | 1.065900 | 2.589000 |
| H | -5.915200 | 0.680000 | 4.057800 |
| H | -6.930400 | -0.629600 | 3.450900 |
| H | -5.847500 | -0.914300 | 4.810400 |
| H | -4.080600 | 3.672600 | 1.669000 |
| H | -4.096600 | 4.257000 | 0.026100 |
| H | -5.749700 | 2.766700 | 0.348700 |
| H | -0.525000 | 1.607900 | -1.786000 |
| H | -1.614100 | 4.333800 | 1.953900 |
| H | -0.409100 | 3.767900 | 0.819700 |
| H | -1.758000 | -0.984700 | -1.204500 |

LNA-OpenmonoCl-Diol_9
Eopt -1448.956170

| | | | |
|---|---|---|---|
| C | -2.481000 | 0.797800 | -0.696800 |
| C | -1.964600 | 0.637700 | 0.741900 |
| O | -2.301400 | 1.845700 | 1.371500 |
| C | -2.310000 | 2.953800 | 0.465800 |
| C | -2.193200 | 2.287100 | -0.934400 |
| N | -2.407600 | -0.528700 | 1.517900 |
| C | -2.025000 | -1.781600 | 1.069500 |
| N | -2.612300 | -2.823400 | 1.723600 |
| C | -3.549700 | -2.821200 | 2.703500 |
| C | -3.960000 | -1.475200 | 3.113200 |
| C | -3.376500 | -0.420300 | 2.501800 |
| C | -5.009800 | -1.336700 | 4.209400 |
| O | -3.974600 | -3.880200 | 3.155300 |
| O | -1.249700 | -2.021300 | 0.144400 |
| C | -3.660400 | 3.702400 | 0.665800 |
| O | -4.796300 | 2.904400 | 0.354900 |
| O | -0.940500 | 2.449700 | -1.594600 |
| C | -1.089500 | 3.834600 | 0.849000 |
| O | -1.829400 | -0.077900 | -1.617900 |
| Cl | -1.173600 | 4.470100 | 2.520200 |
| H | -3.558800 | 0.618000 | -0.675500 |
| H | -0.871200 | 0.560200 | 0.761300 |
| H | -2.960800 | 2.679500 | -1.605500 |
| H | -2.331400 | -3.740200 | 1.420300 |
| H | -3.627900 | 0.591700 | 2.782800 |
| H | -5.224900 | -0.296300 | 4.454200 |

| | | | |
|---|---|---|---|
| H | -5.944200 | -1.811500 | 3.904600 |
| H | -4.673500 | -1.831200 | 5.122900 |
| H | -3.759800 | 4.058300 | 1.692200 |
| H | -3.682100 | 4.589000 | 0.030000 |
| H | -4.962200 | 2.312600 | 1.094900 |
| H | -0.830500 | 1.637200 | -2.112800 |
| H | -0.163900 | 3.263900 | 0.772600 |
| H | -0.996300 | 4.684000 | 0.172400 |
| H | -1.684000 | -0.944600 | -1.194600 |

**Cartesian coordinates for compound 20**

LNA-AldeFree_1
Eopt -1063.347090

| | | | |
|---|---|---|---|
| C | -1.208400 | -0.750700 | 0.865900 |
| C | -0.476800 | 0.281300 | 0.002300 |
| O | -0.462000 | 1.482800 | 0.726000 |
| C | -1.479200 | 1.504300 | 1.739700 |
| C | -2.259100 | 0.170500 | 1.505500 |
| N | 0.839400 | -0.059000 | -0.531000 |
| C | 1.884500 | -0.222000 | 0.362200 |
| N | 3.065400 | -0.573700 | -0.221200 |
| C | 3.363000 | -0.805900 | -1.524600 |
| C | 2.215400 | -0.643200 | -2.423200 |
| C | 1.030000 | -0.284300 | -1.881700 |
| C | -0.770500 | 1.613900 | 3.129100 |
| O | -0.027700 | 0.458100 | 3.487700 |
| O | -3.404400 | 0.211000 | 0.649400 |
| C | -2.392100 | 2.736900 | 1.517100 |
| O | -1.684900 | -1.822300 | 0.054000 |
| O | -2.646400 | 3.557200 | 2.403100 |
| C | 2.417100 | -0.893300 | -3.913100 |
| O | 4.502700 | -1.123000 | -1.852700 |
| O | 1.845400 | -0.087500 | 1.586500 |
| H | -0.519000 | -1.131300 | 1.620500 |
| H | -1.087600 | 0.501300 | -0.876500 |
| H | -2.565400 | -0.248200 | 2.467300 |
| H | 3.840300 | -0.685600 | 0.409500 |
| H | 0.146600 | -0.158400 | -2.492100 |
| H | -0.104600 | 2.479400 | 3.148400 |
| H | -1.504400 | 1.772900 | 3.922500 |
| H | -2.792800 | 2.821900 | 0.507200 |
| H | -1.790100 | -2.608300 | 0.603800 |
| H | 1.501400 | -0.756100 | -4.488300 |
| H | 3.170400 | -0.212400 | -4.313700 |
| H | 2.771200 | -1.911700 | -4.083900 |
| H | 0.736700 | 0.377500 | 2.884600 |
| H | -3.387000 | -0.610900 | 0.139900 |

LNA-AldeFree_10 Eopt
-1063.352368

| | | | |
|---|---|---|---|
| C | -1.221700 | -0.737500 | 0.890900 |
| C | -0.438400 | 0.263300 | 0.010800 |
| O | -0.514400 | 1.495200 | 0.694900 |
| C | -1.451500 | 1.481700 | 1.778700 |
| C | -2.272900 | 0.198800 | 1.489100 |
| N | 0.936200 | 0.007300 | -0.440700 |
| C | 1.432100 | -1.286600 | -0.520200 |
| N | 2.670600 | -1.381300 | -1.082500 |
| C | 3.459700 | -0.424400 | -1.630600 |
| C | 2.862600 | 0.912700 | -1.591900 |
| C | 1.649400 | 1.047000 | -1.012500 |
| C | -0.614700 | 1.432800 | 3.096200 |
| O | -1.410200 | 1.221700 | 4.253800 |
| O | -3.369000 | 0.308300 | 0.578900 |
| C | -2.351100 | 2.741800 | 1.730800 |
| O | -1.736000 | -1.817500 | 0.117900 |
| O | -2.528800 | 3.467600 | 2.714600 |
| C | 3.635200 | 2.074000 | -2.205900 |
| O | 4.553800 | -0.719400 | -2.102300 |
| O | 0.889400 | -2.334400 | -0.171400 |
| H | -0.558700 | -1.102700 | 1.679300 |
| H | -0.961000 | 0.398100 | -0.941400 |
| H | -2.646100 | -0.232500 | 2.421400 |
| H | 3.054900 | -2.309600 | -1.124200 |
| H | 1.156600 | 2.008900 | -0.951900 |
| H | 0.127800 | 0.635500 | 3.047900 |
| H | -0.041400 | 2.354400 | 3.219100 |
| H | -2.813700 | 2.931600 | 0.762100 |
| H | -0.980500 | -2.398600 | -0.081500 |
| H | 3.100000 | 3.020400 | -2.125400 |
| H | 4.601000 | 2.192200 | -1.711000 |
| H | 3.828900 | 1.886700 | -3.263700 |
| H | -1.943500 | 2.015500 | 4.399400 |
| H | -3.444300 | -0.568200 | 0.172400 |

LNA-AldeFree_11 Eopt
-1063.350061

| | | | |
|---|---|---|---|
| C | -1.199200 | -0.742300 | 0.869400 |
| C | -0.482400 | 0.287100 | -0.008000 |
| O | -0.450100 | 1.473500 | 0.739500 |
| C | -1.491200 | 1.511300 | 1.727300 |
| C | -2.253500 | 0.167100 | 1.514700 |
| N | 0.855600 | -0.065000 | -0.471700 |
| C | 1.076400 | -0.289400 | -1.818500 |
| N | 2.371900 | -0.582700 | -2.127800 |
| C | 3.458600 | -0.702200 | -1.322600 |
| C | 3.166500 | -0.450600 | 0.092900 |
| C | 1.893800 | -0.149700 | 0.432800 |
| C | -0.816900 | 1.660100 | 3.130000 |
| O | -0.044300 | 0.531800 | 3.529000 |
| O | -3.415700 | 0.176200 | 0.684600 |
| C | -2.406900 | 2.731300 | 1.453000 |
| O | -1.697500 | -1.825600 | 0.084400 |
| O | -2.665400 | 3.580800 | 2.310900 |
| C | 4.308000 | -0.529300 | 1.099100 |
| O | 4.551500 | -0.999500 | -1.796100 |
| O | 0.238600 | -0.274300 | -2.717900 |
| H | -0.502400 | -1.105400 | 1.627200 |
| H | -1.095000 | 0.499300 | -0.892500 |
| H | -2.537600 | -0.255700 | 2.480900 |
| H | 2.552400 | -0.749600 | -3.102700 |
| H | 1.600200 | 0.071400 | 1.451100 |
| H | -0.176100 | 2.544800 | 3.142200 |
| H | -1.575300 | 1.821500 | 3.899900 |
| H | -2.802800 | 2.778200 | 0.438400 |
| H | -0.966900 | -2.379100 | -0.212100 |
| H | 3.981000 | -0.309700 | 2.115600 |
| H | 4.751700 | -1.526400 | 1.095000 |
| H | 5.093800 | 0.182200 | 0.838600 |
| H | 0.337500 | 0.709300 | 4.395400 |
| H | -3.405700 | -0.679700 | 0.229000 |

LNA-AldeFree_12 Eopt
-1063.343519

| | | | |
|---|---|---|---|
| C | -1.796500 | -0.449500 | 0.234800 |
| C | -0.516600 | 0.349400 | -0.098100 |
| O | -0.382000 | 1.365400 | 0.864700 |
| C | -1.581100 | 1.573900 | 1.615600 |
| C | -2.197400 | 0.150200 | 1.591200 |
| N | 0.741000 | -0.384600 | -0.232900 |
| C | 1.202200 | -1.113500 | 0.850700 |
| N | 2.380700 | -1.760600 | 0.626700 |
| C | 3.153100 | -1.813200 | -0.487400 |
| C | 2.611300 | -1.039800 | -1.609300 |
| C | 1.448100 | -0.377200 | -1.421800 |
| C | -1.182900 | 2.031700 | 3.047200 |
| O | -0.606300 | 3.334200 | 3.110500 |
| O | -3.590400 | 0.045500 | 1.881500 |
| C | -2.452300 | 2.660200 | 0.926400 |
| O | -2.755600 | -0.252800 | -0.796100 |
| O | -3.662800 | 2.516400 | 0.722400 |
| C | 3.384700 | -1.024400 | -2.922600 |
| O | 4.192600 | -2.466700 | -0.490800 |
| O | 0.665200 | -1.236200 | 1.951000 |
| H | -1.574000 | -1.513400 | 0.345700 |
| H | -0.649400 | 0.872000 | -1.047800 |
| H | -1.681300 | -0.439000 | 2.352200 |
| H | 2.733800 | -2.288500 | 1.405900 |
| H | 1.002500 | 0.209600 | -2.213100 |
| H | -2.053300 | 1.998000 | 3.706100 |
| H | -0.463900 | 1.321400 | 3.461300 |
| H | -1.904100 | 3.558900 | 0.643200 |
| H | -3.566500 | -0.702600 | -0.532100 |
| H | 2.895600 | -0.423100 | -3.689200 |
| H | 4.387100 | -0.620500 | -2.768500 |
| H | 3.495400 | -2.038400 | -3.311200 |
| H | -0.191200 | 3.456700 | 3.969900 |
| H | -4.028300 | 0.776800 | 1.415600 |

LNA-AldeFree_13 Eopt
-1063.350051

| | | | |
|---|---|---|---|
| C | -1.225500 | -0.739400 | 0.859300 |
| C | -0.457400 | 0.277600 | -0.011900 |
| O | -0.475500 | 1.477700 | 0.725900 |
| C | -1.480500 | 1.502600 | 1.747900 |
| C | -2.263800 | 0.181200 | 1.511600 |
| N | 0.896700 | 0.013300 | -0.519100 |
| C | 1.366400 | -1.284600 | -0.668200 |
| N | 2.590500 | -1.375500 | -1.261900 |
| C | 3.383400 | -0.410000 | -1.788700 |
| C | 2.811300 | 0.933900 | -1.679700 |
| C | 1.615000 | 1.063800 | -1.065400 |
| C | -0.761400 | 1.606500 | 3.132900 |
| O | 0.005000 | 0.459500 | 3.480000 |
| O | -3.418100 | 0.226000 | 0.670800 |
| C | -2.388900 | 2.740300 | 1.536000 |
| O | -1.761700 | -1.800000 | 0.070600 |
| O | -2.580400 | 3.590800 | 2.410000 |
| C | 3.590500 | 2.107400 | -2.261700 |
| O | 4.460500 | -0.703100 | -2.299700 |
| O | 0.814800 | -2.341200 | -0.361400 |
| H | -0.538600 | -1.129100 | 1.613600 |
| H | -1.010700 | 0.459200 | -0.937900 |
| H | -2.560600 | -0.250500 | 2.470100 |
| H | 2.955700 | -2.307800 | -1.351700 |
| H | 1.142800 | 2.030500 | -0.949000 |
| H | -0.105500 | 2.480000 | 3.144800 |
| H | -1.491400 | 1.761100 | 3.931100 |
| H | -2.844600 | 2.800300 | 0.547600 |
| H | -1.005200 | -2.365800 | -0.169300 |
| H | 3.075400 | 3.058400 | -2.124800 |
| H | 4.570300 | 2.185800 | -1.787000 |
| H | 3.753500 | 1.964100 | -3.331400 |
| H | 0.493300 | 0.638300 | 4.290100 |
| H | -3.433000 | -0.642000 | 0.237300 |

LNA-AldeFree_14 Eopt
-1063.349548

| | | | |
|---|---|---|---|
| C | -1.808600 | -0.431700 | 0.222800 |
| C | -0.508700 | 0.338400 | -0.084900 |
| O | -0.386400 | 1.390900 | 0.841600 |
| C | -1.565200 | 1.561200 | 1.632500 |
| C | -2.216800 | 0.148500 | 1.584200 |
| N | 0.730200 | -0.431200 | -0.177000 |
| C | 1.263800 | -0.965900 | 0.983000 |
| N | 2.380400 | -1.722800 | 0.786100 |
| C | 3.032100 | -2.038900 | -0.361400 |
| C | 2.412800 | -1.465200 | -1.560900 |
| C | 1.307500 | -0.703300 | -1.403300 |
| C | -1.115400 | 2.048600 | 3.038400 |
| O | -0.383600 | 1.077300 | 3.772100 |
| O | -3.618900 | 0.055800 | 1.840100 |
| C | -2.425700 | 2.676600 | 0.985500 |
| O | -2.749200 | -0.186300 | -0.814000 |
| O | -3.658400 | 2.627800 | 0.945500 |
| C | 3.040600 | -1.764100 | -2.917100 |
| O | 4.036100 | -2.744200 | -0.329900 |
| O | 0.838800 | -0.819900 | 2.128800 |
| H | -1.609700 | -1.502300 | 0.315100 |
| H | -0.591600 | 0.842800 | -1.050500 |
| H | -1.730400 | -0.475300 | 2.336600 |
| H | 2.789100 | -2.112000 | 1.618500 |
| H | 0.800900 | -0.262700 | -2.251500 |
| H | -0.495100 | 2.943200 | 2.951600 |
| H | -1.980000 | 2.325600 | 3.646200 |
| H | -1.853800 | 3.503900 | 0.565000 |
| H | -3.583500 | -0.592200 | -0.547900 |
| H | 2.497200 | -1.302000 | -3.741500 |
| H | 4.069600 | -1.400800 | -2.947900 |
| H | 3.067800 | -2.840800 | -3.095500 |
| H | 0.187400 | 0.567100 | 3.169900 |
| H | -4.031600 | 0.861400 | 1.489500 |

LNA-AldeFree_15 Eopt
-1063.349877

| | | | |
|---|---|---|---|
| C | -1.806100 | -0.440100 | 0.228700 |
| C | -0.519300 | 0.348200 | -0.100400 |
| O | -0.382200 | 1.362200 | 0.868800 |
| C | -1.585500 | 1.575900 | 1.615400 |
| C | -2.190200 | 0.152700 | 1.589700 |
| N | 0.732700 | -0.395500 | -0.240000 |
| C | 1.191800 | -1.130200 | 0.840200 |
| N | 2.361900 | -1.790300 | 0.609800 |
| C | 3.128700 | -1.849400 | -0.507800 |
| C | 2.589500 | -1.069300 | -1.626300 |
| C | 1.434300 | -0.394500 | -1.432400 |
| C | -1.202600 | 2.037700 | 3.050400 |
| O | -2.270800 | 1.855300 | 3.979500 |
| O | -3.579900 | 0.057700 | 1.901000 |
| C | -2.482100 | 2.613200 | 0.880900 |
| O | -2.785800 | -0.202900 | -0.771800 |
| O | -2.827800 | 3.683400 | 1.388100 |
| C | 3.356300 | -1.061300 | -2.943500 |
| O | 4.161800 | -2.512600 | -0.516500 |
| O | 0.660900 | -1.246200 | 1.943700 |
| H | -1.603400 | -1.509400 | 0.324600 |
| H | -0.647900 | 0.871900 | -1.049800 |
| H | -1.660900 | -0.430100 | 2.347200 |
| H | 2.712900 | -2.323000 | 1.386700 |
| H | 0.991600 | 0.196400 | -2.222200 |
| H | -0.344500 | 1.459900 | 3.400600 |
| H | -0.876000 | 3.080100 | 3.053300 |
| H | -2.781500 | 2.323400 | -0.125600 |
| H | -3.632000 | -0.472800 | -0.388900 |
| H | 2.869800 | -0.454700 | -3.707600 |
| H | 4.363800 | -0.668400 | -2.794500 |
| H | 3.454200 | -2.076300 | -3.333100 |
| H | -2.062700 | 2.334400 | 4.789000 |
| H | -3.697600 | 0.493200 | 2.759000 |

LNA-AldeFree_2 Eopt
-1063.352305

| | | | |
|---|---|---|---|
| C | -1.338100 | -0.732200 | 0.760200 |
| C | -0.390200 | 0.253700 | 0.040500 |
| O | -0.585700 | 1.510800 | 0.644700 |
| C | -1.400200 | 1.441900 | 1.820200 |
| C | -2.301000 | 0.232500 | 1.468100 |
| N | 1.026100 | -0.112200 | -0.072000 |
| C | 1.317700 | -1.298800 | -0.724600 |
| N | 2.621800 | -1.686200 | -0.637700 |
| C | 3.657100 | -1.121700 | 0.032700 |
| C | 3.288700 | 0.103800 | 0.747600 |
| C | 2.009400 | 0.530300 | 0.658600 |
| C | -0.503900 | 1.201100 | 3.082800 |
| O | 0.405300 | 2.260000 | 3.351700 |
| O | -3.422300 | 0.478200 | 0.621200 |
| C | -2.244100 | 2.733900 | 1.959900 |
| O | -1.947700 | -1.615900 | -0.178800 |
| O | -2.272900 | 3.394400 | 3.003400 |
| C | 4.359700 | 0.827400 | 1.554500 |
| O | 4.767400 | -1.644200 | 0.004800 |
| O | 0.517000 | -2.016200 | -1.326900 |
| H | -0.754000 | -1.291400 | 1.495000 |
| H | -0.687400 | 0.393500 | -1.004600 |
| H | -2.673000 | -0.240000 | 2.380100 |
| H | 2.855900 | -2.536500 | -1.120800 |
| H | 1.690000 | 1.436400 | 1.155000 |
| H | -1.128500 | 1.047600 | 3.965600 |
| H | 0.085000 | 0.290400 | 2.973200 |
| H | -2.801600 | 3.011700 | 1.065000 |
| H | -1.241300 | -2.077000 | -0.667100 |
| H | 3.966700 | 1.705500 | 2.068700 |
| H | 4.785200 | 0.163100 | 2.308700 |
| H | 5.172800 | 1.153800 | 0.903800 |
| H | -0.111100 | 3.052700 | 3.553300 |
| H | -3.470300 | -0.299300 | 0.042400 |

LNA-AldeFree_3 Eopt
-1063.350750

| | | | |
|---|---|---|---|
| C | -1.235200 | -0.746600 | 0.839600 |
| C | -0.471800 | 0.282700 | 0.003900 |
| O | -0.462200 | 1.489000 | 0.722400 |
| C | -1.472900 | 1.498100 | 1.736900 |
| C | -2.270200 | 0.169200 | 1.510300 |
| N | 0.849800 | -0.080700 | -0.498100 |
| C | 1.878700 | -0.225800 | 0.416700 |
| N | 3.064900 | -0.606800 | -0.136800 |
| C | 3.382400 | -0.875600 | -1.428500 |
| C | 2.248300 | -0.739700 | -2.348500 |
| C | 1.057700 | -0.355400 | -1.836800 |
| C | -0.759700 | 1.614800 | 3.119100 |
| O | -0.066100 | 0.441300 | 3.505400 |
| O | -3.460100 | 0.227000 | 0.707700 |
| C | -2.337400 | 2.769200 | 1.580800 |
| O | -1.750100 | -1.756600 | -0.012300 |
| O | -3.546100 | 2.773800 | 1.831400 |
| C | 2.467700 | -1.049900 | -3.824500 |
| O | 4.528500 | -1.196200 | -1.730700 |
| O | 1.824100 | -0.044600 | 1.634800 |
| H | -0.569400 | -1.181400 | 1.586800 |
| H | -1.057900 | 0.510400 | -0.889700 |
| H | -2.540200 | -0.260700 | 2.478300 |
| H | 3.828500 | -0.707400 | 0.509200 |
| H | 0.180000 | -0.256100 | -2.460200 |
| H | -0.051700 | 2.446800 | 3.119300 |
| H | -1.482500 | 1.821200 | 3.912400 |
| H | -1.797700 | 3.649700 | 1.231200 |
| H | -2.704900 | -1.600900 | -0.044200 |
| H | 1.558200 | -0.936700 | -4.414700 |
| H | 3.226600 | -0.387000 | -4.244000 |
| H | 2.821400 | -2.075200 | -3.948700 |
| H | 0.691600 | 0.306700 | 2.900800 |
| H | -4.015300 | 0.943800 | 1.051300 |

LNA-AldeFree_4 Eopt
-1063.350060

| | | | |
|---|---|---|---|
| C | -1.346300 | -0.734300 | 0.741000 |
| C | -0.407200 | 0.259900 | 0.022700 |
| O | -0.548400 | 1.506400 | 0.666600 |
| C | -1.423900 | 1.458000 | 1.798700 |
| C | -2.297600 | 0.214700 | 1.485500 |
| N | 0.995500 | -0.133100 | -0.151800 |
| C | 1.243400 | -1.280800 | -0.886800 |
| N | 2.543000 | -1.692200 | -0.871800 |
| C | 3.611400 | -1.184900 | -0.207700 |
| C | 3.288200 | -0.006400 | 0.601700 |
| C | 2.013700 | 0.442800 | 0.586300 |
| C | -0.584600 | 1.370700 | 3.118700 |
| O | 0.168200 | 0.172900 | 3.269500 |
| O | -3.462500 | 0.410800 | 0.683600 |
| C | -2.285400 | 2.746100 | 1.819300 |
| O | -1.970100 | -1.603500 | -0.206500 |
| O | -2.377400 | 3.475600 | 2.811300 |
| C | 4.399400 | 0.644800 | 1.416500 |
| O | 4.714100 | -1.714100 | -0.313900 |
| O | 0.413300 | -1.945300 | -1.509600 |
| H | -0.750400 | -1.310400 | 1.451900 |
| H | -0.741200 | 0.436100 | -1.005300 |
| H | -2.609400 | -0.261600 | 2.417700 |
| H | 2.743800 | -2.513100 | -1.416600 |
| H | 1.716700 | 1.306000 | 1.162800 |
| H | 0.099300 | 2.220200 | 3.180000 |
| H | -1.241900 | 1.446500 | 3.988300 |
| H | -2.802000 | 2.957300 | 0.882700 |
| H | -1.269500 | -2.040400 | -0.724000 |
| H | 4.048700 | 1.508100 | 1.982300 |
| H | 4.823700 | -0.071200 | 2.122600 |
| H | 5.205800 | 0.978400 | 0.760800 |
| H | 0.636900 | 0.197400 | 4.110700 |
| H | -3.490200 | -0.372300 | 0.110200 |

LNA-AldeFree_5 Eopt
-1063.347229

| | | | |
|---|---|---|---|
| C | -1.798300 | -0.447900 | 0.234900 |
| C | -0.516300 | 0.348000 | -0.096400 |
| O | -0.380300 | 1.370200 | 0.861800 |
| C | -1.579700 | 1.571300 | 1.616600 |
| C | -2.200700 | 0.149500 | 1.591400 |
| N | 0.736100 | -0.394600 | -0.230000 |
| C | 1.213000 | -1.088100 | 0.869500 |
| N | 2.374600 | -1.764100 | 0.642000 |
| C | 3.116900 | -1.873200 | -0.488400 |
| C | 2.556500 | -1.138500 | -1.627200 |

| | | | |
|---|---|---|---|
| C | 1.409400 | -0.449100 | -1.436800 |
| C | -1.163800 | 2.031700 | 3.039100 |
| O | -0.392300 | 3.226600 | 3.027100 |
| O | -3.594500 | 0.046700 | 1.877000 |
| C | -2.451000 | 2.666500 | 0.940300 |
| O | -2.753600 | -0.245800 | -0.798000 |
| O | -3.656800 | 2.517500 | 0.716900 |
| C | 3.291900 | -1.193600 | -2.961000 |
| O | 4.147400 | -2.540200 | -0.491200 |
| O | 0.701900 | -1.152000 | 1.986700 |
| H | -1.577600 | -1.512400 | 0.344500 |
| H | -0.643700 | 0.868900 | -1.048000 |
| H | -1.688800 | -0.444100 | 2.351900 |
| H | 2.739900 | -2.266800 | 1.432400 |
| H | 0.948900 | 0.107600 | -2.241500 |
| H | -2.045700 | 2.178800 | 3.666700 |
| H | -0.572300 | 1.253000 | 3.525400 |
| H | -1.909500 | 3.576900 | 0.683200 |
| H | -3.568000 | -0.691300 | -0.536300 |
| H | 2.789600 | -0.620500 | -3.740500 |
| H | 4.304100 | -0.799000 | -2.853800 |
| H | 3.377400 | -2.225700 | -3.306300 |
| H | 0.457900 | 3.024900 | 2.620100 |
| H | -4.032200 | 0.781800 | 1.417100 |

LNA-AldeFree_6 Eopt
-1063.349547

| | | | |
|---|---|---|---|
| C | -1.818600 | -0.417100 | 0.210500 |
| C | -0.508800 | 0.336900 | -0.086700 |
| O | -0.389000 | 1.387000 | 0.842700 |
| C | -1.568100 | 1.564600 | 1.636200 |
| C | -2.211300 | 0.150600 | 1.581300 |
| N | 0.724800 | -0.442800 | -0.167800 |
| C | 1.245200 | -0.983600 | 0.995900 |
| N | 2.358800 | -1.746900 | 0.806800 |
| C | 3.017500 | -2.066000 | -0.335800 |
| C | 2.413400 | -1.484800 | -1.539100 |
| C | 1.311900 | -0.715800 | -1.389400 |
| C | -1.115100 | 2.041100 | 3.050000 |
| O | -0.464100 | 1.027500 | 3.804400 |
| O | -3.613800 | 0.052300 | 1.844000 |
| C | -2.470900 | 2.627000 | 0.953600 |
| O | -2.769700 | -0.129000 | -0.804100 |
| O | -2.968800 | 3.573400 | 1.568000 |
| C | 3.052100 | -1.783600 | -2.890300 |
| O | 4.014700 | -2.780600 | -0.296600 |
| O | 0.812500 | -0.840700 | 2.139600 |
| H | -1.641600 | -1.493200 | 0.280400 |
| H | -0.578200 | 0.842500 | -1.052700 |
| H | -1.713000 | -0.484700 | 2.315400 |
| H | 2.758700 | -2.140300 | 1.641400 |
| H | 0.817500 | -0.268600 | -2.241300 |
| H | -0.444600 | 2.898200 | 2.958600 |
| H | -1.967100 | 2.380100 | 3.642900 |
| H | -2.630900 | 2.470400 | -0.113000 |
| H | -3.633500 | -0.376300 | -0.446300 |
| H | 2.520000 | -1.314600 | -3.718200 |
| H | 4.083800 | -1.427100 | -2.909900 |
| H | 3.073600 | -2.859700 | -3.072400 |
| H | 0.180600 | 0.567100 | 3.238600 |
| H | -3.773500 | 0.281500 | 2.767400 |

LNA-AldeFree_7 Eopt
-1063.352370

| | | | |
|---|---|---|---|
| C | -1.322300 | -0.735400 | 0.786300 |
| C | -0.395700 | 0.253000 | 0.038500 |
| O | -0.578100 | 1.512700 | 0.649200 |
| C | -1.404500 | 1.444100 | 1.816100 |
| C | -2.298900 | 0.229200 | 1.469700 |
| N | 1.019700 | -0.105600 | -0.117100 |
| C | 1.304300 | -1.308500 | -0.743100 |
| N | 2.614800 | -1.679500 | -0.687600 |
| C | 3.667800 | -1.076900 | -0.081300 |
| C | 3.312500 | 0.174200 | 0.594800 |
| C | 2.024900 | 0.581400 | 0.541400 |
| C | -0.483100 | 1.241500 | 3.062300 |
| O | -1.206500 | 0.947500 | 4.247800 |
| O | -3.406300 | 0.467700 | 0.600300 |
| C | -2.251800 | 2.733700 | 1.949600 |
| O | -1.924400 | -1.653500 | -0.124900 |
| O | -2.330200 | 3.366800 | 3.007800 |
| C | 4.410600 | 0.951800 | 1.310400 |
| O | 4.783400 | -1.585700 | -0.130400 |
| O | 0.497400 | -2.051800 | -1.302700 |
| H | -0.727700 | -1.262200 | 1.537000 |
| H | -0.725200 | 0.384500 | -0.997900 |
| H | -2.679700 | -0.235800 | 2.382500 |
| H | 2.840500 | -2.545000 | -1.147400 |
| H | 1.716700 | 1.508300 | 1.002200 |
| H | 0.218400 | 0.424000 | 2.894200 |
| H | 0.134700 | 2.126600 | 3.229500 |
| H | -2.770600 | 3.032000 | 1.038200 |
| H | -1.214500 | -2.119200 | -0.603500 |
| H | 4.043800 | 1.872500 | 1.764800 |
| H | 4.856600 | 0.342800 | 2.099000 |
| H | 5.206500 | 1.217800 | 0.612000 |
| H | -1.703900 | 1.738200 | 4.500400 |
| H | -3.483700 | -0.342800 | 0.073600 |

LNA-AldeFree_8 Eopt
-1063.349881

| | | | |
|---|---|---|---|
| C | -1.811900 | -0.431900 | 0.224500 |
| C | -0.518000 | 0.348400 | -0.100000 |
| O | -0.381900 | 1.364100 | 0.867700 |
| C | -1.584400 | 1.573800 | 1.614800 |
| C | -2.192900 | 0.152700 | 1.591000 |
| N | 0.732300 | -0.400400 | -0.232700 |
| C | 1.175600 | -1.149300 | 0.844300 |
| N | 2.348800 | -1.806800 | 0.622900 |
| C | 3.131700 | -1.852400 | -0.484000 |
| C | 2.609800 | -1.056900 | -1.599800 |
| C | 1.452200 | -0.384000 | -1.414300 |
| C | -1.201600 | 2.039000 | 3.047700 |
| O | -2.335800 | 2.117500 | 3.906600 |
| O | -3.583200 | 0.049500 | 1.900900 |
| C | -2.480300 | 2.614200 | 0.884300 |
| O | -2.788600 | -0.174600 | -0.774600 |
| O | -2.982800 | 3.580400 | 1.467400 |
| C | 3.396500 | -1.031500 | -2.905100 |
| O | 4.163900 | -2.517000 | -0.487000 |
| O | 0.627700 | -1.280000 | 1.937300 |
| H | -1.618600 | -1.503900 | 0.309400 |
| H | -0.639700 | 0.868600 | -1.052200 |
| H | -1.663100 | -0.434700 | 2.344300 |
| H | 2.688200 | -2.349400 | 1.398300 |
| H | 1.022900 | 0.218200 | -2.202900 |
| H | -0.484100 | 1.339700 | 3.482300 |
| H | -0.695000 | 3.006000 | 3.017900 |
| H | -2.631100 | 2.423400 | -0.178000 |
| H | -3.636300 | -0.455100 | -0.402900 |
| H | 2.922200 | -0.414300 | -3.668300 |
| H | 4.401800 | -0.641500 | -2.735300 |
| H | 3.499900 | -2.041300 | -3.306800 |
| H | -2.832200 | 2.910600 | 3.660000 |
| H | -3.702400 | 0.385000 | 2.800600 |

LNA-AldeFree_9 Eopt
-1063.344608

| | | | |
|---|---|---|---|
| C | -1.223300 | -0.746900 | 0.844400 |
| C | -0.482200 | 0.291400 | -0.005800 |
| O | -0.446300 | 1.475100 | 0.738000 |
| C | -1.489200 | 1.507600 | 1.722200 |
| C | -2.259500 | 0.164800 | 1.519100 |
| N | 0.833800 | -0.053700 | -0.534800 |
| C | 1.866000 | -0.254900 | 0.366300 |
| N | 3.047900 | -0.609500 | -0.213200 |
| C | 3.357000 | -0.811700 | -1.519000 |
| C | 2.223900 | -0.602100 | -2.426500 |
| C | 1.037600 | -0.240100 | -1.889000 |
| C | -0.792600 | 1.667900 | 3.104900 |
| O | -0.064000 | 0.514300 | 3.497400 |
| O | -3.444400 | 0.190500 | 0.723000 |
| C | -2.407800 | 2.737000 | 1.522000 |
| O | -1.718300 | -1.792900 | 0.010700 |
| O | -2.056800 | 3.726700 | 0.876800 |
| C | 2.441400 | -0.806700 | -3.921000 |
| O | 4.494400 | -1.142500 | -1.841800 |
| O | 1.813400 | -0.152100 | 1.594200 |
| H | -0.535300 | -1.148000 | 1.590300 |
| H | -1.087900 | 0.537900 | -0.881200 |
| H | -2.520300 | -0.259000 | 2.491900 |
| H | 3.813600 | -0.750600 | 0.422700 |
| H | 0.164400 | -0.075700 | -2.505000 |
| H | -0.112100 | 2.522800 | 3.091300 |
| H | -1.523800 | 1.865100 | 3.892000 |
| H | -3.386500 | 2.659600 | 1.995700 |
| H | -1.854100 | -2.583500 | 0.546600 |
| H | 1.536300 | -0.631600 | -4.502900 |
| H | 3.212900 | -0.127500 | -4.288700 |
| H | 2.777900 | -1.825600 | -4.121300 |
| H | 0.692000 | 0.399500 | 2.888900 |
| H | -3.355100 | -0.537500 | 0.091400 |

**Cartesian coordinates for compound 21**

LNA-OH-Free_1 Eopt
-1063.347693

| | | | |
|---|---|---|---|
| C | -0.872200 | -0.402900 | 0.299600 |
| C | 0.083100 | 0.776000 | 0.032200 |
| O | -0.013700 | 1.511400 | 1.229800 |
| C | -0.898300 | 0.801900 | 2.118800 |
| C | -0.566700 | -0.644800 | 1.765100 |
| N | 1.471600 | 0.470900 | -0.322700 |
| C | 1.675600 | -0.166800 | -1.532400 |
| N | 2.952500 | -0.597100 | -1.735000 |
| C | 4.032600 | -0.541000 | -0.915700 |
| C | 3.754200 | 0.100500 | 0.373100 |
| C | 2.503200 | 0.561000 | 0.596000 |
| C | -0.772500 | 1.246400 | 3.595300 |
| O | 0.422900 | 0.757200 | 4.185000 |
| O | -1.337600 | -1.672500 | 2.378900 |
| C | -2.322500 | 0.888000 | 1.540800 |
| O | -2.216200 | 0.101000 | 0.386900 |
| O | -2.594200 | 2.203300 | 1.276900 |
| C | 4.883900 | 0.212600 | 1.389900 |
| O | 5.109000 | -1.007300 | -1.277800 |

O 0.818600 -0.382200 -2.387400
H -0.734100 -1.242100 -0.385300
H -0.274200 1.392800 -0.799600
H 0.490800 -0.861200 1.938300
H 3.120200 -1.047600 -2.618000
H 2.243700 1.050700 1.526400
H -0.795900 2.335500 3.660700
H -1.617600 0.877900 4.178900
H -3.077400 0.453600 2.204100
H -1.791600 2.573300 0.893200
H 4.566900 0.698300 2.313400
H 5.267000 -0.776900 1.645800
H 5.712800 0.789400 0.975400
H 0.331500 -0.192800 4.315100
H -2.166100 -1.758300 1.893300

LNA-OH-Free_10 Eopt
-1063.341532
C -0.883700 -0.404000 0.306100
C 0.061600 0.775300 0.018500
O 0.024900 1.489000 1.231400
C -0.888900 0.805500 2.114800
C -0.561900 -0.642900 1.777400
N 1.424600 0.470200 -0.419100
C 1.560300 -0.104900 -1.669300
N 2.821200 -0.533100 -1.958900
C 3.939500 -0.536600 -1.190700
C 3.730400 0.029700 0.145700
C 2.497700 0.490800 0.453300
C -0.767100 1.266900 3.582100
O 0.593300 1.285500 3.982000
O -1.334300 -1.655400 2.425000
C -2.322800 0.868200 1.542500
O -2.218500 0.116300 0.370400
O -2.662600 2.178200 1.310900
C 4.906300 0.063400 1.114700
O 4.991400 -0.993000 -1.629200
O 0.661300 -0.275000 -2.490400
H -0.756500 -1.241300 -0.381400
H -0.340600 1.419900 -0.771000
H 0.497700 -0.849000 1.949300
H 2.939400 -0.933800 -2.873300
H 2.284100 0.923800 1.420000
H -1.184300 2.269000 3.695200
H -1.333600 0.606300 4.240500
H -3.036700 0.360500 2.201300
H -3.526400 2.184500 0.891000
H 4.638200 0.486700 2.083100
H 5.290200 -0.944200 1.284500
H 5.721600 0.660200 0.701600
H 1.025700 1.960000 3.449300
H -1.058900 -2.521800 2.112000

LNA-OH-Free_11 Eopt
-1063.341837
C -0.882700 -0.407900 0.304300
C 0.067700 0.776900 0.017300
O 0.005500 1.491000 1.232500
C -0.898700 0.799400 2.118600
C -0.553200 -0.643200 1.771300
N 1.472500 0.615600 -0.370400
C 2.038900 -0.633300 -0.569900
N 3.296000 -0.600200 -1.096100
C 4.066800 0.455200 -1.459900
C 3.424100 1.751800 -1.228600
C 2.178300 1.755200 -0.705700
C -0.768600 1.224600 3.599100
O -1.506200 0.356000 4.443000
O -1.290200 -1.668500 2.437800
C -2.324600 0.882300 1.539400
O -2.216600 0.112500 0.377600
O -2.599400 2.200700 1.283300
C 4.194200 3.019800 -1.577400
O 5.185200 0.272900 -1.931600
O 1.547500 -1.733800 -0.331500
H -0.789900 -1.243800 -0.388900
H -0.349500 1.355600 -0.811700
H 0.511700 -0.832800 1.934000
H 3.722300 -1.498400 -1.245600
H 1.650700 2.675800 -0.487200
H 0.280500 1.194100 3.896700
H -1.107100 2.252200 3.740500
H -3.069600 0.425800 2.201400
H -1.784800 2.584500 0.948000
H 3.633300 3.926700 -1.350700
H 5.133200 3.059800 -1.021800
H 4.441900 3.034400 -2.640400
H -1.451700 -0.534800 4.060800
H -1.111000 -2.510100 2.002400

LNA-OH-Free_12 Eopt
-1063.339893
C -0.883100 -0.405000 0.300500
C 0.036600 0.792900 0.015700
O 0.031300 1.477000 1.248600
C -0.895100 0.799000 2.123100
C -0.557800 -0.639800 1.773400
N 1.398000 0.608900 -0.491800
C 2.037200 1.734800 -0.983900
N 3.352800 1.548700 -1.284500
C 4.126800 0.440700 -1.171500
C 3.404200 -0.723500 -0.649800
C 2.096400 -0.575500 -0.341700
C -0.784900 1.251400 3.594800
O 0.482700 0.903100 4.124400
O -1.290300 -1.673800 2.421400
C -2.320700 0.880900 1.537700
O -2.219100 0.108000 0.376600
O -2.596900 2.200000 1.290000
C 4.162200 -2.036300 -0.487900
O 5.311100 0.473300 -1.492900
O 1.535300 2.842700 -1.164900
H -0.762300 -1.241000 -0.388700
H -0.450900 1.408000 -0.750900
H 0.503900 -0.825400 1.950900
H 3.827800 2.360100 -1.640800
H 1.524400 -1.408700 0.032100
H -0.942000 2.328700 3.673900
H -1.557400 0.773600 4.199400
H -3.069400 0.416500 2.188600
H -1.812900 2.571600 0.871100
H 3.536300 -2.840000 -0.099900
H 4.568900 -2.361400 -1.447500
H 5.003400 -1.906800 0.195900
H 1.144100 1.441400 3.675000
H -1.021800 -1.707100 3.346500

LNA-OH-Free_13 Eopt
-1063.340144
C -0.872300 -0.391300 0.296900
C 0.058300 0.809800 0.036200
O 0.051500 1.465200 1.284800
C -0.898400 0.793800 2.131800
C -0.564200 -0.645300 1.761900
N 1.411700 0.542300 -0.454300
C 1.515700 -0.048400 -1.700500
N 2.774000 -0.455000 -2.029100
C 3.921400 -0.413600 -1.306400
C 3.752100 0.184000 0.021800
C 2.520500 0.618900 0.369600
C -0.782500 1.200300 3.618500
O -1.282400 2.514400 3.834900
O -1.341000 -1.679700 2.355200
C -2.316800 0.902700 1.525900
O -2.224000 0.083300 0.394700
O -2.557800 2.221500 1.238700
C 4.969500 0.289300 0.932900
O 4.966700 -0.855700 -1.774600
O 0.593700 -0.248100 -2.488500
H -0.727100 -1.222000 -0.395900
H -0.371300 1.481200 -0.716100
H 0.494000 -0.863200 1.932000
H 2.865500 -0.873000 -2.938900
H 2.339300 1.084800 1.327600
H -1.338700 0.499400 4.243900
H 0.260000 1.143900 3.935700
H -3.072200 0.509400 2.212800
H -2.082500 2.733900 1.908600
H 4.737300 0.758600 1.889000
H 5.380500 -0.701600 1.134700
H 5.753300 0.878100 0.452500
H -1.092700 2.782000 4.740200
H -2.179300 -1.726400 1.880700

LNA-OH-Free_14 Eopt
-1063.342276
C -0.886700 -0.414000 0.308400
C 0.072400 0.753100 0.012800
O 0.000100 1.492200 1.212800
C -0.893800 0.805100 2.112800
C -0.553700 -0.641400 1.776100
N 1.450700 0.443500 -0.378500
C 1.618500 -0.253800 -1.560900
N 2.885700 -0.706400 -1.774900
C 3.990600 -0.609500 -0.993500
C 3.757800 0.119500 0.257300
C 2.515000 0.595800 0.492600
C -0.757600 1.245500 3.589500
O -1.139600 2.597500 3.764300
O -1.301200 -1.671600 2.420100
C -2.327100 0.867000 1.545500
O -2.214500 0.126500 0.368300
O -2.638700 2.184400 1.326500
C 4.926500 0.309500 1.217200
O 5.051800 -1.110100 -1.353900
O 0.740600 -0.500700 -2.384700
H -0.773700 -1.266600 -0.363500
H -0.299700 1.365400 -0.815700
H 0.510200 -0.848300 1.920500
H 3.022800 -1.208800 -2.635000
H 2.291100 1.160200 1.387200
H -1.383700 0.620000 4.227900
H 0.270400 1.120900 3.932200
H -3.053700 0.369300 2.197900
H -1.919500 2.543400 0.795100
H 4.651100 0.878200 2.105600
H 5.314200 -0.657700 1.542200
H 5.742300 0.839500 0.721600

H -1.893200 2.769800 3.182100
H -0.909300 -1.885400 3.271900

LNA-OH-Free_15 Eopt
-1063.340323
C -0.881700 -0.406200 0.299200
C 0.037900 0.791100 0.019000
O 0.038000 1.466700 1.256800
C -0.898200 0.797100 2.126500
C -0.555700 -0.639500 1.770200
N 1.397400 0.607100 -0.493600
C 2.031100 1.729700 -0.999800
N 3.345800 1.543800 -1.305000
C 4.122600 0.438100 -1.187300
C 3.405000 -0.723200 -0.652200
C 2.098700 -0.574900 -0.337800
C -0.778400 1.234800 3.605700
O -1.357400 0.283100 4.494500
O -1.296000 -1.663700 2.419300
C -2.320600 0.887000 1.536000
O -2.219600 0.104700 0.379300
O -2.582400 2.207200 1.274800
C 4.166300 -2.033000 -0.483200
O 5.305100 0.470200 -1.515900
O 1.525900 2.834500 -1.191200
H -0.759200 -1.242800 -0.388800
H -0.452300 1.411500 -0.741400
H 0.503600 -0.824400 1.961200
H 3.817000 2.353100 -1.670300
H 1.530100 -1.405400 0.047500
H 0.276000 1.350600 3.862800
H -1.238400 2.214900 3.745700
H -3.070800 0.437600 2.195700
H -1.789000 2.567000 0.863900
H 3.544100 -2.833500 -0.082500
H 4.566000 -2.367100 -1.442600
H 5.012300 -1.895700 0.193100
H -1.355900 0.652000 5.385300
H -1.427200 -1.367900 3.334400

LNA-OH-Free_16 Eopt
-1063.338148
C -0.885000 -0.416200 0.309900
C 0.090600 0.744000 0.010200
O -0.043800 1.522000 1.177100
C -0.892400 0.808800 2.102300
C -0.559400 -0.641200 1.779700
N 1.519100 0.551800 -0.265100
C 2.038200 -0.696000 -0.571900
N 3.327000 -0.674300 -1.015600
C 4.163800 0.371700 -1.228300
C 3.564600 1.669600 -0.907000
C 2.291800 1.682400 -0.454200
C -0.750300 1.280000 3.567900
O 0.557900 1.040600 4.070400
O -1.322800 -1.659100 2.427900
C -2.328500 0.857600 1.554400
O -2.210600 0.134100 0.365000
O -2.659300 2.171700 1.352800
C 4.406100 2.927000 -1.090100
O 5.299700 0.181500 -1.653900
O 1.483000 -1.789100 -0.496600
H -0.812200 -1.263700 -0.369900
H -0.257000 1.291100 -0.869600
H 0.502800 -0.834900 1.954900
H 3.719200 -1.573800 -1.233000
H 1.791700 2.602000 -0.175500
H -0.982600 2.343400 3.642600
H -1.469300 0.752800 4.197500
H -3.040900 0.342200 2.208100
H -1.888600 2.574200 0.940800
H 3.870100 3.833100 -0.807100
H 5.310800 2.870000 -0.481800
H 4.716100 3.029600 -2.131600
H 0.601800 1.324000 4.987300
H -1.018900 -2.516700 2.114600

LNA-OH-Free_17 Eopt
-1063.341113
C -0.876000 -0.409400 0.302500
C 0.092800 0.764000 0.018000
O -0.015100 1.501400 1.214300
C -0.897900 0.803300 2.113100
C -0.562500 -0.643200 1.769600
N 1.516300 0.588200 -0.294900
C 2.017200 -0.627400 -0.732100
N 3.303400 -0.578100 -1.180700
C 4.149800 0.474500 -1.302300
C 3.565300 1.743100 -0.860200
C 2.297600 1.725600 -0.393400
C -0.754300 1.228700 3.593700
O -1.156300 2.575000 3.789600
O -1.339100 -1.665000 2.388000
C -2.325500 0.879000 1.545300
O -2.213800 0.113500 0.380800
O -2.614200 2.196100 1.299900
C 4.415100 3.005600 -0.942500
O 5.281000 0.313300 -1.751100
O 1.447400 -1.713100 -0.770500
H -0.798800 -1.254700 -0.379700
H -0.270700 1.343300 -0.834700
H 0.496800 -0.855800 1.942200
H 3.682700 -1.457100 -1.487700
H 1.811200 2.624500 -0.033800
H -1.358400 0.579900 4.230500
H 0.279800 1.112900 3.921800
H -3.064100 0.419300 2.211300
H -1.830100 2.565300 0.877500
H 3.890100 3.888000 -0.576000
H 5.325900 2.889500 -0.352000
H 4.715300 3.196400 -1.974500
H -1.949000 2.732300 3.260900
H -2.100000 -1.851400 1.827800

LNA-OH-Free_18 Eopt
-1063.342462
C -0.879500 -0.413200 0.307600
C 0.067900 0.761700 0.034500
O -0.035800 1.537300 1.199300
C -0.890800 0.811400 2.106300
C -0.565900 -0.643400 1.774300
N 1.441100 0.490100 -0.400600
C 2.488800 0.500500 0.511200
N 3.716500 0.320100 -0.052200
C 4.068500 0.135800 -1.349300
C 2.926300 0.133000 -2.268300
C 1.692100 0.309800 -1.747400
C -0.709300 1.283300 3.562800
O 0.557400 0.861300 4.042600
O -1.319200 -1.659900 2.429300
C -2.328300 0.863700 1.554800
O -2.211300 0.124500 0.368200
O -2.650200 2.178400 1.343400
C 3.189500 -0.072600 -3.755500
O 5.247200 -0.011000 -1.658800
O 2.433700 0.647400 1.733300
H -0.741800 -1.261800 -0.363100
H -0.333700 1.391300 -0.763700
H 0.487500 -0.874900 1.934500
H 4.487200 0.327000 0.593600
H 0.808800 0.308000 -2.373300
H -0.790500 2.369900 3.622300
H -1.485100 0.867700 4.207400
H -3.049300 0.359200 2.207700
H -1.863100 2.589700 0.972300
H 2.276200 -0.063700 -4.350500
H 3.845500 0.712300 -4.136900
H 3.690900 -1.027500 -3.924800
H 1.222100 1.060200 3.360800
H -1.007800 -1.725300 3.339300

LNA-OH-Free_19 Eopt
-1063.337521
C -0.886000 -0.407100 0.307600
C 0.053200 0.787200 0.014300
O 0.031400 1.479000 1.239800
C -0.890100 0.803800 2.116900
C -0.557300 -0.641000 1.776900
N 1.441200 0.651800 -0.431500
C 2.028700 -0.584500 -0.641000
N 3.272800 -0.525200 -1.195200
C 4.013100 0.545700 -1.577200
C 3.347500 1.828700 -1.334800
C 2.114000 1.806100 -0.784400
C -0.774700 1.270900 3.582300
O 0.504100 0.953400 4.103800
O -1.305400 -1.654600 2.445600
C -2.323100 0.868100 1.540800
O -2.219300 0.119400 0.369000
O -2.662300 2.178900 1.312000
C 4.081300 3.112600 -1.703800
O 5.125800 0.387800 -2.071600
O 1.570400 -1.695500 -0.392700
H -0.786000 -1.252300 -0.373000
H -0.402200 1.387800 -0.777800
H 0.506800 -0.848000 1.921700
H 3.712000 -1.415800 -1.351400
H 1.567800 2.714000 -0.556000
H -0.947700 2.346600 3.646600
H -1.533400 0.787900 4.199700
H -3.040000 0.354000 2.191000
H -3.540500 2.187000 0.922600
H 3.504100 4.006700 -1.467400
H 5.030900 3.175300 -1.169000
H 4.304600 3.128900 -2.772100
H 1.154300 1.415500 3.563800
H -0.901400 -1.807300 3.306300

LNA-OH-Free_2 Eopt
-1063.345859
C -0.873400 -0.403800 0.299600
C 0.078000 0.775300 0.026200
O 0.006100 1.494000 1.236900
C -0.898400 0.800600 2.120100
C -0.564700 -0.643600 1.765700
N 1.456400 0.474700 -0.370100
C 1.625400 -0.176900 -1.578100
N 2.895800 -0.609600 -1.813400

| | | | |
|---|---|---|---|
| C | 4.001100 | -0.537900 | -1.029900 |
| C | 3.763000 | 0.128200 | 0.254600 |
| C | 2.517800 | 0.587900 | 0.510200 |
| C | -0.769800 | 1.212700 | 3.605200 |
| O | -1.145400 | 2.565800 | 3.799300 |
| O | -1.338800 | -1.676700 | 2.365300 |
| C | -2.322400 | 0.888000 | 1.539600 |
| O | -2.216900 | 0.101900 | 0.386100 |
| O | -2.590300 | 2.206100 | 1.276600 |
| C | 4.928000 | 0.271800 | 1.226700 |
| O | 5.066300 | -1.009400 | -1.416800 |
| O | 0.743900 | -0.401300 | -2.405200 |
| H | -0.738900 | -1.245800 | -0.382500 |
| H | -0.296600 | 1.401600 | -0.790700 |
| H | 0.492700 | -0.858800 | 1.943500 |
| H | 3.036700 | -1.073800 | -2.694100 |
| H | 2.289000 | 1.102200 | 1.433600 |
| H | -1.398800 | 0.574700 | 4.228500 |
| H | 0.255600 | 1.074000 | 3.950900 |
| H | -3.073600 | 0.452500 | 2.207500 |
| H | -1.813500 | 2.553400 | 0.822500 |
| H | 4.646400 | 0.789200 | 2.144100 |
| H | 5.318700 | -0.709900 | 1.500800 |
| H | 5.742900 | 0.831600 | 0.763800 |
| H | -1.900300 | 2.755500 | 3.226800 |
| H | -2.170500 | -1.753400 | 1.884500 |

LNA-OH-Free_20 Eopt
-1063.340575

| | | | |
|---|---|---|---|
| C | -0.880900 | -0.397200 | 0.302400 |
| C | 0.057400 | 0.797100 | 0.031200 |
| O | 0.052000 | 1.461700 | 1.274100 |
| C | -0.896400 | 0.797000 | 2.127900 |
| C | -0.555600 | -0.645700 | 1.768400 |
| N | 1.410600 | 0.522800 | -0.457200 |
| C | 1.512500 | -0.098700 | -1.688400 |
| N | 2.771400 | -0.508100 | -2.011500 |
| C | 3.920300 | -0.447100 | -1.292800 |
| C | 3.754200 | 0.185900 | 0.019200 |
| C | 2.522300 | 0.625600 | 0.360100 |
| C | -0.789700 | 1.219300 | 3.609400 |
| O | -1.535500 | 0.344200 | 4.441200 |
| O | -1.301300 | -1.668400 | 2.433000 |
| C | -2.319700 | 0.891000 | 1.529200 |
| O | -2.223100 | 0.097900 | 0.383300 |
| O | -2.603500 | 2.204800 | 1.245100 |
| C | 4.974800 | 0.320900 | 0.922000 |
| O | 4.963400 | -0.903900 | -1.751400 |
| O | 0.587400 | -0.325300 | -2.465600 |
| H | -0.747900 | -1.227500 | -0.392100 |
| H | -0.366100 | 1.467200 | -0.725100 |
| H | 0.509000 | -0.847100 | 1.920400 |
| H | 2.862500 | -0.948100 | -2.910900 |
| H | 2.344300 | 1.119200 | 1.305400 |
| H | 0.255100 | 1.194700 | 3.921700 |
| H | -1.134000 | 2.246200 | 3.742400 |
| H | -3.061400 | 0.439300 | 2.198000 |
| H | -3.498200 | 2.244300 | 0.899200 |
| H | 4.744200 | 0.814500 | 1.866400 |
| H | 5.392500 | -0.661700 | 1.148600 |
| H | 5.753400 | 0.901400 | 0.423300 |
| H | -1.458700 | -0.546100 | 4.063000 |
| H | -1.233200 | -2.489600 | 1.933700 |

LNA-OH-Free_3 Eopt
-1063.344606

| | | | |
|---|---|---|---|
| C | -0.885100 | -0.413800 | 0.307300 |
| C | 0.068400 | 0.758800 | 0.018200 |
| O | 0.006000 | 1.491700 | 1.225600 |
| C | -0.896800 | 0.802900 | 2.116400 |
| C | -0.552900 | -0.640900 | 1.772500 |
| N | 1.441100 | 0.448700 | -0.389300 |
| C | 1.599000 | -0.175900 | -1.613000 |
| N | 2.862000 | -0.624200 | -1.859100 |
| C | 3.970100 | -0.584000 | -1.077200 |
| C | 3.743400 | 0.055600 | 0.222700 |
| C | 2.505300 | 0.526800 | 0.490900 |
| C | -0.767700 | 1.257200 | 3.590900 |
| O | -1.310300 | 0.302700 | 4.498600 |
| O | -1.295800 | -1.654600 | 2.436900 |
| C | -2.325700 | 0.873400 | 1.543600 |
| O | -2.215300 | 0.121500 | 0.371500 |
| O | -2.624700 | 2.190700 | 1.311200 |
| C | 4.911300 | 0.158500 | 1.196600 |
| O | 5.030100 | -1.057300 | -1.476600 |
| O | 0.717500 | -0.360600 | -2.449100 |
| H | -0.764900 | -1.264700 | -0.365500 |
| H | -0.315400 | 1.378200 | -0.800000 |
| H | 0.508300 | -0.846500 | 1.933100 |
| H | 2.992700 | -1.070500 | -2.750600 |
| H | 2.282100 | 1.019300 | 1.426100 |
| H | 0.286700 | 1.399500 | 3.833400 |
| H | -1.248600 | 2.227500 | 3.729100 |
| H | -3.056700 | 0.391700 | 2.202500 |
| H | -1.881700 | 2.551700 | 0.819300 |
| H | 4.638800 | 0.656000 | 2.127400 |
| H | 5.288000 | -0.835800 | 1.444300 |
| H | 5.733700 | 0.717300 | 0.745800 |
| H | -1.329800 | 0.687900 | 5.382200 |
| H | -1.398800 | -1.363700 | 3.356300 |

LNA-OH-Free_4 Eopt
-1063.345725

| | | | |
|---|---|---|---|
| C | -0.882400 | -0.409100 | 0.306300 |
| C | 0.071500 | 0.762200 | 0.016400 |
| O | -0.013900 | 1.515800 | 1.205500 |
| C | -0.891600 | 0.807100 | 2.109000 |
| C | -0.562200 | -0.643000 | 1.776900 |
| N | 1.452300 | 0.445300 | -0.356600 |
| C | 1.639800 | -0.157500 | -1.586900 |
| N | 2.912200 | -0.587600 | -1.816400 |
| C | 3.999800 | -0.566400 | -1.005400 |
| C | 3.735600 | 0.028700 | 0.308500 |
| C | 2.490000 | 0.491100 | 0.556800 |
| C | -0.760600 | 1.264400 | 3.578500 |
| O | 0.603300 | 1.297000 | 3.969400 |
| O | -1.334900 | -1.651600 | 2.428500 |
| C | -2.325800 | 0.866400 | 1.548600 |
| O | -2.213700 | 0.121700 | 0.370300 |
| O | -2.642100 | 2.180800 | 1.325400 |
| C | 4.871000 | 0.086500 | 1.323500 |
| O | 5.070600 | -1.024800 | -1.392400 |
| O | 0.773300 | -0.344600 | -2.438300 |
| H | -0.760500 | -1.249800 | -0.378500 |
| H | -0.298900 | 1.370600 | -0.816400 |
| H | 0.496000 | -0.850500 | 1.953200 |
| H | 3.068600 | -1.008800 | -2.716000 |
| H | 2.237200 | 0.940400 | 1.506500 |
| H | -1.193600 | 2.257600 | 3.708600 |
| H | -1.310900 | 0.589100 | 4.236300 |
| H | -3.048000 | 0.372500 | 2.208800 |
| H | -1.894700 | 2.560700 | 0.854300 |
| H | 4.561800 | 0.523500 | 2.273400 |
| H | 5.253500 | -0.915700 | 1.525900 |
| H | 5.699300 | 0.681000 | 0.933600 |
| H | 1.014400 | 2.039700 | 3.518100 |
| H | -1.100700 | -2.517600 | 2.082200 |

LNA-OH-Free_5 Eopt
-1063.341472

| | | | |
|---|---|---|---|
| C | -0.886000 | -0.415400 | 0.307600 |
| C | 0.081000 | 0.751100 | 0.009400 |
| O | -0.009300 | 1.495900 | 1.201800 |
| C | -0.893300 | 0.806000 | 2.109700 |
| C | -0.556800 | -0.639900 | 1.777300 |
| N | 1.496900 | 0.568600 | -0.326600 |
| C | 2.032100 | -0.684900 | -0.577100 |
| N | 3.298500 | -0.663000 | -1.081400 |
| C | 4.103300 | 0.385700 | -1.385300 |
| C | 3.492500 | 1.687700 | -1.104200 |
| C | 2.239000 | 1.701700 | -0.600400 |
| C | -0.747500 | 1.239700 | 3.588000 |
| O | -1.153000 | 2.585300 | 3.778900 |
| O | -1.309300 | -1.654700 | 2.438100 |
| C | -2.327200 | 0.863800 | 1.549100 |
| O | -2.212400 | 0.128700 | 0.367400 |
| O | -2.643900 | 2.180700 | 1.334400 |
| C | 4.302400 | 2.948500 | -1.382000 |
| O | 5.223300 | 0.193400 | -1.849500 |
| O | 1.507200 | -1.781100 | -0.399100 |
| H | -0.807300 | -1.260000 | -0.376200 |
| H | -0.301500 | 1.316100 | -0.845000 |
| H | 0.506300 | -0.837000 | 1.943400 |
| H | 3.702200 | -1.565100 | -1.265900 |
| H | 1.732800 | 2.625700 | -0.346200 |
| H | -1.350200 | 0.592800 | 4.228100 |
| H | 0.287200 | 1.128300 | 3.914900 |
| H | -3.047200 | 0.360100 | 2.204900 |
| H | -1.878200 | 2.567400 | 0.895700 |
| H | 3.763000 | 3.858700 | -1.118900 |
| H | 5.233500 | 2.935700 | -0.812200 |
| H | 4.565800 | 3.006800 | -2.439700 |
| H | -1.961100 | 2.724900 | 3.267800 |
| H | -1.067600 | -2.511600 | 2.071100 |

LNA-OH-Free_6 Eopt
-1063.342276

| | | | |
|---|---|---|---|
| C | -0.883600 | -0.403600 | 0.305200 |
| C | 0.063500 | 0.778500 | 0.021500 |
| O | 0.033900 | 1.473500 | 1.245500 |
| C | -0.891400 | 0.801600 | 2.119900 |
| C | -0.558200 | -0.642300 | 1.774300 |
| N | 1.426600 | 0.488800 | -0.429700 |
| C | 1.553700 | -0.170100 | -1.638800 |
| N | 2.818000 | -0.592500 | -1.921200 |
| C | 3.951500 | -0.510500 | -1.180300 |
| C | 3.759300 | 0.165900 | 0.106200 |
| C | 2.521500 | 0.618000 | 0.406700 |
| C | -0.761600 | 1.236100 | 3.595900 |
| O | -1.148400 | 2.589600 | 3.735700 |
| O | -1.318600 | -1.653500 | 2.434600 |
| C | -2.322800 | 0.875700 | 1.537900 |
| O | -2.219900 | 0.111800 | 0.374000 |
| O | -2.641700 | 2.190200 | 1.289300 |
| C | 4.961300 | 0.330200 | 1.028900 |
| O | 5.002600 | -0.984700 | -1.601600 |
| O | 0.645000 | -0.416600 | -2.429300 |
| H | -0.758600 | -1.242000 | -0.381100 |

| | | | |
|---|---|---|---|
| H | -0.340400 | 1.429300 | -0.761600 |
| H | 0.504400 | -0.847600 | 1.932800 |
| H | 2.927600 | -1.061100 | -2.804000 |
| H | 2.325100 | 1.143600 | 1.331300 |
| H | -1.390100 | 0.613600 | 4.234800 |
| H | 0.265400 | 1.115000 | 3.942500 |
| H | -3.045900 | 0.385400 | 2.200400 |
| H | -3.507500 | 2.203600 | 0.870900 |
| H | 4.711900 | 0.857200 | 1.950300 |
| H | 5.371800 | -0.644400 | 1.298700 |
| H | 5.751500 | 0.891800 | 0.526900 |
| H | -1.534600 | 2.849100 | 2.884400 |
| H | -1.145600 | -2.508200 | 2.027900 |

LNA-OH-Free_7 Eopt
-1063.342169

| | | | |
|---|---|---|---|
| C | -0.884500 | -0.413800 | 0.308600 |
| C | 0.077700 | 0.756600 | 0.007500 |
| O | -0.022400 | 1.513800 | 1.193000 |
| C | -0.892700 | 0.807100 | 2.105100 |
| C | -0.559200 | -0.641500 | 1.778700 |
| N | 1.495000 | 0.579200 | -0.324800 |
| C | 2.042400 | -0.674300 | -0.548500 |
| N | 3.318100 | -0.651100 | -1.028500 |
| C | 4.119500 | 0.398600 | -1.338300 |
| C | 3.490700 | 1.700400 | -1.098900 |
| C | 2.229300 | 1.713400 | -0.615400 |
| C | -0.751400 | 1.277900 | 3.568900 |
| O | 0.603900 | 1.205200 | 3.978600 |
| O | -1.323400 | -1.658200 | 2.424900 |
| C | -2.327500 | 0.861700 | 1.551600 |
| O | -2.211900 | 0.129600 | 0.367100 |
| O | -2.652800 | 2.175900 | 1.340000 |
| C | 4.290000 | 2.962700 | -1.399800 |
| O | 5.250800 | 0.208600 | -1.774900 |
| O | 1.521000 | -1.771400 | -0.368500 |
| H | -0.803200 | -1.259200 | -0.374100 |
| H | -0.306900 | 1.309200 | -0.854000 |
| H | 0.501900 | -0.838300 | 1.954600 |
| H | 3.731800 | -1.553500 | -1.188200 |
| H | 1.708300 | 2.638200 | -0.399200 |
| H | -1.104600 | 2.305000 | 3.673000 |
| H | -1.361600 | 0.660700 | 4.230900 |
| H | -3.043300 | 0.354800 | 2.209300 |
| H | -1.891800 | 2.573500 | 0.908300 |
| H | 3.734500 | 3.874100 | -1.178200 |
| H | 5.209700 | 2.979500 | -0.811600 |
| H | 4.575000 | 2.990400 | -2.453200 |
| H | 1.124000 | 1.717300 | 3.352200 |
| H | -1.008600 | -2.518600 | 2.130100 |

LNA-OH-Free_8 Eopt
-1063.340825

| | | | |
|---|---|---|---|
| C | -0.885900 | -0.407800 | 0.306800 |
| C | 0.058100 | 0.774600 | 0.022500 |
| O | 0.043200 | 1.465600 | 1.252700 |
| C | -0.893800 | 0.801500 | 2.122300 |
| C | -0.553600 | -0.641100 | 1.772800 |
| N | 1.412300 | 0.479900 | -0.448200 |
| C | 1.521400 | -0.108800 | -1.694800 |
| N | 2.774000 | -0.548000 | -2.003100 |
| C | 3.911100 | -0.533200 | -1.263200 |
| C | 3.735400 | 0.066300 | 0.063400 |
| C | 2.509300 | 0.529700 | 0.392800 |
| C | -0.773600 | 1.254300 | 3.595400 |
| O | -1.347700 | 0.313600 | 4.499700 |
| O | -1.298800 | -1.653100 | 2.439200 |
| C | -2.322300 | 0.875400 | 1.537300 |
| O | -2.220200 | 0.115100 | 0.372000 |
| O | -2.644500 | 2.187800 | 1.294300 |
| C | 4.940200 | 0.140000 | 0.994200 |
| O | 4.953800 | -0.996400 | -1.716900 |
| O | 0.608900 | -0.275400 | -2.501600 |
| H | -0.758200 | -1.253800 | -0.370000 |
| H | -0.357400 | 1.431300 | -0.749700 |
| H | 0.508300 | -0.848300 | 1.928700 |
| H | 2.869100 | -0.967000 | -2.912000 |
| H | 2.321200 | 0.996000 | 1.349100 |
| H | 0.280700 | 1.376100 | 3.848600 |
| H | -1.234600 | 2.235800 | 3.721500 |
| H | -3.046400 | 0.381000 | 2.194500 |
| H | -3.496400 | 2.196700 | 0.850700 |
| H | 4.703500 | 0.611100 | 1.948300 |
| H | 5.324100 | -0.861200 | 1.198800 |
| H | 5.745300 | 0.711500 | 0.528500 |
| H | -1.330100 | 0.689100 | 5.387200 |
| H | -1.392700 | -1.361400 | 3.359200 |

LNA-OH-Free_9 Eopt
-1063.343898

| | | | |
|---|---|---|---|
| C | -0.880900 | -0.404600 | 0.303300 |
| C | 0.069000 | 0.777300 | 0.025100 |
| O | 0.009500 | 1.492500 | 1.241400 |
| C | -0.898700 | 0.799600 | 2.120900 |
| C | -0.555200 | -0.645000 | 1.768900 |
| N | 1.443500 | 0.481700 | -0.389800 |
| C | 1.599800 | -0.180400 | -1.594000 |
| N | 2.870000 | -0.604700 | -1.845100 |
| C | 3.985300 | -0.521100 | -1.077400 |
| C | 3.761600 | 0.155500 | 0.204100 |
| C | 2.517300 | 0.609400 | 0.473600 |
| C | -0.774600 | 1.225900 | 3.601900 |
| O | -1.493300 | 0.344500 | 4.448400 |
| O | -1.301900 | -1.661900 | 2.439400 |
| C | -2.323600 | 0.886300 | 1.537300 |
| O | -2.217300 | 0.106800 | 0.380800 |
| O | -2.594200 | 2.203600 | 1.272500 |
| C | 4.939900 | 0.315900 | 1.157500 |
| O | 5.046900 | -0.993200 | -1.472900 |
| O | 0.708600 | -0.424500 | -2.404200 |
| H | -0.756500 | -1.240700 | -0.386700 |
| H | -0.317300 | 1.405700 | -0.785300 |
| H | 0.508400 | -0.846700 | 1.924900 |
| H | 3.001100 | -1.074800 | -2.724300 |
| H | 2.300100 | 1.131700 | 1.394700 |
| H | 0.275000 | 1.214300 | 3.898600 |
| H | -1.130700 | 2.247600 | 3.744000 |
| H | -3.072500 | 0.440600 | 2.202000 |
| H | -1.795900 | 2.572300 | 0.883800 |
| H | 4.669300 | 0.838900 | 2.075000 |
| H | 5.343100 | -0.660300 | 1.433300 |
| H | 5.743400 | 0.877800 | 0.677300 |
| H | -1.447700 | -0.541800 | 4.054600 |
| H | -1.293600 | -2.469300 | 1.913200 |

**Cartesian coordinates for compound 22**

LNA-AldeAcet_1 Eopt
-1180.043947

| | | | |
|---|---|---|---|
| C | 0.447300 | 1.563700 | 7.354400 |
| C | -1.091300 | 1.821200 | 7.415200 |
| O | -1.659600 | 1.232400 | 6.252300 |
| C | -0.663800 | 1.160200 | 5.227500 |
| C | 0.474400 | 0.687700 | 6.105900 |
| N | -1.835300 | 1.403700 | 8.602700 |
| C | -1.744000 | 0.086200 | 9.019300 |
| N | -2.500900 | -0.206600 | 10.114300 |
| C | -3.314700 | 0.587000 | 10.854900 |
| C | -3.376900 | 1.970000 | 10.371500 |
| C | -2.642200 | 2.297100 | 9.285000 |
| C | -0.829500 | 0.079000 | 4.154600 |
| O | 0.424400 | -0.122200 | 3.466000 |
| C | 1.585300 | -0.311500 | 4.258400 |
| O | 1.680600 | 0.597400 | 5.344600 |
| C | -0.418200 | 2.524400 | 4.521900 |
| O | 1.153400 | 2.787600 | 7.210700 |
| C | 1.601100 | -1.752400 | 4.769300 |
| C | 2.748300 | 0.024200 | 3.352700 |
| O | -1.215800 | 3.460200 | 4.610900 |
| C | -4.267600 | 2.960000 | 11.112400 |
| O | -3.911200 | 0.129100 | 11.825600 |
| O | -1.062900 | -0.811000 | 8.524900 |
| H | 0.797200 | 1.007400 | 8.226800 |
| H | -1.267800 | 2.896200 | 7.327400 |
| H | 0.269800 | -0.319300 | 6.469800 |
| H | -2.446700 | -1.159800 | 10.428300 |
| H | -2.658100 | 3.299300 | 8.878500 |
| H | -1.184600 | -0.859900 | 4.581400 |
| H | -1.594800 | 0.359000 | 3.428600 |
| H | 0.504300 | 2.583600 | 3.944200 |
| H | 2.073300 | 2.577700 | 7.015200 |
| H | 0.674800 | -2.030900 | 5.266700 |
| H | 1.739000 | -2.433300 | 3.929600 |
| H | 2.429500 | -1.886500 | 5.464700 |
| H | 2.673700 | 1.060100 | 3.022100 |
| H | 3.688200 | -0.108800 | 3.888000 |
| H | 2.741200 | -0.625100 | 2.477300 |
| H | -4.248100 | 3.954400 | 10.666000 |
| H | -5.302700 | 2.613500 | 11.112900 |
| H | -3.950800 | 3.052300 | 12.152800 |

LNA-AldeAcet_10 Eopt
-1180.039871

| | | | |
|---|---|---|---|
| C | 0.439800 | 1.631500 | 7.353300 |
| C | -1.099800 | 1.824200 | 7.430400 |
| O | -1.649000 | 1.236000 | 6.253500 |
| C | -0.665900 | 1.153600 | 5.211100 |
| C | 0.475100 | 0.733400 | 6.125700 |
| N | -1.769000 | 1.279100 | 8.610800 |
| C | -2.399200 | 2.138100 | 9.495200 |
| N | -2.984600 | 1.514000 | 10.556700 |
| C | -3.048200 | 0.195000 | 10.870700 |
| C | -2.391300 | -0.670700 | 9.885600 |
| C | -1.796800 | -0.084700 | 8.823100 |
| C | -0.831100 | -0.001800 | 4.184400 |
| O | 0.278000 | -0.929000 | 4.190200 |
| C | 1.583600 | -0.398700 | 4.378600 |
| O | 1.675700 | 0.614300 | 5.369300 |
| C | -0.469300 | 2.539000 | 4.520500 |
| O | 1.096200 | 2.876800 | 7.174500 |
| C | 2.421800 | -1.559200 | 4.870300 |
| C | 2.049100 | 0.210700 | 3.069800 |
| O | -0.456800 | 2.689900 | 3.297100 |
| C | -2.428900 | -2.178900 | 10.100300 |
| O | -3.615700 | -0.173700 | 11.895100 |
| O | -2.463700 | 3.363400 | 9.421900 |

| | | | |
|---|---|---|---|
| H | 0.814100 | 1.106600 | 8.235700 |
| H | -1.336600 | 2.893700 | 7.389100 |
| H | 0.297900 | -0.274700 | 6.514000 |
| H | -3.436700 | 2.125400 | 11.214300 |
| H | -1.321200 | -0.660600 | 8.043600 |
| H | -1.726000 | -0.572200 | 4.433600 |
| H | -1.001000 | 0.327000 | 3.158400 |
| H | -0.358300 | 3.383300 | 5.199500 |
| H | 2.030800 | 2.707400 | 7.007800 |
| H | 2.019100 | -1.941700 | 5.807500 |
| H | 2.415400 | -2.360700 | 4.131700 |
| H | 3.449100 | -1.233700 | 5.032400 |
| H | 1.418900 | 1.048200 | 2.781600 |
| H | 3.072900 | 0.569400 | 3.172700 |
| H | 2.014900 | -0.539100 | 2.279800 |
| H | -1.937300 | -2.727900 | 9.297000 |
| H | -1.937400 | -2.441500 | 11.038800 |
| H | -3.461300 | -2.528500 | 10.161400 |

LNA-AldeAcet_2 Eopt
-1180.039433

| | | | |
|---|---|---|---|
| C | 0.447600 | 1.633600 | 7.343700 |
| C | -1.097300 | 1.822800 | 7.424500 |
| O | -1.652100 | 1.235200 | 6.253600 |
| C | -0.665300 | 1.155900 | 5.216900 |
| C | 0.495100 | 0.749700 | 6.102600 |
| N | -1.811800 | 1.353000 | 8.613200 |
| C | -1.664500 | 0.033400 | 9.007000 |
| N | -2.398800 | -0.308900 | 10.102900 |
| C | -3.235200 | 0.438400 | 10.865600 |
| C | -3.354300 | 1.826800 | 10.409600 |
| C | -2.644500 | 2.202000 | 9.322100 |
| C | -0.794700 | 0.007700 | 4.200900 |
| O | 0.460600 | -0.195500 | 3.516100 |
| C | 1.643500 | -0.267500 | 4.293700 |
| O | 1.700600 | 0.687500 | 5.343300 |
| C | -0.492400 | 2.528100 | 4.497000 |
| O | 1.092400 | 2.888300 | 7.179300 |
| C | 1.758800 | -1.680800 | 4.863400 |
| C | 2.764800 | 0.105600 | 3.351400 |
| O | -0.611900 | 2.670400 | 3.278100 |
| C | -4.272000 | 2.768700 | 11.179900 |
| O | -3.804800 | -0.059500 | 11.832600 |
| O | -0.953200 | -0.827300 | 8.493200 |
| H | 0.834300 | 1.100500 | 8.215500 |
| H | -1.318900 | 2.890200 | 7.366700 |
| H | 0.334500 | -0.258300 | 6.483300 |
| H | -2.304100 | -1.265000 | 10.398700 |
| H | -2.703400 | 3.211700 | 8.940100 |
| H | -1.113200 | -0.918800 | 4.680300 |
| H | -1.569900 | 0.218400 | 3.462700 |
| H | -0.268000 | 3.369900 | 5.149400 |
| H | 2.016200 | 2.714700 | 6.959800 |
| H | 0.851600 | -1.998900 | 5.372300 |
| H | 1.941300 | -2.384100 | 4.051200 |
| H | 2.594800 | -1.729900 | 5.560700 |
| H | 2.606400 | 1.117500 | 2.977100 |
| H | 3.721000 | 0.064800 | 3.872000 |
| H | 2.786600 | -0.580400 | 2.504900 |
| H | -4.293200 | 3.772900 | 10.756300 |
| H | -5.294000 | 2.385000 | 11.183300 |
| H | -3.948500 | 2.849400 | 12.219400 |

LNA-AldeAcet_3 Eopt
-1180.041831

| | | | |
|---|---|---|---|
| C | 0.445600 | 1.567900 | 7.345500 |
| C | -1.091700 | 1.822200 | 7.418100 |
| O | -1.659600 | 1.232200 | 6.251800 |
| C | -0.663400 | 1.159400 | 5.225100 |
| C | 0.457400 | 0.668700 | 6.111100 |
| N | -1.770200 | 1.409800 | 8.643500 |
| C | -1.376600 | 2.050600 | 9.805900 |
| N | -1.885300 | 1.506600 | 10.947400 |
| C | -2.653000 | 0.400800 | 11.119500 |
| C | -2.985000 | -0.280400 | 9.863500 |
| C | -2.524100 | 0.253400 | 8.710200 |
| C | -0.854200 | 0.091200 | 4.142700 |
| O | 0.395300 | -0.161200 | 3.468200 |
| C | 1.537600 | -0.395000 | 4.273200 |
| O | 1.658600 | 0.512500 | 5.354700 |
| C | -0.391500 | 2.525200 | 4.534000 |
| O | 1.118500 | 2.813300 | 7.225300 |
| C | 1.487700 | -1.831800 | 4.791800 |
| C | 2.723500 | -0.111200 | 3.379600 |
| O | -1.228100 | 3.430700 | 4.540700 |
| C | -3.830500 | -1.546700 | 9.923800 |
| O | -2.994500 | 0.053300 | 12.246200 |
| O | -0.606800 | 3.009900 | 9.877800 |
| H | 0.795500 | 1.013000 | 8.219300 |
| H | -1.330600 | 2.891200 | 7.350800 |
| H | 0.216000 | -0.323500 | 6.498100 |
| H | -1.628600 | 1.973600 | 11.800100 |
| H | -2.744900 | -0.193800 | 7.751400 |
| H | -1.252400 | -0.835100 | 4.557500 |
| H | -1.596900 | 0.405000 | 3.406800 |
| H | 0.581800 | 2.612700 | 4.050300 |
| H | 1.060600 | 3.277600 | 8.070400 |
| H | 0.549700 | -2.064400 | 5.289300 |
| H | 1.597700 | -2.521700 | 3.955300 |
| H | 2.310200 | -1.997200 | 5.487700 |
| H | 2.694800 | 0.926500 | 3.047000 |
| H | 3.650700 | -0.277500 | 3.927800 |
| H | 2.701400 | -0.762200 | 2.506000 |
| H | -4.022700 | -1.967700 | 8.936800 |
| H | -3.332500 | -2.309800 | 10.524700 |
| H | -4.794500 | -1.337500 | 10.391600 |

LNA-AldeAcet_4 Eopt
-1180.044108

| | | | |
|---|---|---|---|
| C | 0.454100 | 1.667200 | 7.328700 |
| C | -1.092900 | 1.822100 | 7.418900 |
| O | -1.657000 | 1.232900 | 6.251400 |
| C | -0.664800 | 1.158900 | 5.224700 |
| C | 0.515300 | 0.773800 | 6.095300 |
| N | -1.771600 | 1.343400 | 8.623100 |
| C | -1.687800 | -0.000900 | 8.944500 |
| N | -2.349100 | -0.344500 | 10.085600 |
| C | -3.054100 | 0.422400 | 10.954700 |
| C | -3.090900 | 1.840200 | 10.582400 |
| C | -2.454100 | 2.217400 | 9.451300 |
| C | -0.790400 | 0.017300 | 4.208300 |
| O | 0.451700 | -0.135700 | 3.491300 |
| C | 1.647600 | -0.210700 | 4.252700 |
| O | 1.710000 | 0.725200 | 5.318100 |
| C | -0.565500 | 2.505000 | 4.450400 |
| O | 1.053000 | 2.941100 | 7.158600 |
| C | 1.786700 | -1.631500 | 4.797400 |
| C | 2.753200 | 0.191800 | 3.304200 |
| O | 0.471200 | 3.168900 | 4.377000 |
| C | -3.839100 | 2.810600 | 11.488700 |
| O | -3.584200 | -0.083400 | 11.939900 |
| O | -1.092600 | -0.883100 | 8.329300 |
| H | 0.858800 | 1.151200 | 8.202500 |
| H | -1.345700 | 2.882500 | 7.356200 |
| H | 0.382000 | -0.235400 | 6.482400 |
| H | -2.305200 | -1.319300 | 10.327000 |
| H | -2.446500 | 3.250700 | 9.132800 |
| H | -1.066700 | -0.922100 | 4.689900 |
| H | -1.591300 | 0.205300 | 3.491700 |
| H | -1.496100 | 2.822400 | 3.981100 |
| H | 1.473300 | 2.947500 | 6.284200 |
| H | 0.901100 | -1.960100 | 5.336400 |
| H | 1.945400 | -2.323000 | 3.970300 |
| H | 2.646600 | -1.684700 | 5.465100 |
| H | 2.586000 | 1.213100 | 2.960500 |
| H | 3.715800 | 0.145400 | 3.812800 |
| H | 2.769900 | -0.473200 | 2.441100 |
| H | -3.799600 | 3.837800 | 11.125800 |
| H | -4.889000 | 2.522900 | 11.569300 |
| H | -3.416000 | 2.794200 | 12.494800 |

LNA-AldeAcet_5 Eopt
-1180.041022

| | | | |
|---|---|---|---|
| C | 0.441100 | 1.611900 | 7.348300 |
| C | -1.097000 | 1.823200 | 7.425500 |
| O | -1.652400 | 1.234800 | 6.253000 |
| C | -0.665200 | 1.155800 | 5.216500 |
| C | 0.474200 | 0.717600 | 6.115100 |
| N | -1.776400 | 1.298300 | 8.609400 |
| C | -2.447300 | 2.167200 | 9.453500 |
| N | -3.046600 | 1.560000 | 10.516900 |
| C | -3.092300 | 0.248800 | 10.864800 |
| C | -2.393200 | -0.629200 | 9.920400 |
| C | -1.783100 | -0.059600 | 8.857800 |
| C | -0.811200 | 0.021900 | 4.186400 |
| O | 0.448200 | -0.222800 | 3.525200 |
| C | 1.615900 | -0.333600 | 4.319200 |
| O | 1.686800 | 0.612300 | 5.375100 |
| C | -0.463100 | 2.534200 | 4.513500 |
| O | 1.109800 | 2.851200 | 7.174600 |
| C | 1.678200 | -1.752000 | 4.883100 |
| C | 2.761800 | 0.010200 | 3.395700 |
| O | -0.463200 | 2.670800 | 3.288200 |
| C | -2.405700 | -2.131500 | 10.175600 |
| O | -3.678700 | -0.104900 | 11.883800 |
| O | -2.537300 | 3.388200 | 9.344500 |
| H | 0.810400 | 1.082000 | 8.229700 |
| H | -1.319100 | 2.895600 | 7.374000 |
| H | 0.280500 | -0.283400 | 6.501500 |
| H | -3.528100 | 2.179500 | 11.145600 |
| H | -1.275400 | -0.645900 | 8.107900 |
| H | -1.173200 | -0.897200 | 4.647500 |
| H | -1.561800 | 0.267200 | 3.433000 |
| H | -0.334400 | 3.383000 | 5.183600 |
| H | 2.035000 | 2.672200 | 6.968700 |
| H | 0.753700 | -2.043500 | 5.375400 |
| H | 1.850100 | -2.455400 | 4.068300 |
| H | 2.504500 | -1.831800 | 5.589200 |
| H | 2.639300 | 1.028500 | 3.025000 |
| H | 3.709100 | -0.061300 | 3.929300 |
| H | 2.775900 | -0.670800 | 2.545000 |
| H | -1.881000 | -2.692400 | 9.402300 |
| H | -1.935700 | -2.357600 | 11.134500 |
| H | -3.432200 | -2.500700 | 10.218900 |

LNA-AldeAcet_6 Eopt
-1180.038052

| | | | |
|---|---|---|---|
| C | 0.448100 | 1.692300 | 7.321200 |

| | | | |
|---|---|---|---|
| C | -1.099100 | 1.823900 | 7.429000 |
| O | -1.649500 | 1.235700 | 6.253000 |
| C | -0.666100 | 1.154200 | 5.213000 |
| C | 0.493400 | 0.771000 | 6.105300 |
| N | -1.713400 | 1.304500 | 8.648700 |
| C | -1.375000 | 1.948000 | 9.826800 |
| N | -1.805700 | 1.317400 | 10.955900 |
| C | -2.446900 | 0.130100 | 11.100500 |
| C | -2.718600 | -0.545400 | 9.827000 |
| C | -2.334700 | 0.070400 | 8.686500 |
| C | -0.808300 | -0.009300 | 4.215400 |
| O | 0.442700 | -0.245900 | 3.537700 |
| C | 1.620300 | -0.325000 | 4.320800 |
| O | 1.693400 | 0.651700 | 5.346000 |
| C | -0.499700 | 2.512500 | 4.466800 |
| O | 1.012600 | 2.987900 | 7.176400 |
| C | 1.703900 | -1.725300 | 4.925800 |
| C | 2.753200 | 0.002100 | 3.375400 |
| O | -0.791100 | 2.657400 | 3.277100 |
| C | -3.417400 | -1.899200 | 9.856100 |
| O | -2.735100 | -0.285100 | 12.219000 |
| O | -0.713500 | 2.983000 | 9.918400 |
| H | 0.865400 | 1.179900 | 8.191700 |
| H | -1.421200 | 2.872100 | 7.415600 |
| H | 0.316100 | -0.219900 | 6.529200 |
| H | -1.589900 | 1.782800 | 11.820800 |
| H | -2.516100 | -0.371400 | 7.716900 |
| H | -1.140900 | -0.923000 | 4.708100 |
| H | -1.578800 | 0.196500 | 3.470600 |
| H | -0.114300 | 3.336900 | 5.062800 |
| H | 1.033800 | 3.415500 | 8.042200 |
| H | 0.793100 | -2.007300 | 5.447800 |
| H | 1.865700 | -2.452300 | 4.130100 |
| H | 2.544600 | -1.775500 | 5.617600 |
| H | 2.619100 | 1.009300 | 2.979700 |
| H | 3.705600 | -0.044900 | 3.902900 |
| H | 2.765700 | -0.702500 | 2.544300 |
| H | -3.573700 | -2.310400 | 8.858600 |
| H | -2.830400 | -2.619500 | 10.428800 |
| H | -4.392700 | -1.813000 | 10.338800 |

LNA-AldeAcet_7 Eopt
-1180.044116

| | | | |
|---|---|---|---|
| C | 0.453200 | 1.730900 | 7.306800 |
| C | -1.097300 | 1.822500 | 7.423600 |
| O | -1.652000 | 1.235300 | 6.254000 |
| C | -0.665400 | 1.156100 | 5.217400 |
| C | 0.531400 | 0.819100 | 6.079400 |
| N | -1.743400 | 1.301800 | 8.629100 |
| C | -1.618300 | -0.045000 | 8.926000 |
| N | -2.241600 | -0.423800 | 10.077200 |
| C | -2.943700 | 0.310200 | 10.976300 |
| C | -3.028100 | 1.731800 | 10.627700 |
| C | -2.429400 | 2.143600 | 9.488000 |
| C | -0.789100 | -0.003900 | 4.222100 |
| O | 0.447000 | -0.148600 | 3.489100 |
| C | 1.652000 | -0.189100 | 4.238300 |
| O | 1.708500 | 0.785100 | 5.270100 |
| C | -0.498900 | 2.487100 | 4.428400 |
| O | 0.992900 | 3.038000 | 7.151300 |
| C | 1.817300 | -1.591600 | 4.823800 |
| C | 2.743900 | 0.195400 | 3.265900 |
| O | -0.789900 | 3.582100 | 4.916100 |
| C | -3.781700 | 2.667000 | 11.566000 |
| O | -3.433800 | -0.225000 | 11.966800 |
| O | -1.017900 | -0.904000 | 8.283700 |
| H | 0.883200 | 1.237300 | 8.181400 |
| H | -1.404400 | 2.869200 | 7.376600 |
| H | 0.433500 | -0.185900 | 6.484100 |
| H | -2.166600 | -1.400800 | 10.301100 |
| H | -2.460400 | 3.181300 | 9.185300 |
| H | -1.046100 | -0.938500 | 4.723200 |
| H | -1.604900 | 0.166300 | 3.517800 |
| H | -0.078700 | 2.385200 | 3.427000 |
| H | 0.670100 | 3.413300 | 6.320600 |
| H | 0.936500 | -1.925300 | 5.367800 |
| H | 1.993900 | -2.303400 | 4.017800 |
| H | 2.674900 | -1.608600 | 5.496300 |
| H | 2.565500 | 1.201500 | 2.886900 |
| H | 3.711200 | 0.176100 | 3.767900 |
| H | 2.762600 | -0.500100 | 2.427000 |
| H | -3.781400 | 3.699700 | 11.216800 |
| H | -4.820500 | 2.347300 | 11.667700 |
| H | -3.333500 | 2.649400 | 12.561200 |

LNA-AldeAcet_8 Eopt
-1180.040672

| | | | |
|---|---|---|---|
| C | 0.449600 | 1.618900 | 7.341400 |
| C | -1.092900 | 1.822000 | 7.418700 |
| O | -1.656700 | 1.232900 | 6.251200 |
| C | -0.665100 | 1.158900 | 5.225100 |
| C | 0.484100 | 0.716200 | 6.110600 |
| N | -1.787800 | 1.319400 | 8.603900 |
| C | -2.676200 | 2.145700 | 9.272800 |
| N | -3.292500 | 1.557600 | 10.337100 |
| C | -3.191400 | 0.292700 | 10.818100 |
| C | -2.271200 | -0.551000 | 10.048300 |
| C | -1.631700 | 0.004400 | 8.995400 |
| C | -0.822900 | 0.049100 | 4.178400 |
| O | 0.425900 | -0.155200 | 3.488800 |
| C | 1.598700 | -0.304300 | 4.272700 |
| O | 1.686500 | 0.609700 | 5.353600 |
| C | -0.509000 | 2.521000 | 4.487500 |
| O | 1.106600 | 2.863800 | 7.176100 |
| C | 1.651500 | -1.737900 | 4.797400 |
| C | 2.743700 | 0.054600 | 3.354000 |
| O | 0.564100 | 3.124500 | 4.417700 |
| C | -2.095200 | -2.006100 | 10.465400 |
| O | -3.837800 | -0.052700 | 11.803200 |
| O | -2.951800 | 3.313900 | 9.006700 |
| H | 0.832200 | 1.100600 | 8.223100 |
| H | -1.305200 | 2.896200 | 7.359000 |
| H | 0.293300 | -0.286300 | 6.494300 |
| H | -3.928500 | 2.150600 | 10.841600 |
| H | -0.958900 | -0.565700 | 8.374800 |
| H | -1.160800 | -0.885500 | 4.627700 |
| H | -1.593300 | 0.295700 | 3.445800 |
| H | -1.428800 | 2.905900 | 4.047400 |
| H | 1.405800 | 2.927500 | 6.254600 |
| H | 0.739800 | -2.026300 | 5.314100 |
| H | 1.788300 | -2.423300 | 3.960900 |
| H | 2.497100 | -1.847700 | 5.476300 |
| H | 2.636900 | 1.088400 | 3.023400 |
| H | 3.692000 | -0.048300 | 3.881200 |
| H | 2.745300 | -0.596700 | 2.480300 |
| H | -1.405100 | -2.547300 | 9.818200 |
| H | -1.713900 | -2.063900 | 11.486700 |
| H | -3.054500 | -2.526700 | 10.444000 |

LNA-AldeAcet_9 Eopt
-1180.038729

| | | | |
|---|---|---|---|
| C | 0.444700 | 1.629600 | 7.354300 |
| C | -1.099800 | 1.823600 | 7.428700 |
| O | -1.649200 | 1.236300 | 6.254400 |
| C | -0.665700 | 1.154000 | 5.211900 |
| C | 0.488600 | 0.746200 | 6.113600 |
| N | -1.827100 | 1.356000 | 8.611100 |
| C | -1.655600 | 0.048800 | 9.035300 |
| N | -2.419400 | -0.296500 | 10.109800 |
| C | -3.306900 | 0.437600 | 10.826200 |
| C | -3.453600 | 1.812000 | 10.337400 |
| C | -2.714900 | 2.189500 | 9.270200 |
| C | -0.822400 | -0.004300 | 4.188700 |
| O | 0.322700 | -0.885800 | 4.141300 |
| C | 1.613500 | -0.324100 | 4.338000 |
| O | 1.682500 | 0.681800 | 5.337900 |
| C | -0.481400 | 2.537500 | 4.515500 |
| O | 1.096300 | 2.881300 | 7.191400 |
| C | 2.480400 | -1.468400 | 4.817500 |
| C | 2.058900 | 0.311700 | 3.035100 |
| O | -0.559700 | 2.700000 | 3.295900 |
| C | -4.432100 | 2.736900 | 11.051500 |
| O | -3.896100 | -0.059200 | 11.782000 |
| O | -0.900400 | -0.799200 | 8.566200 |
| H | 0.828900 | 1.095100 | 8.226100 |
| H | -1.313300 | 2.892700 | 7.375000 |
| H | 0.336400 | -0.272800 | 6.483200 |
| H | -2.305400 | -1.243700 | 10.426600 |
| H | -2.794200 | 3.188200 | 8.864100 |
| H | -1.677700 | -0.615300 | 4.477900 |
| H | -1.051800 | 0.321500 | 3.173700 |
| H | -0.288900 | 3.372000 | 5.187300 |
| H | 2.025300 | 2.702400 | 7.000600 |
| H | 2.085000 | -1.868600 | 5.751100 |
| H | 2.492200 | -2.263400 | 4.072200 |
| H | 3.499100 | -1.120000 | 4.984700 |
| H | 1.395900 | 1.125200 | 2.751600 |
| H | 3.068300 | 0.707400 | 3.143000 |
| H | 2.054000 | -0.434100 | 2.240600 |
| H | -4.471900 | 3.730600 | 10.605000 |
| H | -5.439600 | 2.317300 | 11.025100 |
| H | -4.151500 | 2.850700 | 12.100200 |

**Cartesian coordinates for compound 23**

LNA-OHAcet_1 Eopt
-1180.053239

| | | | |
|---|---|---|---|
| C | -4.388900 | 2.448600 | 6.339800 |
| C | -5.651200 | 2.423900 | 5.446700 |
| O | -5.091900 | 2.525100 | 4.148000 |
| C | -3.664800 | 2.600800 | 4.325900 |
| C | -3.480200 | 1.625400 | 5.441500 |
| N | -6.575400 | 1.294300 | 5.581800 |
| C | -7.210400 | 1.155100 | 6.802800 |
| N | -7.886500 | -0.018100 | 6.957500 |
| C | -7.996600 | -1.078900 | 6.118800 |
| C | -7.282100 | -0.896500 | 4.851400 |
| C | -6.617200 | 0.264400 | 4.658700 |
| C | -2.781300 | 2.177000 | 3.148300 |
| O | -1.431900 | 1.940500 | 3.586400 |
| C | -1.263700 | 1.139400 | 4.737800 |
| O | -2.100000 | 1.546100 | 5.800600 |
| C | -3.319000 | 3.947300 | 4.973300 |
| O | -3.833300 | 3.771000 | 6.258600 |
| C | -1.524000 | -0.324000 | 4.378000 |
| C | 0.145500 | 1.412700 | 5.211600 |

| | | | |
|---|---|---|---|
| O | -3.938000 | 4.940900 | 4.262300 |
| C | -7.332100 | -2.017500 | 3.819900 |
| O | -8.642600 | -2.068500 | 6.450200 |
| O | -7.192100 | 1.966700 | 7.725200 |
| H | -4.548600 | 2.082300 | 7.355100 |
| H | -6.281000 | 3.299800 | 5.634600 |
| H | -3.863000 | 0.620100 | 5.258200 |
| H | -8.361600 | -0.128000 | 7.837100 |
| H | -6.089900 | 0.460100 | 3.736400 |
| H | -3.174900 | 1.295000 | 2.643500 |
| H | -2.769400 | 2.962100 | 2.391700 |
| H | -2.236500 | 4.100900 | 5.050600 |
| H | -2.502100 | -0.475500 | 3.928700 |
| H | -0.772000 | -0.658700 | 3.663500 |
| H | -1.447100 | -0.938500 | 5.274900 |
| H | 0.257000 | 2.471800 | 5.445200 |
| H | 0.356600 | 0.831600 | 6.109000 |
| H | 0.861700 | 1.146400 | 4.434600 |
| H | -4.845400 | 4.654200 | 4.130200 |
| H | -6.779900 | -1.777400 | 2.911300 |
| H | -6.914300 | -2.936300 | 4.235700 |
| H | -8.365700 | -2.227200 | 3.537600 |

LNA-OHAcet_2 Eopt
-1180.046441

| | | | |
|---|---|---|---|
| C | -4.404100 | 2.370200 | 6.076900 |
| C | -5.508600 | 1.825300 | 5.131300 |
| O | -4.792300 | 1.623500 | 3.932900 |
| C | -3.474900 | 2.154000 | 4.151300 |
| C | -3.215200 | 1.603100 | 5.512300 |
| N | -6.326700 | 0.646600 | 5.420300 |
| C | -6.065700 | -0.180000 | 6.500100 |
| N | -6.980600 | -1.175900 | 6.674300 |
| C | -8.094200 | -1.479100 | 5.960700 |
| C | -8.317700 | -0.582700 | 4.822700 |
| C | -7.431100 | 0.416900 | 4.622200 |
| C | -2.371100 | 1.678300 | 3.204300 |
| O | -1.072600 | 1.942000 | 3.766300 |
| C | -0.872500 | 1.551200 | 5.109600 |
| O | -1.912100 | 1.996600 | 5.954900 |
| C | -3.546500 | 3.671000 | 4.401500 |
| O | -4.134800 | 3.721600 | 5.666500 |
| C | -0.733000 | 0.029800 | 5.182000 |
| C | 0.359500 | 2.300300 | 5.563700 |
| O | -4.304600 | 4.263500 | 3.422200 |
| C | -9.522700 | -0.831900 | 3.923600 |
| O | -8.802700 | -2.426600 | 6.288800 |
| O | -5.133300 | -0.117700 | 7.296400 |
| H | -4.614900 | 2.264800 | 7.141100 |
| H | -6.252700 | 2.616000 | 5.002200 |
| H | -3.324400 | 0.521100 | 5.602100 |
| H | -6.808000 | -1.782700 | 7.457100 |
| H | -7.516600 | 1.104000 | 3.788600 |
| H | -2.474600 | 0.620000 | 2.964800 |
| H | -2.455000 | 2.198900 | 2.250300 |
| H | -2.544000 | 4.106100 | 4.486600 |
| H | -1.581900 | -0.488100 | 4.742700 |
| H | 0.162900 | -0.277900 | 4.643400 |
| H | -0.636100 | -0.276900 | 6.223500 |
| H | 0.189400 | 3.373900 | 5.483200 |
| H | 0.580300 | 2.056500 | 6.602700 |
| H | 1.213100 | 2.030200 | 4.942500 |
| H | -4.305600 | 5.208600 | 3.591600 |
| H | -9.581800 | -0.121600 | 3.098700 |
| H | -9.479000 | -1.836400 | 3.498500 |
| H | -10.447500 | -0.758200 | 4.498600 |

LNA-OHAcet_3 Eopt
-1180.047857

| | | | |
|---|---|---|---|
| C | -4.186100 | 1.911300 | 6.529900 |
| C | -5.506700 | 2.214800 | 5.792000 |
| O | -5.048300 | 2.655100 | 4.528000 |
| C | -3.612100 | 2.710000 | 4.614400 |
| C | -3.367600 | 1.430000 | 5.336100 |
| N | -6.560700 | 1.206500 | 5.660000 |
| C | -7.789900 | 1.648900 | 5.199700 |
| N | -8.677700 | 0.653700 | 4.920400 |
| C | -8.540600 | -0.692300 | 5.017300 |
| C | -7.225000 | -1.109300 | 5.512600 |
| C | -6.319300 | -0.148300 | 5.801400 |
| C | -2.826100 | 2.695500 | 3.300200 |
| O | -1.452600 | 2.340700 | 3.540000 |
| C | -1.216300 | 1.207800 | 4.350000 |
| O | -1.968300 | 1.235300 | 5.545700 |
| C | -3.201400 | 3.781300 | 5.634700 |
| O | -3.580100 | 3.175900 | 6.837400 |
| C | -1.524900 | -0.058400 | 3.550000 |
| C | 0.227300 | 1.321700 | 4.783500 |
| O | -3.891700 | 4.930300 | 5.350600 |
| C | -6.951300 | -2.599400 | 5.681600 |
| O | -9.465600 | -1.436800 | 4.706100 |
| O | -8.132000 | 2.817400 | 5.027600 |
| H | -4.286900 | 1.244300 | 7.386000 |
| H | -5.998400 | 3.041000 | 6.319000 |
| H | -3.782500 | 0.552700 | 4.838800 |
| H | -9.572900 | 0.962400 | 4.582100 |
| H | -5.346600 | -0.410500 | 6.182900 |
| H | -3.271700 | 2.021200 | 2.568900 |
| H | -2.857500 | 3.682800 | 2.838300 |
| H | -2.117300 | 3.939700 | 5.655700 |
| H | -2.534800 | -0.062300 | 3.147800 |
| H | -0.832400 | -0.131800 | 2.711400 |
| H | -1.392300 | -0.933400 | 4.185900 |
| H | 0.372400 | 2.246400 | 5.342600 |
| H | 0.493300 | 0.479700 | 5.422100 |
| H | 0.881400 | 1.331800 | 3.911900 |
| H | -4.789800 | 4.664400 | 5.128300 |
| H | -5.951100 | -2.804700 | 6.063100 |
| H | -7.671000 | -3.041600 | 6.373300 |
| H | -7.058800 | -3.115700 | 4.725700 |

LNA-OHAcet_4 Eopt
-1180.046783

| | | | |
|---|---|---|---|
| C | -4.396300 | 1.986300 | 6.073900 |
| C | -5.470200 | 1.796700 | 4.987500 |
| O | -4.771800 | 2.042400 | 3.785800 |
| C | -3.438500 | 2.415300 | 4.195800 |
| C | -3.196600 | 1.450700 | 5.312000 |
| N | -6.316200 | 0.601000 | 4.988900 |
| C | -5.912700 | -0.546000 | 4.323100 |
| N | -6.805500 | -1.573600 | 4.392300 |
| C | -8.010000 | -1.649600 | 5.011400 |
| C | -8.384000 | -0.416300 | 5.709800 |
| C | -7.525900 | 0.626500 | 5.659700 |
| C | -2.319600 | 2.251500 | 3.163700 |
| O | -1.031500 | 2.319600 | 3.802100 |
| C | -0.850400 | 1.521800 | 4.954300 |
| O | -1.904700 | 1.674800 | 5.882800 |
| C | -3.500600 | 3.765500 | 4.929700 |
| O | -4.122400 | 3.396500 | 6.131300 |
| C | -0.705200 | 0.056700 | 4.538200 |
| C | 0.371600 | 2.086600 | 5.641800 |
| O | -4.238300 | 4.631800 | 4.166100 |
| C | -9.711300 | -0.377700 | 6.458100 |
| O | -8.672700 | -2.681400 | 4.957400 |
| O | -4.873800 | -0.735800 | 3.695000 |
| H | -4.632500 | 1.510100 | 7.025700 |
| H | -6.192600 | 2.613800 | 5.051900 |
| H | -3.285300 | 0.396300 | 5.063500 |
| H | -6.531100 | -2.410000 | 3.906400 |
| H | -7.744100 | 1.558000 | 6.166000 |
| H | -2.422200 | 1.320200 | 2.605500 |
| H | -2.382800 | 3.044100 | 2.418100 |
| H | -2.504600 | 4.157500 | 5.165100 |
| H | -1.546700 | -0.294500 | 3.945100 |
| H | 0.197200 | -0.061200 | 3.938700 |
| H | -0.616700 | -0.567800 | 5.426800 |
| H | 0.197800 | 3.130300 | 5.904000 |
| H | 0.579700 | 1.525000 | 6.552100 |
| H | 1.234900 | 2.028800 | 4.979100 |
| H | -4.927600 | 4.104400 | 3.752400 |
| H | -9.889600 | 0.578700 | 6.950000 |
| H | -10.539600 | -0.560700 | 5.770700 |
| H | -9.741500 | -1.158200 | 7.220900 |

LNA-OHAcet_5 Eopt
-1180.048033

| | | | |
|---|---|---|---|
| C | -4.384400 | 2.376400 | 6.398900 |
| C | -5.641400 | 2.420300 | 5.501100 |
| O | -5.075300 | 2.570700 | 4.210400 |
| C | -3.645900 | 2.601200 | 4.389700 |
| C | -3.497300 | 1.571800 | 5.464700 |
| N | -6.587200 | 1.303600 | 5.580000 |
| C | -7.254600 | 1.140200 | 6.780500 |
| N | -7.963900 | -0.019300 | 6.878600 |
| C | -8.077600 | -1.049600 | 6.003200 |
| C | -7.320300 | -0.847600 | 4.763800 |
| C | -6.623500 | 0.302200 | 4.625800 |
| C | -2.771700 | 2.200500 | 3.185400 |
| O | -2.012700 | 1.002900 | 3.431500 |
| C | -1.374100 | 0.900100 | 4.692700 |
| O | -2.107800 | 1.456400 | 5.764300 |
| C | -3.267600 | 3.905300 | 5.105000 |
| O | -3.799100 | 3.688300 | 6.376600 |
| C | -1.225400 | -0.582800 | 4.963800 |
| C | -0.074200 | 1.677800 | 4.622300 |
| O | -3.845500 | 4.947600 | 4.432100 |
| C | -7.359500 | -1.939200 | 3.700800 |
| O | -8.760200 | -2.030300 | 6.284300 |
| O | -7.238000 | 1.920200 | 7.730100 |
| H | -4.559100 | 1.967700 | 7.395300 |
| H | -6.253700 | 3.300000 | 5.725600 |
| H | -3.891400 | 0.581800 | 5.219600 |
| H | -8.465700 | -0.145200 | 7.741000 |
| H | -6.057500 | 0.509300 | 3.728600 |
| H | -3.404500 | 2.020300 | 2.316500 |
| H | -2.102500 | 3.007900 | 2.885200 |
| H | -2.185000 | 4.028300 | 5.209400 |
| H | -2.200600 | -1.067400 | 4.972300 |
| H | -0.613300 | -1.041500 | 4.187500 |
| H | -0.746000 | -0.735900 | 5.930600 |
| H | -0.267200 | 2.736300 | 4.457500 |
| H | 0.471600 | 1.566800 | 5.559300 |
| H | 0.541400 | 1.298000 | 3.807200 |
| H | -4.761200 | 4.697100 | 4.280300 |
| H | -6.764100 | -1.689900 | 2.822300 |
| H | -6.982000 | -2.879600 | 4.106700 |
| H | -8.386000 | -2.112500 | 3.372200 |

LNA-OHAcet_6 Eopt
-1180.042438

| | | | |
|---|---|---|---|
| C | -4.233400 | 1.903300 | 6.460300 |
| C | -5.545100 | 2.173800 | 5.691300 |
| O | -5.075600 | 2.540100 | 4.412700 |
| C | -3.646700 | 2.666700 | 4.532500 |
| C | -3.381200 | 1.412900 | 5.291000 |
| N | -6.604000 | 1.164500 | 5.611200 |
| C | -7.866000 | 1.597000 | 5.236700 |
| N | -8.761800 | 0.594100 | 5.016600 |
| C | -8.610600 | -0.750400 | 5.111200 |
| C | -7.263300 | -1.156500 | 5.522600 |
| C | -6.347100 | -0.188200 | 5.745900 |
| C | -2.837800 | 2.647500 | 3.234600 |
| O | -1.458700 | 2.336300 | 3.507000 |
| C | -1.209100 | 1.224500 | 4.342300 |
| O | -1.979500 | 1.258900 | 5.526300 |
| C | -3.274400 | 3.767500 | 5.544600 |
| O | -3.645900 | 3.179500 | 6.756600 |
| C | -1.475900 | -0.064300 | 3.563300 |
| C | 0.224900 | 1.379800 | 4.794600 |
| O | -3.973300 | 4.910700 | 5.251800 |
| C | -6.969800 | -2.644000 | 5.682800 |
| O | -9.549300 | -1.502900 | 4.866300 |
| O | -8.240700 | 2.758900 | 5.094300 |
| H | -4.343700 | 1.247600 | 7.323600 |
| H | -6.026700 | 3.046900 | 6.147400 |
| H | -3.765200 | 0.514600 | 4.807400 |
| H | -9.678800 | 0.897500 | 4.737600 |
| H | -5.350100 | -0.441400 | 6.063300 |
| H | -3.252800 | 1.947000 | 2.510000 |
| H | -2.892500 | 3.624200 | 2.753200 |
| H | -2.191200 | 3.930900 | 5.574500 |
| H | -2.479600 | -0.100400 | 3.147300 |
| H | -0.770100 | -0.137500 | 2.735900 |
| H | -1.332300 | -0.923300 | 4.218400 |
| H | 0.341200 | 2.318000 | 5.337300 |
| H | 0.499800 | 0.556300 | 5.453200 |
| H | 0.891400 | 1.387200 | 3.932300 |
| H | -3.737600 | 5.573600 | 5.904700 |
| H | -5.945500 | -2.840300 | 5.999400 |
| H | -7.640400 | -3.085700 | 6.422400 |
| H | -7.135900 | -3.166400 | 4.738700 |

LNA-OHAcet_7 Eopt
-1180.043813

| | | | |
|---|---|---|---|
| C | -4.421400 | 2.331700 | 6.347100 |
| C | -5.670000 | 2.353800 | 5.435600 |
| O | -5.093200 | 2.456800 | 4.147800 |
| C | -3.671800 | 2.560200 | 4.340900 |
| C | -3.506600 | 1.543000 | 5.424100 |
| N | -6.624900 | 1.249200 | 5.543300 |
| C | -7.297500 | 1.126300 | 6.745600 |
| N | -8.016800 | -0.024200 | 6.874300 |
| C | -8.133300 | -1.079900 | 6.030100 |
| C | -7.369400 | -0.920600 | 4.788700 |
| C | -6.663700 | 0.219600 | 4.619700 |
| C | -2.776500 | 2.178700 | 3.148300 |
| O | -1.983200 | 1.005500 | 3.406900 |
| C | -1.358700 | 0.921700 | 4.676200 |
| O | -2.116400 | 1.466300 | 5.737000 |
| C | -3.338900 | 3.880000 | 5.059800 |
| O | -3.855900 | 3.651900 | 6.333700 |
| C | -1.180400 | -0.556500 | 4.955200 |
| C | -0.076000 | 1.727900 | 4.617100 |
| O | -3.938900 | 4.922300 | 4.401400 |
| C | -7.413400 | -2.042300 | 3.757700 |
| O | -8.823100 | -2.047600 | 6.338400 |
| O | -7.277400 | 1.929800 | 7.675900 |
| H | -4.600200 | 1.919100 | 7.340800 |
| H | -6.275100 | 3.248600 | 5.614100 |
| H | -3.877100 | 0.543200 | 5.181400 |
| H | -8.522800 | -0.120200 | 7.737800 |
| H | -6.093900 | 0.399100 | 3.718900 |
| H | -3.398100 | 1.976400 | 2.276100 |
| H | -2.131100 | 3.004900 | 2.849000 |
| H | -2.259500 | 4.021200 | 5.174100 |
| H | -2.144900 | -1.062200 | 4.953200 |
| H | -0.549100 | -1.004500 | 4.188100 |
| H | -0.710100 | -0.695600 | 5.928500 |
| H | -0.291200 | 2.780000 | 4.440100 |
| H | 0.459600 | 1.636200 | 5.562000 |
| H | 0.558500 | 1.355400 | 3.813100 |
| H | -3.794300 | 5.715100 | 4.923900 |
| H | -6.814500 | -1.821100 | 2.874000 |
| H | -7.042600 | -2.972900 | 4.191300 |
| H | -8.440200 | -2.217900 | 3.431500 |

LNA-OHAcet_8 Eopt
-1180.043534

| | | | |
|---|---|---|---|
| C | -4.378700 | 2.410900 | 6.188300 |
| C | -5.520600 | 1.945800 | 5.245600 |
| O | -4.849100 | 1.883700 | 4.000300 |
| C | -3.480700 | 2.265400 | 4.239500 |
| C | -3.250300 | 1.608900 | 5.562200 |
| N | -6.288700 | 0.710900 | 5.433700 |
| C | -6.140100 | -0.079600 | 6.561700 |
| N | -7.056200 | -1.083800 | 6.667600 |
| C | -8.092000 | -1.415000 | 5.856700 |
| C | -8.214900 | -0.542600 | 4.685600 |
| C | -7.318700 | 0.458800 | 4.547000 |
| C | -2.417900 | 1.765300 | 3.241000 |
| O | -1.458200 | 0.881200 | 3.847800 |
| C | -0.962400 | 1.251300 | 5.122700 |
| O | -1.907400 | 1.895800 | 5.950900 |
| C | -3.446000 | 3.759600 | 4.583800 |
| O | -4.071700 | 3.769200 | 5.830300 |
| C | -0.562700 | -0.040200 | 5.806100 |
| C | 0.161200 | 2.247300 | 4.912200 |
| O | -4.132400 | 4.438700 | 3.613400 |
| C | -9.335400 | -0.813500 | 3.688700 |
| O | -8.819600 | -2.364000 | 6.134000 |
| O | -5.300500 | 0.018500 | 7.451000 |
| H | -4.568100 | 2.279000 | 7.253200 |
| H | -6.298800 | 2.713600 | 5.240900 |
| H | -3.411400 | 0.527100 | 5.576900 |
| H | -6.956200 | -1.669900 | 7.478500 |
| H | -7.338300 | 1.131600 | 3.698500 |
| H | -2.903700 | 1.219400 | 2.432400 |
| H | -1.900600 | 2.593200 | 2.754900 |
| H | -2.427000 | 4.137300 | 4.716000 |
| H | -1.422200 | -0.703300 | 5.896300 |
| H | 0.209300 | -0.544100 | 5.225100 |
| H | -0.176700 | 0.172900 | 6.802800 |
| H | -0.213100 | 3.154600 | 4.441700 |
| H | 0.603100 | 2.510600 | 5.873200 |
| H | 0.930400 | 1.808900 | 4.276900 |
| H | -4.933700 | 3.934400 | 3.449600 |
| H | -9.329400 | -0.115000 | 2.851900 |
| H | -9.247200 | -1.823300 | 3.283200 |
| H | -10.307400 | -0.741600 | 4.180400 |

## 7.0 References


[1] F. Mohamadi, N. G. J. Richards, W. C. Guida, R. Liskamp, M. Lipton, C. Caufield, G. Chang, T. Hendrickson, W. C. Still, *J Comput Chem* **1990**, *11*, 440–467.

[2] N. MacroModel Schrödinger, LLC, New York, **2014**.

[3] **2020**.

[4] G. Chang, W. C. Guida, W. C. Still, *J Am Chem Soc* **1989**, *111*, 4379–4386.

[5] E. Harder, W. Damm, J. Maple, C. Wu, M. Reboul, J. Y. Xiang, L. Wang, D. Lupyan, M. K. Dahlgren, J. L. Knight, J. W. Kaus, D. S. Cerutti, G. Krilov, W. L. Jorgensen, R. Abel, R. A. Friesner, *J Chem Theory Comput* **2016**, *12*, 281–296.

[6] T. Lewis-Atwell, P. A. Townsend, M. N. Grayson, *Journal of Organic Chemistry* **2022**, *87*, 5703–5712.

[7] M. J. Frisch, G. W. Trucks, H. B. Schlegel, G. E. Scuseria, M. a. Robb, J. R. Cheeseman, G. Scalmani, V. Barone, G. a. Petersson, H. Nakatsuji, X. Li, M. Caricato, a. V. Marenich, J. Bloino, B. G. Janesko, R. Gomperts, B. Mennucci, H. P. Hratchian, J. V. Ortiz, a. F. Izmaylov, J. L. Sonnenberg, Williams, F. Ding, F. Lipparini, F. Egidi, J. Goings, B. Peng, A. Petrone, T. Henderson, D. Ranasinghe, V. G. Zakrzewski, J. Gao, N. Rega, G. Zheng, W. Liang, M. Hada, M. Ehara, K. Toyota, R. Fukuda, J. Hasegawa, M. Ishida, T. Nakajima, Y. Honda, O. Kitao, H. Nakai, T. Vreven, K. Throssell, J. a. Montgomery Jr., J. E. Peralta, F. Ogliaro, M. J. Bearpark, J. J. Heyd, E. N. Brothers, K. N. Kudin, V. N. Staroverov, T. a. Keith, R. Kobayashi, J. Normand, K. Raghavachari, a. P. Rendell, J. C. Burant, S. S. Iyengar, J. Tomasi, M. Cossi, J. M. Millam, M. Klene, C. Adamo, R. Cammi, J. W. Ochterski, R. L. Martin, K. Morokuma, O. Farkas, J. B. Foresman, D. J. Fox, **2016**, Gaussian 16, Revision A.01, Gaussian, Inc., Wallin.

[8] J. Da Chai, M. Head-Gordon, *Physical Chemistry Chemical Physics* **2008**, *10*, 6615–6620.

[9] R. Ditchfield, W. J. Hehre, J. A. Pople, *J Chem Phys* **1971**, *54*, 720–723.

[10] F. Weigend, R. Ahlrichs, *Physical Chemistry Chemical Physics* **2005**, *7*, 3297–3305.

[11] F. Weigend, *Physical Chemistry Chemical Physics* **2006**, *8*, 1057–1065.

[12] A. V Marenich, C. J. Cramer, D. G. Truhlar, *J Phys Chem B* **2009**, *113*, 6378–96.

[13] A. Armstrong, R. A. Boto, P. Dingwall, J. Contreras-García, M. J. Harvey, N. J. Mason, H. S. Rzepa, *Chem Sci* **2014**, *5*, 2057–2071.

[14] Q. Peng, F. Duarte, R. S. Paton, *Chem Soc Rev* **2016**, *45*, 6093–6107.

[15] M. Bursch, J.-M. Mewes, A. Hansen, S. Grimme, *Angewandte Chemie* **2022**, *134*, e202205735.

[16] S. Schenker, C. Schneider, S. B. Tsogoeva, T. Clark, *J Chem Theory Comput* **2011**, *7*, 3586–3595.

[17] S. Grimme, *Chemistry – A European Journal* **2012**, *18*, 9955–9964.

[18] G. Luchini, J. V Alegre-Requena, I. Funes-Ardoiz, R. S. Paton, R. Pollice, *F1000Research 2020 9:291* **2020**, *9*, 291.

[19] C. Y. Legault, **2009**, CYLview, 1.0b.
